# Dynamics and determinants of land change in India: integrating satellite data with village socioeconomics

**DOI:** 10.1007/s10113-016-1068-2

**Published:** 2016-10-27

**Authors:** Prasanth Meiyappan, Parth S. Roy, Yeshu Sharma, Reshma M. Ramachandran, Pawan K. Joshi, Ruth S. DeFries, Atul K. Jain

**Affiliations:** 1 Department of Atmospheric Sciences, University of Illinois, Urbana, Urbana, IL 61801 USA; 2 University Center for Earth and Space Science, University of Hyderabad, Hyderabad, 500046 India; 3 Lab for Spatial Informatics, International Institute of Information Technology, Hyderabad, 500032 India; 4 School of Environmental Sciences, Jawaharlal Nehru University, New Delhi, 110067 India; 5 Department of Ecology, Evolution, and Environmental Biology, Columbia University, New York, NY 10027 USA

**Keywords:** Land use change, Drivers, Causes, Deforestation, Agriculture, Food security

## Abstract

We examine the dynamics and spatial determinants of land change in India by integrating decadal land cover maps (1985–1995–2005) from a wall-to-wall analysis of Landsat images with spatiotemporal socioeconomic database for ~630,000 villages in India. We reinforce our results through collective evidence from synthesis of 102 case studies that incorporate field knowledge of the causes of land change in India. We focus on cropland–fallow land conversions, and forest area changes (excludes non-forest tree categories including commercial plantations). We show that cropland to fallow conversions are prominently associated with lack of irrigation and capital, male agricultural labor shortage, and fragmentation of land holdings. We find gross forest loss is substantial and increased from ~23,810 km^2^ (1985–1995) to ~25,770 km^2^ (1995–2005). The gross forest gain also increased from ~6000 km^2^ (1985–1995) to ~7440 km^2^ (1995–2005). Overall, India experienced a net decline in forest by ~18,000 km^2^ (gross loss–gross gain) consistently during both decades. We show that the major source of forest loss was cropland expansion in areas of low cropland productivity (due to soil degradation and lack of irrigation), followed by industrial development and mining/quarrying activities, and excessive economic dependence of villages on forest resources.

**Electronic supplementary material:**

The online version of this article (doi:10.1007/s10113-016-1068-2) contains supplementary material, which is available to authorized users.

## Introduction

India’s per capita land availability is ~0.25 ha per person compared to the global average of ~2.3 ha per person (Census of India 2011). India’s cattle density is ~62 heads per km^2^ compared to the global average of ~10 heads per km^2^ (Robinson et al. 2014). This high human and animal pressure, coupled with increasing standards of living (Hubacek et al. 2007; United Nations 2014; World Bank Group 2015), has placed tremendous pressure on India’s land resources for food, fiber, fuel, and shelter causing extensive environmental degradation (Table 1).

**Table 1:** Comparison by numbers: the role of land-use and land-cover change (LULCC) on key environmental problems compared between world and India for present/past period

Environmental problem	Role of LULCC	
	World	India
Human land use	55% of land area	83% of land area
Climate change	20–24% of GHG emissions	25–30% of GHG emissions
Biodiversity loss	14% of species richness	22% of species richness
Land degradation	8–41% of land area	~57% of land area
Water use for agriculture	70% of withdrawal	91% of withdrawal
Nutrient excess in crops (water pollution)	56% of nitrogen; 48% of phosphorous	74% of nitrogen; 71% of phosphorous

The comparison indicates that LULCC contribution to environmental problems in India is of greater magnitude compared to global case. See Table S1 for details

The pressure on India’s land resources is expected to further intensify in the future, with the growing economy (Hubacek et al. 2007; United Nations 2014; World Bank Group 2015) and human population (United Nations 2015), expected increase in demands for animal products (Alexandratos and Bruinsma 2012), and climate change (Singh et al. 2002; Krishna Kumar et al. 2004; O’Brien et al. 2004; Lobell et al. 2008, 2012; Auffhammer et al. 2012; MoEFCC 2015). Therefore, a key challenge for land use planning in India is to enhance food production and simultaneously minimize environmental degradation from land-use and land-cover change (LULCC). Land in India is also closely tied to livelihood security as over half of India’s population is employed in agriculture and forestry (Census of India 2011). India being one of the ten most forest-rich nations of the world, has received increasing attention under the REDD+ (Reducing Emissions from Deforestation and Forest Degradation) mechanism to protect its forests to help mitigate climate change, preserve its rich biodiversity, and support ecosystem services (Agrawal et al. 2011; Ravindranath et al. 2012; MoEFCC 2015). For similar reasons, India’s national forest policy aims to increase its forest cover from the existing ~21% of its total geographical area to a minimum of 33% (MoEF 1988; Joshi et al. 2011). Better monitoring and understanding of the determinants and drivers of LULCC at national scale is crucial to: (1) better understand their environmental and socioeconomic impacts and (2) provide valuable guidance for land use policies toward addressing the future challenges for LULCC in India.

There are three aspects to our study. *First*, we quantified land cover conversions (complete replacement of one land cover by another) at national scale using a wall-to-wall analysis of high-resolution (~30 m) Landsat MSS/TM imageries at decadal time intervals (1985–1995 and 1995–2005). Importantly, our study period (1985–2005) includes the period of economic liberalization in India (1991 onwards) following which the pressure on land resources intensified. We report LULCC estimates at national (Tables S2-S4) and state level (Table S5; Dataset S1), and by agro-ecological zones (AEZs) (Table S6; Dataset S2) considering their policy relevance to forest and agriculture (see Text S1 for rationale). AEZs are regions delineated by similar climatic and soil conditions (Velayutham et al. 1999; Gajbhiye and Mandal 2000). In Indian context, AEZs are the optimal units for macro-level land use planning and efficient transfer of technology, as India’s economy is highly dependent on agriculture and forestry (Velayutham et al. 1999; Gajbhiye and Mandal 2000).

*Second*, we investigated the spatial determinants (defined following Meyfroidt 2015) of three broad LULCC that are central to land use planning in India (Saxena 2006; Maji et al. 2010; DoLR 2013; MoEFCC 2014, 2015): cropland–fallow land conversions, forest area losses, and forest area gains. Our forest definition is consistent with IGBP land classification scheme (Belward 1996) and excludes non-forest tree categories such as commercial plantations of coconut, cashew, coffee and rubber, and fruit orchards (see Table S7 for land class definitions). Cropland area refers to area under crops in any of the three prominent cropping seasons of India (summer monsoon, winter, and summer). We only account for net cropped area, i.e., multiple cropping is counted once. Fallow land refers to land taken up for cultivation, but temporarily allowed to rest, un-cropped across all three cropping seasons. Fallow is typically unproductive agricultural land, but may provide important services, e.g., nutrient replenishment, use by livestock and wildlife, and groundwater recharge. As per capita land is low in India, understanding cropland–fallow land conversions is crucial to plan and evaluate agricultural development efforts to improve food security (Saxena 2006; Maji et al. 2010). We do not classify cropland and fallow land into further sub-categories based on seasons (e.g., rabi, kharif, zaid).

*Third*, we evaluate and reinforce our modeled results on spatial determinants through collective evidence from synthesis of 102 case studies (see Table S8-S11 for study-wise summary; Text S1 for methods) that incorporate field knowledge of the causes of LULCC mainly through social surveys and local expertise. While ground studies (social surveys, local expertise) offer crucial qualitative insights, data collection is typically expensive and therefore covers small regions. It is hard to generalize and quantify the causal relations of LULCC by studying few villages in a country of over 600,000 villages with diverse agro-ecological and sociocultural environment. Our synthesis helps to identify accumulated effects that are statistically stronger than any individual case study due to increased sample size and greater diversity. It is important to note that the case studies often relate to the triggers of the change (see Meyfroidt 2015) as opposed to the location factors (spatial determinants) identified through our modeling analysis (second aspect). Therefore, while both our modeled results and synthesis of case studies are complimentary and inform each other, the characteristic of information provided by them are different.

Our study differs from existing satellite-based national assessments of LULCC in India on two aspects. *First*, our land cover conversion estimates rely on Landsat analysis that covers longer time period, uses uniform classification scheme, maps patch to patch land dynamics, and is validated using ground data (Roy et al. 2015). Earlier high-resolution land cover mapping activities at national scale were one-time effort (see review by Roy et al. 2015) hence unavailable for monitoring at regular time intervals; their project-specific classification scheme and varying data quality make compilation of consistent time series images difficult. Tracking patch-level dynamics is crucial because the environmental impacts vary depending on the preceding and replaced land cover class (Don et al. 2011; Mahmood et al. 2014). Notably, India monitors forest cover including trees outside forest biannually (FSI 2015), but not patch to patch land dynamics. Our land cover maps have been extensively validated with over 12,600 stratified random samples (ground-verified GPS points) distributed uniformly in different land cover classes following Congalton and Green (1999). Our data have an overall mapping accuracy of 95% (across eleven land classes defined in Table S7), thus providing accurate and reliable information on LULCC. See Roy et al. (2015) for further details on validation.

*Second*, this is the first study to use village-level socioeconomic data at national scale to investigate the spatial determinants of LULCC. Villages are the highest level of spatial disaggregation in India (>630,000 administrative units; Fig. S1). Thus far, no geospatial socioeconomic database exists for complete India at village level; our data are a significant improvement in spatial resolution compared to existing national datasets (~5500 administrative units or coarser; see Fig. S2). Overall, we compiled spatial data on over 200 socioeconomic variables for two consecutive census years (1991 and 2001; for use with respective decadal LULCC analysis) (Text S1). The use of village-level data is crucial for two reasons. *First*, it captures the high granularity in socioeconomics (Fig. S2) that is crucial to explain the spatial variations in high-resolution Landsat data. The granularity gets masked at coarser administrative levels (Fig. S2). *Second*, we use over forty village-specific categorical/qualitative variables (Table S12) that cannot be represented at coarser administrative levels (e.g., village-specific primary occupations that reflect the base of the socioeconomic culture prevalent in rural parts of India). We also include key biophysical factors (Text S1) hypothesized to affect the spatiotemporal patterns of land change in India (Table S12).

## Methods

Here, we describe our methods and data briefly. See Text S1 for further details.

### Data

Table S13 summarizes key datasets used with references. We highlight socioeconomic and LULUC data, both of which are central to our analysis.

We created the spatial socioeconomic database by combining tabular information from the Indian census (both 1991 and 2001; each household is surveyed and aggregated to village/town level) with seamless village- and town-level administrative boundaries of India corresponding to 2001 census specifically prepared for this study, sourced from Survey of India topographic sheets (analog maps). Both the tabular data and administrative boundaries required substantial amount of organization, data cleaning, and quality checks prior to being linked together.

We have a dedicated article describing the technical details and validation of the LULCC database, with basic land cover area statistics (Roy et al. 2015). In contrast, this study presents detailed land conversion analysis of the LULCC database. What follows is a summary. Our data have ~30 m resolution, with features mapped at 1:50,000 scale. We mapped the entire country using on-screen visual interpretation of satellite data for two decades (1985–1995–2005). Our land types are defined following the IGBP land classification scheme (Belward 1996; see Table S7 for definitions). We projected the multitemporal Landsat MSS/TM data to WGS84 datum (UTM 44N projection) at sub-pixel level. We used satellite images from three seasons, viz. winter (January–March), summer (April–June), and summer monsoon (mid-October to December) to identify cropland and fallow land (we do not capture multiple cropping). Our analysis does not allow harvested areas as we select images of peak crop growth in a cropping season. When cloud-free Landsat images were unavailable, we used IRS 1C–LISS III (1994–1995) and Resourcesat 1 (2004–2005) images by geometrically correcting them with sub-pixel accuracy, relative to Landsat (ortho-rectified). We used first-order polynomial equation with allowable root-mean-square error of less than one pixel for geometric rectification. The minimum number of ground control points we used to georectify the satellite images was 15 for flat terrains and 30 for hilly terrain. Manual interpretation of detailed Landsat/LISS III images is laborious. Therefore, studies with large spatial coverage typically interpret Landsat images on sampling basis, representative of the study region (e.g., Gibbs et al. 2010). In contrast, our analysis is a wall-to-wall mapping effort at national scale.

### Quantifying land cover conversions

We first interpreted 2005 Landsat scenes to produce a national map of land cover. To minimize errors in land change detection between 2005 and 1995, we overlaid 1995 Landsat images over 2005 map and traced patches where land change had occurred, leaving unchanged patches unmodified (for greater consistency). We preferred this method for two reasons. *First*, it reduces the effort required to produce 1995 map as only patches that underwent change between 1995 and 2005 are traced. *Second*, as patches that remained unchanged over time were preserved, it minimizes errors in land change detection by eliminating human errors in visual interpretation of unmodified patches that can occur if 1995 map were interpreted independent of 2005 map and if land change were inferred by differencing the two maps. We followed similar approach to detect land change between 1985 and 1995, using 1995 map as reference.

### Modeling the determinants of LULCC

We quantify the (spatial) determinants by estimating spatial logistic regressions (Text S1) between land cover conversion estimates (dependent variable) and hypothesized socioeconomic and biophysical factors (or their proxies) grounded through local case studies (Table S12). We estimate regression models (Table S14) specific to land cover conversion and decadal time period, at both national scale and for sub-national hot spots identified by AEZs (Table S6). Our regression analysis is carried out at 1 km × 1 km resolution (see Text S1 for data preprocessing). The 1-km resolution was mainly a tradeoff between the 30-m LULCC data and relatively coarser socioeconomic data (~2 km × 2 km per village on average). To minimize loss of information, while aggregating the 30-m LULUC data, we calculated the fraction of 1-km grid cell undergoing various land cover conversions, as opposed to approximating the entire grid cell area to undergo one (dominant) land cover conversion.

Our statistical modeling technique draws on our recent work (Meiyappan et al., 2014) and is common to land change modeling studies. We model the relationship between dependent and independent variables as a “fractional” binomial logit model (Text S1). The model allows for fractional outcomes in dependent variables, consistent with our LULCC data aggregation technique. As our independent variables have different units and scale, we standardized all continuous variables using z-score prior to estimation. We use a state-of-the-art method, the elastic net penalty for variable selection (account for multicollinearity across independent variables). We used bootstrap resampling with 500 replicates, where we resampled the observations (grid cells) and we fitted a new model to the data. The bootstrap, in addition to providing confidence intervals, also accounts for spatial autocorrelation typical to gridded LULCC datasets.

### Synthesis of case studies

Our synthesis provides a bottom-up analysis on the causes of LULCC in India. Furthermore, we used the synthesis literature to ground our hypothesized socioeconomic and biophysical factors for statistical estimation (Table S12). We performed a systematic literature search on ISI Web of Science and Google Scholar for studies on LULCC covering India and our study period. We additionally included key (sub-) national reports, not indexed in either literature database. In total, we reviewed 643 articles, of which we discarded 177 as irrelevant (38 of which discussed causes of LULCC processes not a focus of our study). Of the remaining 466 articles, over three-fourth focused only on land change detection, highlighting the relatively less attention on understanding the causes of change. The 102 articles in our synthesis provide information on the causes of land change typically by combining one or more of: household surveys, field transects, and regional/local expertise of authors. Often, studies also included remote sensing component. The studies are summarized in Tables S8-S11, and the study locations are visualized in Fig. S3. To quantify the results of our synthesis, we analyzed the frequency of causes across case studies. We grouped the studies by LULCC processes and into broad clusters of causes (see Dataset S3 for study-wise grouping details and Text S1 for detailed methods); the clusters being specific to LULCC process.

## Results

We present the LULCC conversion estimates and spatial determinants in the first three subsections. LULCC conversion estimates are based on analysis of satellite data. All our estimates pertain to the sum of urban, peri-urban, and rural areas within the region of quantification (national level or AEZs as identified). Our results on spatial determinants are based on regression analysis of satellite data with hypothesized biophysical and village socioeconomic variables. We present the results of synthesis from 102 case studies in the fourth subsection.

### Conversions between cropland and fallow land

**Fig. 1:**
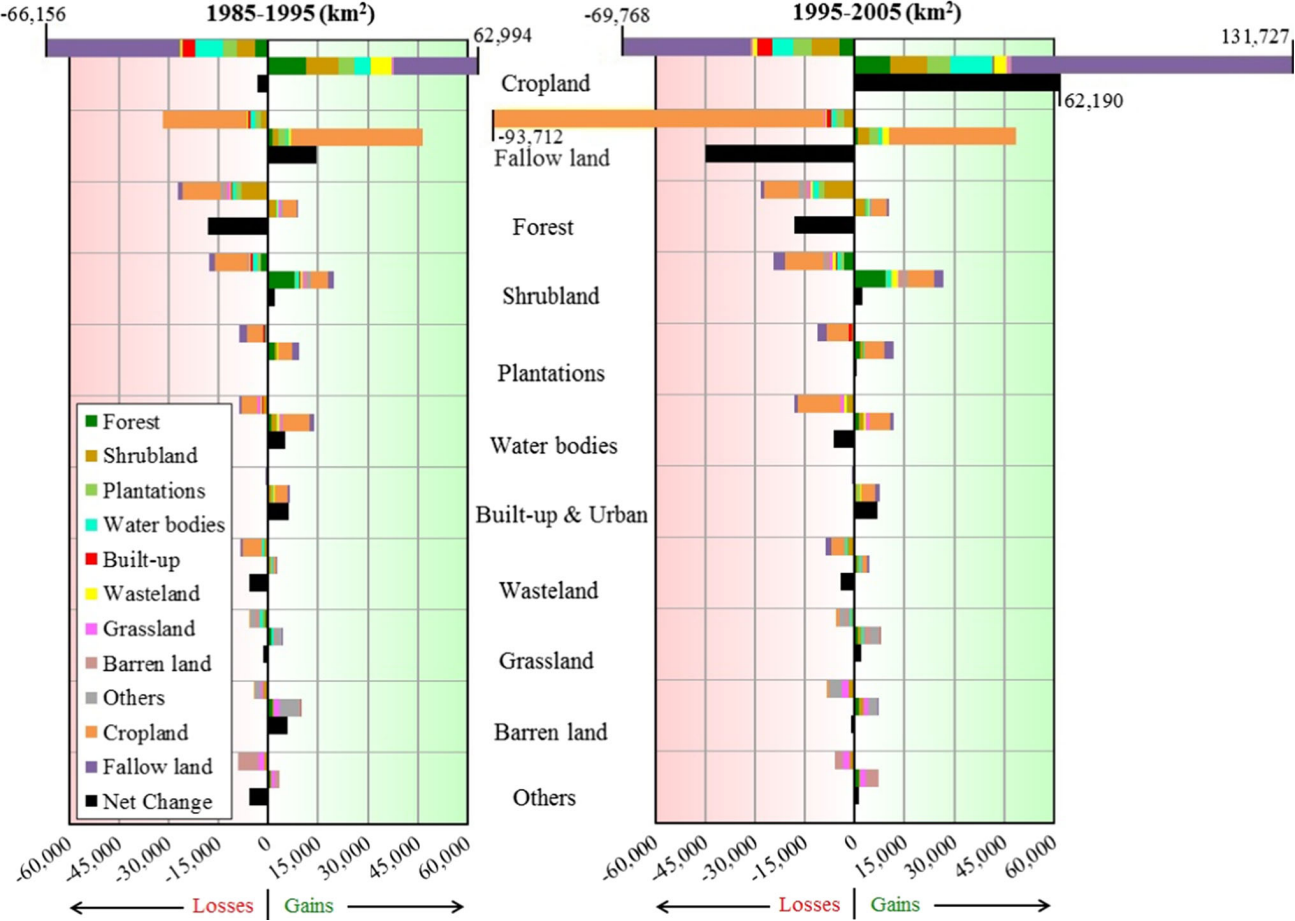
Gross *gains*, gross *losses*, and net changes in land use and land cover areas at aggregate national scale for the two decades (km^2^/decade): 1985–1995 and 1995–2005. Aqua culture and permanent wetlands is included within “Water bodies.” “Others” category include Salt Pan, Snow and Ice. Data from this figure are provided in Table S2-S4 (color figure online)

**Fig. 2:**
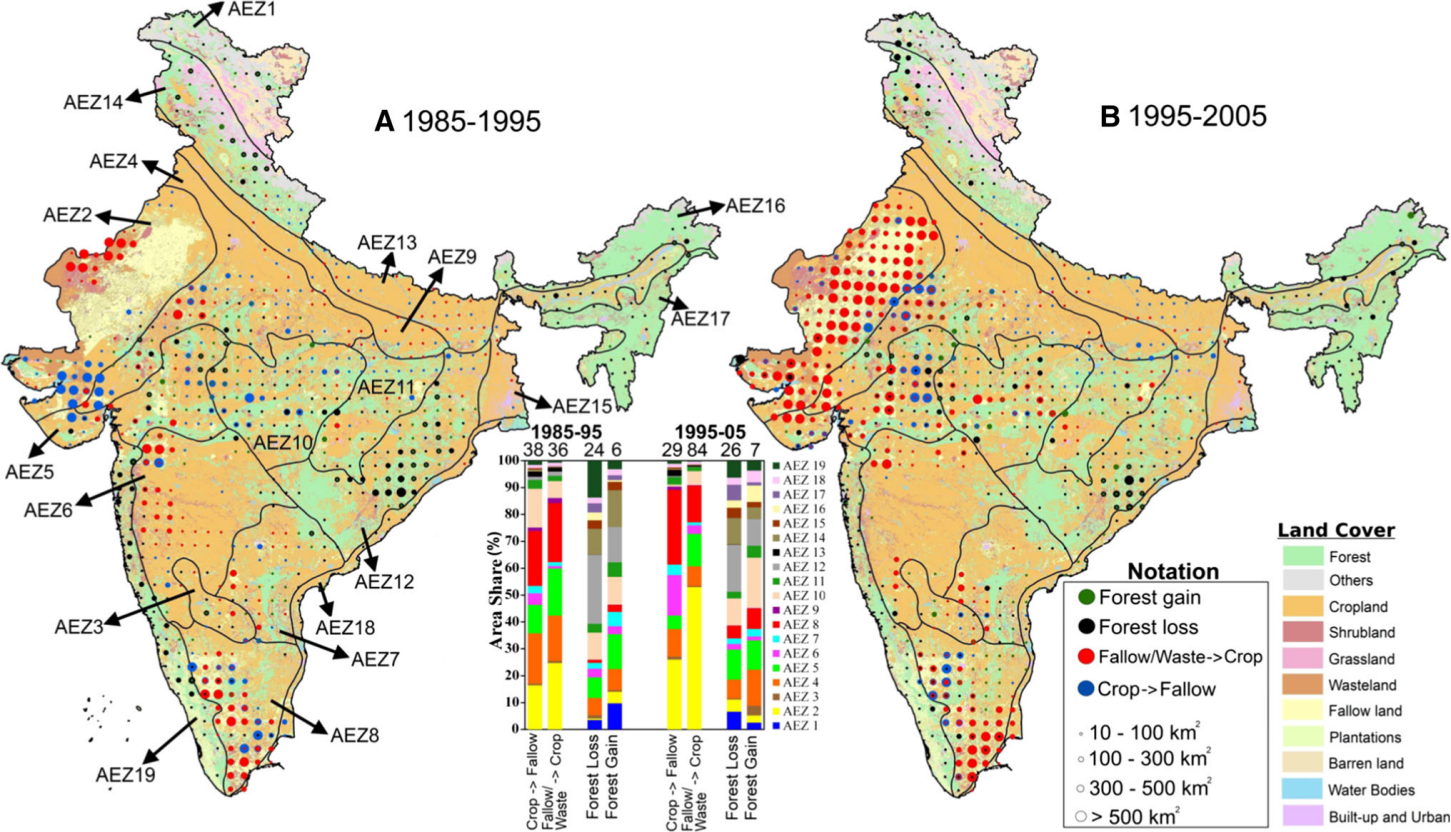
Spatial breakdown of major land cover conversions: forest loss, forest gain, conversions from cropland and fallow land, and reclamation of fallow land and wasteland to cropland. The size of circles is proportional to the magnitude of change. The *inset bar plot* shows the percent contribution by region to the national total (shown besides bar; units in ×1000 km^2^/decade and rounded to nearest integer). The regions are based on agro-ecological zones (AEZs) of India (Table S6). The *background colors* in the map correspond to the type of land cover present at before conversion (see “legends” for color coding). See Fig. S4 for a more detailed breakdown by AEZ (color figure online)

We find major shifts between cropland and fallow land during the period of study (Fig. 1). About 35% (1985–1995) and 46% (1995–2005) of all areas that underwent land cover conversion in India resulted from changes between cropland and fallow land. Furthermore, data suggests that ~10% of existing wastelands (sparsely vegetated land with signs of erosion and land deformation; see Table S7) are consistently reclaimed to cropland during each decade. These development efforts are, however, countered by the much larger amount of cropland being fallowed concurrently. A spatial disaggregation (Fig. 2; Dataset S2) indicates that over 70% of shifts from cropland to fallow land and vice versa are confined to five agro-ecological zones (AEZs): the Western Plain, Kachchh, and part of Kathiawar peninsula (AEZ2), and the semiarid zones (AEZ4, 5, 6, and 8). These five zones also enclose over 90% of wasteland reclaimed to cropland during each decade (Fig. 2; Dataset S2). This indicates that within the same AEZ, wasteland reclamation adds to cropland area on the one hand, and on the other, cropland is being fallowed concomitantly representing a net negative outcome for wasteland reclamation efforts.

Land can be kept under fallow temporarily to restore and maintain soil fertility in multiple cropping systems. However, as our maps are decadal, we cannot identify whether the cropland–fallow conversions observed are a part of land restoration process or not. Therefore, for further insights, we complied annual (1984–2012) district-level ground statistics data on fallow land from the Government of India (Dataset S4). The statistic indicates that in both AEZ2 and AEZ8 (top two regional hot spots of cropland–fallow conversions) the area of long fallows (land not cultivated for 1–5 years) exceeds that of temporary fallows (<1 year). Furthermore, 3.5% of India’s land area was consistently under long fallows over the past decade (Dataset S4).

**Fig. 3:**
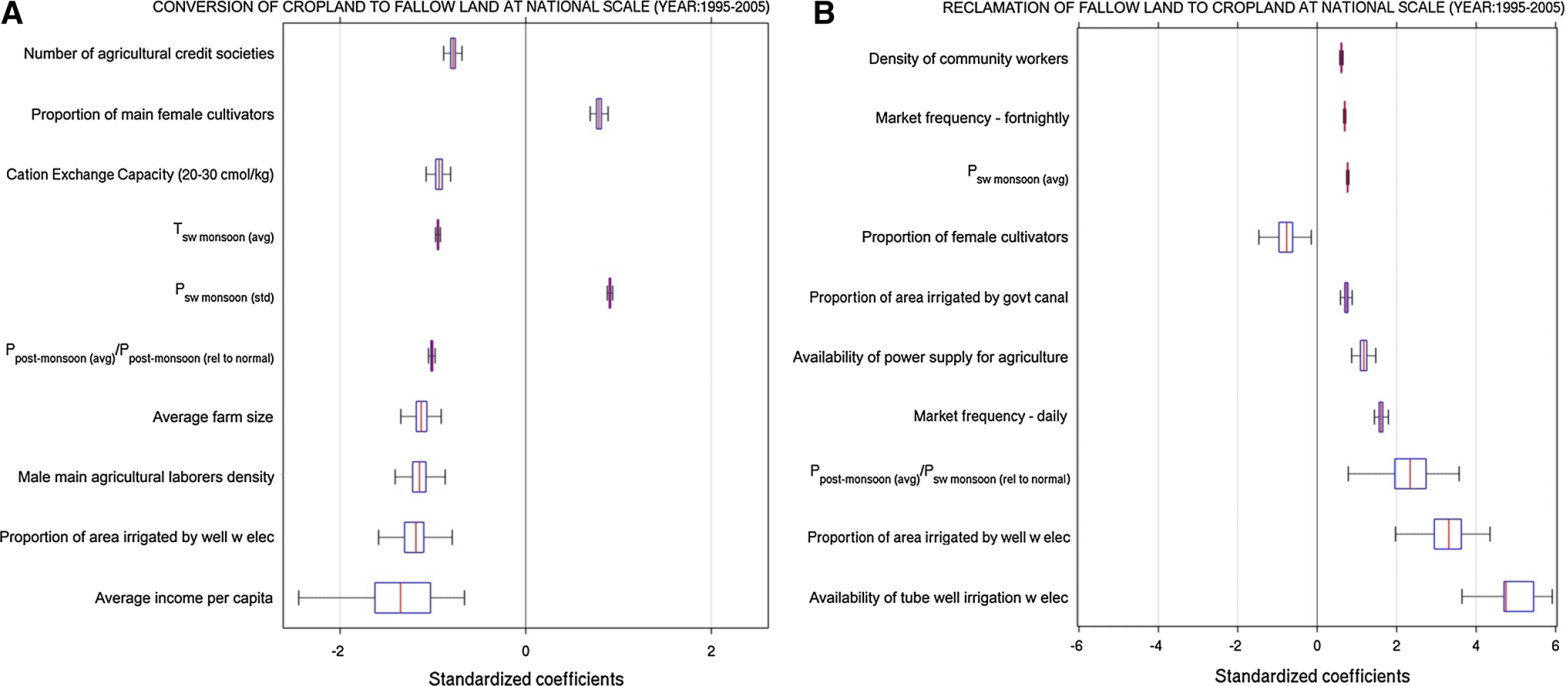
Factors most prominent in explaining: **a** conversion of cropland to fallow land at national scale (1995–2005), and **b** vice versa conversion, i.e., conversion of fallow land to cropland at national scale (1995–2005). The plots show the standardized regression coefficients of the ten most important variables (largest absolute mean estimates across coefficients) estimated using the spatial logistic regression model (see “Methods” section). Standardized coefficients refer to how many standard deviations a dependent variable will change, per standard deviation increase in the independent variable. Standardized coefficients allow comparisons of the relative effects of independent variables measured on different scales. Results from bootstrap resampling with 500 replicates: *central red line* shows mean estimate; *error boxes* (*blue*) show 25–75% confidence interval; *whiskers* show 5–95% confidence interval. See Fig. S5 for national-scale estimates corresponding to 1985–1995. See Table S15 for description of factors (color figure online)

Our regression analysis at national scale (Fig. 3a, S5a) indicates higher monsoon and post-monsoon precipitation is negatively associated with conversion from cropland to fallow land, echoing previous studies (e.g., Krishna Kumar et al. 2004; Lobell et al. 2008; Auffhammer et al. 2012) (see Table S15 for a description of all biophysical and socioeconomic variables). Post-liberalization period, we observe widespread spatial changes in main male agricultural (wage) laborers and male marginal cultivators (main + marginal) (Fig. S6), primarily driven by urbanization and better income opportunities (relatively less strenuous and more stable non-agricultural jobs) (Mitra and Murayama 2009; Srivastava 2011). During 1995–2005, we find areas converted from cropland to fallow land had substantially lower male main agricultural labor (AEZ2) and total (main + marginal) male marginal cultivators (semiarid hot spots) compared to counterfactual buffer villages (Fig. S7b). These results imply that availability of labor is an emerging factor in determining fallow land. We also find positive association between fallow land and proportion of main female cultivators, indicating gender-biased labor markets (Shiferaw et al. 2006; Gupta and Sharma 2010; Shah 2010; Singh et al. 2011).

Factors prominent in explaining conversion from cropland to fallow land (Fig. 3a, S5a, S7-S10) often were also prominent in explaining vice versa conversion (i.e., reclamation of fallow land to cropland), but with opposite sign (Fig. 3b, S5b, S11-S13). At national scale, the following factors show prominent positive association with reducing fallow land (in decreasing order of importance based on Fig. 3b, S5b): availability of tube well and well irrigation with electricity; higher monsoon and post-monsoonal rainfall; increased market frequency; availability of power supply for agriculture; density of community workers (proxy for technical assistance and incentives for agriculture); availability of communication facility (e.g., bus, trains; proxy for connectivity to markets); and availability of agricultural credit institutions, and higher average income per capita (both indicating access to capital and ability to invest).

In AEZ2, 6, and 8 (Figs. S11-S13), knowledge to reclaim land is an important factor to reduce fallow land (proxies: proportion of literate population, access to information such as magazine and newspapers). We find contrasting relationships between farm size (average size within each grid cell) and fallow land across sub-national hot spots. Cropland to fallow land conversion is positively associated with larger farm size in AEZ2 (Fig. S7), and positively with smaller farm size in semiarid hot spots (AEZ4, 5, and 8) (Figs. S8-S10). In AEZ2, resources are a limiting factor to fuller land utilization, as also indicated by negative relationship between fallow land and availability of labor, capital, and irrigation (Fig. S7). The massive reclamation of fallow land to cropland in AEZ2 during 1995–2005 (Fig. 2) is primarily from extension of tube well and well irrigation facilities (Figs. S13, S14). In semiarid hot spots, we find smaller farms are prone to soil erosion (Table S16), as small farms are uneconomical to mechanize (Yadav 1996; Reddy 2003; Singh 2013).

### Gross forest area loss

During 1985–1995, India lost ~3.1% (~23,800 km^2^ of gross forest loss, i.e., sum of all forest area loss) of the forest area that existed in 1985 (~764,100 km^2^), and the rate increased to ~3.5% during 1995–2005 (~25,780 km^2^ gross loss of ~745,100 km^2^ forest in 1995) (Fig. 1). Overall, India experienced a net forest loss (gross loss minus gross gain) of ~18,000 km^2^ consistently during both decades (see Text S2 for extended discussion). Cropland was the major source of forest conversion during both decades, contributing to ~39% of gross forest loss in 1985–1995, and ~35% during 1995–2005. The relative area of gross forest loss to shrubland increased from ~31% in 1985–1995 to ~32% in 1995–2005. Expansion of commercial plantations into forests accounted for ~7% of gross forest loss during both decades. These trends are in stark contrast with the 1988 National Forest Policy that regards forest as a national asset and imposed strict rules to protect them (Agrawal et al. 2011; Ravindranath et al. 2012).

A regional breakdown indicates that gross forest loss is widespread across India, and forest loss hot spots change over time (Fig. 2; Dataset S2). For example, in AEZ19 that enclose the Western Ghats (biodiversity hot spot), 6.8% of the regions forest area in 1985 was converted to other land use (gross forest loss of 3080 km^2^) by 1995 (35% each to shrubland and plantation, and 23% to cropland). In 1995–2005, the region’s gross forest area loss declined to 1630 km^2^. In AEZ5, 4.9% of the regions forest area in 1985 was converted to other land use by 1995, and the rate increased to 7.9% in subsequent decade. Nonetheless, Eastern Plateau and Eastern Ghats (AEZ12), Central Highlands (AEZ10), and Western Himalayas (AEZ14) emerged as persistent hot spots for both decades. AEZ5, 10, 12, and 17 collectively accounted for ~59% (1985–1995) and ~56% (1995–2005) of the national total of gross forest area lost to cropland. AEZ4, 5, 10, 12, and 19 collectively accounted for ~84% (1985–1995) and ~80% (1995–2005) of the national total of forest area converted to shrubland. AEZ12 alone accounted for 40% (1985–1995) and 35% (1995–2005) of the national total of gross forest area lost to shrubland.

**Fig. 4:**
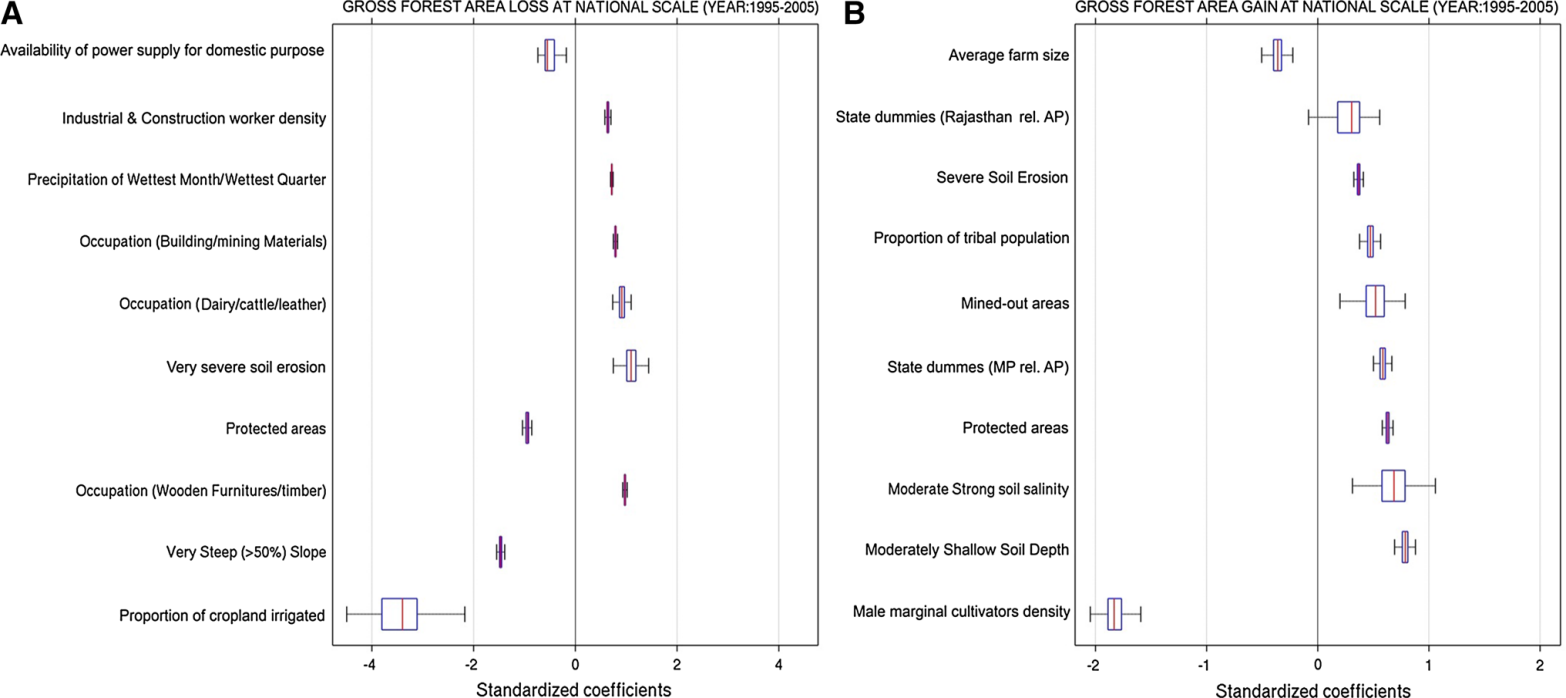
Similar to Fig. 3, but for: **a** forest area loss at national scale (1995–2005), and **b** forest area gain at national scale (1995–2005). See Figs. S15 and S22 for national-scale estimates corresponding to 1985–1995 (color figure online)

National-scale analysis of spatial determinants (Fig. 4a, S15) show strong negative association between proportion of cropland irrigated and gross forest area loss indicating that improvements in irrigation infrastructure can help to reduce the pressure on adjoining forests. We also find strong spatial association between forest area loss and village primary occupations (Fig. 4a, S15). Villages with following activities were prominently related to forest loss, compared to counterfactual buffer villages (in decreasing order of importance from Fig. 4a): wooden furnitures/timber products; cattle/dairy/leather products (due to overgrazing); mining/quarrying activities; and industrial development (proxy: industrial and construction worker density). Colder and wetter conditions and lack of electricity were also positively associated with forest loss (Fig. 4a, S15) suggesting over-extraction for fuel wood and construction materials.

We find prominent negative association of gross forest area loss with steep slope (difficult to access), and protected areas (Fig. 4a, S15). While land protection reduces forest loss, 9% (1985–1995) and 7.6% (1995–2005) of total gross forest loss have still occurred within protected areas, and 11.2% (1985–1995) and 8.7% (1995–2005) within 5 km buffer from the perimeter of protected areas (critical to maintain the functionality of protected landscapes) (Fig. S16), indicating level of protection is important and has improved over time.

Across AEZ hot spots, the following agriculture-related variables show prominent negative association with gross forest area loss: proportion of irrigated areas (Figs. S17-S19), higher fertility of agricultural soils (proxy: cation exchange capacity; Figs. S17a, S18-S20), average farm size (proxy for economic feasibility to mechanize; Figs. S17a, S18-S20), availability of power supply for agriculture (Figs. S17a, S20), proportion of main (=1-marginal) agricultural laborers (lower income dependence on forests; Fig. S17a), and proximity to agricultural credit institutions (proxy for access to capital; Fig. S20). These relationships broadly indicate that higher agricultural productivity tends to reduce the pressure on adjoining forests. Most diversion of forest to cropland is encroachment, because national forest policy does not favor diversion of forest to non-forest, which requires prior approval from central government (MoEF 1988; Joshi et al., 2011). Furthermore, we find the forest area diverted to cropland have not declined with time (Fig. 1), indicating weak implementation of national forest policy.

A regional analysis indicates that in AEZ19 that encloses the Western Ghats, mining activities, manufacturing of wooden agricultural implements, and villages dependent on coconut and coffee plantations (encroachment) show positive association with forest loss (Fig. S19). Across all hot spots in central India (AEZ5, 10, and 12), mining/quarrying activities, industrial development, and factors associated with low agricultural productivity (e.g., high erosion) show positive association with forest loss (Figs. S20, S17, S18). Other factors prominently associated with forest loss are wooden furniture/timber extraction and cattle overgrazing (AEZ5; Fig. S20); villages making bamboo products (AEZ12; Fig. S18); villages making forest products (e.g., tendu leaves/*beedi*, leaf plates, baskets, brooms, match sticks, paper pulp) (AEZ10; Fig. S17); colder temperatures (over-extraction of firewood and construction materials), wooden furniture/timber, and making of woolen blankets (indicating sheep over-browsing) (AEZ14; Fig. S21).

### Gross forest area gain

India recorded a positive trend in gross forest area gain over time (Fig. 1). The gross forest area gain in 1995–2005 was 24% higher than the preceding decade, compensating for the increased gross forest area loss during 1995–2005. Reversion of cropland and shrubland together explain 65% (1985–1995) and 78% (1995–2005) of gross forest area gain. AEZ5, 10, and 12 were persistent hot spots of gross forest area gain in both decades (Fig. 2; Dataset S2); however, the magnitude was much smaller compared to the gross forest area loss in the respective zones. During 1995–2005, substantial area of shrubland recovered to forest in AEZ4, 5 and 12 (Fig. 2).

Both nationally (Fig. 4b, S22) and across sub-national hot spots (Figs. S23-S26), we find prominent positive association between gross forest area gain and following agriculture-related variables (in decreasing order of importance based on Fig. 4b, S22): lower male marginal cultivators; higher levels of soil degradation (characterized by one or more of: shallow depth, salinization, and erosion); and smaller average farm size. These relationships indicate abandonment of marginally productive cropland, followed by either regrowth of forest tree species or conversion to forest plantations. We also find positive association between gross forest area gain and protected areas (Fig. 4b, S22-S26), proportion of tribal population (Fig. 4b, S22-S24), and area of sacred groves (Figs. S22, S24-S26). Tribes are culturally linked to forests, and they are typically motivated by state forest department to jointly manage forest through protection, restoration of degraded forest, and enrichment plantations (World Bank 2005; Government of India 2007; Macura et al. 2011) (notable exception of North-East India where tribes practice shifting cultivation). Sacred groves are typically protected by local community due to cultural/religious beliefs (Ormsby and Bhagwat 2010; Bhagwat et al. 2014).

Across the three sub-national hot spots (AEZ5, 10 and 12), gross forest area gains were positively associated with state administrative divisions, mined-out areas, density of forestry workers, and density of community workers (Figs. S23, S26, S24). The identified state administrative divisions typically have larger amount of forest inundated to water bodies (irrigation projects), and forest diverted to built-up land (e.g., roads, industries) (Fig. S27; Dataset S1). Both state administrative divisions and greening of mined-out areas indicate compensatory afforestation by respective state governments to partly compensate for forest loss. The forestry workers are employed by forest department and are a proxy for level of protection and control. These workers are typically involved in forest maintenance, wildlife protection, fire observations, and interface with tourism, among others. Community workers help with restoration efforts (e.g., greening firewood and fodder) by involving forest department and local communities.

### Comparison of modeled results with 102 ground studies

**Fig. 5:**
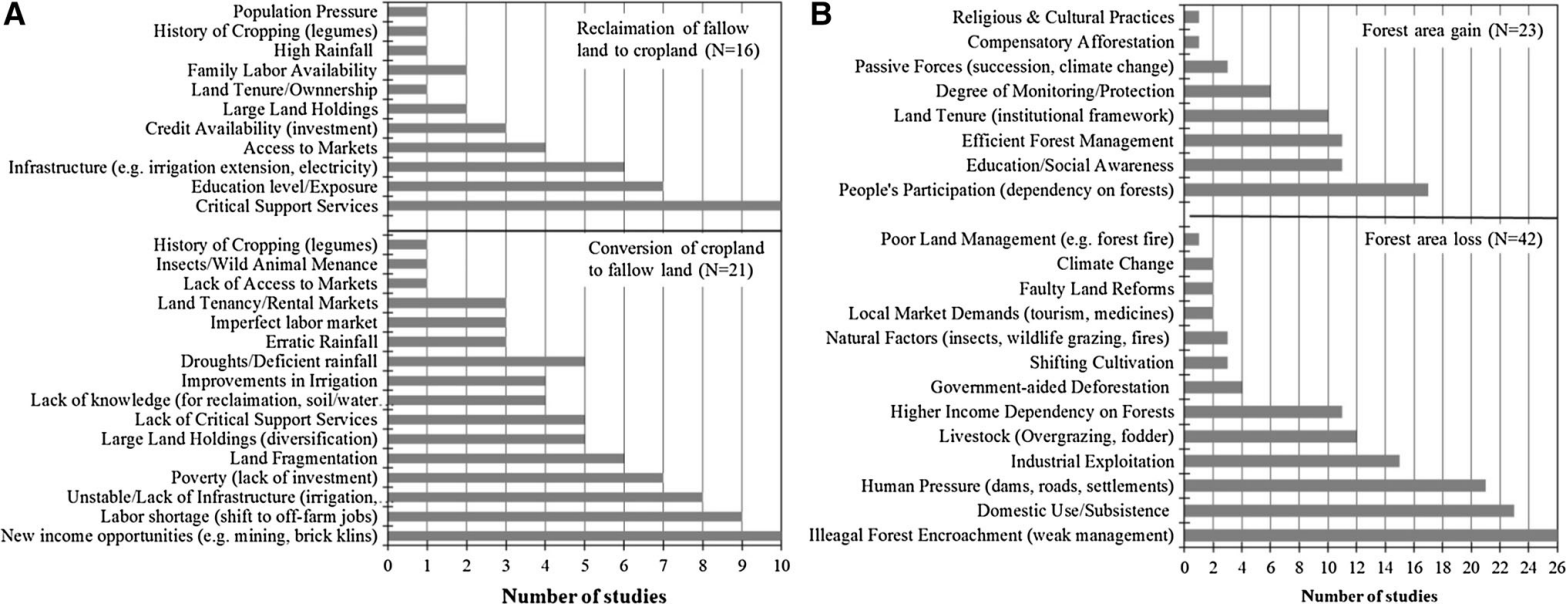
Frequency distribution of the causal factors identified from the synthesis of 102 case studies. **a** Conversions from cropland to fallow land and vice versa, and **b** forest area losses and gains

Our synthesis indicates that the three LULCC (cropland–fallow land conversions; forest area losses; and forest area gains) are driven by different combinations of factors. Nonetheless, the accumulated effects (Fig. 5; based on data summarized in Dataset S3) broadly concur with results of our regression analysis at national scale. Our synthesis indicates that fallow land is mainly associated with (based on 37 studies, i.e., *N* = 37) labor shortage/migration driven by new income opportunities (*N* = 14), lack of infrastructure (irrigation and electricity; *N* = 8), lack of access to capital (*N* = 7), and cropland fragmentation (smaller average farm size; *N* = 6). Reclamation of fallow land depends mainly on (based on 16 studies) critical support services (e.g., access to markets and capital; *N* = 10), level of education (knowledge to reclaim land; *N* = 7), and village infrastructure (mainly irrigation; *N* = 6). Illegal forest encroachment (for cropland expansion due to low productivity; *N* = 26), wood extraction for subsistence (*N* = 23), expansion of man-made structures (*N* = 21), industrial exploitation (*N* = 15), and cattle overgrazing (*N* = 12) are common causes of forest loss. Unlike cropland fragmentation that drives fallow land, no case studies (*N* = 42) suggested that forest fragmentation drives forest loss. Regarding forest area gains, only three case studies (D6, D7, and D10 in Table S11) were designed to consider passive forces (regrowth following land abandonment), with other studies focusing on factors that influence the effectiveness of participatory forest management programs (e.g., Joint Forest Management). Our study finds passive forces to be a major factor for forest area increase. The prominent socioeconomic factors of forest area gain identified from our regression analysis are echoed in our synthesis (involvement of local community, education/awareness, and effective forest protection).

Causal factors uncommon at national scale can be most important regionally. For example, both our study and the synthesis literature (Table S10) report wood extraction for construction materials as a main determinant of forest loss in AEZ14. Some factors can also behave differently in individual cases. For example, different case studies (Tables S8, S9) stemming from same AEZ show opposing effects on how education affects fallow land. Education (proxies: literate population, availability of educational facilities) causes a shift to off-farm jobs, thus increasing fallow land. In contrast, with education farmers perceive higher returns to investment on land, invest more on resource conservation, and have better access to information leading to fuller land utilization. Such heterogeneity is concurrent and important to recognize; in such cases, our statistical analysis covering the entire region helps identify the dominant effect.

## Discussion

Our analysis provides a comprehensive spatial coverage of the dynamics and spatial determinants of LULCC in India by integrating remote sensing data with rich and uniform socioeconomic data collected from each village and town at national scale. The analysis is important because a general understanding of the spatiotemporal dynamics and determinants of LULCC over larger regions of India is limited, hindering effective national-level planning and policy making.

Our analysis of spatial determinants is useful because it adds a quantitative component to our study. Determinants help identify biophysical and socioeconomic variables that contribute to the statistical explanation of (the location of) observed LULCC. However, determinants do not necessarily imply causality; they only provide some empirical support for causal relations. On the other hand, local case studies often identify causality. Our synthesis of case studies (Tables S8-S11) helps to identify causes that are common across case studies (Fig. 5). The synthesis is useful because it provides a more generalized understanding of the causes of LULCC in India. However, the study design varied widely across the 102 cases we examined. Therefore, we relied on frequency analysis to identify common causes across case studies, as opposed to a more formal quantitative assessment. Nonetheless, the generalized understanding from our synthesis reinforces the findings of our spatial determinants and can inform national-level policies and governance options.

### Caveats

Three caveats are in order. *First*, as we estimated LULCC from decadal satellite images, they capture only the decadal changes in LULCC, and can mask within-decade variations including intermediary land uses. Especially, inter-annual climate variability causes fluctuations in fallow land (Dataset S4). However, the conversions between cropland and fallow inferred between decadal end points reflect only the climate effect of end point. Our decadal data also cannot identify land fallowed as a part of multiple cropping systems to restore and maintain soil fertility. Except cropland–fallow systems, other land cover conversions (e.g., forest to cropland) tend to be unidirectional at decadal timescale due to high cost of land reversion (Gibbs et al. 2010; Pandey and Seto 2015).

*Second*, both forest degradation and regrowth are gradual and cause subtle modifications to land cover. However, our Landsat analysis detects changes only when the magnitude of modification is large enough to cause shift from one land cover category to another (e.g., forest to shrubland for forest degradation). The resulting bias is likely minimal because: (1) persistent modification of forest would likely manifest as a change in land cover within a decade, and (2) our statistical estimation weighs each observation (grid cell) by the magnitude of land change; thus, small changes have less influence in our model.

*Third*, our analysis does not extend beyond 2005 due to data limitations. Wall-to-wall analysis of Landsat scenes is laborious, and efforts are underway to extend our decadal land cover conversion estimates to 2015. Furthermore, while India has conducted the 2011 socioeconomic census, tabular data on village profiles is on hold, pending consistency and quality checks. Nonetheless, our analysis already covers two decades and offers key insights on the non-stationary of factors associated with LULCC in India.

### Implications for land use planning

Our results highlight the dichotomy where on the one hand, large amounts of India’s cropland area are converted fallow, thereby not contributing to agricultural production. On the other, forest area is being encroached for agriculture. We show that both land conversions occur in areas of low agricultural productivity as broadly indicated by factors related to deficits in infrastructure (irrigation and markets), knowledge and critical support services. Our results imply that strategies to improve agricultural productivity can have a positive effect by enhancing food production and simultaneously help reduce the pressure on forest (our analysis, however, excludes indirect impacts that may offset the effectiveness). This is crucial for sustainable land use planning in India because India is among the world’s fastest growing economy and population, with constant land area. Henceforth, we discuss specific implications of our results for land use planning in India.

Our results indicate that labor shortage; land fragmentation; and deficits in infrastructure, knowledge, and access to capital are key factors associated with crop to fallow conversions. There are threefold implications of our results. First, with the National Rural Employment Guarantee Scheme (NREGS; Ministry of Rural Development 2005), rural wages have increased through alternative job opportunities in rural areas and new job opportunities in the fast-growing urban centers (note that NREGS was introduced in 2005 which is beyond our study period; however, watershed development programs (Gray and Srinidhi 2013) were a precursor to NREGS). With higher wages, the incentive to produce agricultural crops reduces, thereby pulling people to off-farm jobs (Mitra and Murayama 2009; Srivastava 2011), causing more fallow land. This implies that despite labor shortage, keeping the prices of food and agricultural produce cheap would require encouraging mechanization and better market access to farmers to protect their rights (reduce middlemen exploitation). Cheaper food is important in the short-run because one-third of India’s population lives below the poverty line (Gulati et al. 2012). Furthermore, our analysis indicates that livestock overgrazing is a key factor associated with forest loss. Protecting existing forest from overgrazing would require confined feeding which implies higher cost for farmers (except for milch animals in certain areas). Therefore, encouraging mechanization would not only help improve agricultural viability, but also help reduce the pressure on forests.

Second, small farms have low technical efficiency and have increased risk of soil degradation (see Table S16 for AEZ-wise correlation statistics). Importantly, our reported process of fallowing small, less productive farms combined with job opportunities from an industrializing economy show striking similarity to the path outlined in forest transition theory (Rudel et al. 2005; Mather 2007; Meyfroidt and Lambin 2011). The problem of cropland fragmentation is likely compounded in the future with increasing population and further subdivision of households. Effective strategies to prevent further land fragmentation and consolidation of farmers fragmented land holdings can help to improve the economic viability of agriculture in some cases (Jha et al. 2005; Niroula and Thapa 2005; Kumar et al. 2015).

Third, our results underscore the critical need to extension and better management of irrigation infrastructure and other common-pool resources to help reduce fallow land. Improving irrigation infrastructure requires both efficient management of surface irrigation and equitable use of ground water resources. Our analysis suggests that wastelands have already been consistently reclaimed to cropland (primarily AEZ2, 5, and 8), with support from both public and private initiatives, e.g., through building Indira Gandhi Canal in AEZ2 and Integrated Wasteland Development Programs (Rao and Pant 2001; Saxena 2006; Ghosh 2010; Maji et al. 2010). Concurrently, farmers have fallowed much larger areas of existing cropland, representing an undesired trade-off of wasteland reclamation. Numerable social surveys have shown that Indian farmers invest more on protecting fertile cropland (Maikhuri et al. 1997; Shiferaw et al. 2006; Kuppannan and Devarajulu 2009; Wani et al. 2011; Nüsser et al. 2012) than restoring degraded soils. Therefore, better orientation of investment portfolios with farmer’s attitude can help reduce fallow land.

Finally, our results show prominent positive association between forest loss and the economic dependence of village communities on forests across many regions. Currently, ~173,000 villages in India depend on forest for subsistence due to lack of alternative economic opportunities (Nayak et al. 2012). The ongoing and future planned privatization of afforestation programs in India tends to maximize corporate profits, with little space for community involvement (Planning Commission 1998; Bramhane et al. 2000; Saxena 2015). Our analysis underscores the critical need for forest policies to widely adopt a bottom-up approach by involving local communities and village councils to effectively implement afforestation programs, e.g., by creating minor forest resources outside of forest area that benefit the local community. There already exist best practices on forest management tested at community level in India (Lise 2000; Prasad and Kant 2003; Nagendra 2009; Bhattacharya et al. 2010; Dilip Kumar 2015). However, forest protection would benefit if these models are upscaled, ingrained as policy, and integrated with implementation system through capacity building and technology upgrades.

## Data access

Our satellite LULC data for three decades can be downloaded for free from http://dx.doi.org/10.3334/ORNLDAAC/1336. We are sharing the data at 100 m spatial resolution to conform to the map dissemination guidelines imposed by India’s 2005 National Map Policy (Survey of India). Our geospatial village-level socioeconomic database (covering 1991 and 2001) will be made available for download for free from NASA Socioeconomic Data and Applications Center (SEDAC; http://sedac.ciesin.columbia.edu/). Contact the first author for more information.

## Electronic supplementary material

Below is the link to the electronic supplementary material.

Supplementary material 1 (XLS 458 kb)

Supplementary material 2 (XLS 541 kb)

Supplementary material 3 (XLS 49 kb)

Supplementary material 4 (XLS 79 kb)

Supplementary material 5 (DOCX 7671 kb)

Supplementary material 6 (DOCX 615 kb)

Supplementary material 7 (PDF 852 kb)
