# Supplementary material for: Dynamics and determinants of land change in India: integrating satellite data with village socioeconomics
Source: Reg Environ Change. 2016 Oct 27;17(3):753–66. doi: 10.1007/s10113-016-1068-2 (PMC7064035; doi:10.1007/s10113-016-1068-2)

Figures

Figure S1. Visualization of the seamless village-level boundaries of India prepared for this study. We manually digitized the village boundaries from publicly available village boundary maps (hard copy maps obtained from respective district headquarters) of each state of India. We first complied the digitized village maps to state level, and then combined from state to national level. The national boundaries are from Openstreet map (opendatacommons.org) distributed under the Open Database License (http://www.openstreetmap.org/copyright).

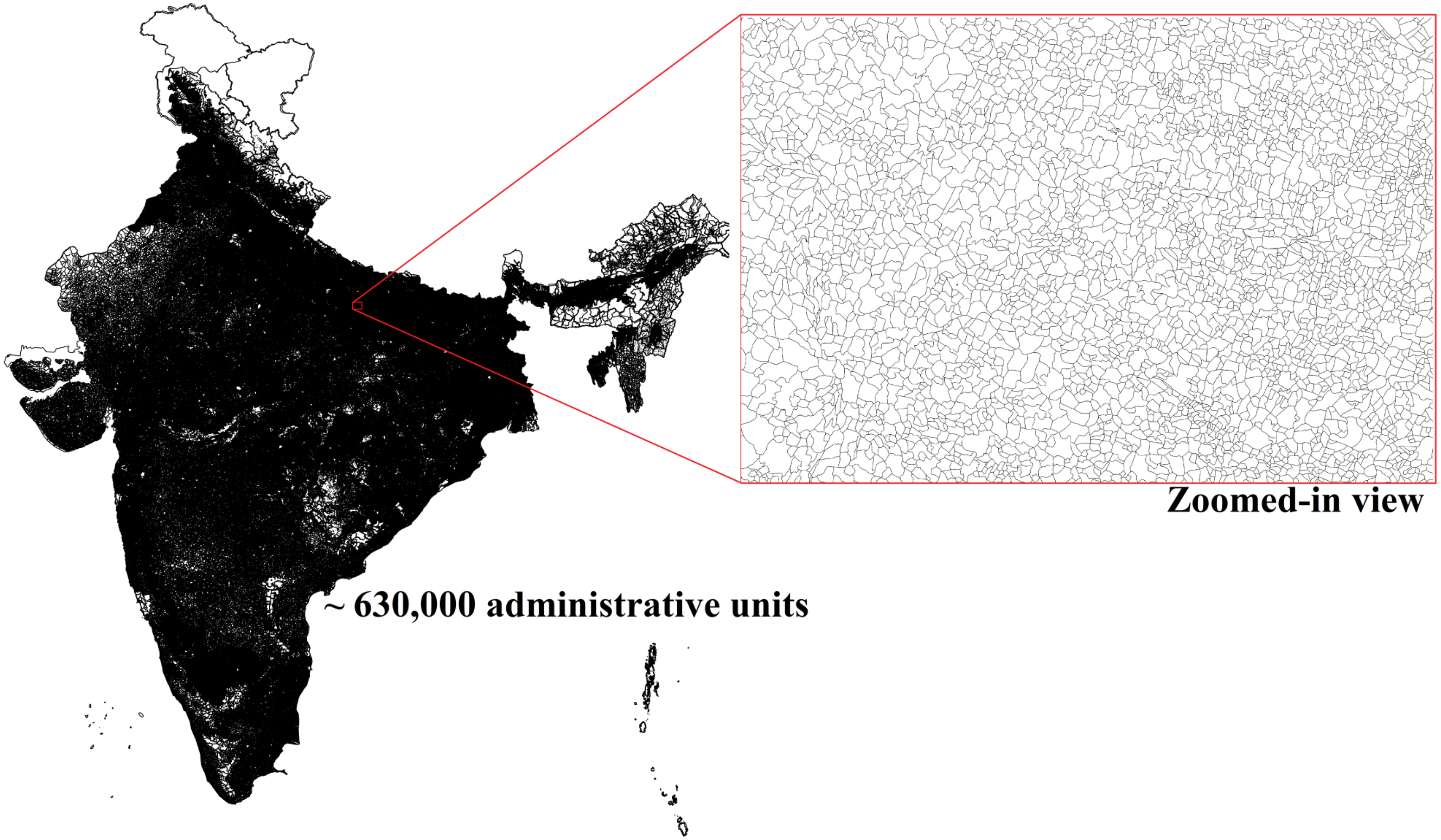

Figure. S2. Four examples illustrating the granularity of census data (2001). The adjacent panels compare the same data at two different spatial levels of disaggregation: village/town (used in our study), and taluka level (the subsequent administrative hierarchy). Four examples shown: (a) Total population data, (b) Total agricultural laborers density, (c) Mining/Quarrying worker density, and (d) Irrigation by tube well with electricity.

Sub-plot (a)

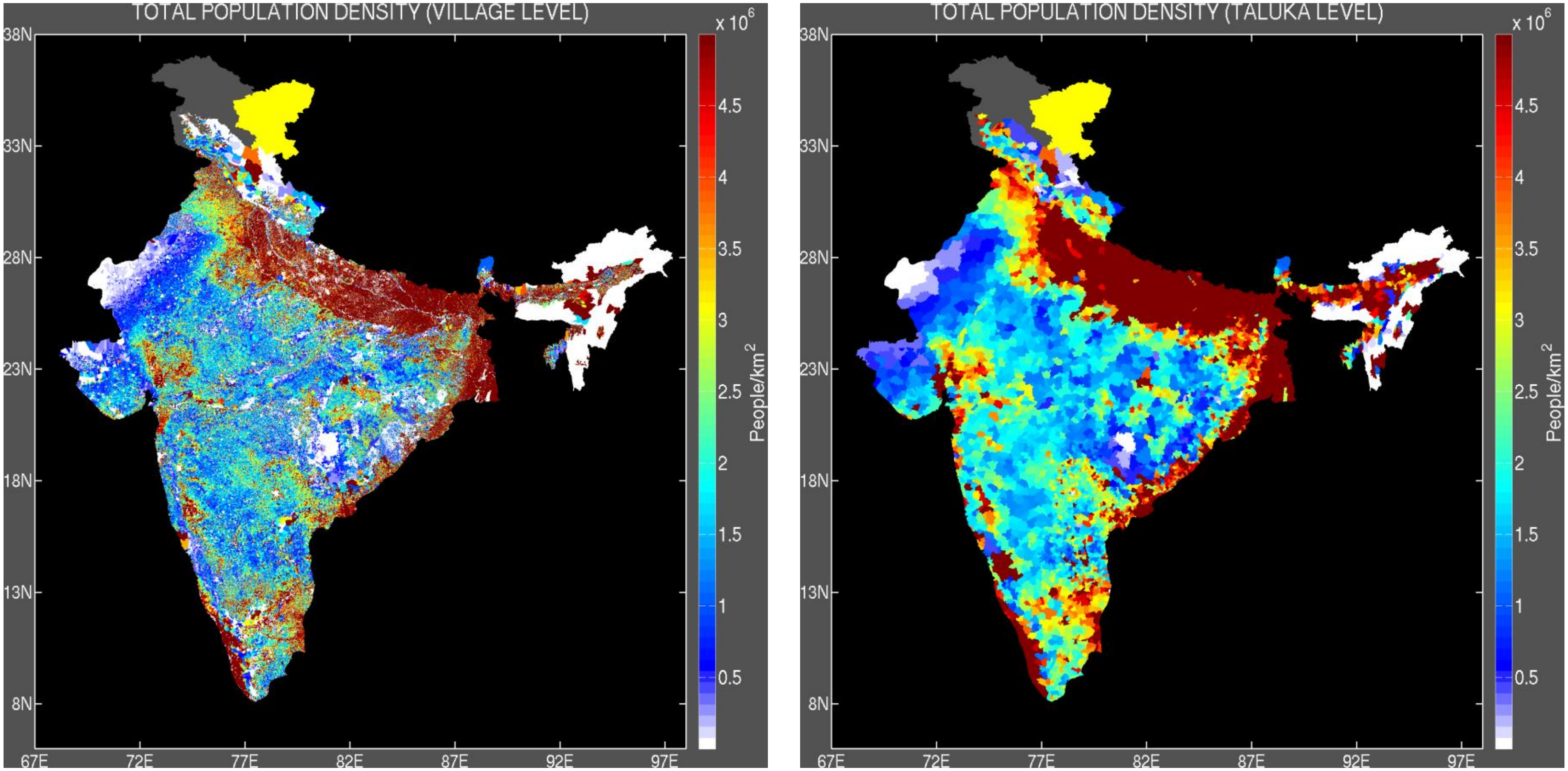

Sub-plot (b)

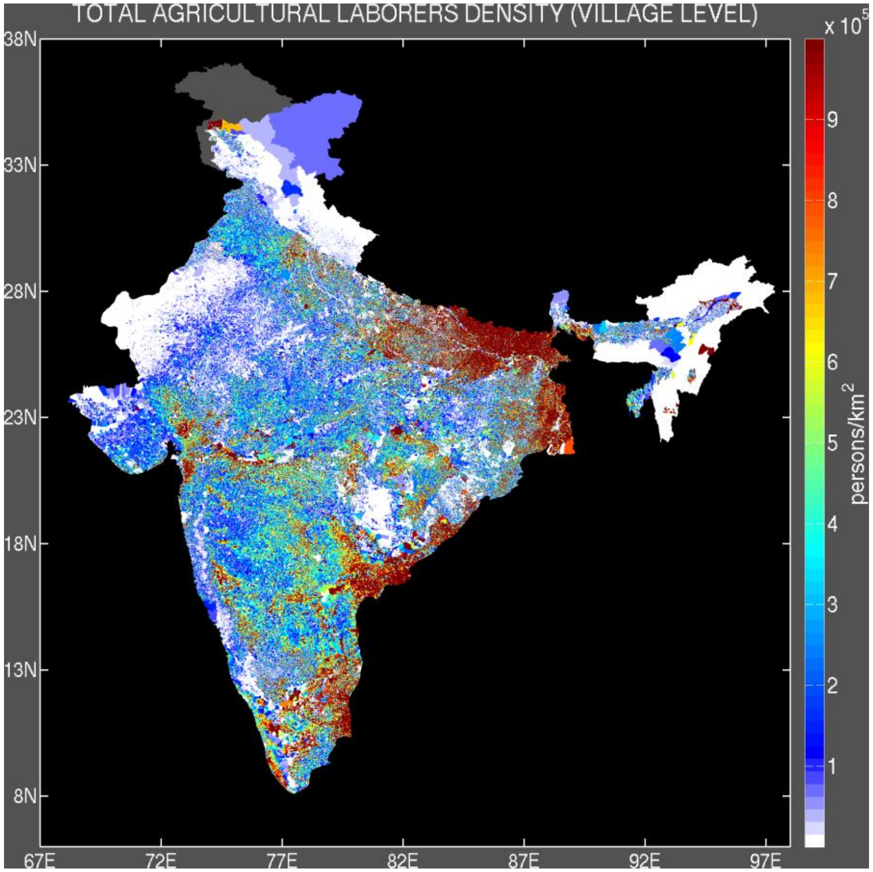
TOTAL AGRICULTURAL LABORERS DENSITY (VILLAGE LEVEL)
x 10^5
persons/km^2
38N
33N
28N
23N
18N
13N
8N
67E
72E
77E
82E
87E
92E
97E
1
2
3
4
5
6
7
8
9

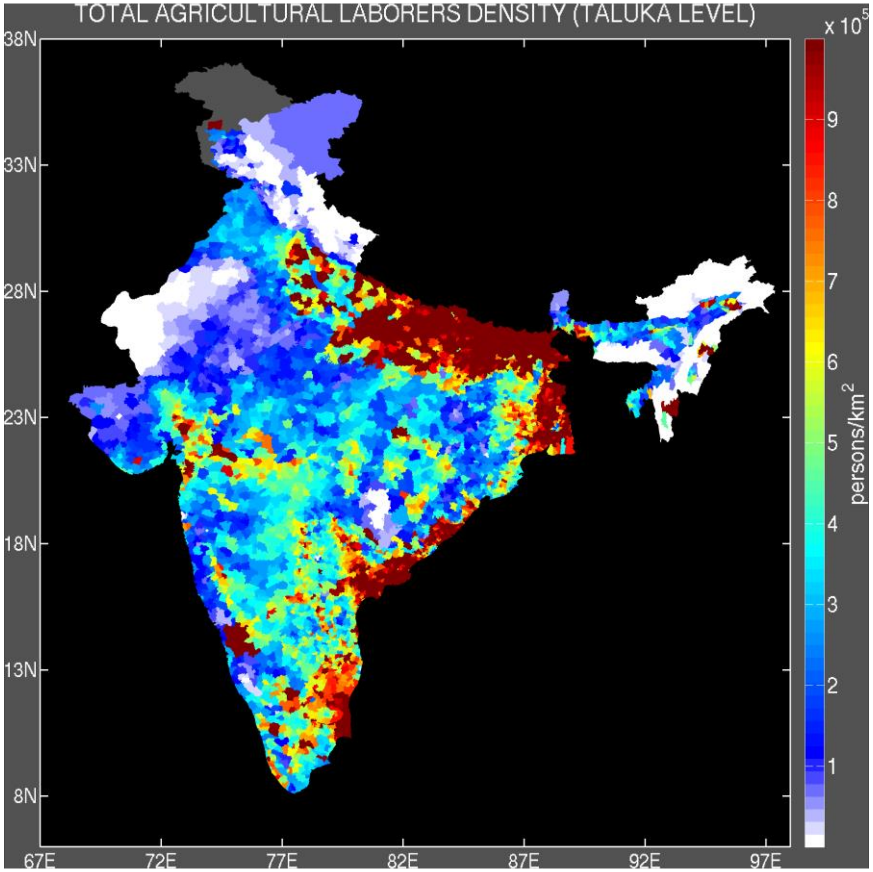
TOTAL AGRICULTURAL LABORERS DENSITY (TALUKA LEVEL)
x 10^5
persons/km^2
38N
33N
28N
23N
18N
13N
8N
67E
72E
77E
82E
87E
92E
97E
1
2
3
4
5
6
7
8
9

Sub-plot (c)

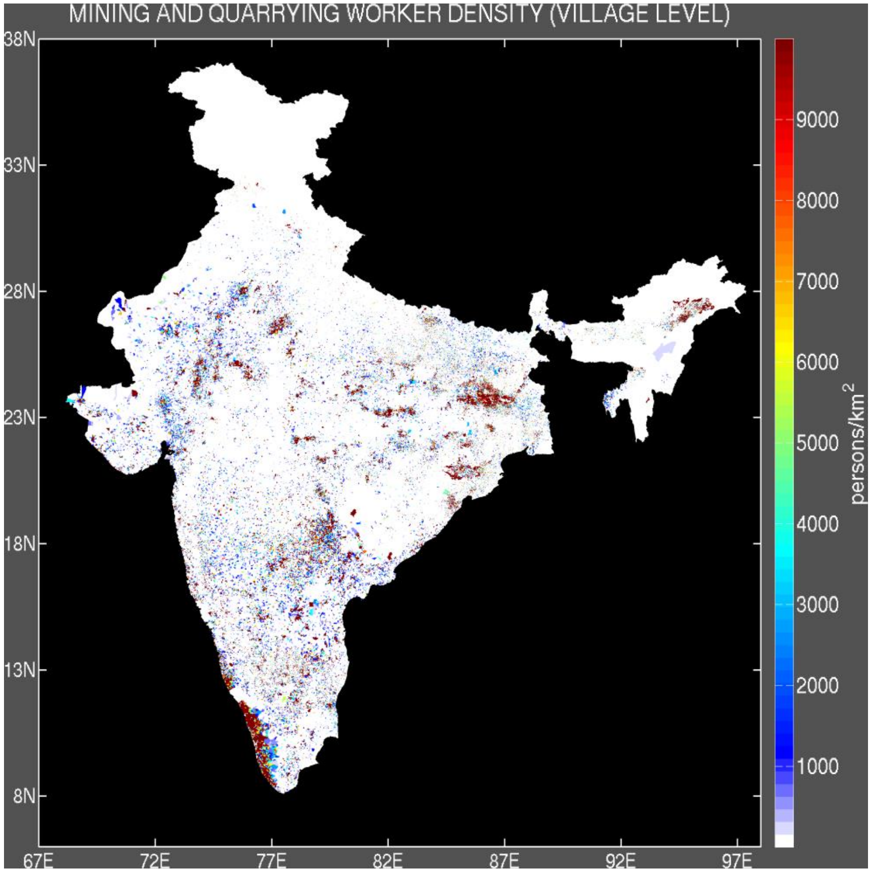
MINING AND QUARRYING WORKER DENSITY (VILLAGE LEVEL)
38N
33N
28N
23N
18N
13N
8N
67E
72E
77E
82E
87E
92E
97E
9000
8000
7000
6000
5000
4000
3000
2000
1000
persons/km2

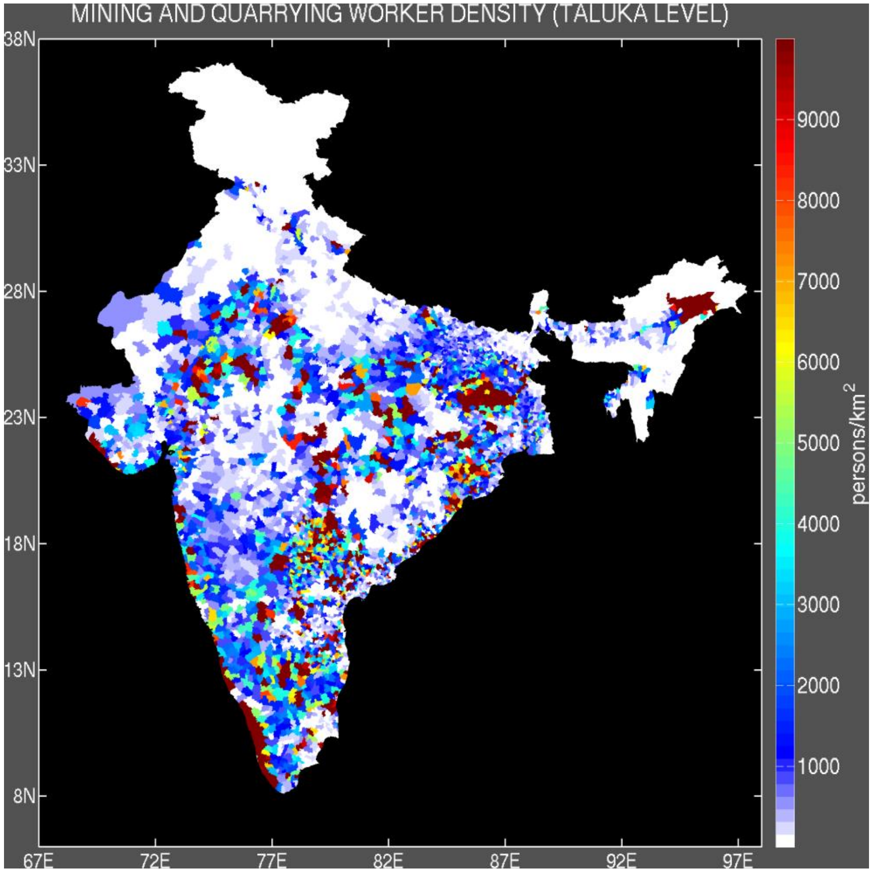
MINING AND QUARRYING WORKER DENSITY (TALUKA LEVEL)
38N
33N
28N
23N
18N
13N
8N
67E
72E
77E
82E
87E
92E
97E
9000
8000
7000
6000
5000
4000
3000
2000
1000
persons/km2

Sub-plot (d)

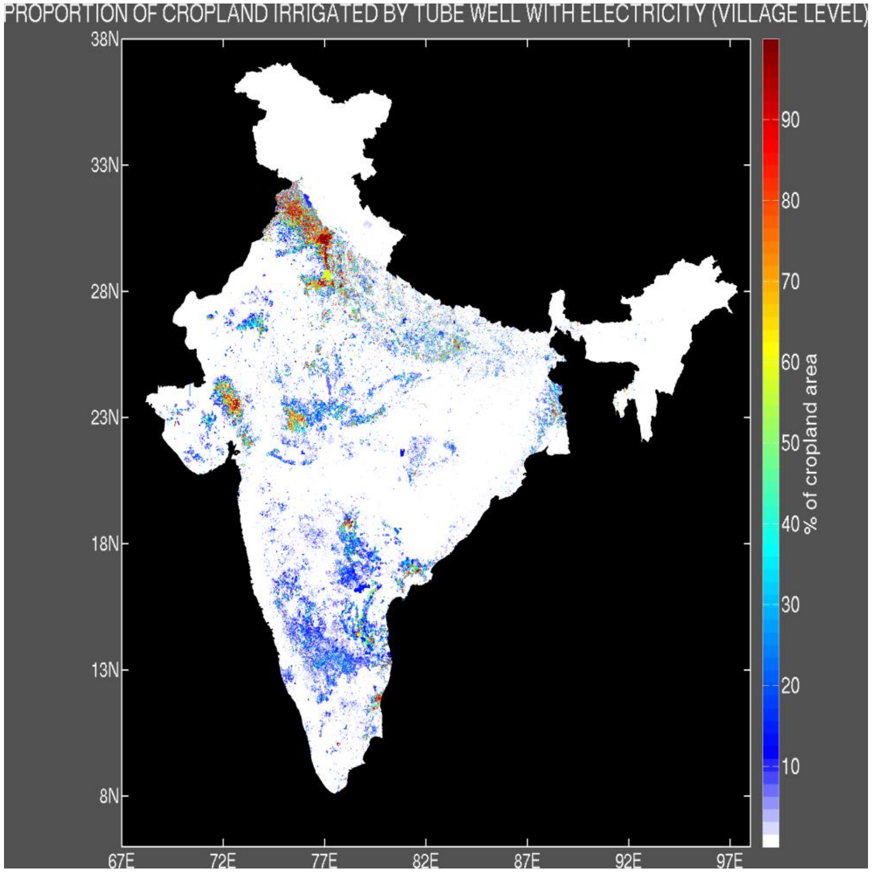
PROPORTION OF CROPLAND IRRIGATED BY TUBE WELL WITH ELECTRICITY (VILLAGE LEVEL)
38N
33N
28N
23N
18N
13N
8N
67E
72E
77E
82E
87E
92E
97E
90
80
70
60
50
40
30
20
10
% of cropland area

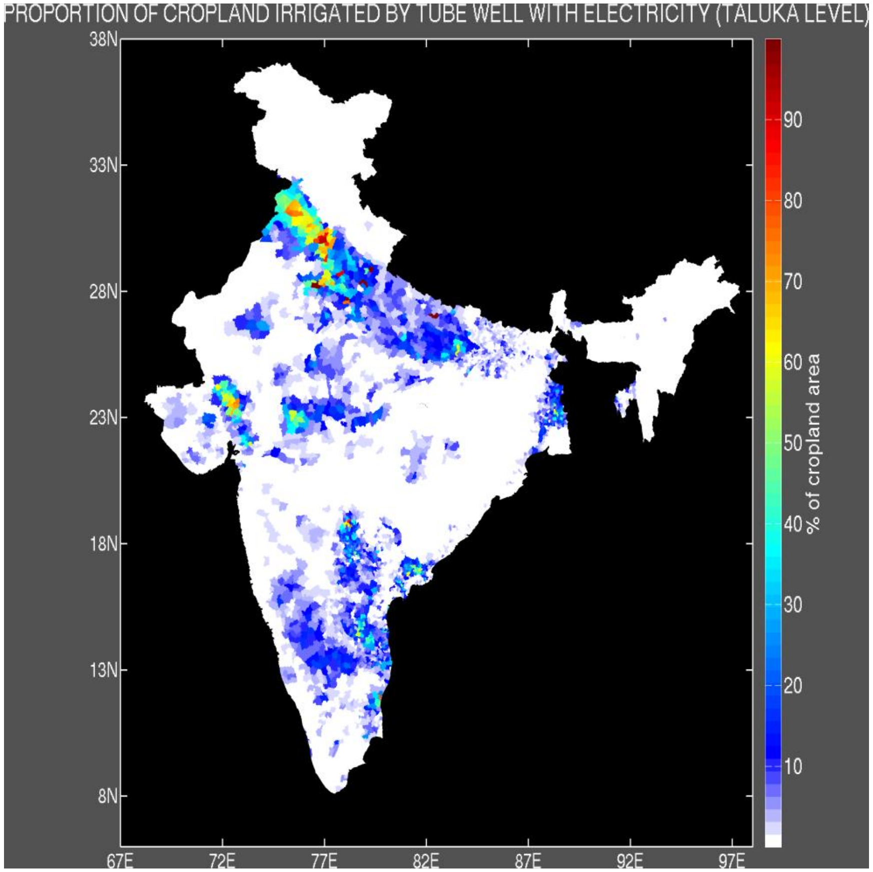
PROPORTION OF CROPLAND IRRIGATED BY TUBE WELL WITH ELECTRICITY (TALUKA LEVEL)
38N
33N
28N
23N
18N
13N
8N
67E
72E
77E
82E
87E
92E
97E
90
80
70
60
50
40
30
20
10
% of cropland area

Figure S3. Location of the 102 studies included in our synthesis.

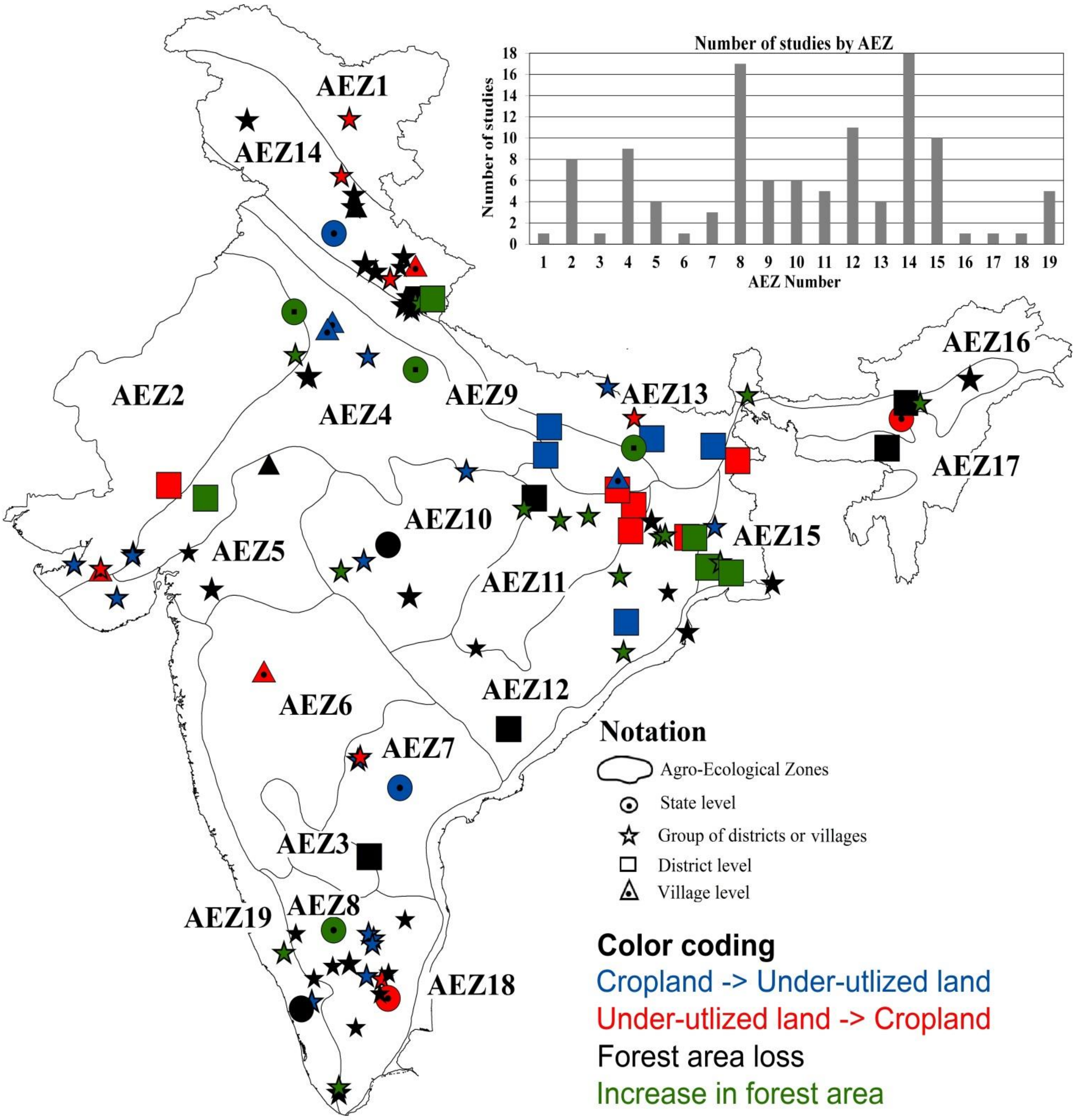

Figure S4. Extension to Fig. 2 showing the regional breakdown of key land-cover conversions. The bar plots show the percent contribution by Agro-Ecological Zones (AEZ) to the national total (national total shown besides bar; units in x1000 km^2/decade and rounded to nearest integer). See Table S6 for definition of AEZs.

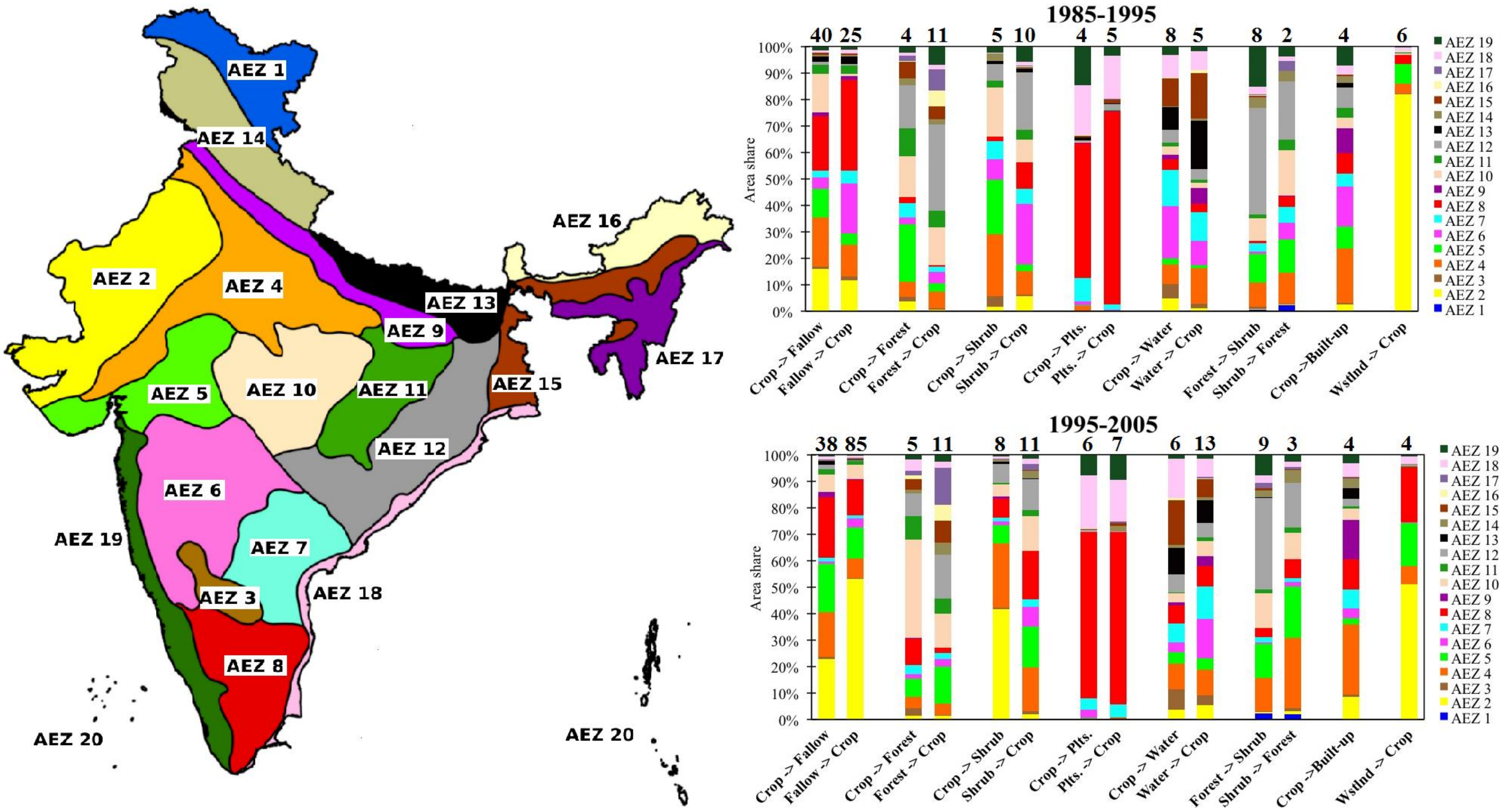

Figure. S5. Similar to Fig. 3 but for 1985-1995. Factors most prominent in explaining: (a) conversion of cropland to fallow land at national scale (1985-1995), and (b) vice-versa conversion i.e. conversion of fallow land to cropland at national scale (1985-1995).

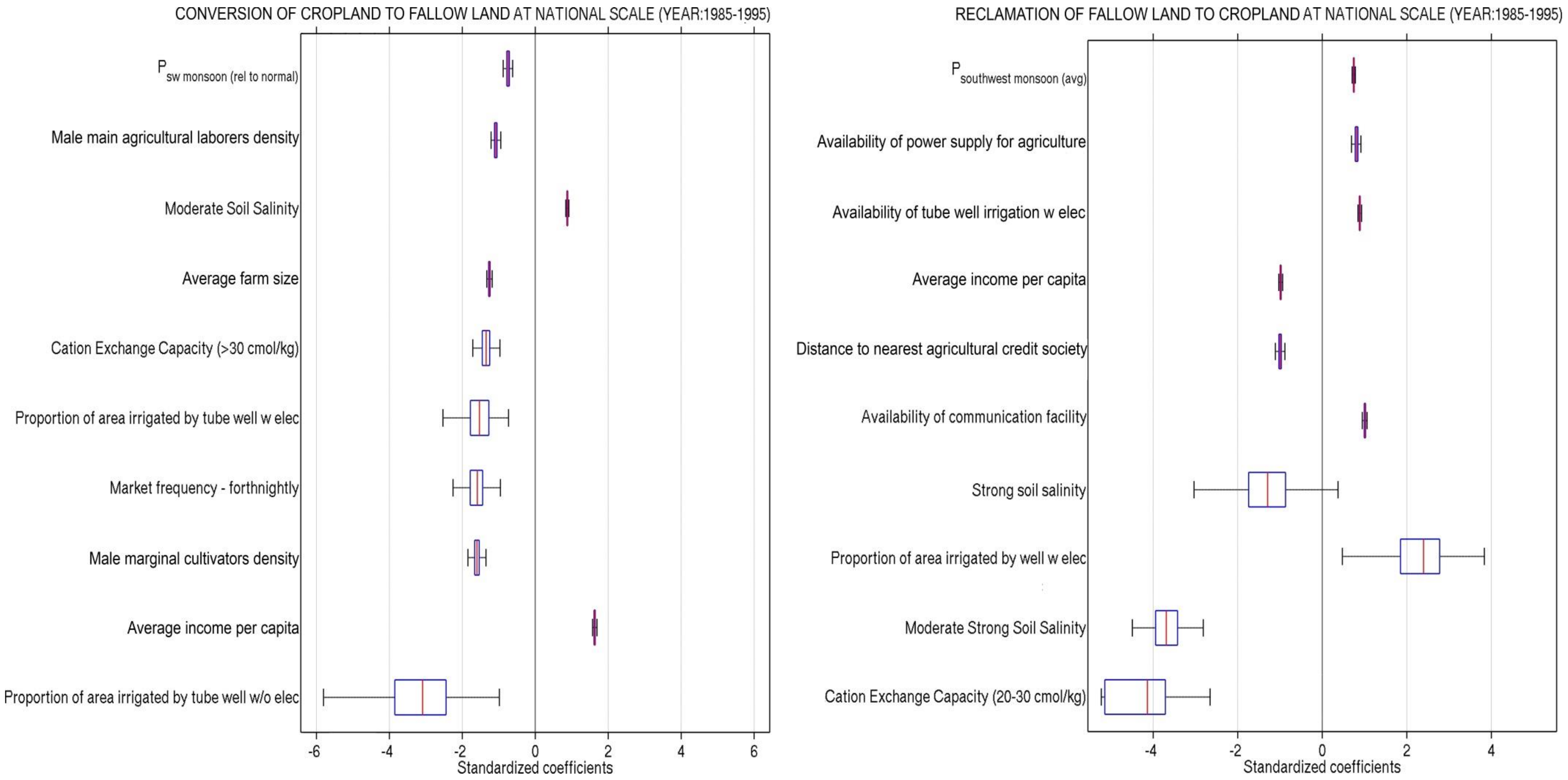

Figure. S6. The series of 14 figures show changes (between 1991 and 2001) in spatial patterns of farm labor calculated from village level census database (~630,000 political units). The detailed breakdown is shown for broad interpretation of results, and also because this is the first time Indian farm labor force is being visualized at this level of spatial and demographic detail. The data is broken down by three broad components: (1) by gender, (2) agricultural laborers and cultivators, and (3) main and marginal laborers. Agricultural laborers are people who worked in another person's land for wages in cash, kind or share. Such a person had no risk in cultivation but merely worked in another person's land for wages. An agricultural laborer had no right of lease or contract on land on which he worked. Cultivators are people who was engaged either as employer, single worker or family worker in cultivation of land owned or held from government of held from private persons or institutions for payment in money, kind or share of crop. Cultivation included supervision or direction of cultivation. Main workers were those who had worked for the major part of the year preceding the date of enumeration i.e., those who were engaged in any economically productive activity for 183 days (or six months) or more during the year. Marginal workers were those who worked any time at all in the year preceding the enumeration but did not work for a major part of the year, i.e., those who worked for less than 183 days (or six months).

Sub-plot captions are as follows: (a) Total agricultural laborers density, (b) Total cultivators density, (c) Main agricultural laborers density (male + female), (d) Marginal agricultural laborers density (male + female), (e) Male main agricultural laborers density, (f) Female main agricultural laborers density, (g) Male marginal agricultural laborers density, (h) Female marginal agricultural laborers density, (i) Main cultivators density, (j) Marginal cultivators density, (k) Male main cultivators density, (l) Female main cultivators density, (m) Male marginal cultivators density, and (n) Female marginal cultivators density. Positive values indicate an increase in population from 1991 to 2001, and negative values indicate the vice-versa.

Fig. S6 (Continued). Sub-plots (a) and (b)

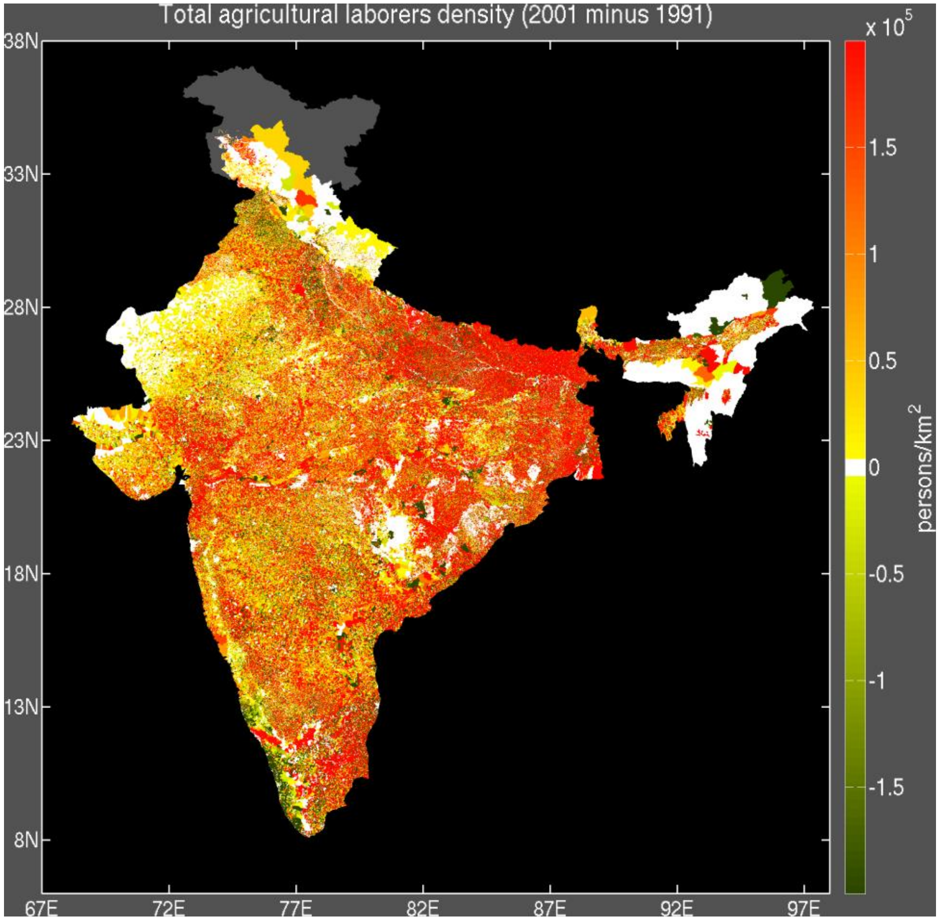

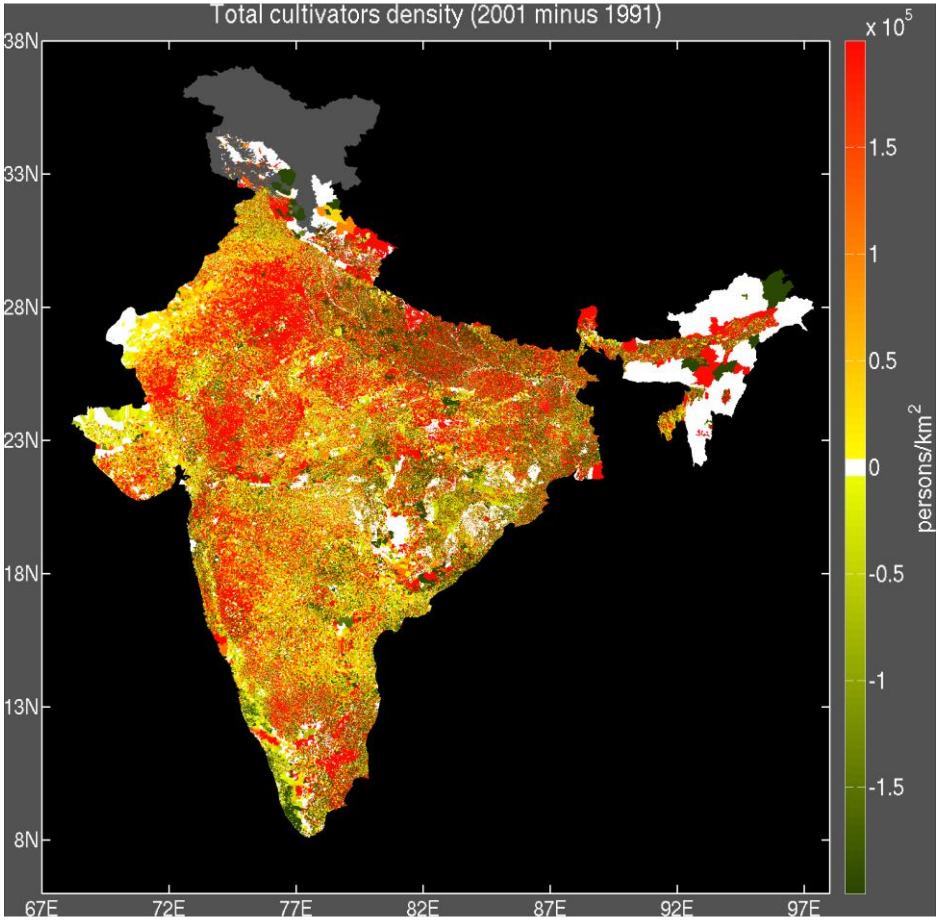

Fig. S6 (Continued). Sub-plots (c) and (d)

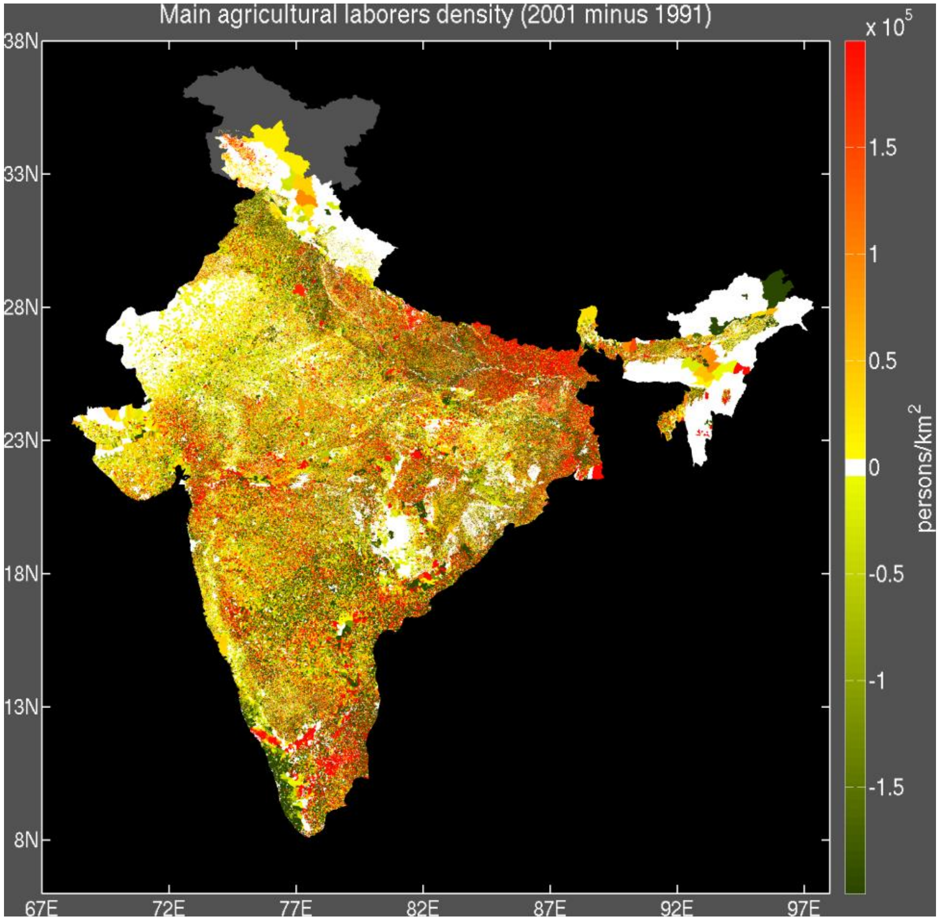

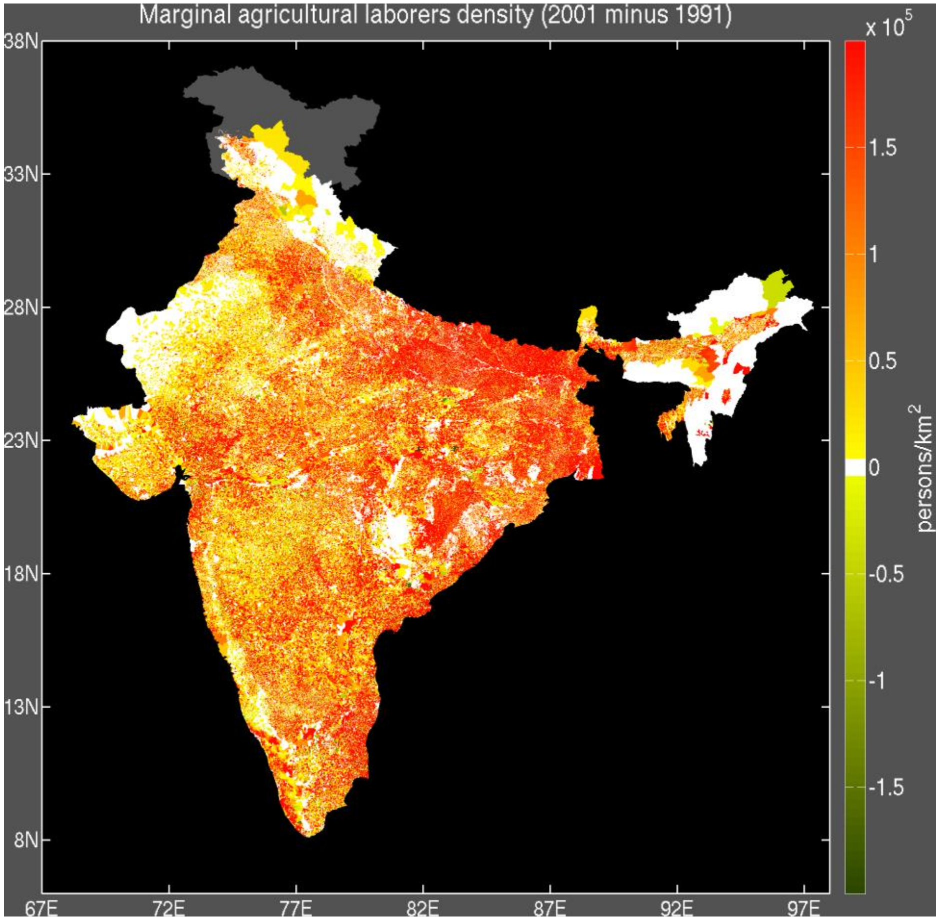

Fig. S6 (Continued). Sub-plots (e) and (f)

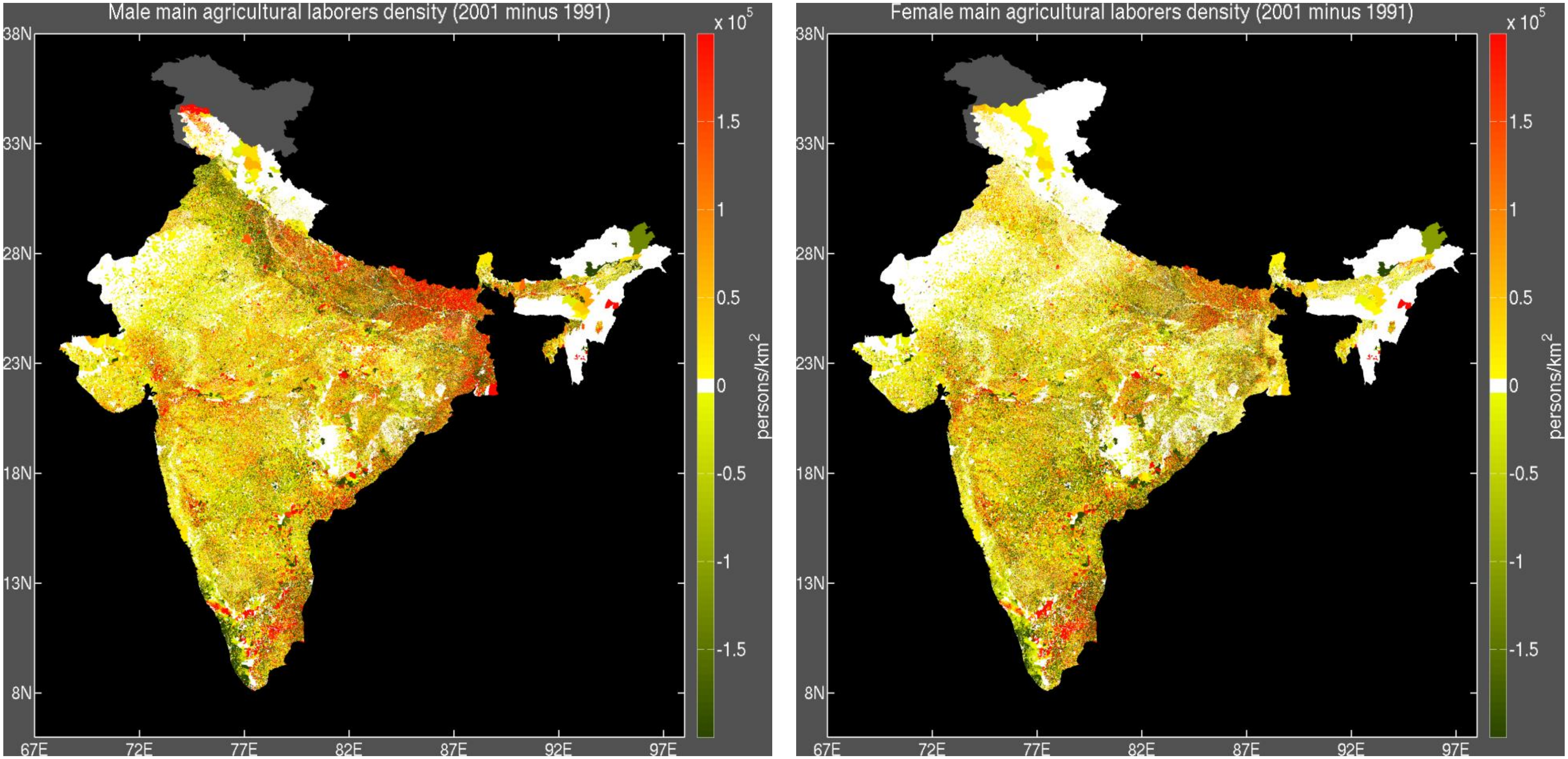

Fig. S6 (Continued). Sub-plots (g) and (h)

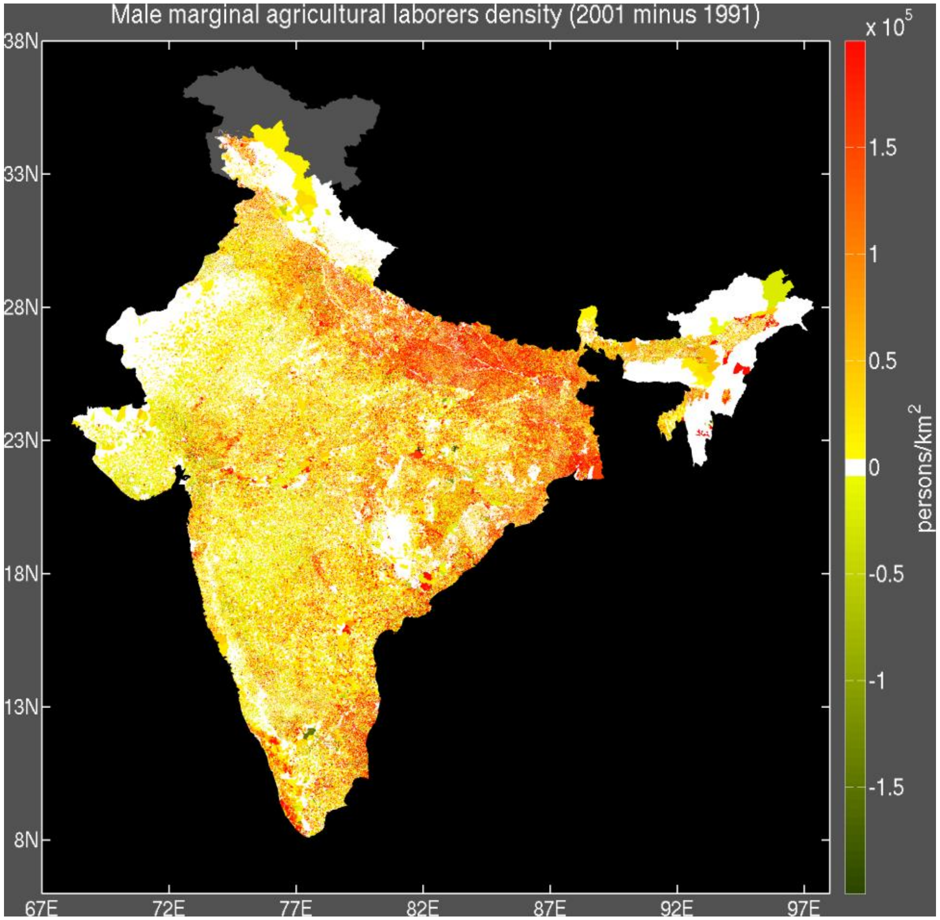

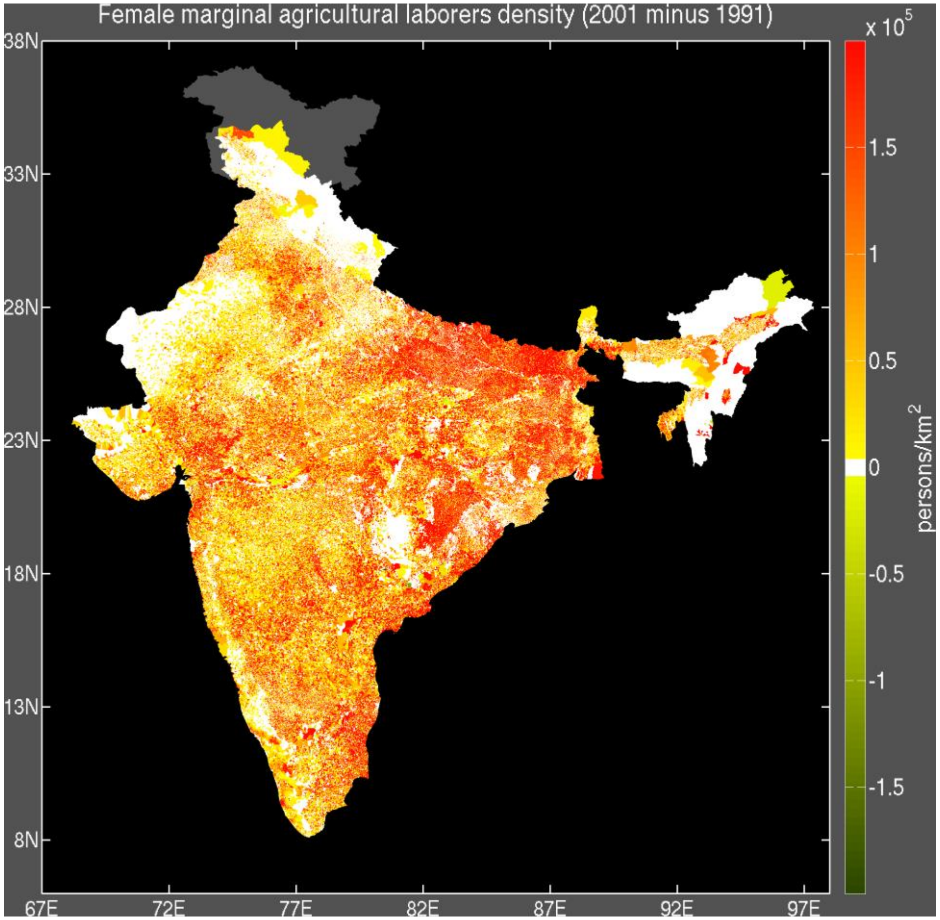

Fig. S6 (Continued). Sub-plots (i) and (j)

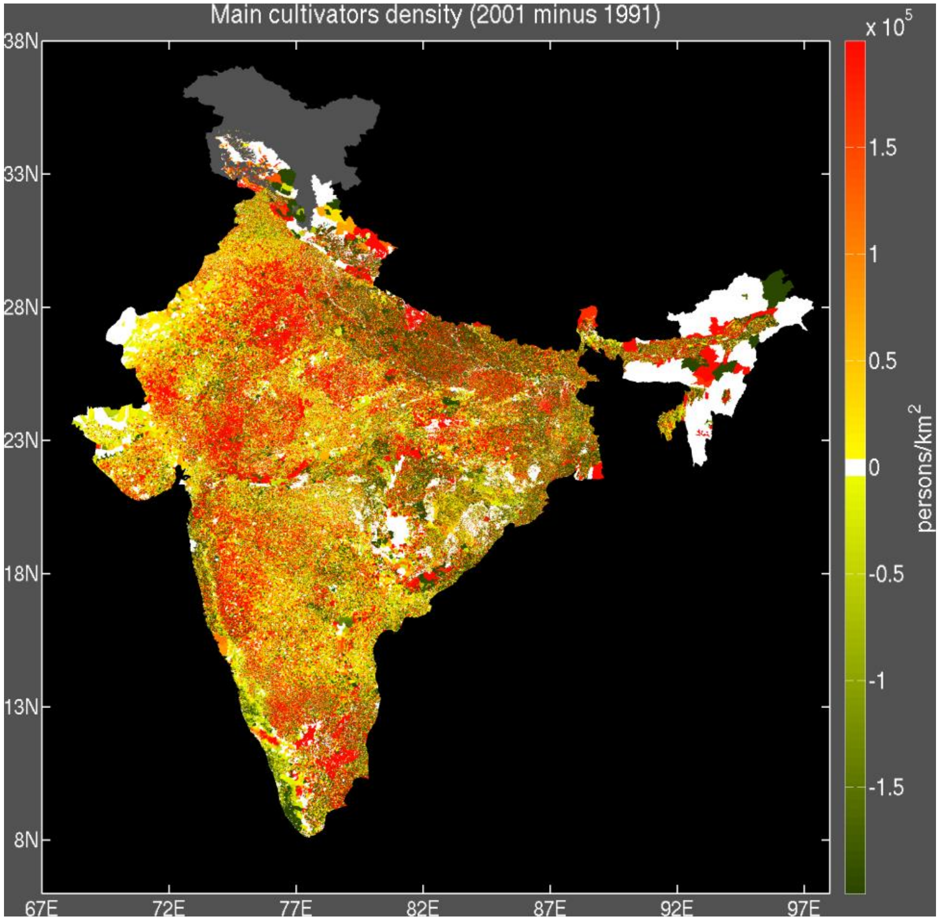

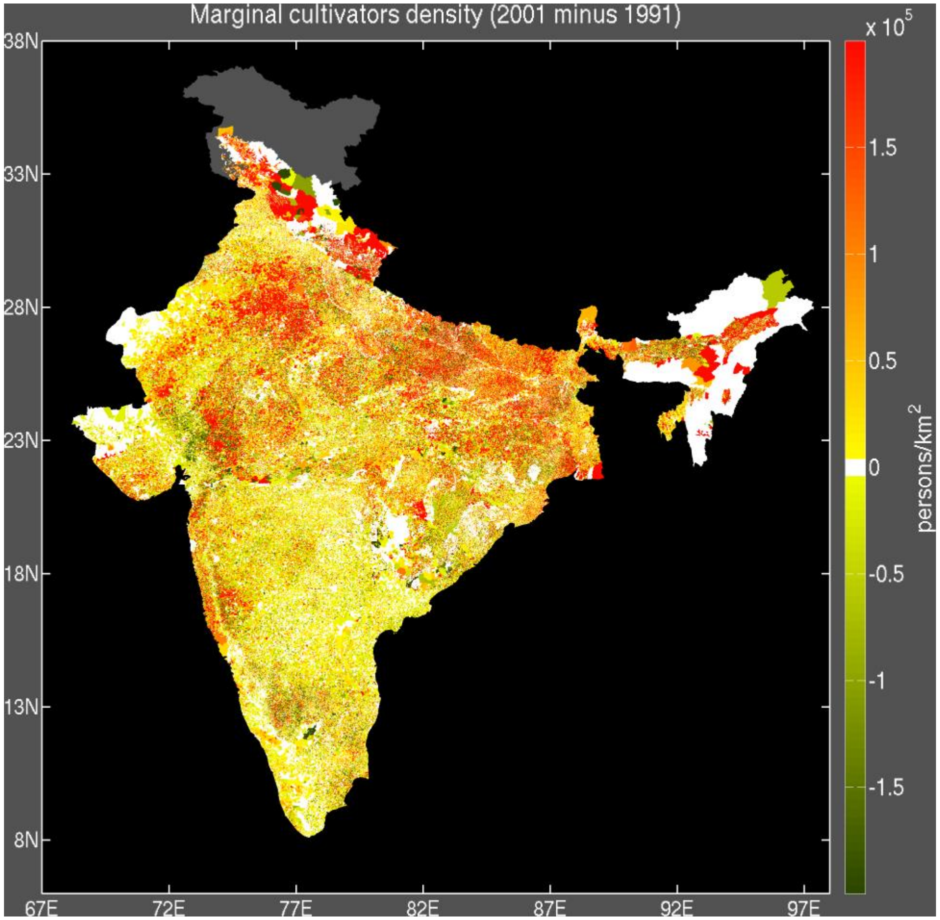

Fig. S6 (Continued). Sub-plots (k) and (l)

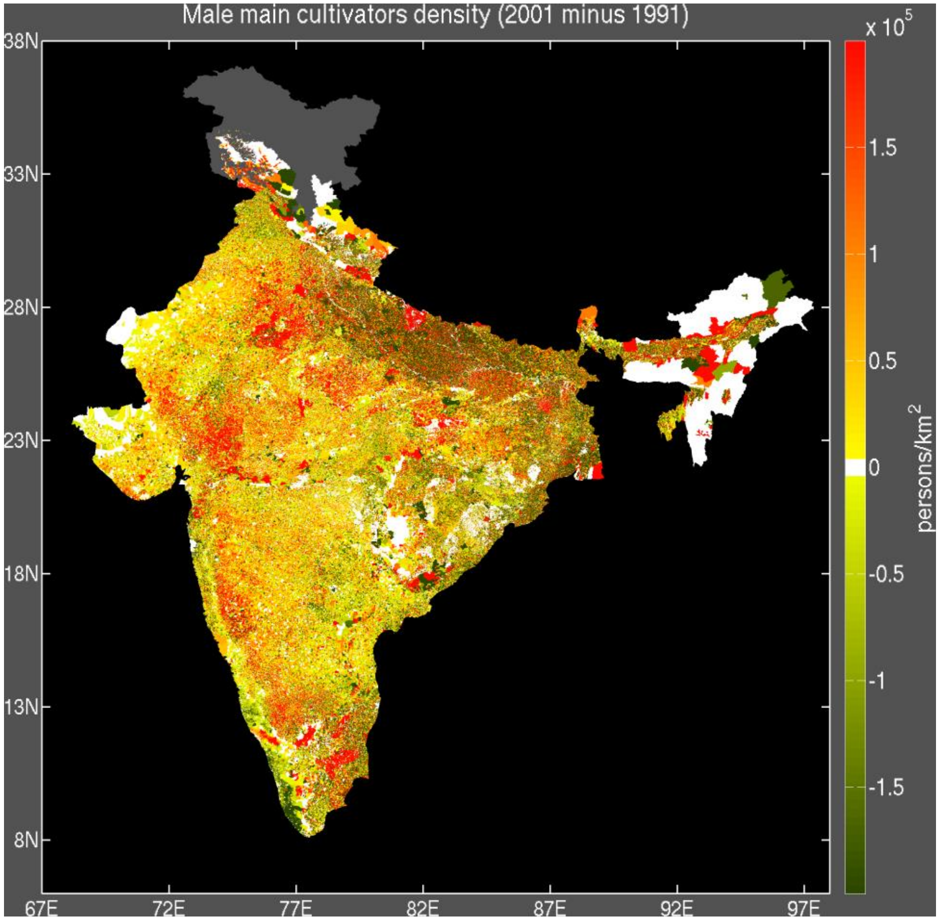

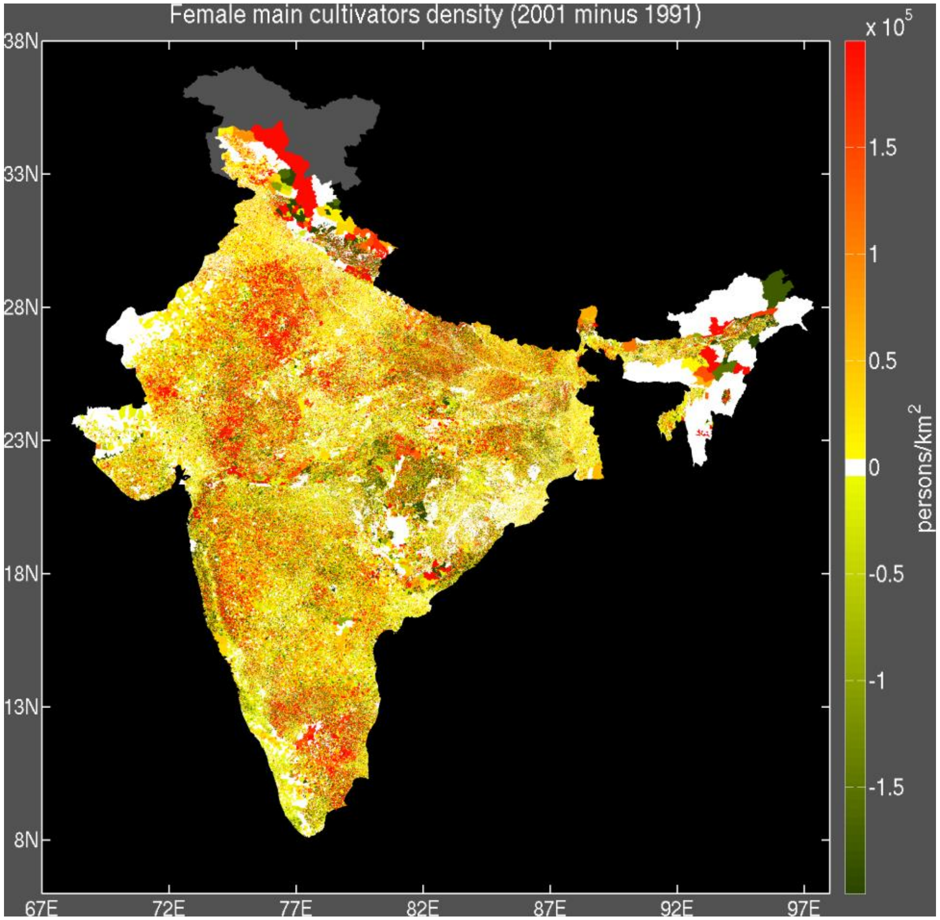

Fig. S6 (Continued). Sub-plots (m) and (n)

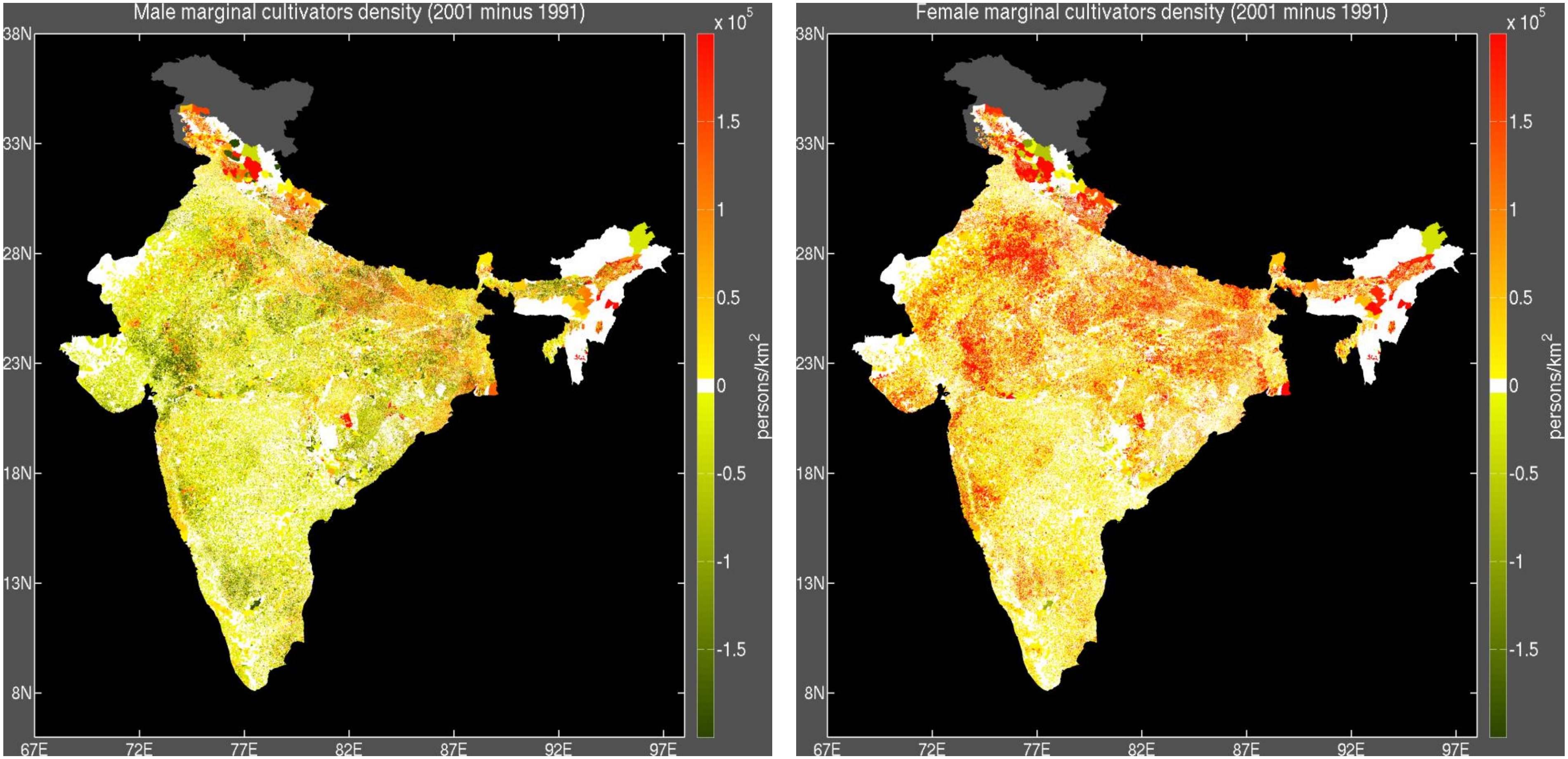

Figure. S7. Similar to Fig. 3a but for AEZ2 (a) 1985-1995, and (b) 1995-2005.

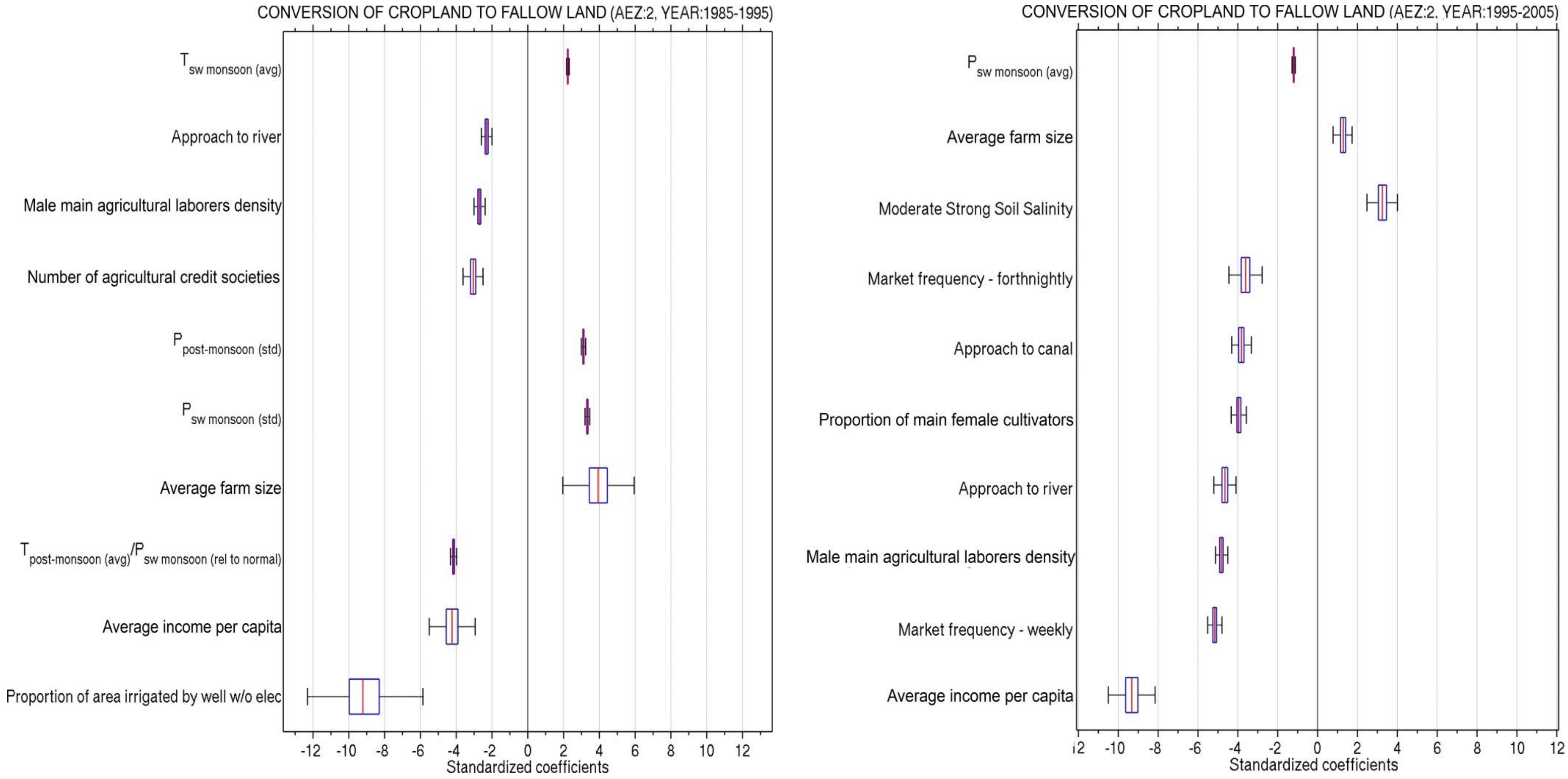

Figure. S8. Similar to Fig. 3a but for AEZ8 (a) 1985-1995, and (b) 1995-2005.

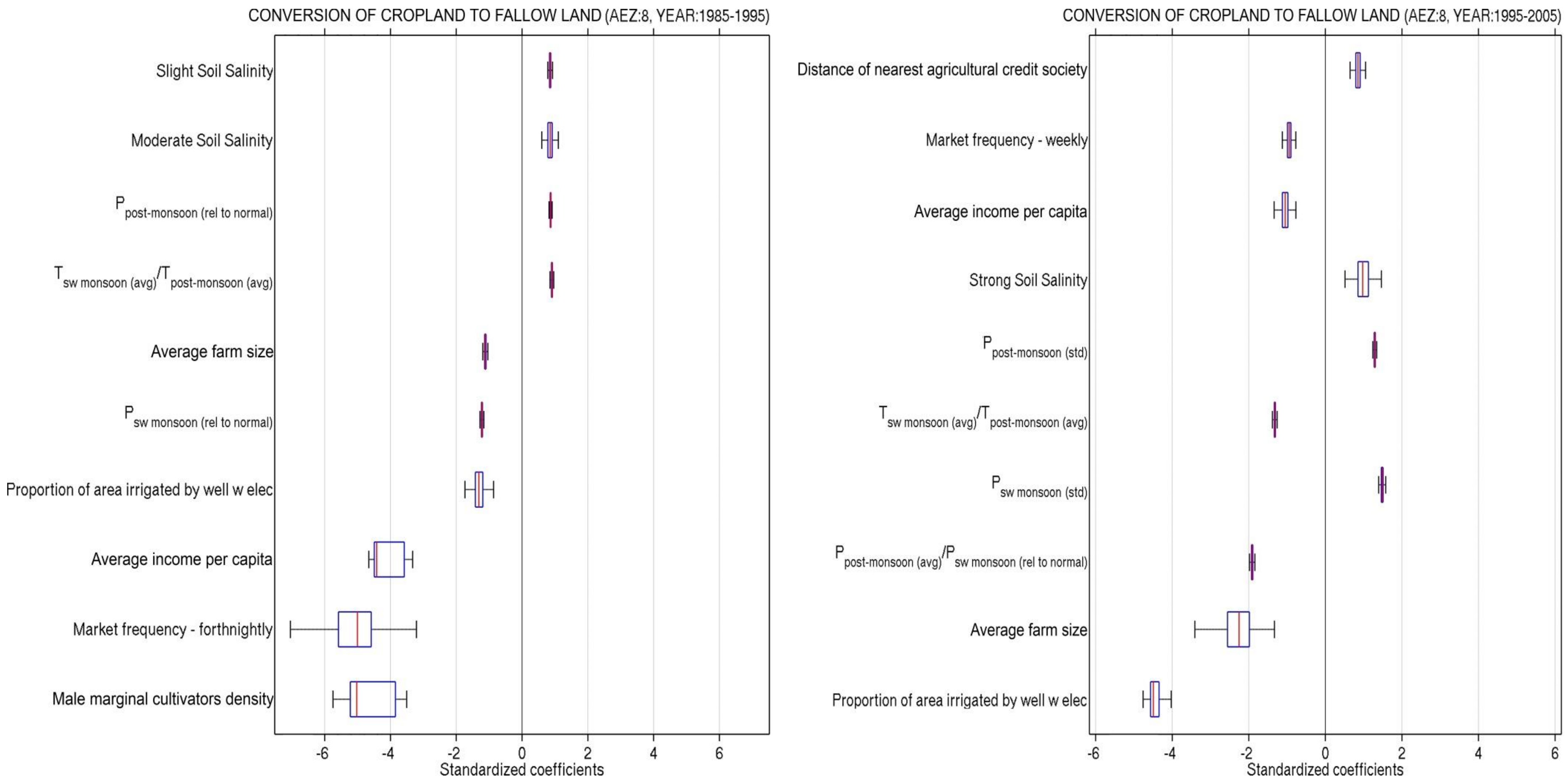

Figure. S9. Similar to Fig. 3a but for AEZ4 and for 1985-1995.

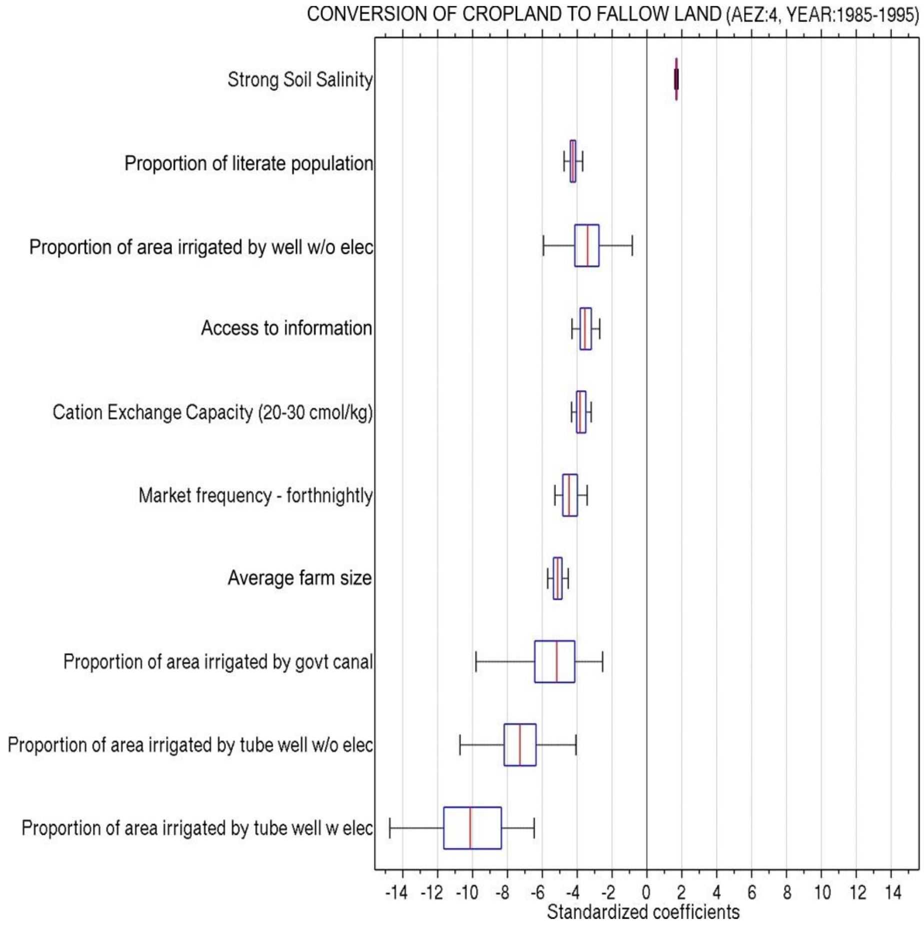

Figure. S10. Similar to Fig. 3a but for AEZ5 and for 1995-2005.

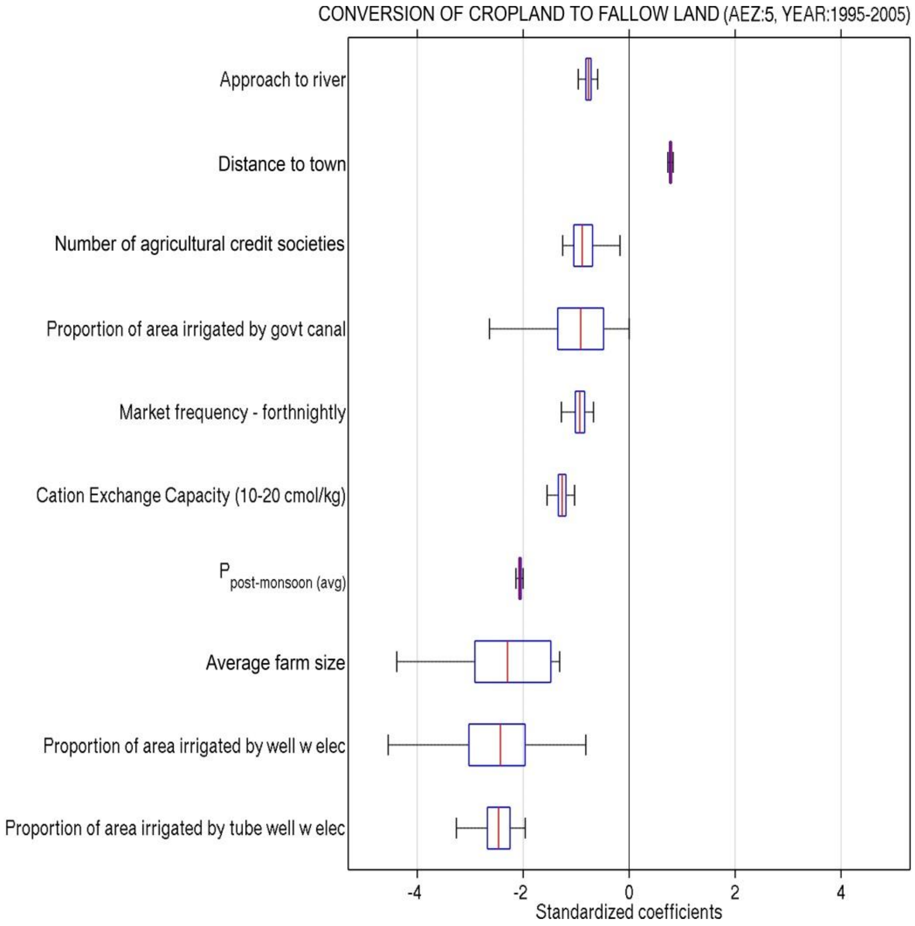

Figure. S11. Similar to Fig. 3b but for AEZ8 (a) 1985-1995, and (b) 1995-2005.

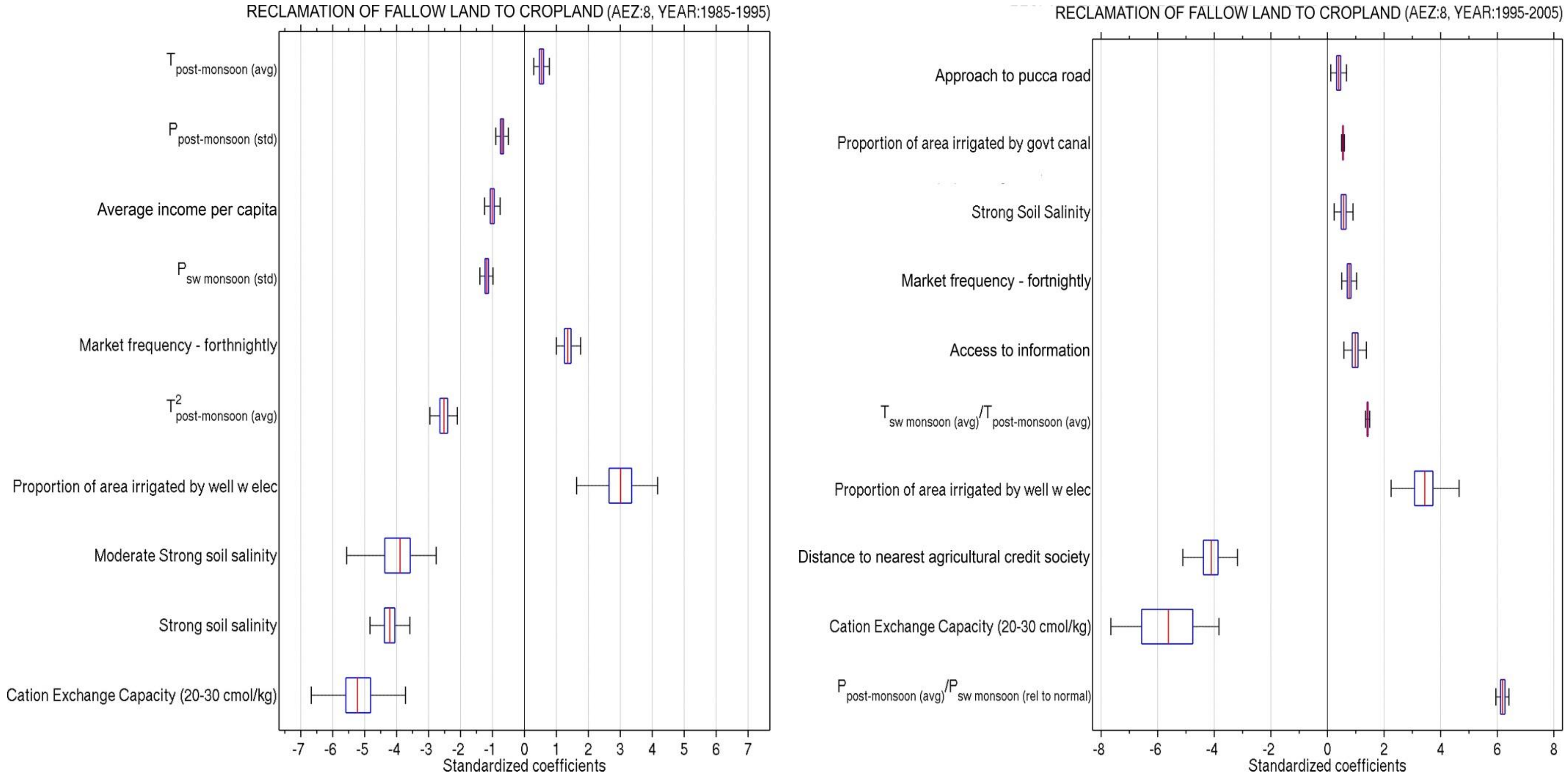

Figure. S12. Similar to Fig. 3b but for AEZ6 and for 1985-1995.

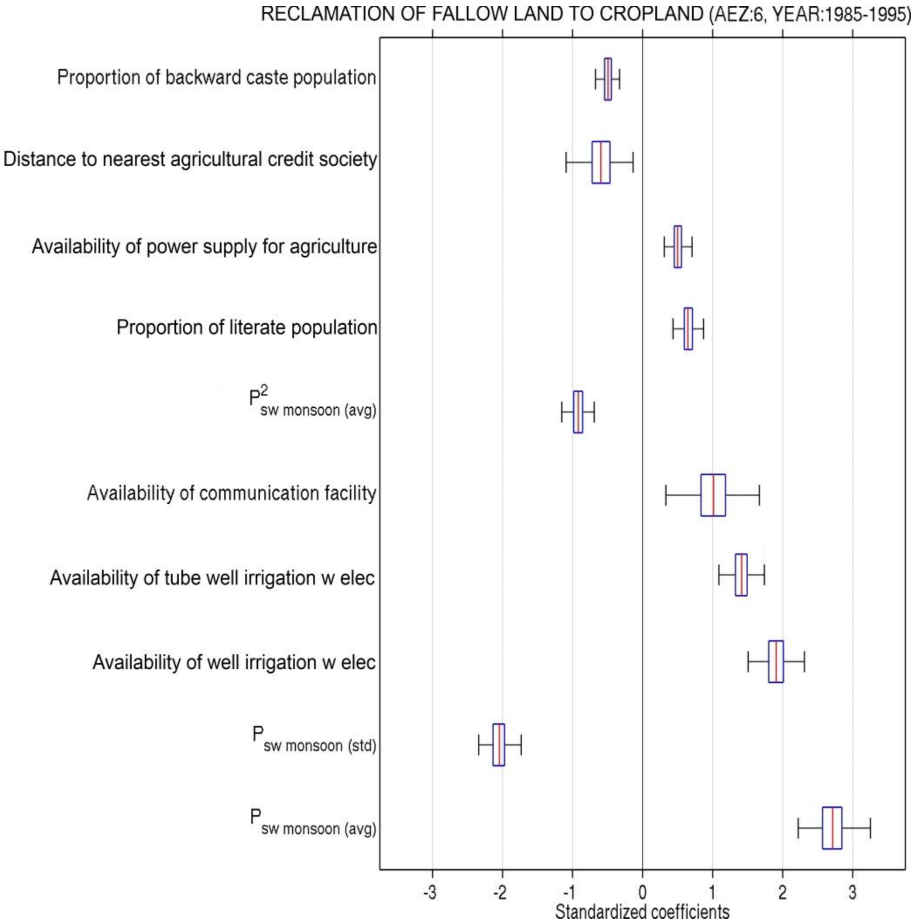

Figure. S13. Similar to Fig. 3b but for AEZ2 and for 1995-2005.

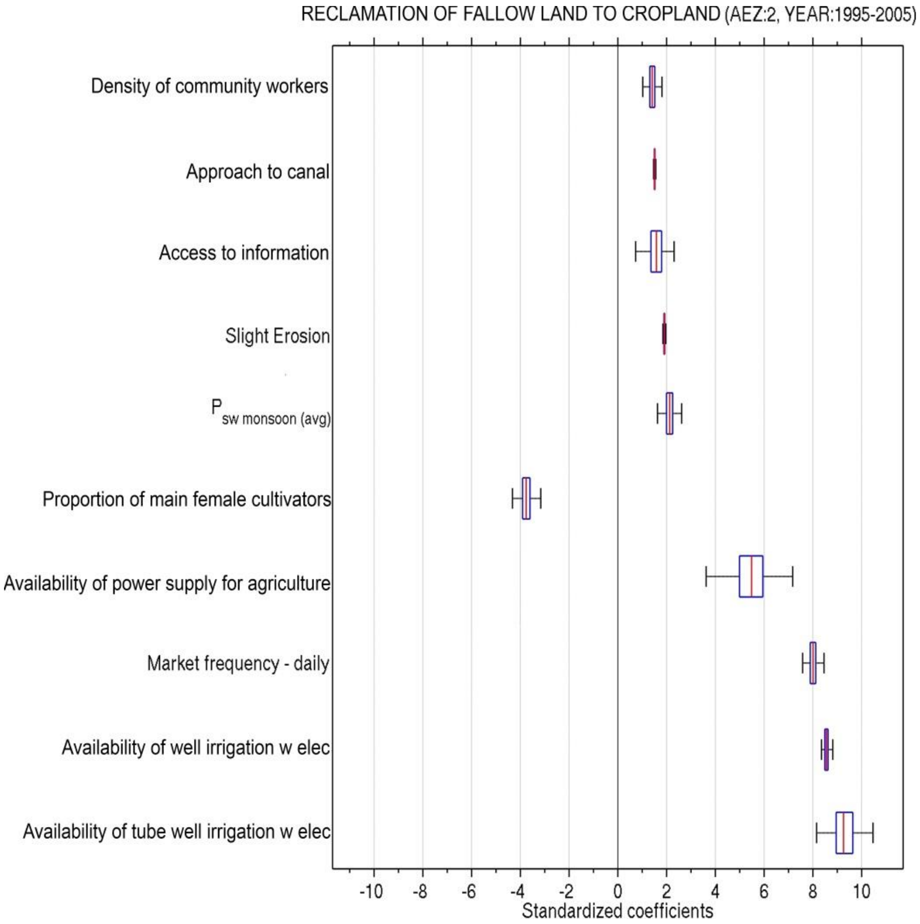

Figure. S14. The series of figures show changes (between 1991 and 2001) in spatial patterns of six key types of irrigation calculated from village level census database (~630,000 political units). (a) Irrigation by well with electricity, (b) Irrigation by well without electricity, (c) Irrigation by tube well with electricity, (d) Irrigation by tube well without electricity, (e) Irrigation by government canal, (f) Irrigation by tanks. The boundaries (black solid lines) show the state boundaries according to 2001 census. The dotted black lines indicate AEZ boundaries.

Sub-plots (a) and (b)

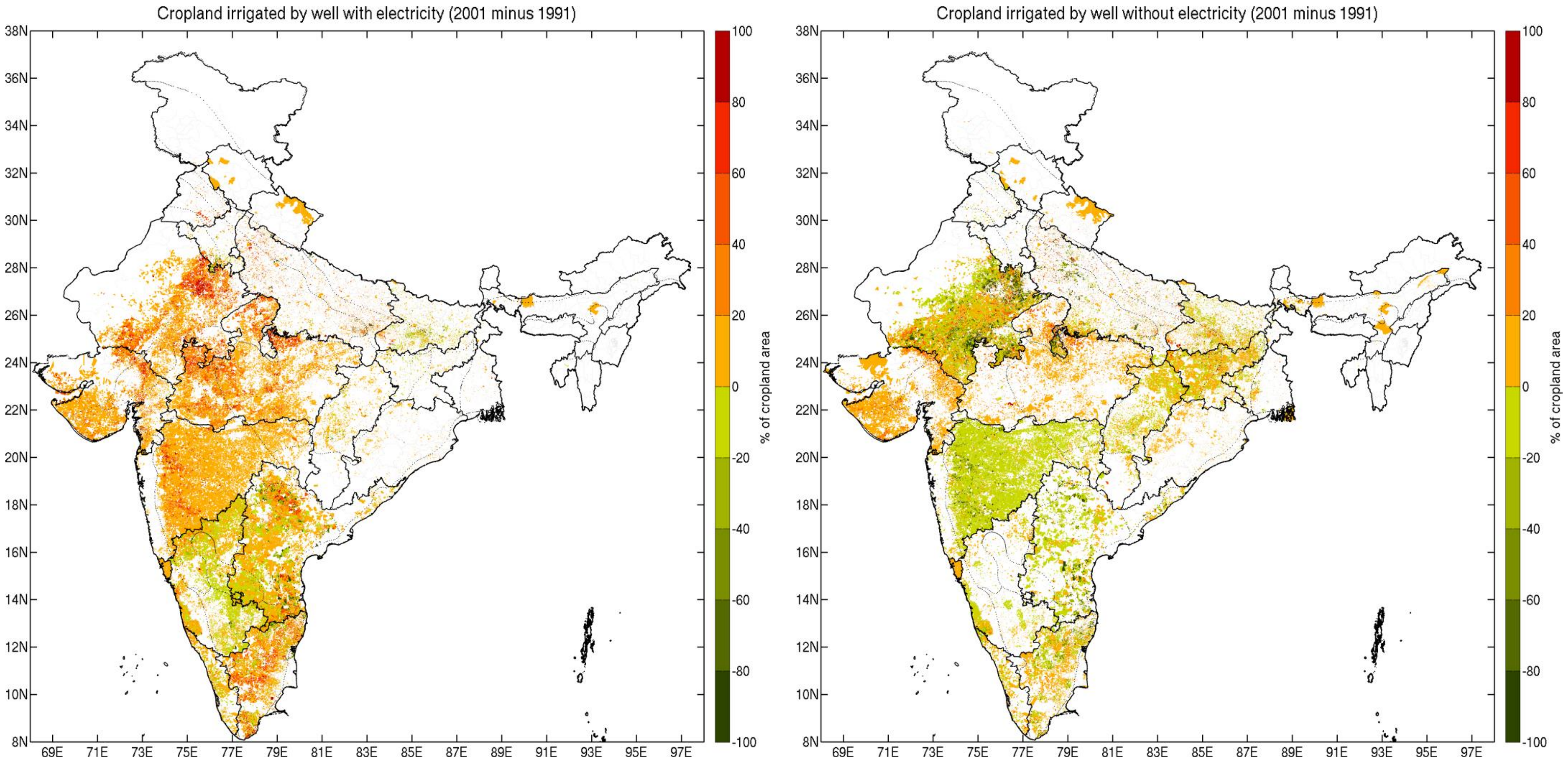

Fig. S14 (continued) Sub-plots (c) and (d)

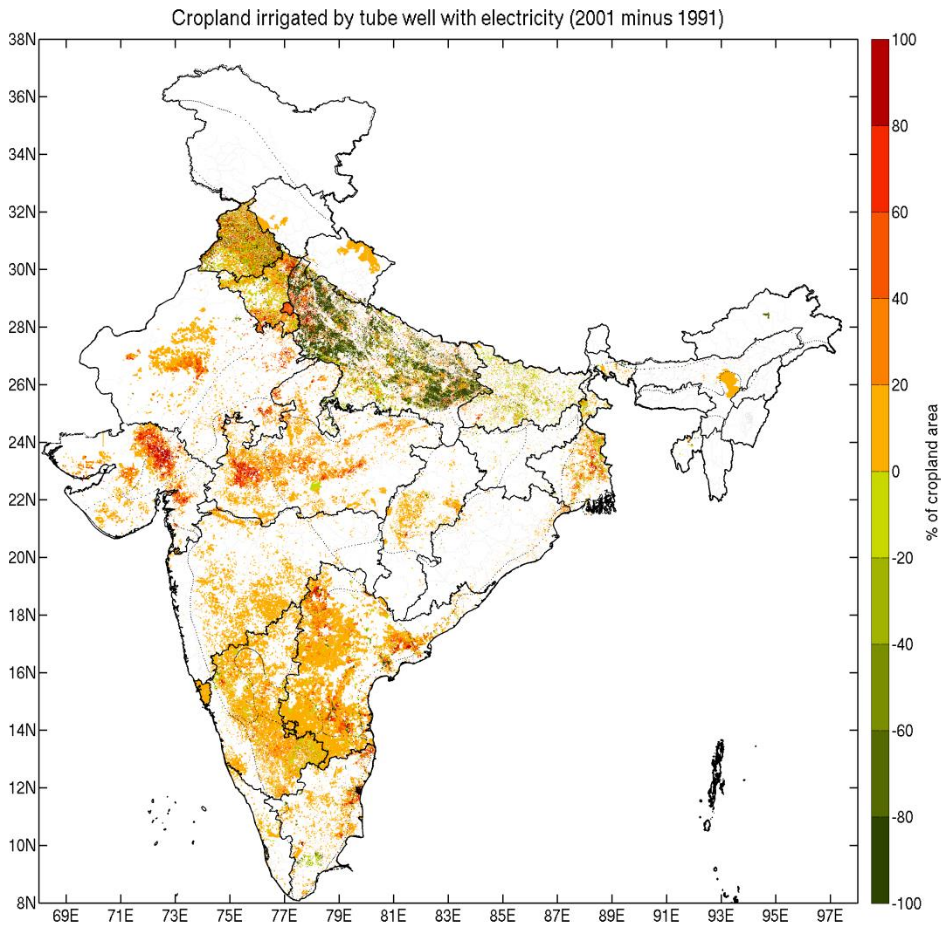

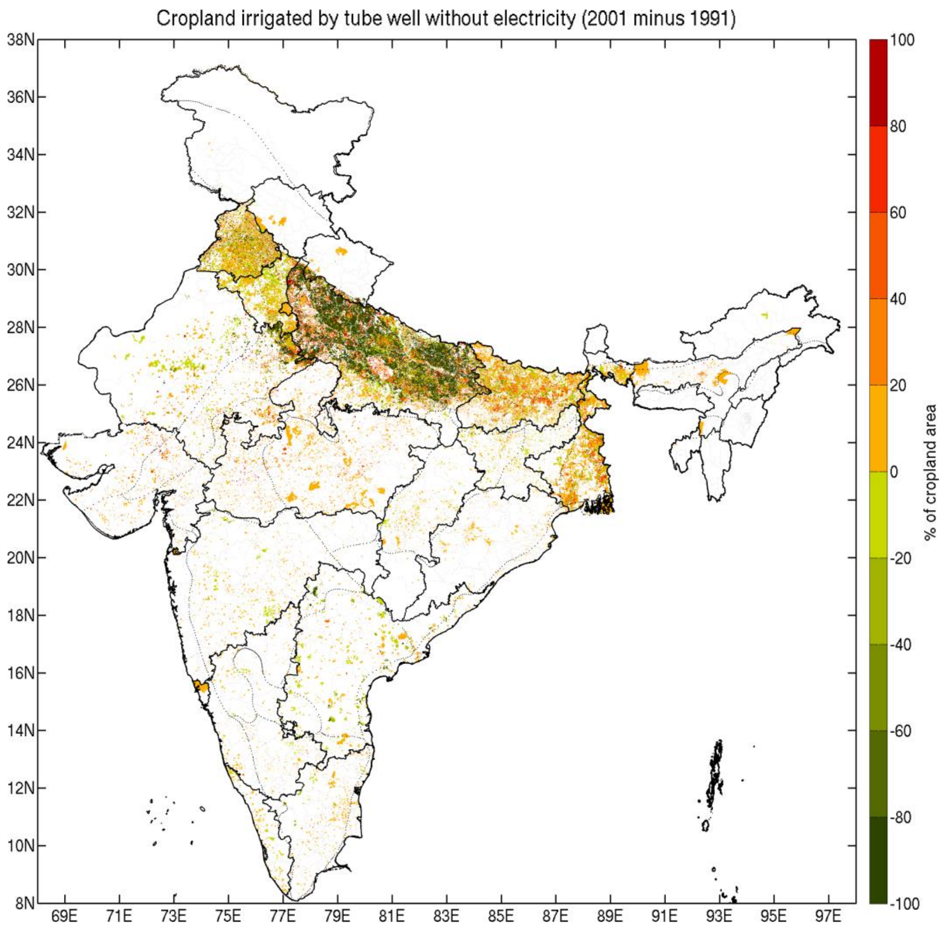

Fig. S14 (continued) Sub-plots (e) and (f)

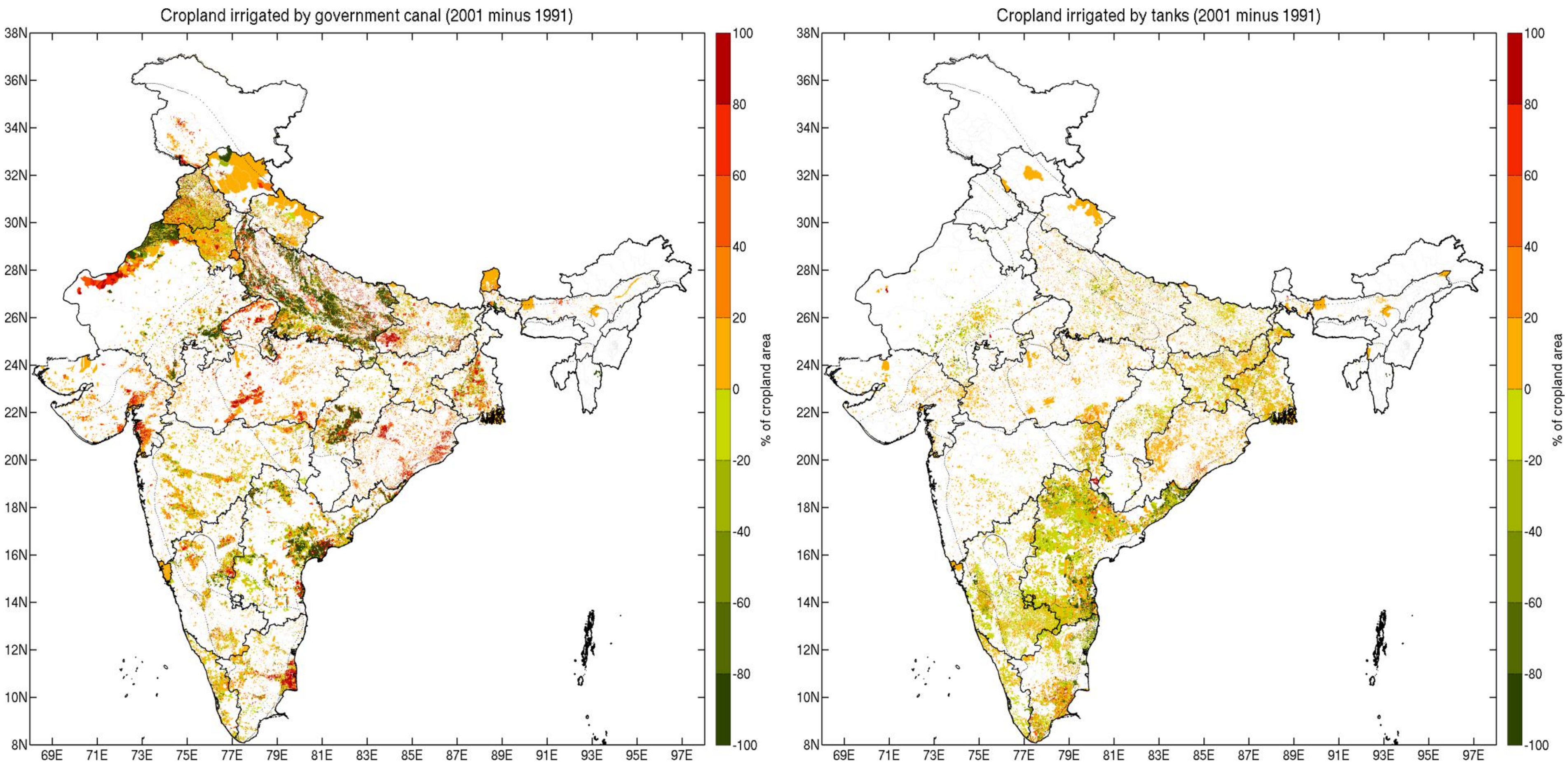

Figure. S15. Similar to Fig. 4a but for 1985-1995.

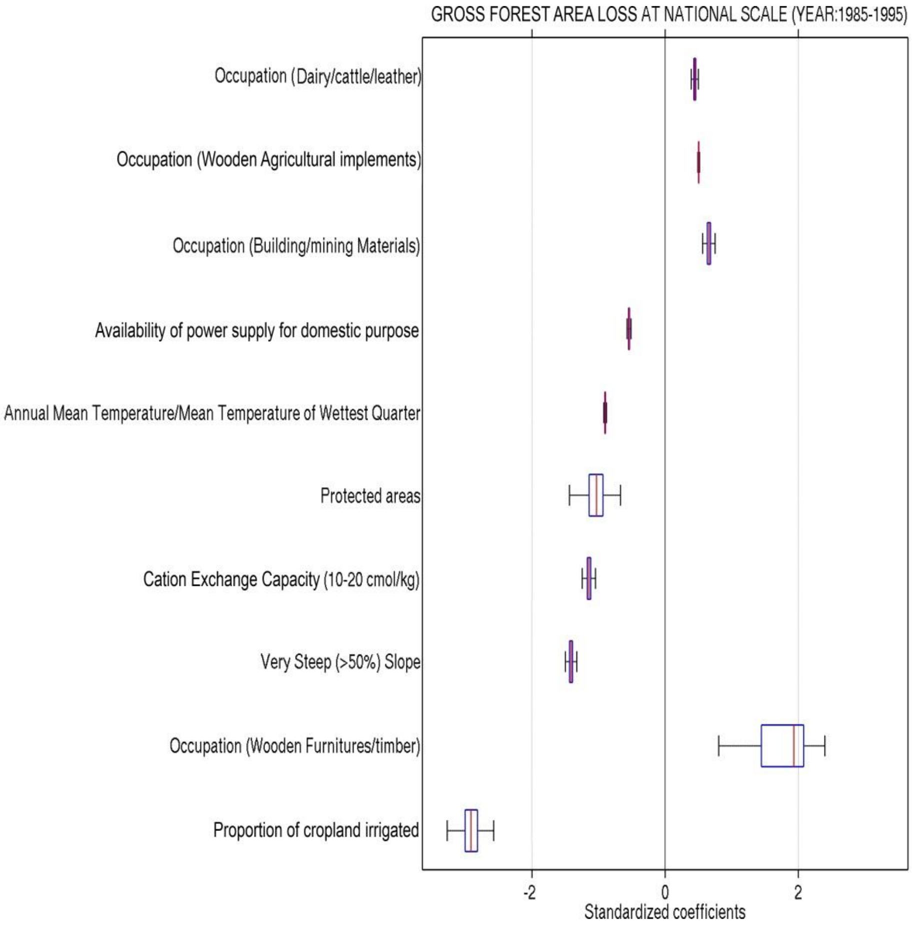

Figure. S16. Grouping of the total forest area loss by type of land protection (y-axis). Analysis is at national scale: (a) 1985-1995, and (b) 1995-2005. Central red line show mean estimate; error bars (blue) show 5% to 95% confidence interval from bootstrap resampling with 500 replicates (to account for spatial autocorrelation); whiskers show 25% to 75% confidence interval.

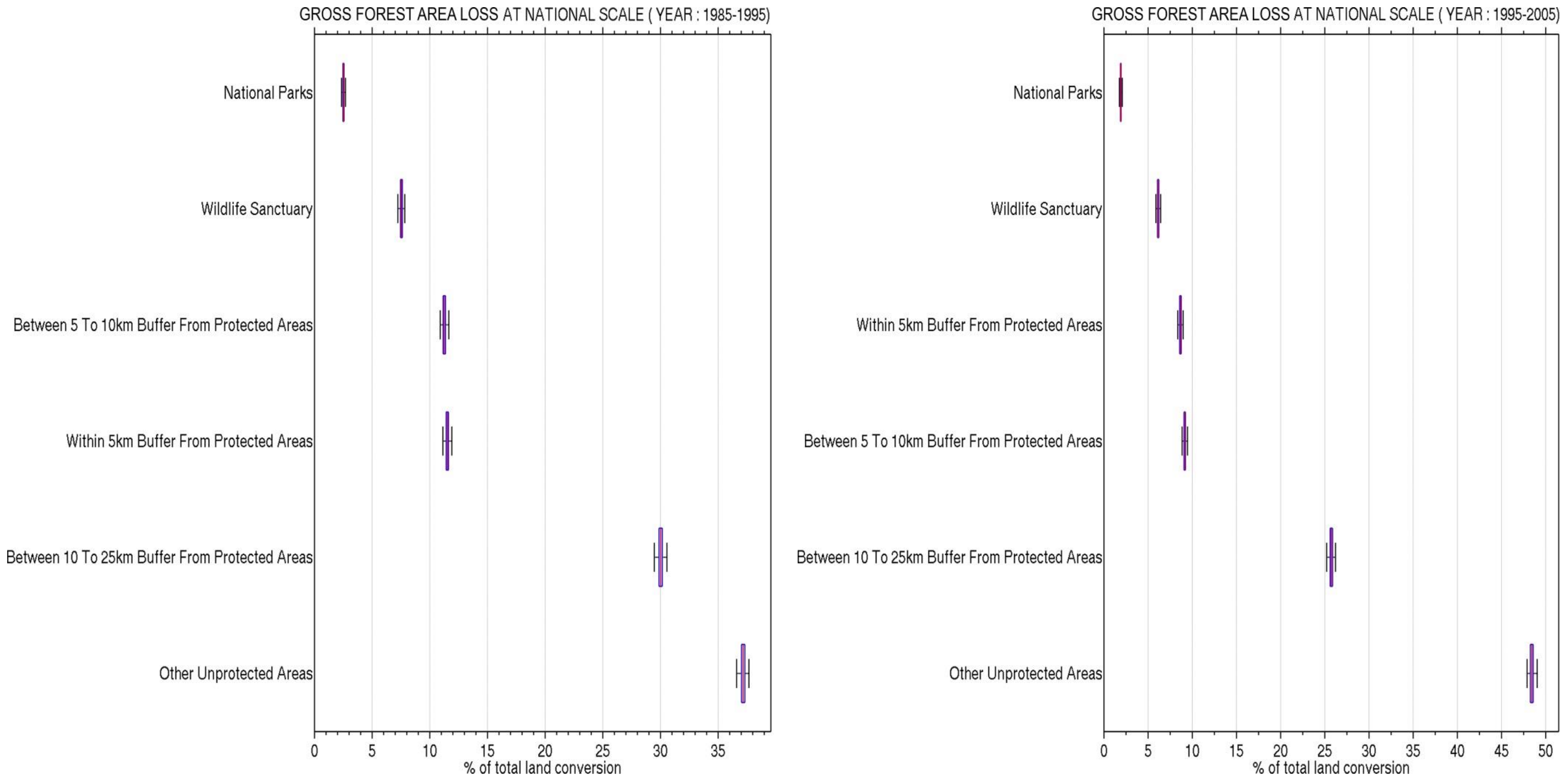

Figure. S17. Similar to Fig. 4a but for AEZ10 (a) 1985-1995, and (b) 1995-2005.

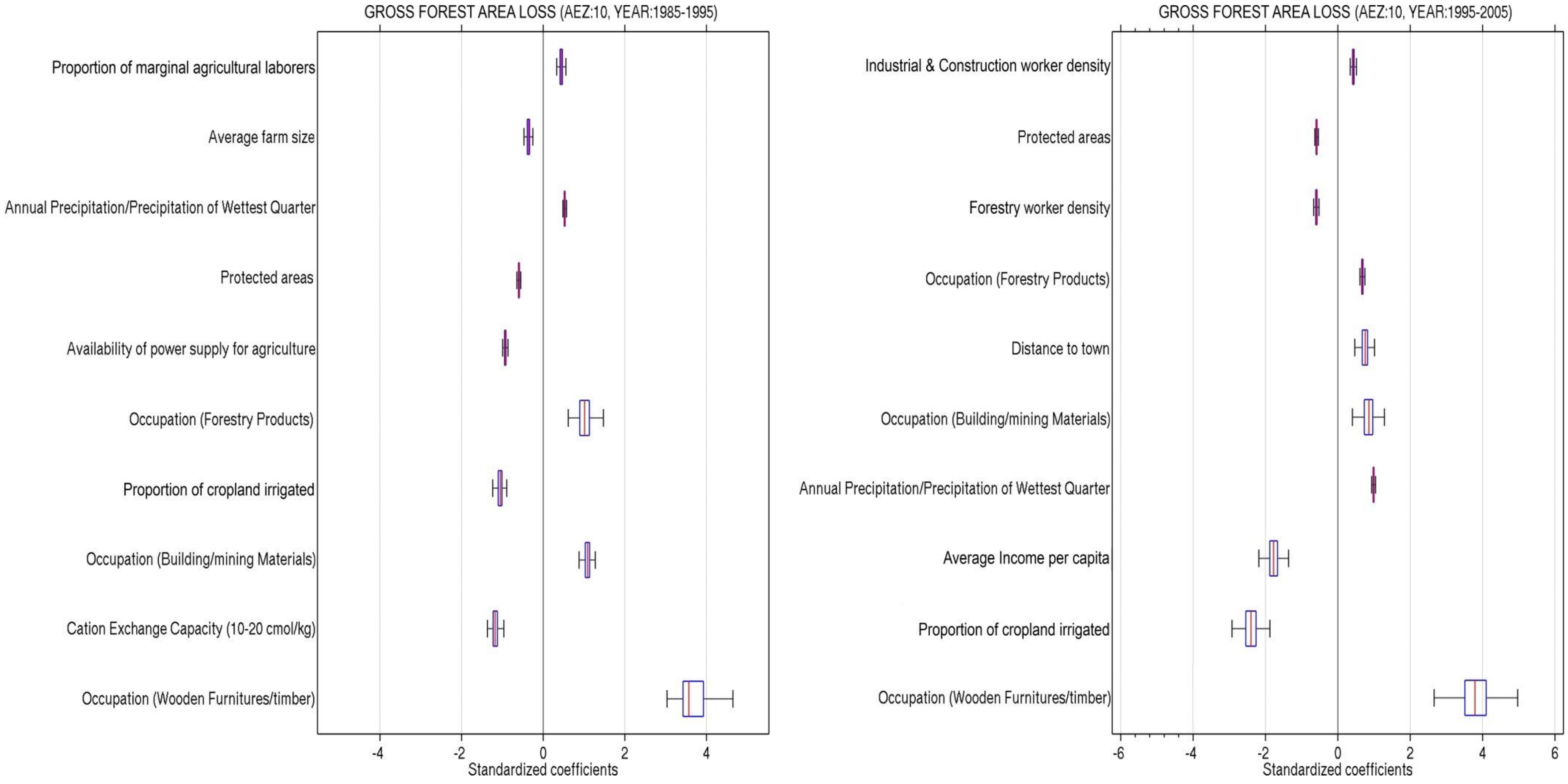

Figure. S18. Similar to Fig. 4a but for AEZ12 (a) 1985-1995, and (b) 1995-2005.

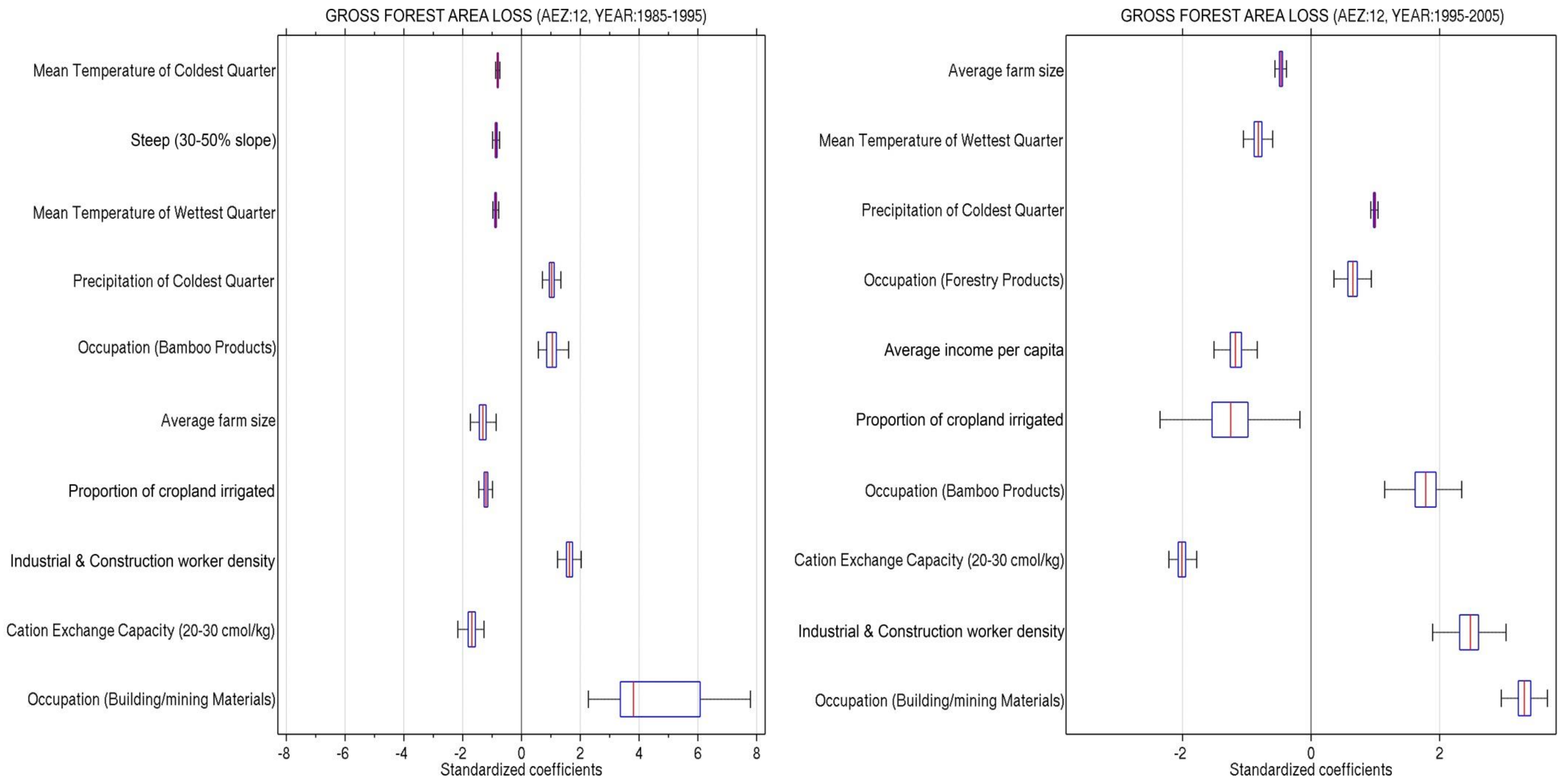

Figure. S19. Similar to Fig. 4a but for AEZ19 and for 1985-1995.

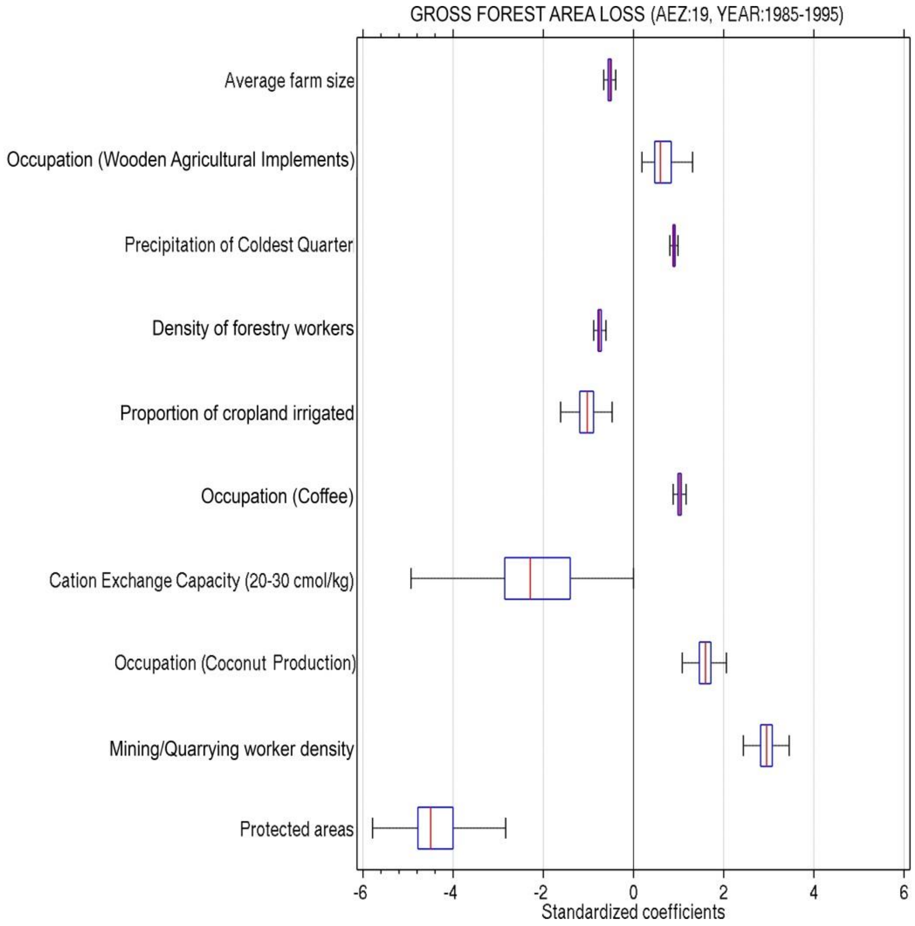

Figure. S20. Similar to Fig. 4a but for AEZ5 and for 1995-2005.

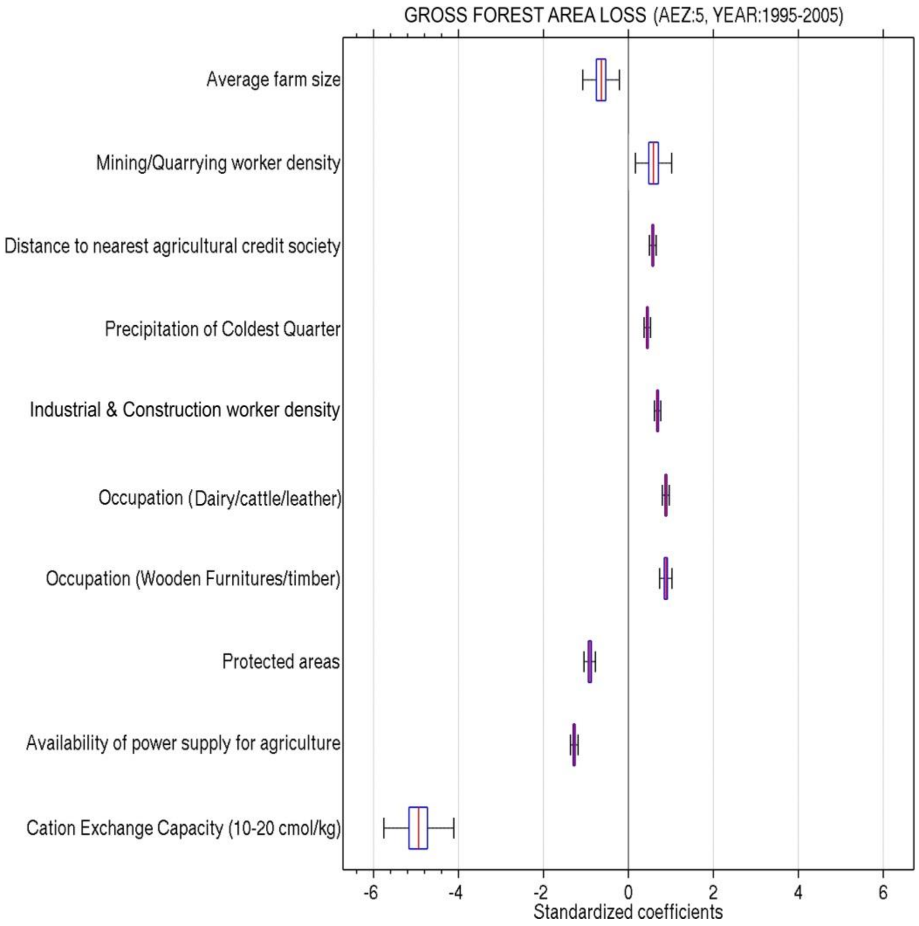

Figure. S21. Similar to Fig. 4a but for AEZ14 and for 1995-2005.

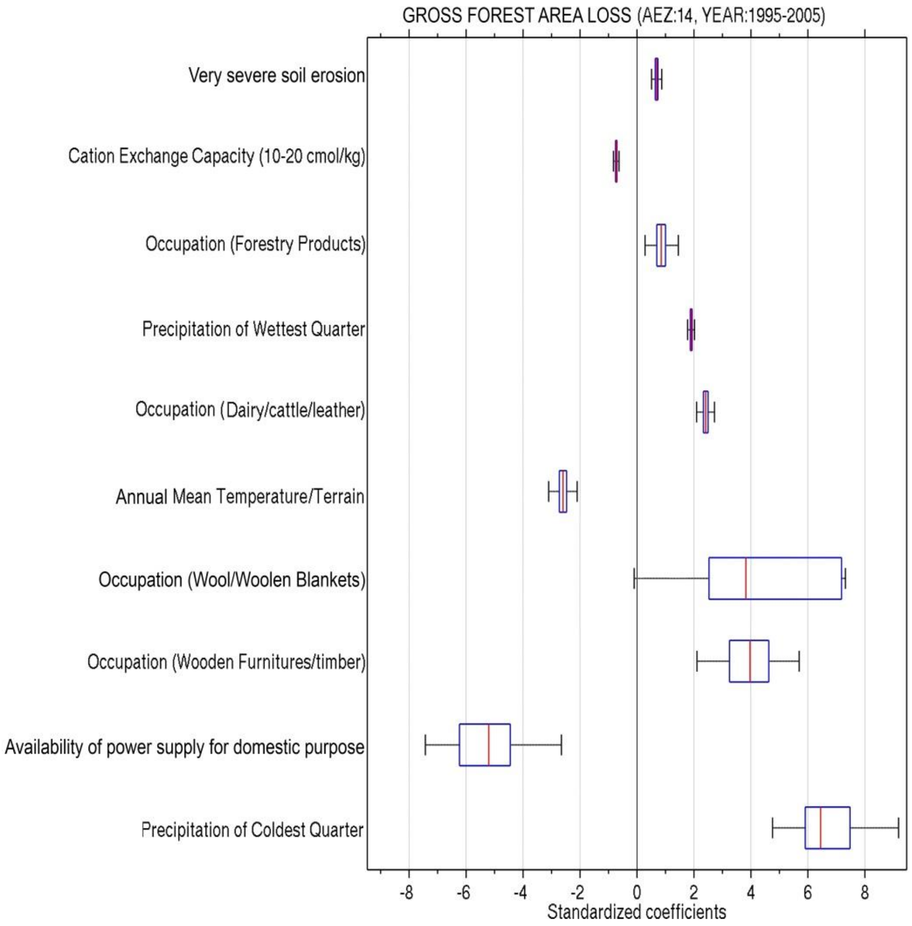

Figure. S22. Similar to Fig. 4b but for 1985-1995.

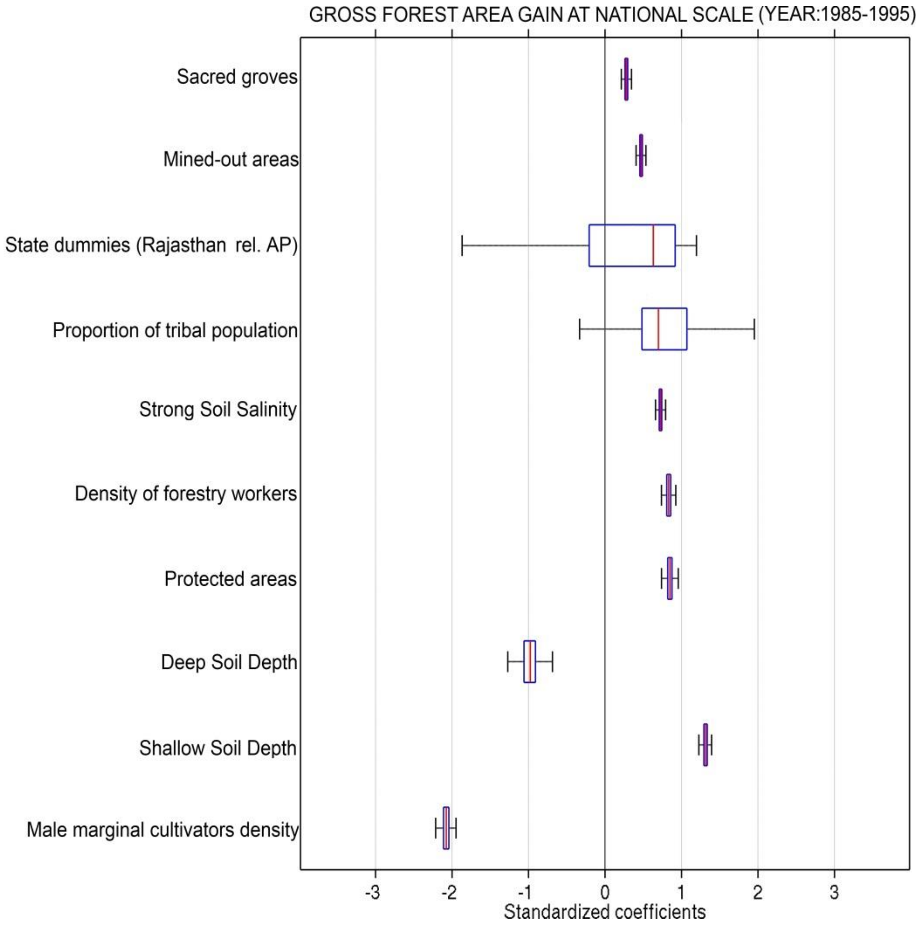

Figure. S23. Similar to Fig. 4b but for AEZ5 (a) 1985-1995, and (b) 1995-2005.

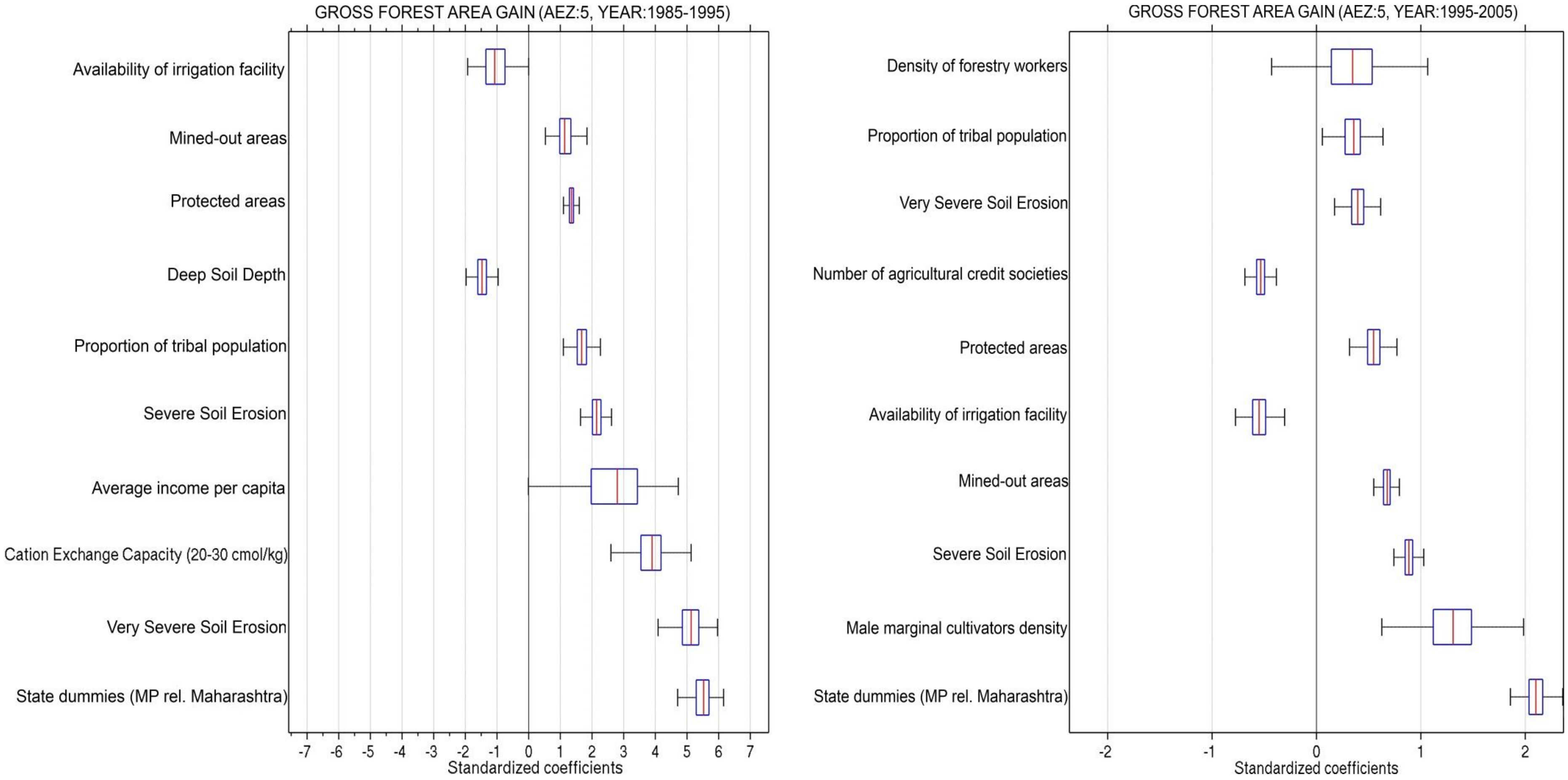

Figure. S24. Similar to Fig. 4b but for AEZ12 and for 1985-1995.

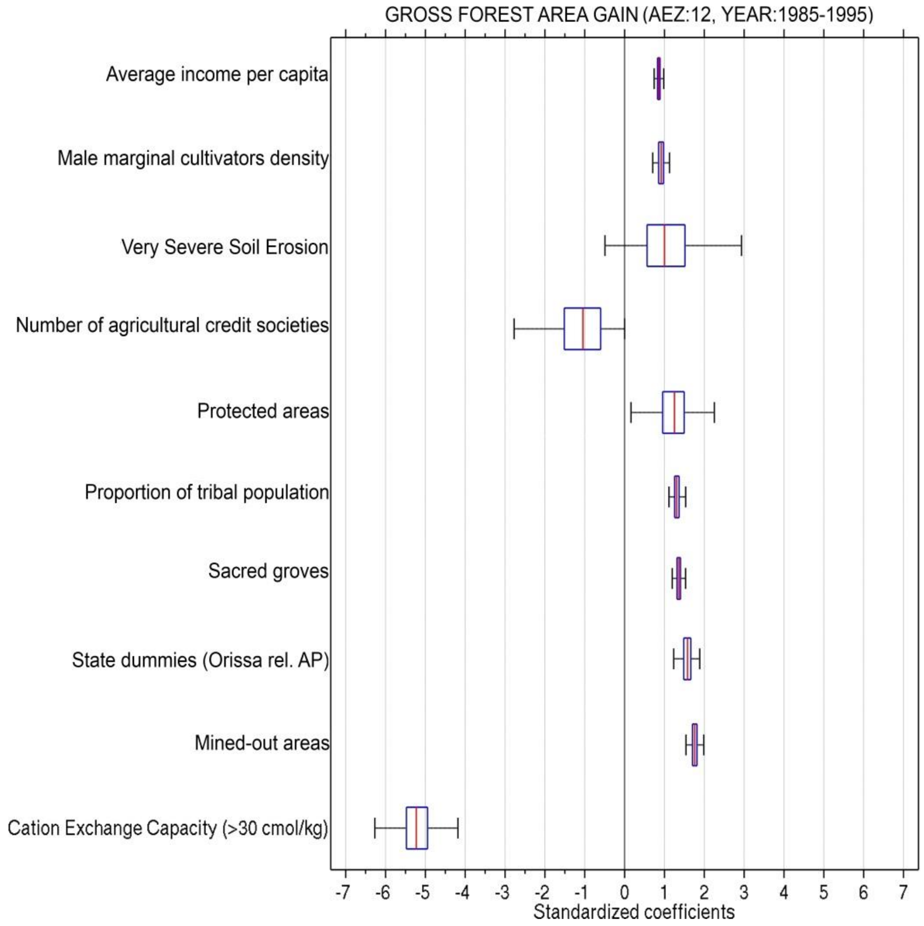

Figure. S25. Similar to Fig. 4b but for AEZ4 and for 1995-2005.

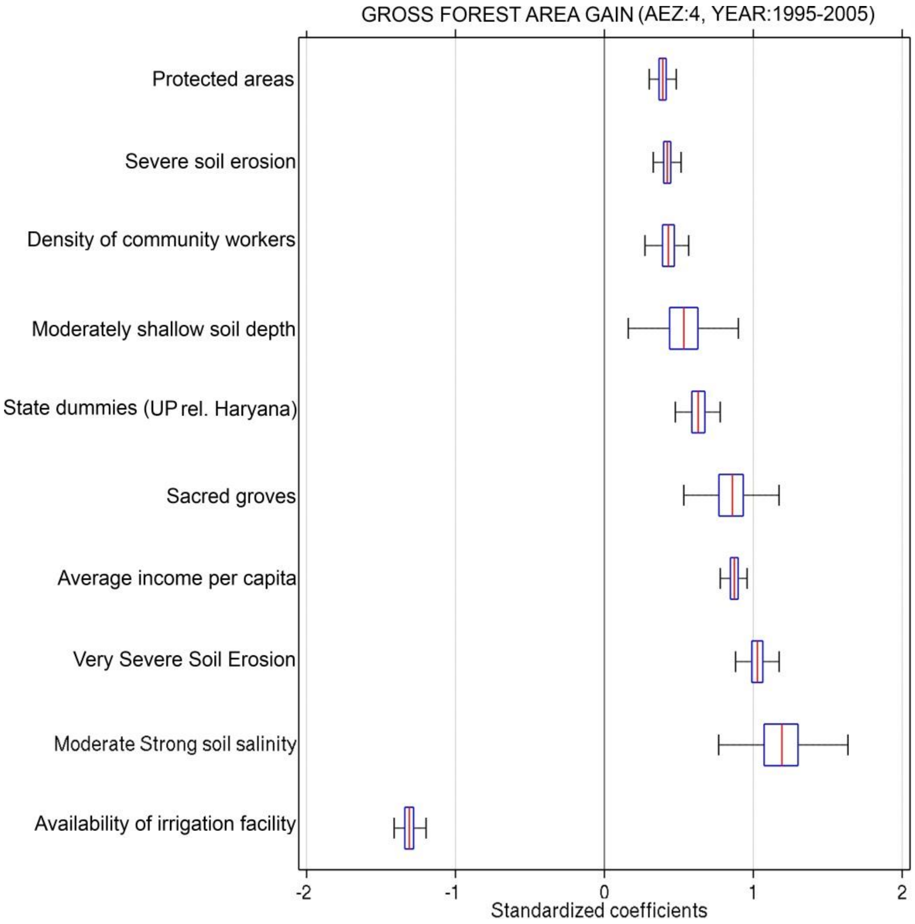

Figure. S26. Similar to Fig. 4b but for AEZ10 and for 1995-2005.

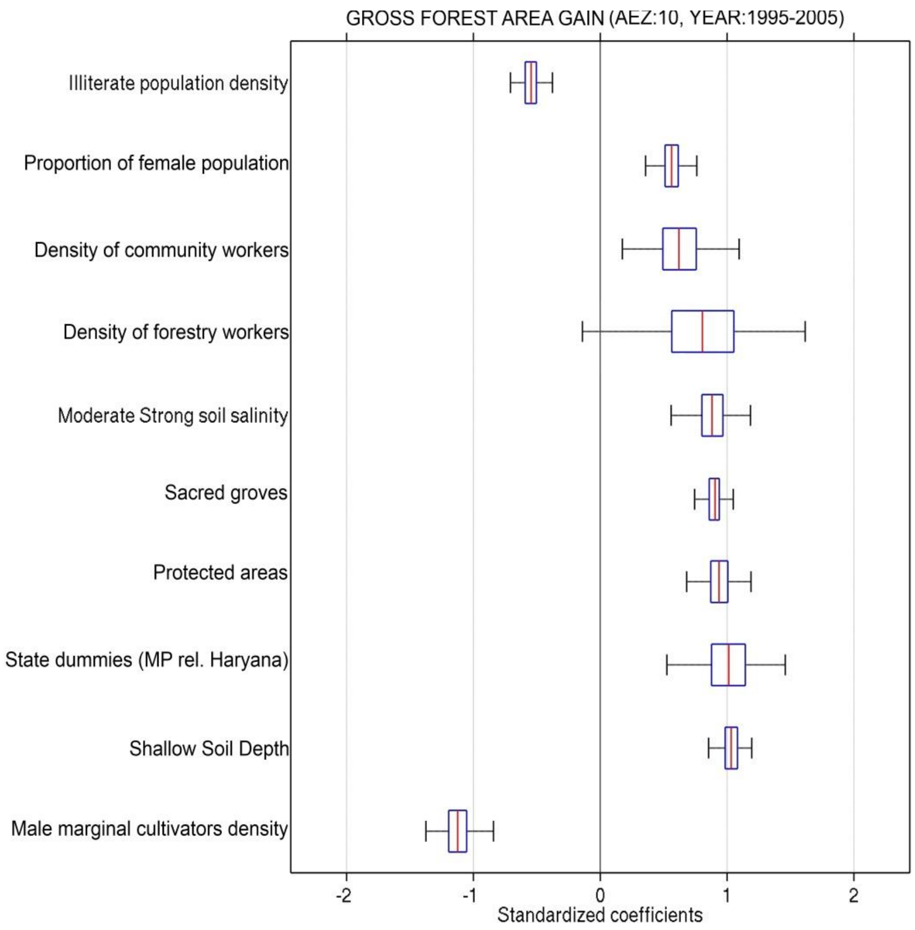

Figure S27. State-wise analysis of forest area diverted to built-up land and water bodies analyzed through Landsat data: (a) 1985-1995, and (b) 1995-2005. For 1985-1995 analysis, state boundaries correspond to 1991 census. For 1995-2005 analysis, state boundaries correspond to 2001 census. For Gujarat state, we have excluded changes within "Rann of Kutch" region which is predominantly covered by shallow wetland which submerges in water during the rainy season and becomes dry during other seasons. Therefore, the changes in water bodies areas observed in this region in our data is because of using Landsat scenes from different season across the decadal maps, thereby representing natural seasonal variations rather than human land conversions.

Sub-plot (a)

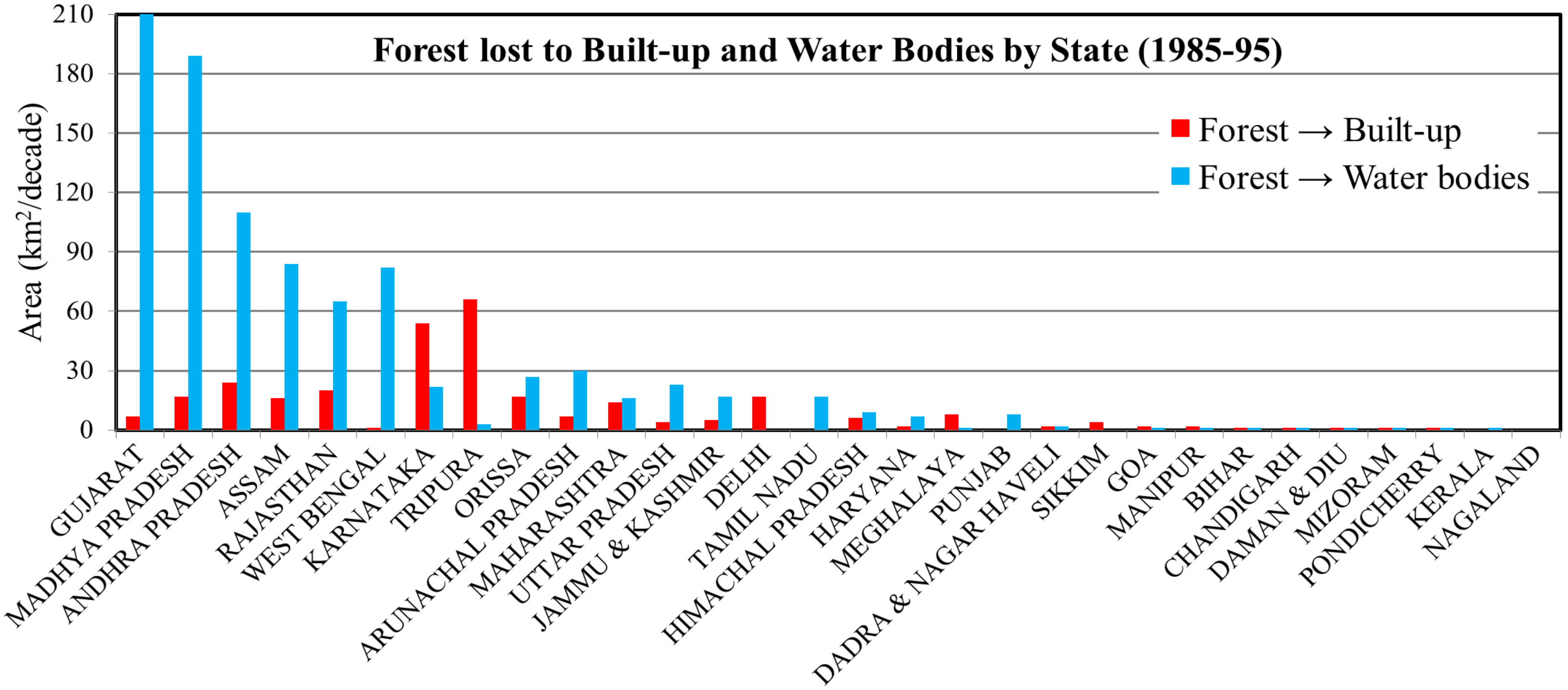

Sub-plot (b)

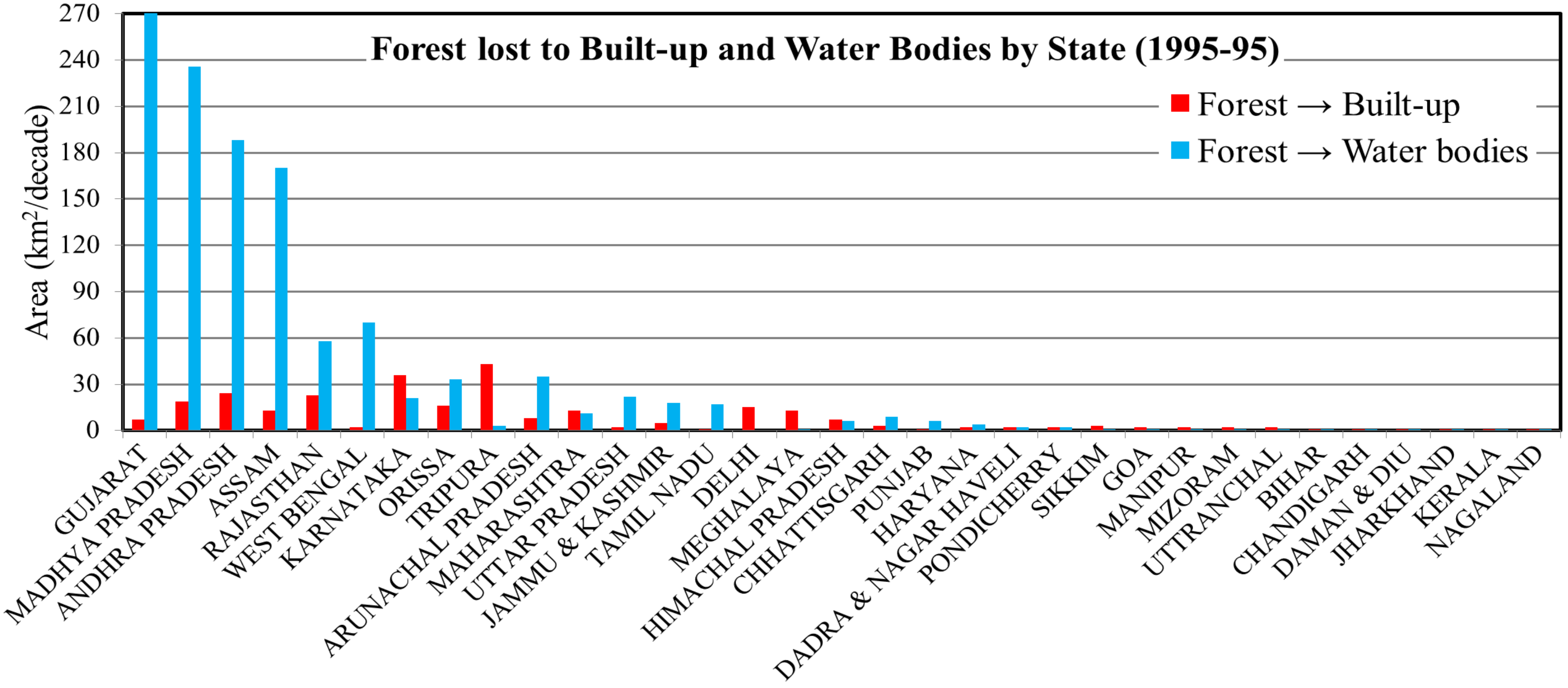
Forest lost to Built-up and Water Bodies by State (1995-95)
Forest → Built-up
Forest → Water bodies
Area (km²/decade)
270
240
210
180
150
120
90
60
30
0
GUJARAT
MADHYA PRADESH
ANDHRA PRADESH
ASSAM
RAJASTHAN
WEST BENGAL
KARNATAKA
ORISSA
TRIPURA
ARUNACHAL PRADESH
MAHARASHTRA
UTTAR PRADESH
JAMMU & KASHMIR
TAMIL NADU
DELHI
MEGHALAYA
HIMACHAL PRADESH
CHHATTISGARH
PUNJAB
HARYANA
DADRA & NAGAR HAVELI
PONDICHERRY
SIKKIM
GOA
MANIPUR
MIZORAM
UTTRANCHAL
BIHAR
CHANDIGARH
DAMAN & DIU
JHARKHAND
KERALA
NAGALAND

Figure S28. Example cross-validation curve from Simulation 1 (Table S14) for $\alpha = 0.4$ (one value of the *10*-fold cross-validation). The sequence of λ's used in the fits is shown in bottom x-axis. The top x-axis show the number of non-zero variables. The type of loss used for validation is binomial deviance (y-axis). The blue points are the mean cross-validated error along the grid of λ sequence; the grey error bars show the upper and lower standard deviation curves along the λ sequence. Two selected λ's are indicated by the vertical yellow dotted lines. The left-most line is the value of λ that gives minimum mean cross-validated error ('best model' corresponding to the chosen value of α). The other λ is the most regularized model such that error is within one standard error of the minimum.

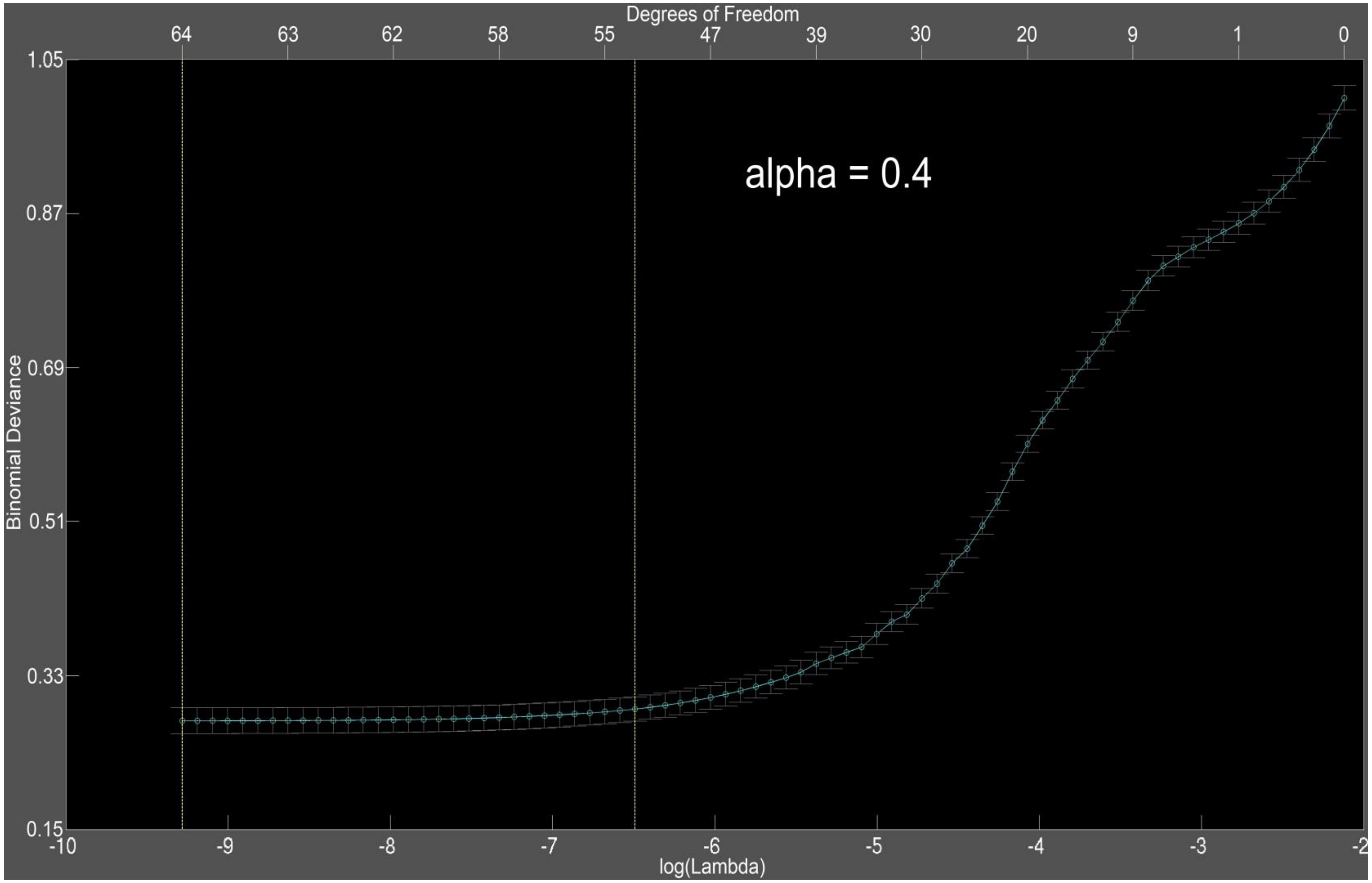

Supplement: Supplementary file 5 — Supplementary material 5 (DOCX 7671 kb) [file 10113_2016_1068_MOESM5_ESM.pdf]
