# Supplementary material for: Dynamics and determinants of land change in India: integrating satellite data with village socioeconomics
Source: Reg Environ Change. 2016 Oct 27;17(3):753–66. doi: 10.1007/s10113-016-1068-2 (PMC7064035; doi:10.1007/s10113-016-1068-2)
Supplement: Supplementary file 6 — Supplementary material 6 (DOCX 615 kb) [file 10113_2016_1068_MOESM6_ESM.pdf]

Dynamics and determinants of land change in India: integrating satellite data with village socioeconomics

Prasanth Meiyappan[a,1], Parth S. Roy[b], Yeshu Sharma[c], Reshma M. Ramachandran[b], Pawan K. Joshi[d], Ruth S. DeFries[e], Atul K. Jain[a,1]

[a]Department of Atmospheric Sciences, University of Illinois, Urbana, IL 61801, USA.

[b]University Center for Earth and Space Science, University of Hyderabad, Hyderabad 500046, India.

[c]Lab for Spatial Informatics, International Institute of Information Technology, Hyderabad 500032, India.

[d]School of Environmental Sciences, Jawaharlal Nehru University, New Delhi 110067, India.

[e]Department of Ecology, Evolution, and Environmental Biology, Columbia University, New York, NY 10027, USA.

[1]**Corresponding Authors**: prasanthnitt89@gmail.com; jain1@illinois.edu (Ph.: 1-217-333-2128)

Journal: Regional Environmental Change

Text S1. Extended methods and data

Study area. Our study area conforms to the official national boundary published by the Survey of India (http://www.surveyofindia.gov.in/) and includes Indian territories currently disputed with China and Pakistan. Our LULCC estimates and analysis of spatial determinants cover mainland India. We did not map/estimate LULCC for two union territories that together account for ~0.25% of India's land area: Andaman and Nicobar Island (mostly comprised of protected forests), and Lakshadweep (mostly plantations).

Data. A summary of key input datasets used in this study is provided in Table S13. LULCC data is fully described in the methods section of the main paper and references cited therein. Here, we expand on rationale and processing of biophysical and socioeconomic variables used in our analysis of spatial determinants. See Table S12 for hypothesized biophysical and socioeconomic variables. Our hypothesis of explanatory variables was grounded based on synthesis of case studies (detailed in subsequent sub-section).

Biophysical data. Given the significant impacts of climate change on Indian agriculture (Singh et al. 2002; O'Brien et al. 2004; Kumar et al. 2004; Mall et al. 2006; Lobell et al. 2008; Aggarwal 2008; Guiteras 2009; Auffhammer 2012; Lobell et al. 2012; Mondal et al. 2014; Mondal et al. 2015), we included seasonal mean temperature and precipitation as potential explanatory variables in simulations aimed at understating conversions between cropland and fallow land. We defined seasons following Kumar et al. 2004: winter, summer, southeast monsoon, and autumn/post-monsoon season.

We derived seasonal precipitation from the daily gridded product of Indian Meteorological Department (Table S13). The data is the most comprehensive gridded product for India, as it integrates most number of rain gauge stations (N>7000). We derived seasonal temperature from the APHRODITE daily temperature product for Monsoon Asia (Table S13). Both precipitation and temperature products were at 0.25^{o}x0.25^{o} lat/long which is relatively coarser in spatial resolution compared to our LULCC dataset; however, there are no gridded time-varying climate dataset at higher spatial resolution covering our study period (1985-2005). Available higher spatial resolution climate products are either static (Hijmans et al. 2005), or cover shorter periods (2000-present) for temperature only (Huete et al. 2002). We addressed the issue of difference in spatial resolution

among LULCC, biophysical datasets, and socioeconomic datasets by bringing all datasets to a common spatial resolution of 1kmx1km lat/long for our analysis of spatial determinants as detailed in "data processing" sub-section.

According to agronomic studies, crop growth is partly a non-linear function of weather conditions, especially temperature (Schlenker and Roberts 2006; Schlenker and Roberts 2008; Schlenker and Roberts 2009; Lobell et al. 2011). To account for non-linearity, we included squared terms for seasonal temperature and precipitation variables as potential explanatory variables in our analysis. Additionally, to test for interactive effects, we included the within season interactions between precipitation and temperature as potential explanatory variables.

For simulations aimed at understanding changes in forest area, we used bioclimatic variables (Hijmans et al. 2005) available at higher spatial resolution (1km x 1km lat/long), and static representative of contemporary climatic conditions. We chose the static data over coarser resolution (but transient) data products as higher spatial resolution was a priority over transient information, to study forest area change at decadal time scale.

To test for the effects of soil conditions on land-cover conversions, we included a wide range of soil parameters across all simulations: depth, reaction (pH), drainage, slope, erosion, salinity, sodicity, cation exchange capacity (measure of soil fertility and nutrient exchange capacity), flooding, and organic carbon content. All soil parameters were ordinal variables, and the data was sourced from the National Bureau of Soil Survey and Land Use Planning, India (Table S13).

Socioeconomic data. Our geospatial socioeconomic database covers over 200 variables at the highest level of spatial disaggregation in India: ~630,000 administrative units at village/town level (Fig. S1). We collected tabular data for two consecutive census years (1991 and 2001) from "Primary Census Abstract" and "Village Directory" data series of the Indian census. We used a sub-set of these variables for our analysis of spatial determinants; the sub-set being specific to land-cover conversion studied (Table S12). We derived some hypothesized socioeconomic variables by combining two or more census variables, for example, we derived average heads per household by dividing "total village population" with "total village households".

Our village level spatial database has two key advantages. First, these variables show high granularity (Fig. S2) that is important to explain the spatial variation in high-resolution land-cover conversion estimates (i.e. Landsat data). The granularity will get masked even at taluka level, thus making them less suitable for our analysis (Fig. S2). Second, several key explanatory variables are village-specific categorical variables (e.g. infrastructure such as market availability, availability of power supply for agriculture, primary village occupation) that becomes irrelevant at coarser administrative levels (unlike population that can be aggregated).

We started by collecting tabular data for each village/town for both 1991 and 2001 census from the online digital database of the Census of India (http://censusindia.gov.in/). We undertook extensive data cleaning to ensure the data are quality-controlled, and standardized. Our next step was to convert the village/town tabular data into geospatial data. This required administrative boundaries at village/town level at national scale corresponding to census years. Official Geographic Information System (GIS) information on the administrative boundaries of India are made accessible (restricted) only at (sub-) district level (note the broad administrative hierarchy in India: state>district>sub-district>village/town). Therefore, we created a national level GIS file for 2001 with village/town boundaries by digitizing boundaries from the official cadastral maps published by the Survey of India. We also capitalized on village/town level GIS files for several states that were already available to us from earlier projects. We designed algorithms to link both the 2001 and 1991 tabular data to 2001 GIS boundary file, accounting for region-specific data limitations described next.

It should be noted that in some regions, data quality may be poor due to misreporting, human-errors in computerization, quality of village/town boundaries, or data missing due to political strife (e.g. parts of Jammu and Kashmir). We excluded villages/towns from our analysis for which data for all variables were missing. For villages/town where some variables were missing, we did multiple imputations following Baraldi and Enders (2010). We replaced each missing value with a set of plausible values that represent the uncertainty about the right value to impute. We then analyzed the multiple imputed data sets using standard procedures (Baraldi and Enders 2010) for complete data and combine the results (point estimates and standard errors) from these analyses. In

general, we found 1991 census data to be more error-prone, likely because it was the first census when officials were trained to digitize village level information. While our algorithm corrected for many erroneous outliers that were apparent, in some cases, it was hard to detect if the outliers were real, or erroneous. In such cases, our statistical estimation may have been influenced by these errors, but unlikely to have influenced our conclusions. Furthermore, we reduced the impacts of such errors on our final results through bootstrap resampling (wrapped around data imputation), where we resampled the observations 500 times, and each time we fit a new model to the data (described in "statistical estimation" sub-section).

Data processing for statistical estimation. Following earlier land change modeling studies (e.g. Serneels and Lambin 2001; Verburg et al. 2006; Sohl et al. 2012), we brought all data to a common spatial resolution for statistical estimation. By experimentation, we chose 1km x 1km lat/long resolution as the best tradeoff between the fine-resolution (30m) LULCC data, and relatively coarse resolution transient climate data (~27km), and socioeconomic datasets (~2.2km on an average calculated by dividing India's geographical area by the number of administrative units in our village/town GIS file). In aggregating the land-cover conversion estimates from 30m to 1km grid cells, we calculated fractional values of each land-cover conversion within each 1km grid cell, so as to retain maximum information. This is unlike discrete spatial aggregation, where the entire area within 1km grid cell is approximated to either undergo one (dominant) type of land conversion, or remain unchanged. Our statistical estimation methods are specifically designed to handle fractional LULCC outcomes, consistent with our data aggregation method.

We used standard bilinear interpolation to downscale the coarser-resolution (transient) temperature and precipitation data to 1km x 1km lat/long resolution. For socioeconomic datasets, we assumed all grid cells falling within a village/town to have the same values as that village/town (note that all continuous variables were normalized by village/town area before gridding to 1km). For grid cells that fall on multiple village/town, we followed two approaches depending on the nature of variable. For continuous variables (e.g. population density), we calculated area weighed averages. For categorical variables (e.g. availability of irrigation facility), we took the value of village/town that has maximum area falling within the grid cell.

The independent variables used in our analysis have different units and scales (order of magnitude). Therefore, the statistical estimates cannot be interpreted directly to make standardized comparisons across explanatory variables. We addressed this issue by standardizing the explanatory variables across observations prior to statistical analysis. We apply standardization only to continuous variables. For categorical (and ordinal) variables, we dummy coded and used them in the model without applying any transformation. The dummy coding converts a categorical variable with 'k' levels into a set of 'k' binary variables. We dropped one variable from each set of dummy variables representing a categorical variable in the model. This is because, in the presence of an intercept (constant) term in the model, inclusion of all dummies from a categorical variable will result in perfect multicollinearity, a scenario known as "dummy variable trap".

We standardized each continuous variable using standardized z-score, with a modification. For each continuous variable, we calculated two statistics from the set of observations: the mean and standard deviation. We computed the z-score standardized value in each grid cell by subtracting the mean from the actual value, and then dividing by standard deviation. The subtracting by mean centers the data to have mean zero, but is strictly not necessary for variable standardization; centering is typically helpful to interpret the main effects in the presence of interactions. The division by standard deviation scales the data to have one standard deviation. This is the typical z-score standardization. However, in the presence of binary variables that are roughly symmetric (with equal probabilities of 0 and 1; hence, with a mean and standard deviation of 0.5), the model estimated coefficients for binary variables correspond to a comparison of two standard deviations, and hence cannot be compared directly with z-score standardized continuous variables that correspond to one standard deviation. Therefore, to put both categorical and continuous variables on same scale, we further divided the z-score standardized continuous variables by 2 following Gelman (2008).

Notably, to derive the standardized squared seasonal climate variables (Table S12), we first standardized the seasonal climate variables, and then squared the standardized variables. We do not standardize the squared seasonal climate variables itself. In other words, we standardized the continuous input variables, not the explanatory variables (predictors) themselves. Similarly, we

multiplied the standardized seasonal precipitation and temperature terms to derive the standardized interaction terms.

Rationale for inclusion of state-fixed effects as potential explanatory variables (Table S12). Natural resource management in the Indian constitution falls under three categories: national, state, and concurrent. Forest is concurrent listed; forest policies are made at national level, and state implements them. State also manages its forest (including compensatory afforestation); however, clearing forest for other land use requires prior approval from the central government.

Agricultural land in India is under private holdings. State deals with market creation, national food secure procurement, minimum price fixation, and provides necessary infrastructure to support agriculture. Further, every state has its own agriculture universities which support farmers with extension work. National government is involved in funding projects such as irrigation, and soil conservation. Therefore, state plays a key role in both agriculture and forestry.

Our statistical estimation for analysis of spatial determinants is done either at national level or for regional hotspots (identified by AEZs; see "analysis" sub-section for rationale), meaning the set of observations can belong to different states. Across all simulations, we tested for state-fixed effects (e.g. agriculture and forest policies) that may not be captured by variations in biophysical and socioeconomic variables. We accounted for state-fixed effects by inclusion of state-specific dummies as explanatory variables in our statistical model (one column made as reference category). Our state boundaries are based on Survey of India (censusindia.gov.in). We used 1991 and 2001 state boundaries corresponding to simulations covering 1985-1995 and 1995-2005 decades respectively.

Analysis.

Rationale for analysis of spatial determinants by AEZ, and by epoch. We break down our national-scale analysis into 19 sub-regions based on Agro-Ecological Zones (AEZs) specifically developed for India (Table S6; Gajbhiye and Mandal 2000). There are two reasons for identifying regional hotspots for our analysis of spatial determinants by AEZ. First, in Indian context, AEZs are the optimal units for macro-level land use planning and efficient transfer of technology as India's

economy is highly dependent on agriculture and allied sectors including forestry (Alagh 1996; Velayutham et al. 1999; Mandal et al. 2014). Second, for statistical modeling, it is desirable to delineate regions by similar characteristics to avoid heteroscedasticity (occurs when sub-populations have different variability from others). AEZs by definition are regions delineated by similar soil and climatic conditions, and exhibit homogeneity in LULCC processes (Alagh 1996; Velayutham et al. 1999; Gajbhiye and Mandal 2000; Mandal et al. 2014). Typically, AEZs are also used as optimal units for modeling LULCC in global-scale economic models that simulate the interactions among socioeconomics, LULCC, and climate change, for e.g. GTAP-AEZ (Lee et al. 2009) and GCAM (Kyle 2011). Earlier studies (NRC 2014; Agarwal et al. 2002; Verburg et al. 2004; Briassoulis 2000; Parker et al. 2003; Irwin and Geoghegan 2001) have shown that the set of factors and their importance to determining LULCC (e.g. conversion of cropland to fallow land) vary with time. To account for temporal variations we estimated statistical models separately for the two decades (1985-1995 and 1995-2005).

Time considerations for linking decadal LULCC data to concomitant explanatory factors. As our LULCC estimates are assessed from decadal Landsat imageries, they capture only the decadal trends in LULCC, and therefore can mask the within-decade variations in LULCC (e.g. year-to-year conversions between cropland and fallow mainly driven by inter-annual variations in rainfall).

Common to all simulations (Table S14), we assumed the decadal LULCC reflects the net result of time-varying socioeconomic forces (typically change gradually with time) that acted within the respective decade. The census in India is conducted once in 10-years, and we assumed 1991 socioeconomics to reflect the time-averaged conditions between 1985 and 1995, and 2001 socioeconomics to reflect the time-averaged conditions between 1995 and 2005. Accordingly, we used 1991 socioeconomics to relate with LULCC in 1985-1995, and 2001 socioeconomics to relate with LULCC in 1995-2005.

Earlier studies have shown that cropland area in India is sensitive to inter-annual variations in climate, especially rainfall (Tables S8, S9). The decadal changes between cropland and fallow land inferred from Landsat will reflect only the climate effect of end year of the respective decade.

Therefore, for simulations aimed at understanding conversions between cropland and fallow land, we related 1994-95 averaged climate variables to LULCC between 1985 and 1995, and related 2004-05 averaged climate variables with LULCC between 1995 and 2005.

Statistical estimation.

Overview. Our methodology for relating the spatial (at 1km x 1km lat/long) patterns of land-cover conversion (dependent or response variable) to concomitant biophysical and socioeconomic factors (or their proxies; the independent or explanatory variables) originates from our recent work (Meiyappan et al. 2014). Our method is broadly consistent with land change modeling literature (NRC 2014; Agarwal et al. 2002; Verburg et al. 2004; Briassoulis 2000; Parker et al. 2003; Irwin and Geoghegan 2001; Lesschen et al. 2005). Each simulation listed in Table S14 is subject to the statistical analysis detailed below.

The overall approach can be broken down into four steps. First, we select observations (i.e. grid cells within the study area e.g. AEZ hotspot) based on which we estimate the model. Second, we explain the "fractional" binomial logistic regression that we use to model the relationship between the dependent and independent variables. The model allows for fractional outcomes in dependent variables, consistent with our Landsat-based LULCC data aggregation technique. Third, we detail the algorithm to account for multicollinearity across independent variables. As a safety check (prior to step 3), we ensured that no highly collinear variables (Pearson's $r > 0.9$) were present in the model. Finally, we explain the how we estimate and interpret the regression coefficient.

Selecting observations. For ease of understanding, we explain this section with an example of Simulation 1 (i.e. conversion between cropland to fallow land at national scale between 1985 and 1995; Table S14). The approach however is similar across all simulations.

As we are interested in conversions between cropland and fallow land, we masked out grid cells within the study region (i.e. national for Simulation 1) where both cropland and fallow land (fallow land + wasteland) area in initial year (1985) was zero. In the masked grids, both cropland to fallow land conversion or the reverse conversion cannot occur between the two time points. For statistical estimation, we included all grids where non-zero cropland area in 1985 was converted to

fallow land by 1995 (i.e. the conversion of interest). We further included buffer grids around zones of cropland to fallow land conversion. In buffer grids, all cropland either remained unchanged between 1985 and 1995, or had seen reverse conversion (i.e. fallow land to cropland). We selected the buffer size such that summed across observations, the area of cropland converted to fallow land (1985-1995) roughly equaled the counter-factual (sum of area of cropland that remained unchanged between 1985 and 1995 or have undergone reverse conversion). The buffer size is simulation specific (Table S14), and depends on the spatial confirmation of the land change patterns typically varying between 1.5 and 7 km.

The two response variables in our "fractional" binomial logistic regression are (Simulation 1): (1) the cropland area in each grid cell (during 1985) that was converted to fallow land by 1995 $('l'=1)$, and (2) the counter-factual which is the sum of cropland land area in each grid cell that remained unchanged between 1985 and 1995, and fallow land area that was converted to cropland between 1985 and 1995 (i.e. reverse conversion) $('l'=2)$. We normalized the response variables in each grid cell by the sum of cropland and fallow land area during 1985 in that grid cell, so that sum of responses adds to 1.0 in each grid cell, and each response takes a value from 0 to 1. We weigh each grid cell (observation) in the regression by the sum of cropland and fallow land area fraction in 1985, so that larger area changes are assigned greater weights in our regression. We standardized each explanatory variable (based on statistics computed from the observations) prior to model estimation as described in an earlier sub-section titled "data processing for statistical estimation".

Regularized logistic regression. We represent $'F_{\mathrm{lg}}'$ to be the fractional area (dependent variable) of the two land fractions $(1 \le 'l' \le 2)$ as explained above in grid cell $'g'$. The grid cell constraints can be mathematically expressed as:

$$0 \le F_{\mathrm{lg}} \le 1, \ \sum_{l=1}^{2} F_{\mathrm{lg}} = 1 \tag{1}$$

We assume $'F_{\mathrm{lg}}'$ to be a function of a matrix $'X_g'$ with dimension $'p'$ of explanatory variables (Table S12; specific to simulation). We model the relationship between the dependent and explanatory variables as a "fractional" binomial logistic (FBNL) model proposed by Papke and Wooldridge (Papke and Wooldridge 1996; Papke and Wooldridge 2008). The FBNL regression

allows for fractional outcomes of each dependent variable, thus being able to account for fractional land areas within each 1km grid cell, consistent with our LULCC data spatial aggregation technique. Therefore, FBNL allows for spatial heterogeneity within grid cells.

The logistic regression model (Eqs. 2, 3) represents the class-conditional probabilities through a linear function of the explanatory variables.

$$F_{1g} = \frac{1}{1+e^{-\left(\beta_0 + X_g^T \beta\right)}} \quad (2)$$

$$F_{2g} = \frac{1}{1+e^{\left(\beta_0 + X_g^T \beta\right)}} = 1 - F_{1g} \quad (3)$$

In Eqs. (2, 3), the superscript ^{T} indicates the vector transpose. β_0 is a constant coefficient and β is a vector of coefficients with a component for each explanatory variable. β_0 and β are unknowns that need to be estimated.

Alternatively Eqs. (2, 3) implies that

$$\log\left(\frac{F_{1g}}{F_{2g}}\right) = \beta_0 + X_g^T \beta \quad (4)$$

We fit this model by regularized maximum binomial likelihood. We derive the objective function by maximizing the penalized log-likelihood:

$$\max_{(\beta_0,\beta)\in {}^{p+1}} \left[\frac{1}{N}\sum_{g=1}^{N}\left(F_{1g}\left(\beta_0 + X_g^T \beta\right) - \log\left(1+e^{\left(\beta_0 + X_g^T \beta\right)}\right)\right)\right] - \lambda P_\alpha(\beta) \quad (5)$$

where 'N' is the number of observations in the study region. In Eq. (5), the first term is the log-likelihood part, a concave function of the parameters. The second term $\lambda P_\alpha(\beta)$ is the penalization applied. The penalization is included to prevent over fit due to multicollinearity of the covariates as explained in the next section. $\lambda \geq 0$ is the shrinkage parameter, and P_α is the elastic-net regularization term (40). The penalization term shrinks the coefficients towards 0, relative to the log-likelihood estimates.

Variable selection: Multicollinearity is a common problem in land change modeling where one or more explanatory variables are dependent on each other. High degree of multicollinearity results in high standard errors and spurious coefficient (β_l) estimates. We use elastic-net penalty (Zou and Hastie 2005) to account for multicollinearity. Elastic-net linearly combines lasso (L1) and ridge (L2) penalties respectively. Ridge method capitalizes on the strengths of correlated variables, by shrinking the value of their coefficients towards each other. In other words, when highly correlated predictors are present, ridge has a grouping effect on the variables, thereby avoiding omitted variable bias that is typical of other traditional methods used in land change modeling (e.g. Pearson correlation, forward selection, backward elimination). However, ridge cannot do variable selection, and keeps all predictors in the model. In contrast, lasso can do variable selection where many coefficients are expected to be close to zero, and a small subset to be larger and non-zero. However, the method is extremely variable because in the presence of highly correlated predictors, lasso will randomly pick one and ignore the rest. The elastic-net creates a useful compromise between ridge and lasso; it does variable selection like lasso, but also has a grouping effect as ridge, where strongly correlated explanatory variables are retained in the model.

The elastic-net penalty term in Eq. (5) can be explicitly written as:

$$P_\alpha(\beta) = \left[\frac{(1-\alpha)}{2} \quad \beta \, {}_2^2 + \alpha \quad \beta \, {}_1 \right] \tag{6}$$

In our model, the elastic-net penalty is controlled by mixing parameter α . When $\alpha = 1$, the first term in Eq. (6) becomes zero, and only the second term remains, which is the lasso penalty (L1 indicated by subscript 1). Conversely, when $\alpha = 0$ the second term in vanishes, and only the first term remains, which is the ridge penalty (indicated by subscript 2). A value of α between 0 and 1 is a mix of lasso and ridge penalty. For each simulation, we chose the α parameter using k-fold cross-validation (with k=10), where we randomly partitioned the observations within the study region into 10 equal size subsamples. Of the 10 subsamples, we trained a single subsample as the validation data for testing the model, and we used the remaining 9 subsamples as training data. We repeated the cross-validation process 10 times (the *folds*), with each of the 10 subsamples used exactly once as the validation data. We then averaged (combined) the 10 results from the folds to produce a single estimation. Overall, we fitted a sequence of model for 9 different values of α equally spaced between 0 and 1 (0.1, 0.2..., 0.9), each over a grid of λ values (Fig. S28 for an example). The

variables we selected (variables with non-zero coefficients) for the final estimation corresponds to the 'best model', defined as the model (α and λ parameter) with minimum binomial deviance. See Table S14 for α and λ parameters corresponding to the 'best model' for each simulation.

We use the cylindrical coordinate descent method (Friedman et al. 2007; Friedman at al. 2010) for fitting the elastic-net regularization path for FBNL. The cylindrical coordinate method optimizes each parameter, while keeping other parameters fixed, and repeats the cycling until convergence. The individual grid weights are accounted within coordinate descent takes solves a penalized weighed least-square problem. While the FBNL procedure is based on regularized maximum multinomial likelihood (penalized log-likelihood), the elastic-net is based on penalized least squares. The coordinate descent algorithm fuses FBNL and elastic-net through a three step procedure: 1. simple least squares coefficients are computed on the partial residual, 2. soft-thresholding is applied to account for lasso, and, 3. proportional shrinkage is applied to take care of ridge penalty. Further technical details are spelled out in refs. (Friedman et al. 2007; Van der Kooij 2007). The coordinate descent method takes advantage of sparsity in the data and is extremely efficient for fitting large datasets as used here.

Bootstrap resampling for confidence interval. We use the penalized regression only for variable selection, because the regression coefficients (β_0 and β) estimated by penalization typically has a downward bias. Therefore, for unbiased coefficient estimates, we refit a standard logistic regression with only the variables selected from the elastic-net (i.e. from the 'best model'). For standard logistic regression, we fit Eq. (5), but by setting λ to zero (thus eliminating the penalty term). As we have standardized all explanatory variables prior to model fitting, the fitted β coefficients can be compared to infer the relative importance of different explanatory variables. The standardized β coefficients refer to how many standard deviations a dependent variable will change, per standard deviation increase in the independent variable. We obtained estimates of the mean (in figures: central mark on the box), 5th to 95th percentile confidence interval (ends of whiskers), and 25th to 75th percentile confidence interval (boxes) by bootstrap resampling, where we resampled the observations and we fitted a new model to the data. For each simulation, we used 500 bootstrap samples, so that e.g. the 25th percentile corresponds to the 125th lowest value. The bootstrap serves three purposes. First, the percentile range provides an uncertainty estimate on the

impact of each explanatory variable. Second, the procedure minimizes the effect of any erroneous socioeconomic data that we were unable to identify through the data cleaning process. Third, resampling procedure accounts for spatial autocorrelation that is typical of spatial LULCC datasets. If we disregard spatial autocorrelation in LULCC data, we violate a key statistical assumption that residuals are independent and identically distributed. For brevity, across all simulations we present the 10 variables with largest absolute mean estimates (i.e. 10 most important). The elastic-net parameters and the binomial deviance of the fitted model corresponding to each simulation are presented in Table S14.

Synthesis of case studies. There were two reasons for synthesizing existing ground-based studies on the causes of LULCC in India. First, we used the synthesis to hypothesize the initial set of variables to test over larger spatial regions through our statistical analysis (Table S12). This was important given that we have a large number of socioeconomic variables (N>200), and testing for all of them is resource intensive, considering the high spatial resolution of analysis. Second, from synthesis we could identify common effects (and variations) across studies that are statistically stronger than any individual study due to larger sample size and greater diversity (Fig. 5; Dataset S3). The synthesis therefore provides a second line of evidence (from ground) to complement and evaluate our modeled results.

We reviewed the English language literature for LULCC studies covering India published between 1980 and May 2015. We relied on two scientific indexing services: Web of Science and Google Scholar because many relevant studies were published in national journals that were indexed only in one of the two databases. For initial screening, we searched the literature broadly to include all LULCC processes using the following key word search in Web of Science (similar keywords used for Google Scholar): TI=(Drivers OR determinants OR causes OR dynamics) AND TS=(India AND land*) AND TS=(crop* OR fallow* OR *forest* OR agricul* OR shrub* OR defor* OR wasteland* OR degrade*). The broad literature search for India was meant to get an understanding of the weightage (i.e. number of studies) assigned to studying different LULCC processes, and the type of analysis involved (e.g. land change quantification only, or includes analysis of spatial determinants, and methods of data collection). Overall, the literature review resulted in more than

630 articles that we studied in detail. In order to be included in our analysis, the study had to meet the following four criteria:

1. Study must have dealt LULCC processes that are a focus of our study i.e. conversions between cropland and fallow land, or forest area conversions (both gains and losses). The study region should be within India.
2. Study must discuss the causes of LULCC based on field data (e.g. household surveys, field transects), and/or local/regional expertise of the authors.
3. Study must have covered at least a part of our 20-year study period (1985-2005).
4. Study must not repeat the results presented in another paper.

Among the 630+ articles, we filtered those that met criteria 1, which resulted in 453 articles. In other words, about 72% of all articles focused on LULCC processes that are a focus of our study, highlighting their importance in Indian context. We further narrowed the set of articles to those that meet criteria 2, which yielded 103 articles. In exception, we retained about five studies (counted in 103 studies) that did not meet criteria 2 as they yielded significant insight through modeling, but supported through ground evidence from other published studies. Stage 2 elimination indicates that over three-fourth of the articles focused only on quantifying the magnitude of change (typically from remote-sensing at sub-national scale), indicating less attention has been given to identifying their causes. Applying criteria 3 and 4 resulted in 98 articles. In addition to this set of peer-reviewed articles, we reviewed and included five reports (PhD thesis; reports from government or external agencies such as World Wildlife Fund) that were similar in method and scientific rigor, and were not indexed in either literature database. In total, our synthesis includes 102 studies covering 64 journals.

We have summarized all the 102 studies in Tables S8-S11. See Fig. S3 for a visualization of the study locations. The number of studies by LULCC processes was: conversions between cropland and fallow land (N=37), forest area loss (N=42), and forest area gain (N=23). We included studies that examined the causes of failure to effectively implement forest protection mechanisms (thus causing continued forest loss) under "forest area gain". The studies vary in sample size, spatial extent and location, time period, and method of data collection and interpretation. Following earlier

land change synthesis studies (e.g. van Vliet et al. 2016; Magliocca et al. 2015; Geist and Lambin 2002; van Asselen et al. 2013), we analyzed the frequency of causes across studies (*meta-study*). We grouped the studies by LULCC processes and into broad clusters of causes (see Dataset S3 for study-wise grouping details); the clusters being specific to LULCC process. The results from frequency analysis are shown in Fig. 5.

Text S2. Forest transition in India

According to Forestry Survey of India (FSI), India experienced forest transition from net-deforestation in 1985-1995 (-8600 km^2) to net-reforestation in 1995-2005 (~57500 km^2) (see FSI 2013) and earlier reports cited therein). In contrast, our analysis shows that India experienced a net-deforestation of over 18000 km^2 during both decades (Fig. 1). The discrepancy in the sign and magnitude of change in forest area between the two studies is attributable to the difference in definition of 'forest cover'. Unlike the IGBP definition we followed (see Table S7), the FSI counts all the land with more than one hectare with a tree canopy density of more than 10% as the 'forest cover' which encompass many non-forest (land use) tree categories such as commercial plantations, orchids, tea and coffee gardens. In particular, the FSI definition of 'forest cover' has been questioned for including the green patch of 'open area' (trees outside demarcated forest areas, where most of the increase took place) because green cover accounting cannot be generalized by assessing a small patch of land, in particular 'open forest' area as 'forest cover' (Gilbert 2012; Ravindranath et al. 2014; Puyravaud et al. 2010; Pandit et al. 2007). Our study underscores the need to have a consistent definition of forest across countries, especially if carbon credits are attached to help protect tropical forests (Agrawal et al. 2011; Ravindranath et al. 2012).

References

Agarwal C, Green GM, Grove JM, Evans TP, Schweik CM (2002) A Review and Assessment of Land Use Change Models: Dynamics of Space, Time, and HumanChoice. US Department of Agriculture, Forest Service, Northeastern Research Station, Newton Square, PA, pp. 12–27.

Aggarwal PK (2008) Global climate change and Indian agriculture: impacts, adaptation and mitigation. Indian J Agr Sci. 78: 911.

Agrawal A, Nepstad D, Chhatre A (2011) Reducing emissions from deforestation and forest degradation. Annu. Rev. Environ Res. 36:373-396. doi: 10.1146/annurev-environ-042009-094508

Alagh YK (1996) Next Stage of Agro-Climatic Planning. In Agro-climatic Regional Planning in India: Concept and applications Concept Publishing Company, New Delhi, India. pp 300-309.

Auffhammer M, Ramanathan V, Vincent JR (2012) Climate change, the monsoon, and rice yield in India. Clim. Change 111:411-424. doi: 10.1007/s10584-011-0208-4

Baraldi AN, Enders CK (2010) An introduction to modern missing data analyses. J Sch Psychol. 48:5-37. doi: http://dx.doi.org/10.1016/j.jsp.2009.10.001

Briassoulis H (2000) Analysis of land use change: theoretical and modeling approaches. In: Loveridge S (Ed.) The Web Book of Regional Science. West Virginia University, Morgantown.

Census of India (2011) Administrative Atlas of India, 2011. Published by the Office of the Registrar General & Census Commissioner, India and Ministry of Home Affairs, Government of India. (Available from: http://censusindia.gov.in).

Friedman J, Hastie T, Höfling H, Tibshirani R (2007) Path wise coordinate optimization. Ann App Stat. 1:302-332.

Friedman J, Hastie T, Tibshirani R (2010) Regularization paths for generalized linear models via coordinate descent. J stat softw 33:1.

FSI (2013) India State of Forest Report. Forestry Survey of India (Ministry of Environment and Forest). Dehradun, India.

Gajbhiye KS, Mandal C (2000) Agro-ecological zones, their soil resource and cropping systems. Status paper, In: Status of Farm Mechanization in India. Indian agricultural statistical institute, New Delhi, pp 1-32.

Geist HJ, Lambin EF (2002) Proximate Causes and Underlying Driving Forces of Tropical Deforestation. BioScience 52:143-150. doi: 10.1641/0006

Gelman A (2008) Scaling regression inputs by dividing by two standard deviations. Stat. Med. 27:2865-2873.

Gilbert N (2012) India's forest area in doubt. Nature 489:14-15.

Guiteras R (2009) The impact of climate change on Indian agriculture. Manuscript, Department of Economics, University of Maryland, College Park, Maryland.

Hijmans RJ, Cameron SE, Parra JL, Jones PG, Jarvis A (2005) Very high resolution interpolated climate surfaces for global land areas. Int. J. Climatol. 25: 1965-1978. doi: 10.1002/joc.1276

Huete A, Didan K, Miura T, Rodriguez EP, Gao X, Ferreira LG (2002) Overview of the radiometric and biophysical performance of the MODIS vegetation indices. Remote Sens. Environ. 83:195-213. doi: http://dx.doi.org/10.1016/S0034-4257(02)00096-2

Irwin EG, Geoghegan J (2001) Theory, data, methods: developing spatiallyexplicit economic models of land use change. Agric. Ecosyst. Environ. 85:7–24. doi:http://dx.doi.org/10.1016/S0167-8809(01)00200-6

Krishna Kumar K, Kumar RK, Ashrit RG, Deshpande NR, Hansen JW (2004) Climate impacts on Indian agriculture. Int. J. Climatol 24: 1375-1393. doi: 10.1002/joc.1081

Kyle P (2011) GCAM 3.0 agriculture and land use: data sources and methods. Richland: Pacific Northwest National Laboratory. Richland, Washington.

Lee H, Hertel T, Rose S, Avetisyan M (2009) Economic Analysis of Land Use in Global Climate Change Policy. In An Integrated Land Use Data Base for CGE Analysis of Climate Policy Options. Abingdon, UK: Routledge Press.

Lesschen JP, Verburg PH, Staal SJ (2005) Statistical Methods for Analysing the Spatial Dimension of Changes in Land Use and Farming Systems. International Livestock Research Institute LUCC Focus 3 office.

Lobell DB, Burke MB, Tebaldi C, Mastrandrea MD, Falcon WP, Naylor R (2008) Prioritizing climate change adaptation needs for food security in 2030. Science 319:607-610. doi: 10.1126/science.1152339

Lobell DB, Schlenker W, Costa-Roberts J (2011) Climate trends and global crop production since 1980. Science 333:616-620. doi: 10.1126/science.1204531

Lobell DB, Sibley A, Ortiz-Monasterio JI (2012).Extreme heat effects on wheat senescence in India. Nat. Clim. Change 2:186-189. doi:10.1038/nclimate1356

Magliocca NR, Van Vliet J, Brown C, Evans TP, Houet T, Messerli P, Messina JP, Nicholas KA, Ornetsmüller C, Sagebiel J, Schweitzer V, Verburg PH, Yu Q (2015) From meta-studies to modeling: Using synthesis knowledge to build broadly applicable process-based land change models. Environ. Model. Softw 72:10-20. doi: http://dx.doi.org/10.1016/j.envsoft.2015.06.009

Mall RK, Singh R, Gupta A, Srinivasan G, Rathore LS (2006) Impact of climate change on Indian agriculture: a review. Clim Change 78(2-4):445-478. doi: 10.1007/s10584-005-9042-x

Mandal C, Mandal DK, Bhattacharyya T, Sarkar D, Pal DK, Prasad J, Sidhu GS, Nair KM, Sahoo AK, Das TH, Singh RS, Srivastava R, Sen TK, Chatterji S, Chandran P, Ray SK, Patil NG, Obireddy GP, Mahapatra SK, Kumar KSA, Das K, Singh AK, Reza SK, Dutta D, Srinivas

S, Tiwary P, Karthikeyan K, Venugopalan MV, Velmourougane K, Srivastava A, Raychaudhuri M, Kundu DK, Mandal KG, Kar G, Durge SL, Kamble GK, Gaikwad MS, Nimkar AM, Bobade SV, Anantwar SG, Patil S, Gaikwad KM, Sahu VT, Bhondwe H, Dohtre SS, Gharami S, Khapekar SG, Koyal A, Sujatha , Reddy BMN, Sreekumar P, Dutta DP, Gogoi L, Parhad VN, Halder AS, Basu R, Singh R, Jat BL, Oad DL, Ola NR, Wadhai K, Lokhande M, Dongare VT, Hukare A, Bansod N, Kolhe A, Khuspure J, Kuchankar H, Balbuddhe D, Sheikh S, Sunitha BP, Mohanty B, Hazarika D, Majumdar S, Garhwal RS, Sahu A, Mahapatra S, Puspamitra S, Kumar A, Gautam N, Telpande BA, Nimje AM, Likhar C, Thakre S (2014) Revisiting agro-ecological sub-regions of India-a case study of two major food production zones. Curr. Sci (00113891) 107.

Meiyappan P, Dalton M, O'Neill B, Jain AK (2014) Spatial modeling of agricultural land use change at global scale. Ecol. Model. 291:152-174. DOI: 10.1016/j.ecolmodel.2014.07.027. doi:http://dx.doi.org/10.1016/j.ecolmodel.2014.07.027

Mondal P, Jain M, DeFries RS, Galford GL, Small C (2015) Sensitivity of crop cover to climate variability: Insights from two Indian agro-ecoregions. J. Environ. Manage. 148:21-30. doi: http://dx.doi.org/10.1016/j.jenvman.2014.02.026

Mondal P, Jain M, Robertson AW, Galford GL, Small C, DeFries RS (2014) Winter crop sensitivity to inter-annual climate variability in central India. Clim Change 126:61-76. doi: 10.1007/s10584-014-1216-y

NRC 2014 Advancing Land Change Modeling: Opportunities and Research Requirements. National Academy Press, Washington, DC.

O'Brien K, Leichenko R, Kelkar U, Venema H, Aandahl G, Tompkins H, Javed A, Bhadwal H, Barg S, Nygaard L, West J (2004) Mapping vulnerability to multiple stressors: climate change and globalization in India. Glob. Environ. Chang. 14:303-313. doi: http://dx.doi.org/10.1016/j.gloenvcha.2004.01.001

Pandit MK, Sodhi NS, Koh LP, Bhaskar A, Brook BW (2007) Unreported yet massive deforestation driving loss of endemic biodiversity in Indian Himalaya. Biodivers. Conserv 16:153-163. doi: 10.1007/s10531-006-9038-5

Papke LE, Wooldridge JM (1996) Econometric methods for fractional response variables with an application to 401(k) plan participation rates. J Appl Econom. 11: 619-632. doi: 10.1002/(SICI)1099-1255(199611)11:6<619::AID-JAE418>3.0.CO;2-1.

Papke LE, Wooldridge JM (2008) Panel data methods for fractional response variables with an application to test pass rates. J Econom 145:121-133. doi: http://dx.doi.org/10.1016/j.jeconom.2008.05.009

Parker DC, Manson SM, Janssen MA, Hoffmann MJ, Deadman P (2003) Multi-agent systems for the simulation of land use and land-cover change: a review. Ann. Assoc. Am. Geogr. 93:314–337. doi: 10.1111/1467-8306.9302004

Puyravaud JP, Davidar P, Laurance WF (2010) Cryptic loss of India's forests. Conserv. Lett.3: 390–394. doi: 10.1111/j.1755-263X.2010.00141.x

Ravindranath NH, Murthy I K, Priya J, Upgupta S, Mehra S, Nalin S (2014) Forest area estimation and reporting: implications for conservation, management and REDD. Curr Sci 106:1201.

Ravindranath NH, Srivastava N, Murthy IK, Malaviya S, Munsi M, Sharma N (2012) Deforestation and forest degradation in India- implications for REDD+. Curr Sci 102:1117-1125.

Sohl TL, Sleeter BM, Sayler KL, Bouchard MA, Reker RR, Bennett SL, Sleeter RR, Kanengieter RL, Zhu Z (2012) Spatially explicit land-use and land-cover scenarios for the Great Plains of the United States. Agric. Ecosyst. Environ. 153: 1-15. doi:http://dx.doi.org/10.1016/j.agee.2012.02.019

Schlenker W Roberts MJ (2009) Nonlinear temperature effects indicate severe damages to US crop yields under climate change. PNAS 106: 15594-15598. doi: 10.1073/pnas.0906865106

Schlenker W, Roberts MJ (2006) Nonlinear effects of weather on corn yields. AEPP 28:391-398. doi: 10.1111/j.1467-9353.2006.00304.x

Schlenker W, Roberts MJ (2008) Estimating the impact of climate change on crop yields: The importance of nonlinear temperature effects (No. w13799). National Bureau of Economic Research.

Serneels S, Lambin EF (2001) Proximate causes of land-use change in Narok District, Kenya: a spatial statistical model. Agric. Ecosyst. Environ. 85: 65-81. doi:http://dx.doi.org/10.1016/S0167-8809(01)00188-8

Singh RB, Kumar P, Woodhead T (2002) Smallholder Farmers in India: Food Security and Agricultural Policy. FAO Regional Office for Asia and the Pacific, Bangkok, Thailand.

Sohl TL, Sleeter BM, Sayler KL, Bouchard MA, Reker RR, Bennett SL, Sleeter RR, Kanengieter

van Asselen S, Verburg PH, Vermaat JE, Janse JH (2013) Drivers of wetland conversion: A global meta-analysis. PLoS ONE. doi:http://dx.doi.org/10.1371/journal.pone.0081292.

Van der Kooij A (2007) Prediction accuracy and stability of regresssion with optimal scaling transformations. Technical report, Dept. Data Theory, Leiden Univ.

van Vliet J, Magliocca NR, Büchner B, Cook E, Benayas JMR, Ellis EC, Heinimann A, Keys E, Lee TM, Liu J, Mertz O, Meyfroidt P, Moritz M, Poeplau C, Robinson BE, Seppelt R, Seto KC, Verburg PH (2016) Meta-studies in land use science: Current coverage and prospects. Ambio 45(1): 15-28. doi: 10.1007/s13280-015-0699-8

Velayutham M, Mandal DK, Mandal C and Sehgal J (1999). Agro-ecological Sub-regions of India for Planning and Development. NBBS (National Bureau of Soil Survey and Land Use Planning) Publication 35, Nagpur, India.

Verburg PH, Schot PP, Dijst MJ, Veldkamp A (2004) Land use change modelling: current practice and research priorities. Geo J 61:309–324. doi:10.1007/s10708-004-4946-y

Verburg PH, Schulp CJE, Witte N, Veldkamp A (2006) Downscaling of land use change scenarios to assess the dynamics of European landscapes. Agric. Ecosyst. Environ 114:39-56. doi: http://dx.doi.org/10.1016/j.agee.2005.11.024

Zou H, Hastie T (2005) Regularization and variable selection via the elastic net. J R Stat Soc Series B Stat Methodol. 67:301-320. doi: 10.1111/j.1467-9868.2005.00503.x
