# Supplementary material for: Dynamics and determinants of land change in India: integrating satellite data with village socioeconomics
Source: Reg Environ Change. 2016 Oct 27;17(3):753–66. doi: 10.1007/s10113-016-1068-2 (PMC7064035; doi:10.1007/s10113-016-1068-2)
Supplement: Supplementary file 7 — Supplementary material 7 (PDF 852 kb) [file 10113_2016_1068_MOESM7_ESM.pdf]

Supplementary Tables

Table S1. Extension to Table 1.

**Environmental Problem**	**Remarks**
Human land use	Estimated as the sum of four categories from the data of Ellis et al. 2010. The four categories are: 1. Dense settlements, 2. Villages, 3. Croplands and 4. Rangelands. The data corresponds to year 2000.
Climate change	Estimates averaged over the period 2001-2010.<br><br>Greenhouse gas (GHG) emissions from LULUC for India and World were based on FAOSTAT database (FAO 2013). The estimates include emissions from agricultural activities, net forest conversion, biomass burning, peat fires, cultivation of histosols, and histosols under grassland. They do not include forest sinks.<br><br>Total anthropogenic GHG emissions for India and world (totals needed to compute %) for 2001-2010 were based on EDGARv4.2FT2012 (2014).
Biodiversity loss	Net biodiversity change before year 2005 as estimated by Newbold et al. 2015
Land degradation	Range for global estimates as reviewed by Gibbs and Salmon (2015). We excluded two conservative estimates that were limited to cropland degradation only (both estimates lower than the lower bound presented in table 1). For India, we relied on the official estimate reported by the Government of India (Government of India 2014). While differences in the definition of land degradation may exist, the comparison indicates that the land degradation estimated for India is greater than the upper bound estimate for the global case.<br>As per the national report, the major causes of land degradation in India include: loss of vegetation due to deforestation, cutting beyond permissible limits, unsustainable fuel wood and fodder extraction, shifting cultivation, overgrazing, encroachment into forest lands, forest fires, overgrazing, inadequate soil conservation measures, improper crop rotation, indiscriminate use of agro-chemicals, improper management of irrigation systems and excessive extraction of ground water, urbanization, poverty, inequitable sharing of resources.
Water use for agriculture	Circa (2010); Based on FAO (2015) for both India and the world; Numbers indicate the % of total freshwater withdrawal that was used for agriculture.
Nutrient excess in crops (Water pollution)	Circa (2000); Data from West et al. (2014); The numbers indicate the applied nutrient that is in excess i.e. not harvested in the plant. The numbers are based on analysis of 17 major crops that account for 73% of nitrogen and 68% of phosphorous applied globally.

EDGARv4.2FT2012 (2014) European Commission, Joint Research Centre (JRC)/PBL Netherlands Environmental Assessment Agency. Emission Database for Global Atmospheric Research (EDGAR), release version 4.2. http://edgar.jrc.ec.europe.eu, 2014.

Ellis EC., Klein Goldewijk K, Siebert S, Lightman D, Ramankutty N (2010) Anthropogenic transformation of the biomes, 1700 to 2000. Glob. Ecol. Biogeogr 19:589-606.

FAO (2013) FAOSTAT Emissions Database http://faostat.fao.org/

FAO (2015) AQUASTAT database, Food and Agriculture Organization of the United Nations (FAO). Website accessed on [16/07/2015 20:19]

Gibbs HK, Salmon JM (2015) Mapping the world's degraded lands. Appl. Geogr 57:12-21. doi: http://dx.doi.org/10.1016/j.apgeog.2014.11.024

Government of India (2014) Elucidation of the Fifth National Report submitted to United Nations Convention to Combat Desertification (UNCCD). Ministry of Environment and Forests, Government of India. Report available from http://www.moef.gov.in/

Newbold T, Hudson LN, Hill SL, Contu S, Lysenko I, Senior RA, Börger L, Bennett DJ, Choimes A, Collen B, Day J, De Palma A, Díaz S, Echeverria-Londoño S, Edgar MJ, Feldman A, Garon M, Harrison ML, Alhusseini T, Ingram DJ, Itescu Y, Kattge J, Kemp V, Kirkpatrick L, Kleyer M, Correia DL, Martin CD, Meiri S, Novosolov M, Pan Y, Phillips HR, Purves DW, Robinson A, Simpson J, Tuck SL, Weiher E, White HJ, Ewers RM, Mace GM, Scharlemann JP, Purvis A (2015) Global effects of land use on local terrestrial biodiversity. Nature 520:45-50. doi:10.1038/nature14324

West PC, Gerber JS, Engstrom PM, Mueller ND, Brauman KA, Carlson KM, Cassidy ES, Johnston M, MacDonald GK, Ray DK, Siebert S (2014) Leverage points for improving global food security and the environment. Science. 345: 325-328. doi: 10.1126/science.1246067

Table S2. Gross area losses, gross area gains, and net area changes in land use and land cover areas for 1985-1995 and 1995-2005. All values have been rounded up to nearest integers. For reference, the total geographical area (TGA) of India is 3,287,590 km^2.

Land use and land cover class	1985-1995 (km^2)			1995-2005 (km^2)		
	Losses	Gains	Net Change	Losses	Gains	Net Change
Built-up & Urban	-531	6724	6193	-651	7667	7016
Cropland	-66156	62994	-3162	-69768	131727	61959
Fallow land	-31837	46520	14683	-93712	48958	-44754
Forest	-27169	8905	-18264	-28352	10278	-18074
Plantations	-8788	9247	463	-11131	11767	636
Shrub land	-17682	19733	2051	-24341	26709	2369
Grassland	-5646	4172	-1474	-5833	7676	1793
Barren land	-4082	9848	5766	-8723	7378	-1345
Waste land	-8349	2584	-5765	-8804	4540	-4264
Water bodies[1]	-8802	13831	5029	-18190	11938	-6252
Others[2]	-8978	3462	-5516	-6395	7312	917
**Total**	**-188020**	**188020**	**0**	**-275900**	**275900**	**0**

1 Includes Aqua Culture, Water bodies, and Permanent Wetlands.
2 Includes Salt Pan, Snow and Ice.

Table S3. Land-use and land-cover change (LULCC) transition matrix for 1985-1995 in km^2. Marginal transitions (<1% of total gains and losses) have been termed as not significant (n.s.).

**To 1995 →** / **From 1985 ↓**	Built-up & Urban	Cropland	Fallow land	Forest	Plantations	shrub land	Grassland	Barren land	Waste land	Water bodies[1]	Others[2]
Built-up & Urban		291	26	48	26	78	n.s.	n.s.	36	22	n.s.
Cropland	3811		39551	4065	4336	5221	91	182	712	8187	n.s.
Fallow land	946	25356		319	1861	1708	131	89	135	1291	n.s.
Forest	298	11470	1418		1839	8059	838	1245	251	1102	649
Plantations	585	4728	2530	176		341	n.s.	n.s.	85	327	n.s.
Shrubland	736	9670	1618	2250	887		123	339	534	1341	183
Grassland	n.s.	457	n.s.	638	n.s.	736		1650	n.s.	1013	1079
Barren land	n.s.	231	n.s.	371	n.s.	1079	701	62205	49	89	1458
Waste land	139	5946	563	247	132	830	n.s.	n.s.		438	n.s.
Water bodies[1]	177	4845	714	400	120	1044	813	n.s.	526		n.s.
Others[2]	n.s.	n.s.	n.s.	390	n.s.	636	1460	6216	249	n.s.	

1 Includes Aqua Culture, Water bodies, and Permanent Wetlands.
2 Includes Salt Pan, Snow and Ice.

Table S4. Land-use and land-cover change (LULCC) transition matrix for 1995-2005 in km^2. Marginal transitions (<1% of total gains and losses) have been termed as not significant (n.s.).

To 2005 → / From 1995↓	Built-up & Urban	Cropland	Fallow land	Forest	Plantations	Shrubland	Grassland	Barren land	Waste land	Water bodies[1]	Others[2]
Built-up & Urban		390	74	35	37	57	n.s.	n.s.	14	34	n.s.
Cropland	4394		38392	4669	5776	8192	295	315	1619	6116	n.s.
Fallow land	1308	84521		482	2680	2632	77	199	641	1172	n.s.
Forest	271	10602	1023		1795	9167	961	1305	473	1451	1305
Plantations	812	6776	2588	272		364	n.s.	n.s.	67	234	n.s.
Shrubland	512	11285	3571	3264	913		959	1552	774	1088	422
Grassland	n.s.	908	n.s.	79	n.s.	543		1375	n.s.	928	1817
Barren land	n.s.	543	n.s.	109	n.s.	1545	2190		225	n.s.	3723
Waste land	164	3828	1928	189	99	1862	n.s.	n.s.		710	n.s.
Water bodies[1]	145	12874	1075	594	335	1563	846	n.s.	674		n.s.
Others[2]	n.s.	n.s.	n.s.	584	n.s.	783	2230	2481	n.s.	n.s.	

1 Includes Aqua Culture, Water bodies, and Permanent Wetlands.
2 Includes Salt Pan, Snow and Ice.

Table S5. State-wise summary of key land-cover conversions based on our decadal analysis of Landsat data (the state-wise results pertain to the sum of urban, peri-urban, and rural areas). See Dataset S1 for supporting analysis. The state boundaries are based on 2001 census. In this table, the term "forest degradation" refers to "forest to shrub land conversion".

STATE	KEY CHANGES
**NORTH INDIA<br>Summary: Alarming loss in forest area: loss of 3.4% of region's forest area in 1985-1995, to 4.7% in 1995-2005. Region with maximum conversion of cropland to built-up areas in both decades.**	
**CHANDIGARH**	Planned city with no expansion in the interior areas. Increase in built-up area at the expense of cropland in the periphery due to new planned residential estate (Panchkula Urban Estate) and increasing urban sprawl due to nearness of Chandigarh city and good network of roads.
**DELHI (capital region of India)**	Increasing rates of conversion of cropland to built-up.
**HARYANA**	Increasing rates of conversion of cropland to built-up. During 1995-2005 decade, significant amount of fallow land was brought under cultivation, and water bodies were lost to expansion of cropland and forest.
**HIMACHAL PRADESH**	Overall net loss in forest cover during both decades, but the rates of forest loss has reduced with time. Significant conversions among three categories: others (snow cover), grassland and barren land.
**JAMMU & KASHMIR**	Significant interactions between barren land and grassland/snow cover. The loss in forest cover has more than doubled from 1985-1995 to 1995-2005 resulting in more barren land, snow cover, shrub land, & grassland due to degradation and clear felling.
**PUNJAB**	State with highest cropland to built-up conversion in 1995-2005.
**RAJASTHAN**	The net increase in cropland area nearly tripled from 1985-1995 to 1995-2005 due to recovery of fallow land, wasteland, and shrub land. Increasing rates of forest degradation (Forest → Shrubland) and cropland degradation/abandonment (Cropland → Shrubland). State with second highest area of forest recovery between 1985 and 2005. State with maximum recovery of wasteland area in both decades.
**UTTAR PRADESH**	State with second highest decrease in surface water spread between 1985 and 2005 due to cropland expansion. Significant conversions between cropland and fallow land. Overall, cropland, fallow land, and water bodies area has decreased whereas built-up and shrub land area has increased.
**UTTARAKHAND**	Transition from net decrease to net increase in snow cover between 1985-1995 and 1995-2005, and vice-versa for barren land, shrub land, and grassland.
**WEST INDIA<br>Summary: Region with high dynamism in conversions between cropland and fallow land in both decades**	
**DADRA & NAGAR HAVELI**	No major/significant change.
**DAMAN & DIU**	No major/significant change.

**GOA**	Loss of forest to shrub land (degradation) and cropland. Increase in built-up area.
**GUJARAT**	Huge areas of cropland were converted fallow in 1985-1995, and vice-versa in 1995-2005. Increasing loss in forest cover to cropland (some of which was subsequently converted fallow). Increasing reclamation of wasteland mainly to cropland, shrub and, forests, and water bodies. Decrease in expansion of built-up areas with time.
**MAHARASHTRA**	Increasing loss of surface water spread, with increasing proportions from to cropland expansion (90% loss in water bodies is for cropland in 1995-2005). Increase in built-up area at the expense of cropland. Consistent recovery of fallows to cropland. Decrease in forest and shrub land area, for expansion of cropland and built-up area. Some forests are converted to shrub land, and subsequently to cropland.
**SOUTH INDIA**<br>**Summary: Region with most dynamism in transition between cropland and plantations/fallow land/water bodies.**	
**ANDHRA PRADESH**	State with most decrease in surface water spread between 1985 and 2005. The loss of water bodies more than doubled between the two decades, with increasing proportions converted to cropland (~85% in 1995-2005). Also, the state with highest cropland to water body conversion (24-27% of national total). Transition from net decrease to net increase in cropland area between 1985-1995 and 1995-2005. Significant conversion between cropland and fallow land, and cropland and water bodies during both decades. Net decrease in forest cover by ~780 km^2 during both decades, due to conversion to cropland and shrub land (degradation). Increasing rate of conversion of cropland and fallow land to built-up area. Net decrease in shrub land during both the decades for conversions to cropland, plantations, forest, and built-up areas.
**KARNATAKA**	Net increase in built-up, fallow land, plantation and water bodies and corresponding decrease in cropland, forest, and shrub land. Plantations show accelerated increase due to conversion of cropland, fallow land, and forests.
**KERALA**	Net increase in built-up and plantations area, and net loss of forest area. Plantations are the main source of built-up land, and forests and cropland are the main sources of plantations.
**PUDUCHERRY**	No major/significant change.
**TAMILNADU**	State with maximum dynamism among cropland, fallow land, and plantations during both decades. Increasing rates of conversion of plantations, fallow land, shrub land, and wasteland to cropland. Net increase in built-up area sourced mainly from cropland. Increased rates of conversion of forest to plantations. Overall, net increase in cropland area and net decrease in fallow land, wasteland, shrub land, plantations, and forests.
**EAST INDIA**<br>**Summary: Region with highest loss in forest area: loss of 6.5% of region's forest area in 1985-1995, and 5.1% in 1995-2005. Highest rates of forest degradation in both decades.**	
**BIHAR**	No major land changes during both decades relative to the state's area, indicating lack of development. Land conversions are mainly among cropland, water bodies, fallows, and shrub land. Increasing rates of conversion of cropland to built-up areas.
**JHARKHAND**	Net decrease in cropland, and corresponding increase in fallow land. Net decrease in forest area due to cropland

	expansion.
**ODISHA**	State with highest rate of forest loss in both decades (10.8% and 8.8% of forest cover was lost in decades 1985-1995 and 1995-2005 respectively) due to conversion to shrub land (degradation) and cropland. State with maximum forest degradation in both decades, and maximum deforestation in 1985-1995. The proportion of deforestation for shrub land has increased from 51% to 68% between the two decades. The shrub land are subsequently converted to cropland.
**WEST BENGAL**	No major land changes, relative to state's area. The major conversions are conversion of cropland to water bodies, and vice-versa and reclamation of fallow land to cropland.
**CENTRAL INDIA**<br>**Summary: Region with maximum forest regrowth from cropland abandonment during both decades.**	
**CHHATTISGARH**	Net increase in cropland mainly sourced from fallow, forest, and shrub land. Overall, net decrease in forest, shrub land, and fallow land during both decades.
**MADHYA PRADESH**	State with highest deforested area in 1995-2005, and second highest in 1985-1995 due to conversion to cropland and shrub land (degradation). State with maximum area of forest recovery during both decades. Transition from net decrease to net increase in cropland from 1985-1995 to 1995-2005, and corresponding vice-versa change in fallow land.
**NORTH-EAST INDIA**<br>**Summary: Roughly 80% of forest loss in the region in both decades is for cropland expansion (shifting cultivation) indicating the region is not headed towards settled cultivation.**	
**ARUNACHAL PRADESH**	Deforestation for cropland expansion during both the decades.
**ASSAM**	Increasing rates of deforestation for cropland expansion. Water bodies, grassland and plantations are the other sources of cropland expansion. Net increase in water bodies due to conversion of cropland and grassland. Water bodies interact with all natural and man-mad classes, due to occasional water flooding.
**MANIPUR**	Increasing rates of deforestation for cropland expansion.
**MEGHALAYA**	Deforestation for cropland expansion and forest degradation (forest → shrub land).
**MIZORAM**	Increasing rates of deforestation for cropland expansion.
**NAGALAND**	Increasing rates of deforestation for cropland expansion.
**SIKKIM**	Transition from net decrease in snow cover to net increase in snow cover from 1985-1995 and 1995-2005, and corresponding vice-versa change in barren land.
**TRIPURA**	Increasing loss of forest cover for expansion of cropland and built-up area.

Table S6. Classification of India into twenty Agro-Ecological Zones (AEZs) following National Bureau of Soil Survey and Land Use Planning, India (Gajbhiye and Mandal 2000). See Text S1 for rationale.

Ecosystem type	AEZ	Physiography	Climate	Soils	Growing period
Arid ecosystem	AEZ1	Western Himalayas	Cold arid	Shallow skeletal soil	<90 days
	AEZ2	Western Plain, Kachchh and part of Kathiawar Peninsula	Hot arid	Desert and saline soil	<90 days
	AEZ3	Karnataka Plateau (Rayalseema as inclusion)	Hot arid	Red and black soils	<90 days
Semiarid ecosystem	AEZ4	Northern Plain and Central Highlands including Aravallis	Hot semi-arid	Alluvium-derived soils	90-150 days
	AEZ5	Central Highlands (Malwa), Gujarat plain and Kathiawar Peninsula	Hot semi-arid	Medium and deep black soils	90-150 days
	AEZ6	Deccan Plateau	Hot semi-arid	Shallow and medium black soils	90-150 days
	AEZ7	Deccan plateau (Telangana) and Eastern Ghats	Hot semi-arid	Red and black soils	90-150 days
	AEZ8	Eastern Ghats and Tamil Nadu Uplands and Deccan (Karnataka) Plateau	Hot semi-arid	Red loamy soils	90-150 days
Subhumid ecosystem	AEZ9	Northern Plain	Hot subhumid (dry)	Alluvium-derived soils	150-180 days
	AEZ10	Central Highlands (Malwa and Bundelkhand)	Hot subhumid	Red and black soils	150-210 days
	AEZ11	Moderately to gently sloping Chattisgarh/Mahanadi Basin	Hot subhumid	Red and yellow soils	150-180 days
	AEZ12	Eastern Plateau (Chhotanagpur) and Eastern Ghats	Hot subhumid	Red and lateritic soils	150-210 days
	AEZ13	Eastern Plain	Hot subhumid (moist)	Alluvium-derived soils	180-210 days

	AEZ14	Western Himalaya	Warm subhumid to humid (with inclusion of perhumid)	Brown forest and Podzolic soils	180-210+ days
Humid-Perhumid ecosystem	AEZ15	Assam and Bengal Plain	Hot subhumid (moist) to humid (inclusion of perhumid)	Alluvium-derived soils	210+ days
	AEZ16	Eastern Himalayas	Warm perhumid	Brown and red hill soils	270+ days
	AEZ17	North-Eastern Hills (Purvachal)	Warm perhumid	Red and lateritic soils	270+ days
Coastal ecosystem	AEZ18	Eastern Coastal Plain	Hot subhumid to semi-arid	Coastal and Deltaic alluvium-derived soils	90-210+ days
	AEZ19	Western Ghats and Coastal Plain	Hot humid-perhumid	Red, lateritic and coastal alluvium-derived soils	210+ days
Island ecosystem	AEZ20	Islands of Andaman-Nicobar and Lakshadweep	Hot humid to perhumid island	Red loamy and sandy soils	240+ days

Gajbhiye KS, Mandal C (2000) Agro-ecological zones, their soil resource and cropping systems. Status paper, In: Status of Farm Mechanization in India. Indian agricultural statistical institute, New Delhi, pp 1-32.

Table S7. Definition of the 11 land-use and land cover classes used in our study, consistent with the IGBP land classification scheme (Belward 1996).

No.	Land-use/cover classes	Definition
1	Cropland	Temporarily cropped area followed by harvest and a bare soil period (e.g. single and multiple cropping systems). Note that perennial woody crops will be classified as either forest or shrubland, whichever is appropriate. Includes orchards. We do not differentiate between different types of cropland based on seasons (e.g. kharif, rabi, zaid).
2	Fallow land	Land taken up for cultivation, but are temporarily allowed to rest, un-cropped for one or more seasons. We do not differentiate between different seasonal cropland types e.g. kharif, rabi, zaid) being fallowed.
3	Forest	Land with woody vegetation with greater than 60% cover and height exceeding 2 m. Also includes savannas (both woody and non-woody) defined as herbaceous and other understory systems, with forest canopy cover of 10-60%, and height exceeding 2m.<br><br>"Forest" here was obtained by combining six forest classes from IGBP Level II classification: Deciduous broadleaf forest, Deciduous needle leaf forest, Evergreen broadleaf forest, Evergreen needle leaf forest, mixed forests, and savannas (woody + non-woody).<br><br>Note that IGBP definition of forest is different from that of Forestry Survey of India (FSI 2015. FSI defines forest cover as all lands more than 1 ha in area, with a tree canopy density of more than 10% as forest, irrespective of ownership and legal status. FSI reported forest area includes areas of trees outside forest (forest plantation, and agriculture plantations). In our study, forest plantations are a separate category ("Plantations" category), and agricultural plantations are included within "Cropland" category.
4	Shrubland (open & closed)	Land with woody vegetation less than 2 m in height and with greater than 10% shrub canopy cover. The shrub foliage can be either evergreen or deciduous.
5	Plantations	Commercial horticulture plantations and tree cash crops.
6	Water bodies	Areas with surface water, either impounded in the form of ponds, lakes, reservoirs, aquaculture, permanent wetlands or flowing as streams, rivers, etc. Permanent wetland is defined as a land with a permanent mosaic of water and herbaceous or woody vegetation. The vegetation can be present in either salt, brackish, or fresh water.
7	Built-up &Urban	Land covered by buildings and other man-made structures.

8	Wasteland	Sparsely vegetated land with signs of erosion and land deformation that could be attributed to lack of appropriate water and soil management, or natural causes. These are land identified as currently underutilized and could be reclaimed to productive uses with reasonable effort. Degraded forest (<10% tree cover) with signs of erosion is classified under wasteland.
9	Grassland	Land with herbaceous land cover. Tree and shrub cover is less than 10%.
10	Barren land	Exposed soil, sand, or rocks and has less than 10% vegetation cover throughout the year.
11	Others	Includes land user snow/ice cover for most of the year. Also includes Salt Pan (land covered with salt and minerals).

Belward AS (1996) The IGBP-DIS Global 1km Land Cover Data Set 'DISCOVER': proposal and implementation plans. Report WP No. 13, IGBP-DIS, Stockholm, Sweden.

FSI (2015) India State of Forest Report. Forestry Survey of India (Ministry of Environment and Forest). Dehradun, India.

Table S8. Summary of studies (N=21) on conversion of cropland to fallow land. Keywords in the third column are highlighted in bold for skimming.

Study #	LULCC	Key results	Study details	Methods	Ref
A1	Cropland → Fallow → Grassland	• **Out-migration** to urban areas for better jobs<br>• **Labor shortage**<br>• **Water scarcity**	**Location**: 11 villages in South India covering three states (Andhra Pradesh, Karnataka & Tamil Nadu)<br><br>**Study period**: 1981-2006<br><br>**Focus**: Cropland abandonment	Land investigations<br><br>Household survey (sample size unknown)<br><br>Participatory rural appraisal	Tiwari et al. 2010
A2	Cropland → Fallow	• **Inter-annual fluctuations in rainfall**: over half the cultivated areas in Tamil Nadu are rain fed<br>• **Continuous failure** & **delayed onset of monsoon**<br>• Dry & drought-prone regions with mostly tank irrigation (**unstable water source**), & little well irrigation (relatively stable)<br>• **Improvements in irrigation facilities**: leads to diversification to water-intensive cash crops in small areas, converting some lands fallow. Crop selection depends on its price behavior in markets/demands<br>• **Labor scarcity** & **increase in urban wage rates** (fallow conversions in regions with high rainfall): with labor scarcity, wages increase which increases the cost of cultivation keeping the land fallow<br>• **Inadequate capital** & **non-**	**Location**: Six regions in the state of Tamil Nadu, with differing agro-ecological zones, irrigation system & farming practices<br><br>**Study period**: 1960-2000	Survey of 900 farms<br><br>Secondary data on district level land use statistics	Ramasamy et al. 2005; Nadkarni and Deshpande (1979)

		**availability of credit facilities**: especially in tank irrigated areas which is unstable with more rice-based system & have no access to ground water<br>• **Higher non-agricultural income**: shortage of family labor because off-farm jobs are less strenuous, & generate higher & stable income<br>• **Larger size of land holdings**: Credits, labor, & water becomes limiting factor to expansion			
A3	Cropland ↔ Fallow	• **Large size of land holdings**<br>• **Land tenancy**: higher leased-in area/land owned fraction results in less fallow land<br>• **Better irrigational facilities**: focused efforts on small areas, leaving other areas fallow<br>• **Land rental markets**/Formal land tenancy: increases land access & provides stable livelihoods to poor reducing fallows	**Location**: National level<br><br>**Study period**: 1992-2005<br><br>**Note**: The study has no primary data component, but we included in our analysis as the study provides key insights on cropland-fallow land dynamics in India.	Statistical analysis of state level panel data on land utilization from Indian government combined with census statistics	Bardhan and Tewari (2010)
A4	Cropland → Fallow	• **Drought-prone regions**<br>• **High capital requirements for adopting modern outputs** viz. irrigation (especially well irrigation), tractors & commercialization: reason applies to regions with high rainfall; in drought-prone regions technology diffusion is poor.	**Location**: Andhra Pradesh (comparison across districts within the state & by size class)<br><br>**Study period**: 1955-1987	Primary data collected at farm level (sample size & distribution unclear)<br><br>Secondary data on land use statistics	Reddy (1991)
A5	Cropland → Fallow	• **Introduction of irrigation facilities**: leads to diversification to water-intensive cash crops (e.g. cotton,	**Location**: Command area of Tawa irrigation project, Hoshangabad district, Madhya Pradesh	Regional/local expertise	Shrivastva et al. 1991

		oilseeds) in small areas, converting some lands fallow. Crop selection depends on its price behavior in markets/demands	**Study period**: 1970-1980	Secondary data on land use statistics	
A6	Cropland → Fallow/Wasteland/Barren	• **Division of land**<br>• **Higher land holding size**<br>• **Number of fragments of operational holdings**<br>• **Poor irrigational facilities**<br>• **Decrease in family labor** (out-migration for non-farm jobs): less incentive to invest in soil conservation<br>• **Higher dependency on farm income**: More fertilizer inputs leading to more degradation<br>• **Higher education** has two effects:<br>➢ More awareness → less degradation<br>➢ More off-farm jobs → more degradation<br>• Wild animal menace<br>• Weeds infestation	**Location**: Himachal Pradesh<br><br>**Study period**: 1995-2005<br><br>**Focus**: Land degradation in farms	Survey of 200 farm HH in 20 villages<br><br>Secondary data on land use statistics	Gupta and Sharma (2010)
A7	Cropland → Fallow → Grassland	• **Out-migration** to urban areas due to **unemployment** (less demand for labor)<br>• **Low labor wage rates** for farming<br>• **Small land holdings**<br>• **Risk in agriculture**	**Location**: Majhgawan block, Satna district, Madhya Pradesh<br><br>**Survey period**: Circa 2000<br><br>**Note**: See Rajendran (1993) for an associated discussion on labor use in Thanjavur district, Tamil Nadu. See Oberai & Ahmed (1981) for labor use behavior in agriculture based on household survey of 26 villages in Ludhiana district, Punjab.	Survey of 140 HH: half from agriculturally modernized & half from un-modernized villages	Lenka et al. 2002

A8	Cropland → Fallow/Wasteland/Barren	• **Access to stored rainwater** e.g. proximity to pond/micro-level spatial organization of cropland<br>• **Diversification to cash-oriented crop**s<br>• **Inability of small farmers (correlated to caste) to cope with agricultural crisis**, due to inability to compete with big farmers for control of natural & human resources<br>• **More profits & risk free nature of non-agricultural jobs**: need for money to sustain HH<br>• **Labor shortage**: out-migration for better jobs in urban areas (rural employment diversification)<br>• **Small size of land holdings** make mechanization uneconomical (lack of labor & oxen)<br>• **Lack of medical facilities** (high oxen mortality)<br>• **Weak institutional arrangements**: feudal culture & faulty land reforms<br>• **Droughts**<br>• Road constructions created **new rural jobs** (e.g. dairy industry & fisheries)	**Location**: Magadha area, Gaya district of South Bihar.<br><br>**Study period**: 1960-present<br><br>**Focus**: Case of marginalization of agricultural land by forced tenancy when off-farm jobs become more profitable & risk-free than earnings from small farms	Data collected through participation-observation<br><br>Regional/local expertise	Singh S (2013)

A9	Cropland → Fallow/Wasteland/Barren	• **Droughts**, **water scarcity**, & **soil degradation**<br>• Low economic returns to investments: related to **soil quality/productivity** & **structure of markets**<br>• Other variables that determine conversions:<br>➢ **Access to credit**, **labor scarcity**, **scarcity of land**, **caste**, **social capital** & distance from home (supervision problem, high transaction cost)<br>• **Imperfect labor markets**: male workforce are better in resource conservation management<br>• **Size of farm**:<br>➢ Large land holdings → less incentive to invest<br>➢ Scarce land (land/person) → more investment<br>• **More off-farm income** → less incentive for investment in soil-water conservation, seeds & irrigation<br>• Availability of water for irrigation in post-rainy season (rabi crops)<br>• High rainfall in rainy/kharif seasons (difficult working conditions)<br>• **History of cropping & fallowing**: Fewer fallows where legumes (nitrogen-fixing) were grown in previous year (crop rotation system)	**Location**: Six villages in Rangareddy district, Andhra Pradesh<br><br>**Survey period**: 2001-2002<br><br>**Focus**: Causes of agricultural land degradation & conservation through natural conservation management<br><br>**Context**: The six villages are semi-arid regions prone to droughts, water scarcity & soil degradation. One village is under community water shed management program, & other five adjoining villages are not.	Survey of 60 HH within each village<br><br>Detailed plot- & crop-wise input & output data collected from all operational holdings of surveyed HH (n=568)	Shiferaw et al. 2006a; Shiferaw et al. 2006b
A10	Cropland → Fallow/Wasteland/Barren	• New income opportunities: in booming tourism sector, government, & army<br>• Brick production for houses in	**Location**: Upper Indus Basin of Central Ladakh (high altitude desert region), Northern India (Trans-Himalayan environment)	Remote sensing of land cover<br><br>Qualitative	Nüsser (2012)

		fallows<br>• Labor scarcity<br>• Increased monetary income	**Study period**: 1969-2006<br><br>**Survey period**: 2007-2009<br><br>**Focus**: Case of subsistence-based agriculture, where primary income is from non-farm sector.<br><br>The authors present two local case studies at the village level (Stok & Indus valley) & an overview of the complete Central Ladakh Basin, Leh.	interviews with experts from both government & non-governmental organization<br><br>Regional/local expertise	
A11	Cropland → Fallow → Grassland	• **Labor scarcity** due to male out-migration: most are landless or small landholders who work as wage laborers during cropping season<br>• **Higher education** among family labor: young literates prefer off-farm jobs<br>• **Imperfect labor markets**: Women, elderly, & children take care of farming & their decisions about resource-use efficiency is poor compared to non-migrant farmers<br>• **Small land holdings** per capita<br>• **Less family income** other than remittances<br>• **Proportion of lower caste**: proxy for available input capital & land availability<br>• **Lack of critical support services →** drives migration	**Location**: Kathihar & Samastipur district, Bihar; Varanasi & Azamgarh district, Uttar Pradesh<br><br>**Study period**: 1990s<br><br>**Focus**: Migration induced agriculture land abandonment. Both the states account for ~40% of total out-migrants in India from rural places (especially male), & the rate of out-migration is increasing rapidly. Out-migration is mainly from Bihar to Delhi & Punjab; Uttar Pradesh to Maharashtra & Delhi.	Structured interviews of randomly selected 200 out-migrant families & 200 non-migrant families with land holdings less than 2 ha from each state<br><br>Secondary data from National Sample Survey & from statistical abstract published by the Government of India	Singh et al. 2011
A12	Cropland → Fallow/Wast	• **Higher land-man ratio**: more degradation where agricultural	**Location**: National extent; Study broken down at regional level (by	Expert knowledge on the dynamics	Reddy (2003);

	eland/Barren	pressure is less i.e. low population agriculturally backward regions are more prone to degradation than developed intensively practiced regions<br>• **Size of land holdings**: Small land areas are put to more intense use, than bigger land holdings where some land is irrigated & rest is allowed to remain fallow<br>• **Irrigation increases** salt affected & water logged **degradation when managed poorly**<br>• Poverty, population, institutional credits, & rainfall (hypothesized natural factor) not sufficiently related to degradation	agro-ecological zones, districts, & states)<br><br>**Study period**: Various (1980s)<br><br>**Focus**: Farmland degradation	on soil degradation in India<br><br>Statistical modeling combining remote sensing of land cover with socio-economic, demographic, technological, institutional & biophysical factors	Yadav (1996)
A13	Cropland → Fallow/Barren/Wasteland	• **Distress out-migration**: positive feedback as leads to sub-optimal land use & further degradation of land due to shortage of labor or able bodied persons of the HH<br>• **Lack of irrigation**: perception that people think if land is irrigated it is not degraded, even if the land is saline or eroded<br>• **Risk aversion attitude**: Leasing out lands as risk aversion strategy or using them for short & more remunerative crops	**Location:** Three dryland, drought-prone regions (Surendranagar, Amreli, & Jamnagar) of Surashtra, Gujarat<br><br>**Survey period**: circa 2005<br><br>**Focus**: Migration induced land degradation. In dry regions water, rather than land is the limiting factor.	Survey of 1227 HH in six villages	Shah (2010)
A14	Cropland → Fallow	• **Irrigation facilities**: also a proxy for mechanization, because it is associated with intensive farming<br>• **Lack of access to institutional credit**: larger farms have more access	**Location**: 2 sub-divisions in Burdwan district, West Bengal<br><br>**Survey period**: 2005-2006	Survey of 185 farmers from 5 villages	Ghosh (2010)

		to more agricultural implements as their asset base is stronger<br>• **Size of land holdings**: Uneconomical to use modern costly agricultural implements & machinery<br>• **Lack of support services** through government extension agencies for providing knowledge & information on modern agriculture	**Focus**: Small & marginal farmers who have difficulties exploiting agricultural implements. Farm mechanization is related to higher productivity & incomes.		
A15	Cropland → Bareland	• **Growth of mining industry & thermal power plants**: Extension of mining areas, infrastructure & residential complexes of mining industry due to increased job availability	**Location**: Singaruli district, Madhya Pradesh<br><br>**Study period**: 1978-2010	Regional/local expertise<br><br>Historical literature<br><br>Satellite mapping of land cover	Areendran et al. 2013; Singh et al. 1991; Singh et al. 1997; Khan and Javed (2012)
A16	Cropland → Wasteland/ Barren	• For **production of brick kilns** due to urbanization:<br>➢ Demands for bricks have increased rapidly, its rate stimulated by the centrifugal forces of the city & its influence in the region<br>➢ Higher profits than agriculture: Land owners make quick money from leasing out their land for soil quarrying<br>➢ Requires less labor efforts<br>➢ Brick kiln owners earn higher wages from: employment in brick kilns, transportation of bricks from the quarrying site to the kilns for being baked, & from kilns to the demand site (city)	**Location**: Peripheral areas of Aligarh City, North India<br><br>**Survey period**: 2001-2002<br><br>**Background**: Brick making is mostly confined to rural & peri-urban areas. India is the second largest producer of bricks in the world. The Gangetic plain of North India accounts for about 65% of the total brick production due to availability of good fertile alluvial soils (Punjab, Haryana, Uttar Pradesh, Bihar, & West Bengal); Peninsular & coastal India accounts for rest 35% production (Gujarat, Orissa, Madhya Pradesh, Maharashtra & Tamil Nadu).	20 field survey in each of the 59 surrounding villages (10 village HH & 10 brick kiln workers HH)<br><br>Remote sensing of land cover<br><br>Secondary data from village land use records	Singh and Asgher (2005)
A17	Cropland →	• Cropland → Fallow (1980-90):	**Location**: Sadiyagad micro	Field	Rao and

	Fallow	**Restricting access to government forest** under conservation forestry **provided less manure** (lack of leaf litter) making agricultural land unproductive<br>• Fallow → Cropland (1990-95): **Government intervention on watershed development** (through World Bank) by providing agricultural assistance (irrigation, pesticide & fertilizer)<br>• Cropland → Fallow (1990-): **Lack of appropriate follow-up/monitoring** after the development program ended: aid driven development syndrome where capital accrues immediately after withdrawal of project	watershed, in mid-elevation zone of Central Himalaya<br><br>**Study period**: 1980-1996<br><br>**Focus**: Forest policy effects on cropland	investigations<br><br>Regional/local expertise<br><br>Remote sensing of land cover, topographical maps combined with socioeconomic data collected from conducting series of workshops & interviews with local population	Pant (2001)
A18	Cropland → Wasteland/Barren land	• **Unsustainable land use practices**, different from traditional tribal cultivation (e.g. farming deep-rooted oil crops that caused soil erosion)	**Location**: Two villages in Attappady block, Palakkad district, Kerala<br><br>**Study period**: 1930s-present<br><br>**Survey period**: circa 1995<br><br>**Focus**: Land use & crop selection of aboriginals & in-migrants	Survey of 367 farm HH (6% of total farm HH) in two villages<br><br>Secondary data on settlements & land use patterns	Velluva and Velluva (2006)
A19	Cropland → Barren/Waste land	• **Transforming into suburbs, residential, & commercial land uses** e.g. farm houses, godowns, mills & brick kilns especially in areas:<br>➢ that have good water supply & growing water levels<br>➢ close to urban center which gives much higher returns compared to agriculture	**Location**: Two development blocks in the metropolitan periphery of Delhi (Alipur & Najafgarh)<br><br>**Study period**: 1990s<br><br>**Survey period**: 1998<br><br>**Note**: See ref. 26 for similar study in Delhi based on survey of 896 farming	Interview of 300 persons based on pre-structured questionnaire	Singh and Mohan (2001)

			HH.		
A20	Cropland → Wasteland	• Single factor causation: **Advancement of mining and industrial activity**.	**Location**: Talcher-Angul region, Orissa.<br><br>**Study period**: 1973-2011<br><br>**Focus**: Focused on land degradation.	Remote sensing<br><br>Topographical maps<br><br>Ground validation using Google imageries.	Panwar et al. 2011
A21	Cropland → Fallow/Wasteland/Shrubland	• Inadequate supply & erratic **availability of electricity**: Hindering use of modern agricultural equipment such as cold storage & food processing industries<br>• Lack of **marketing & storage facilities**<br>• Lack or poor quality **agriculture extension facilities**<br>• Poor **diagnostic/medical labs** for both crops & livestock<br>• **Unprofessional attitude of authorities**	**Location**: National scale: Comparison of Eastern & North-Eastern India (has lower diversification that rest of India) with rest of India<br><br>**Survey year**: 2003<br><br>**Focus**: Improving cropland stability through agricultural diversification: enhances profits & stability of farm incomes, generates employment opportunities, alleviate poverty & improve the sustainability of agricultural systems.	Statistical analysis of farm level information from 2003 National Sample Survey, based on information collected from over 178000 HH plots	Kumar and Singh (2012); Kumar (2009)

Areendran G, Rao P, Raj K, Mazumdar S, Puri K (2013) Land use/land cover change dynamics analysis in mining areas of Singrauli district in Madhya Pradesh, India. Trop. Ecol. 54: 239-250.

Bardhan D, Tewari SK (2010) An Investigation into Land Use Dynamics in India & Land Underutilization. Indian J Agric. Econ. 65:658.

Firdaus G, Ahmad A (2011) Impact analysis of urbanization on rural livelihood–an empirical study of an urban centre of Delhi, India. Int. J. Urban Sci. 15: 147-160. doi:http://dx.doi.org/10.1080/12265934.2011.626176

Ghosh BK (2010) Determinants of Farm Mechanization in Modern Agriculture: A Case Study of Burdwan Districts of West Bengal. Int. j. agric. res. 5: 12. doi: 10.3923/ijar.2010.1107.1115

Gupta S, Sharma RK (2010) Dynamics of land utilisation, land degradation & factors determining land degradation in Himachal Pradesh. Indian J Agric. Econ. 65: 245-260.

Khan I, Javed A (2012) Spatio-temporal land cover dynamics in open cast coal mine area of Singrauli, MP, India. J Geogr Inf Syst. 4: 521-529. doi: 10.4236/jgis.2012.46057

Kumar A (2009) Rural employment diversification in Eastern India: Trends & determinants. Agric Econ Res Rev. 22: 47–60.

Kumar C, Singh SP (2012) Determinants of agricultural land use diversification in eastern & northeastern India. J Agric Food Syst Community Dev. 3: 73-98.

Lenka S, Singh KK, Vishwavidyalaya MGG (2002) Trends & causes of migration in agriculturally under modernized vis-a-vis modernized villages. Ann Agric Res. 23:289-296.

Nadkarni MV, Deshpande RS (1979) Underutilization of land: climatic or institutional factors? Indian J Agric. Econ. 34:1-16.

Nüsser M, Schmidt S, Dame J (2012) Irrigation & development in the Upper Indus Basin: characteristics & recent changes of a socio-hydrological system in Central Ladakh, India. Mt Res Dev. 32:51-61. doi: http://dx.doi.org/10.1659/MRD-JOURNAL-D-11-00091.1

Panwar S, Sinha RK, Singh G (2011) Time sequential surface change analysis of Talcher-Angul region of Orissa using Remote Sensing and GIS. Int. J. Geomat. Geosci. 1: 828.

Ramasamy C, Balasubramanian R, Sivakumar SD (2005) Dynamics of Land Use Pattern with Special Reference to Fallow Lands-An Empirical Investigation in Tamil Nadu. Indian J Agric Econ. 60: 629.

Rao KS, Pant R (2001) Land use dynamics & landscape change pattern in a typical micro watershed in the mid elevation zone of central Himalaya, India. Agric Ecosyst Environ. 86: 113-124. doi:http://dx.doi.org/10.1016/S0167-8809(00)00274-7

Reddy VR (1991) Underutilization of land in Andhra Pradesh: Extent & determinants. Indian J Agric. Econ. 46: 555-567.

Reddy VR (2003) Land degradation in India: Extent, costs & determinants. Econ. Polit. Wkly. 4700-4713.

Shah A (2010) Land degradation & migration in a dry land region in India: extent, nature & determinants. Environ Dev Econ. 15:173-196. doi: http://dx.doi.org/10.1017/S1355770X09990131

Shiferaw BA, Reddy VR, Wani SP, Rao GN (2006a) Watershed management & farmer conservation investments in the semi-arid tropics of India: analysis of determinants of resource use decisions & land productivity benefits. J. SAT Agric. Res.2: 1-25.

Shiferaw BA, Reddy VR, Wani SP, Rao GN (2006b) Watershed management & farmer conservation investments in the semi-arid tropics of India: analysis of determinants of resource use decisions & land productivity benefits. Working Paper Series - International Crops Research Institute for the Semi-Arid Tropics, 16 pp 28.

Shrivastva SN, Mishra BL, Baghel AS (1991) Dynamics of land use & cropping pattern in Tawa command area of Hoshangabad District, Madhya Pradesh. Agricultural Situation in India 45:743-748.

Singh AL, Asgher MS (2005) Impact of brick kilns on land use/landcover changes around Aligarh city, India. Habitat International 29: 591-602.

Singh JS, Singh KP, Agrawal M (1991) Environmental degradation of the Obra-Renukoot-Singrauli area, India, & its impact on natural & derived ecosystems. Environmentalist 11:171-180. doi:10.1007/BF01263230

Singh NP, Mukherjee TK, Shrivastava BBP (1997) Monitoring the impact of coal mining & thermal power industry on landuse pattern in & around Singrauli Coalfield using remote sensing data & GIS. J Indian Soc Remote**.** 25: 61-72. doi:10.1007/BF02995419

Singh NP, Singh RP, Kumar R, Padaria RN, Singh A , Varghese N (2011) Determinants & farm - level impacts of labour out migration in the Indo-Gangetic Plains of India. Indian J Agric. Econ. 66: 127-138.

Singh RB, Mohan J (2001) Diversification of Rural Agricultural Land Due to Urban Intrusion-A Case-Study of the Metropolitan Periphery of Delhi, India, Pub. RB Singh, J. Fox & Y. Himiyama, Science Pub., Inc., Enfield (NH), USA, 155-168.

Singh S (2013) Dynamics of agricultural marginalization in emergent rural economy: A study in south Bihar. Int J Rural Management 9: 71-96. doi: 10.1177/0973005213479208

Tiwari R, Murthy IK, Killi J, Kandula K, Bhat PR, Nagarajan R, Kommu V, Rao KK, Ravindranath NH (2010) Land use dynamics in select village ecosystems of southern India: drivers & implications. J Land Use Sci. 5:197-215. doi:http://dx.doi.org/10.1080/1747423X.2010.500683

Velluva S, Velluva S (2006) Land settlement & degradation: dynamics of land use in recently settled forest areas of Kerala. Land settlement & degradation: dynamics of land use in recently settled forest areas of Kerala. Serials Publications, Delhi, India, pp 205.

Yadav JSP (1996) Extent, nature, intensity & causes of land degradation in India. Soil Management in Relation to Land Degradation & Environment. Indian Soc. Soil Sci. Bull 17: 1-26.

Table S9. Summary of studies (N=16) on reclamation of fallow land to cropland. Keywords in the third column are highlighted in bold for skimming.

Study #	LULCC	Key results	Study details	Methods	Ref
B1	Cropland stability (preventing fallow conversion)	• **Adoption of new technology** among tribal community, determined by:<br>➢ **Income from crops**<br>➢ **Credit orientation of farmers**<br>➢ **Attitude towards high yielding varieties**<br>➢ **Risk orientation**<br>➢ **Age of farmers**: young farmers adopt more<br>➢ **Size of land holding**: big farm size/less fragmentation → more adoption indicating easy orientation; small farm size → less adoption indicated by more labor workers	**Location**: Hazaribagh & Ranchi districts, Chotanagpur region of South Bihar<br><br>**Study period**: 1988-89<br><br>**Focus**: Cropland productivity/stability among tribal farms	Survey of 160 tribal farm holdings	Chandra and Singh (1992)
B2	Cropland stability (preventing fallow conversion)	• Social factors: farmers **caste**, availability of **family labor**, **land ownership**, **legumes in cropping sequence**<br>• Infrastructural factors: **Irrigation facilities**, **seed type**, optimal plant population, **labor & capital investment** & use of organic manure	**Location**: Two distinct agro-ecological zones in Eastern India (Bankura & Malda district, West Bengal)<br><br>**Focus**: Focused on smallholder farmers, especially on maize yields	180 farm-level surveys in four villages	Banerjee et al. 2014
B3	Fallow → Cropland	• Availability of **private well irrigation**<br>• **High rainfall & industrially less progressive** regions<br>• **Expansion of irrigation facilities**<br>• Availability to **road facilities** (market access)	**Location**: Six regions in the state of Tamil Nadu, with differing agro-ecological zones, irrigation system & farming practices<br><br>**Study period**: 1960-2000	Survey of 900 farms<br><br>Secondary data on district level land use statistics	Ramasamy et al. 2005; Nadkarni and Deshpande (1979)

B4	Fallow → Cropland	• **Access to weather information** (through television, newspaper, etc.)/ perceived changes in temperature<br>• **Availability of capital**: wealthier farmers more likely to take risks<br>• Participation in social institutions relating to **agriculture/natural resource management** : better adaptation to erratic & variable/delayed monsoon rainfall	**Location**: 7 villages in Bihar<br><br>**Survey period**: Various (1990s & 2000s)<br><br>**Focus**: Changes in land management practices e.g. increased cropping & irrigation.	981 HH surveys	Wood et al. 2014
B5	Fallow/Wasteland/Shrubland → Cropland	• **Level of urbanization/infrastructure**: capital investment capacity of the HH, use of new technology & knowledge, cost advantage (markets & roads) of transportation of high value crops, their quick sales, & increased demand<br>• **Availability of capital**: ability to acquire assets & equipment's necessary to cultivate high value crops & other such allied activities<br>• **Educational level** of HH head (exposure)<br>• Soil quality<br>• **Size of land holdings**<br>• Status of land possession/**land tenure**<br>• Equitable provision of economic security in terms of **credit supply, subsidies**, etc. to all religious & social (caste) classes. e.g. to scheduled caste/tribe farmers for more settled cultivation to augment agricultural diversification	**Location**: National scale: Comparison of Eastern & North-Eastern India (has lower diversification that rest of India) with rest of India<br><br>**Survey year**: 2003<br><br>**Focus**: Improving cropland stability through agricultural diversification: enhances profits & stability of farm incomes, generates employment opportunities, alleviate poverty & improve the sustainability of agricultural systems.	Statistical analysis of farm level information from 2003 National Sample Survey, based on information collected from over 178000 HH plots	Kumar and Singh (2012); Kumar (2009)
B6	Fallow → Cropland	• **Community-based watershed management** through customary governance institutions<br>• **Government program incentives**:	**Location**: Kuchgad micro watershed, Almora District, Central Himalayas	Discussion interviews with officials of management	Wakeel et al. 2005

		Building of roads & vegetable storage facility sustained agriculture for urban exports & access to products outside the region	**Study period**: 1967-1997<br><br>**Survey period**: 1997-2000<br><br>**Note**: See Dhyani et al. (2006) who conducted studies in Khootgad & Mohnagad watershed in Central Himalayas where fodder production expanded to uncultivated rainfed land.	institutions (e.g. forest department, *vanpanchayats*, & local inhabitants in village meeting)<br><br>Regional/local expertise<br><br>Interpretation of satellite data, information on legal & policy changes	
B7	Fallow/Wasteland/Barren → Cropland	• **Development interventions** by government: New structures to prevent flash flooding, sedimentation & to divert water to higher parts<br>• **External influences**: Non-governmental organization support in watershed management programs, resource management efficiency, expansion & improvement of irrigation infrastructure, & agrarian innovations	**Location**: Upper Indus Basin of Central Ladakh (high altitude desert region), Northern India (Trans-Himalayan environment)<br><br>**Study period**: 1969-2006<br><br>**Survey period**: 2007-2009<br><br>**Focus**: Change in irrigated agriculture in mountainous environment with artificial irrigation fed by melt water from glaciers & snow cover, controlled entirely by gravity.<br><br>The authors present two local case studies at the village level (Stok & Indus valley) & an overview of the complete	Remote sensing of land cover<br><br>Qualitative interviews with experts from both government & non-governmental organization<br><br>Regional/local expertise	Nüsser et al. 2012

			Central Ladakh Basin, Leh.		
B8	Fallow/Wasteland → Cropland	• Investment in **irrigation, wells & agricultural development**: Resulted in spread of dry-season cropping & year-around monoculture due to faster rotations under irrigation	**Location**: Godwar, Rajasthan<br><br>**Study period**: 1986-1999	Remote sensing of land cover; Historical data; HH production information; Discourse of planners & state experts	Robbins (2001)
B9	Fallow → Cropland	• During 1990-95: **Government intervention on watershed development** (through World Bank) by providing agricultural assistance (irrigation, pesticide & fertilizer)	**Location**: Sadiyagad micro watershed, in mid-elevation zone of Central Himalaya<br><br>**Study period**: 1980-1996<br><br>**Focus**: Forest policy effects on cropland	Field investigations<br><br>Regional/local expertise<br><br>Remote sensing of land cover, topographical maps combined with socioeconomic data collected from conducting series of workshops & interviews with local population	Rao and Pant (2001)
B10	Barren/Wasteland → Cropland	• **People's participation**: Depends on people's perceptions , priorities & involving them in decision-making process<br>• Using traditional knowledge based agroforestry systems, with **water management** (irrigation capacity) as an integral component	**Location**: Banswara village, Chamoli district, Uttarakhand<br><br>**Study period**: 1990-1995 (period of model implementation)<br><br>**Focus**: Restoration of degraded community	Survey of 219 HH (>85% of HH in village)	Maikhuri et al. 1997

			lands/abandoned agricultural lands with various degrees of degradation		
B11	Fallow/Grass land/Barren/ Wasteland → Cropland	• **Soil & water conservation** through watershed development increased ground water recharge for irrigation<br>• **Diversification** to short-term **water-efficient cash crops** from perennial (traditional) crops that boost income of farmers	**Location**: Rajasamadhiyala (Gujarat) & Shekta (Maharashtra) watershed located in semi-arid regions<br><br>**Study period**: 1998-2005<br><br>**Focus**: Restoration of degraded land	Interview of 20% of farmers in each watershed selected through stratified random sampling	Wani et al. 2011
B12	Barren/Shrub land → Cropland	• **Increasing population pressure**<br>• **Altitude & land availability for clearing**: most cropland expansion in middle zone; higher zone unfit for cultivation; no land available in lower zone<br>• Out-migration in lower zone villages due to better access to road, educational facilities & increased willingness to buy property outside the region reduces some pressure on land, which partly compensates for increasing population pressure	**Location**: High altitude, cold desert of Lahaul-Spiti district, Himachal Pradesh<br><br>**Study period**: not mentioned	300 HH surveys collected across 10 villages lying across three altitudinal zone (>4500 m; 3000-4500 m; <3000 m)<br><br>Secondary data on land cover, census, & topography	Warpa and Singh (2014)
B13	Barren/Shrub land/Wasteland → Cropland	• **Higher education** (exposure)<br>• **Attitude** towards Jatropha/perception of risk<br>• More **income dependency of agriculture**<br>• **Availability of support services**: technical help from non-governmental organization & agricultural department<br>• Higher **minimum expected income** has negative effect: with higher minimum	**Location**: North East India. The authors use Assam & Arunachal Pradesh as sample to represent altitude & topography of other states in the region<br><br>**Survey period**: 2011-2012<br><br>**Focus**: Reclamation of wasteland for biofuel	144 key informant interviews in 23 villages in the two states	Choudhury and Goswami (2013)

		expected income, the possibility of getting that income goes down, which leads to non-fulfillment of the expectations of farmers, which discourages them to expand<br>• Insignificant factors (but were expected to be important): age of HH head, primary occupation, distance to nearest market, availability of unemployed family member, shortage of labor for agriculture, non-farm employment opportunity, expected price of jatropha seed, labor cost of jatropha, access to bank credit, & extension services	production (Jatropha)<br><br>**Note**: In this study, wasteland includes: land with/without scrub, land under shifting cultivation, degraded forestland. In North-East India, 46% of wasteland is in shrub, 17% in shifting cultivation, & 8% in degraded forest (scrub dominated).		
B14	Barren/Waste land → Cropland	• **Community-based watershed management** through customary governance institutions, local user groups & non-governmental organizations:<br>➢ improved the water table<br>➢ increased perenniality of water wells<br>➢ increased the availability of water for livestock & domestic use	**Location**: Tamil Nadu<br><br>**Study period**: 1990-present<br><br>**Focus**: An assessment of overall performance of watershed development programs on restoring degraded lands (non-forest wasteland)	Synthesis of published case studies	Kuppannan and Devarajulu (2009)
B15	Barren/Waste land → Cropland	• **Community-based watershed management**:<br>➢ improved ground water recharge & availability in both upstream & downstream villages<br>➢ better economic returns (more crop yields) & stable livelihood for farmers	**Location**: Rajasamadhiyala micro-watershed, Rajkot district, Gujarat<br><br>**Study period**: 1995-2003<br><br>**Focus**: Impact assessment of a watershed that was created in 1983	Focused group discussion & stratified detailed HH surveys of 20% of farm HH/farmers in study site (on-site) & two villages downstream (off-site assessment)	Sreedevi et al. 2006
B16	Fallow/Wast	• **Farmers attitude**: willingness to invest	**Location**: Six villages in	Survey of 60 HH	Shiferaw et

	eland/Barren → Cropland	in maintaining current fertility levels than restoring degraded lands<br>• **Access to new production & resource management technology** through watershed management program<br>➢ Better recharging of ground water<br>➢ Shift towards paddy & irrigated crops (vegetables) that cannot be sustained in water-scarcity<br>• **Higher education** (exposure):<br>➢ More investment in soil-water conservation<br>➢ More access to information<br>• Higher perceived returns to investments on land	Rangareddy district, Andhra Pradesh<br><br>**Survey period**: 2001-2002<br><br>**Focus**: Restoring degraded agricultural land through natural conservation management<br><br>**Context**: The six villages are semi-arid regions prone to droughts, water scarcity & soil degradation. One village is under community water shed management program, & other five adjoining villages are not.	within each village<br><br>Detailed plot- & crop-wise input & output data collected from all operational holdings of surveyed HH (n=568)	al. 2006a; Shiferaw et al. 2006b

Banerjee H, Goswami R, Chakraborty S, Dutta S, Majumdar K, Satyanarayana T, Jat ML, Zingore S (2014) Understanding biophysical & socio-economic determinants of maize (Zea mays L.) yield variability in eastern India. NJAS-Wageningen. J. Life Sci. 70: 79-93.

Chandra N, Singh RP (1992) Determinants & impact of new technology adoption on tribal agriculture I Bihar. Indian J. Agric. Econ. 47: 397-403.

Choudhury HK, Goswami K (2013) Determinants of expansion of area under jatropha plantation in North East India: A Tobit analysis. Forest Policy Econ. 30:46-52.

Kumar A (2009) Rural employment diversification in Eastern India: Trends & determinants. Agric Econ Res Rev. 22: 47–60.

Kumar C, Singhb SP (2012) Determinants of agricultural land use diversification in eastern & northeastern India. J Agric Food Syst Community Dev. 3: 73-98.

Kuppannan P, Devarajulu SK (2009) Impacts of Watershed Development Programmes: Experiences and Evidences from Tamil Nadu. Agricultural economic research review 22: 387-396.Maikhuri RK, Semwal RL, Rao KS, Saxena KG (1997) Agroforestry for rehabilitation of degraded community lands: a case study in the Garhwal Himalaya, India. Int. Tree Crops J. 9: 91-101.

Nadkarni MV, Deshpande RS (1979) Underutilization of land: climatic or institutional factors? Indian J.Agric. Econ. 34: 1-16.

Nüsser M, Schmidt S, Dame J (2012) Irrigation & development in the Upper Indus Basin: characteristics & recent changes of a socio-hydrological system in Central Ladakh, India. Mt Res Dev.32: 51-61. doi: http://dx.doi.org/10.1659/MRD-JOURNAL-D-11-00091.1

Ramasamy C, Balasubramanian R, Sivakumar SD (2005) Dynamics of Land Use Pattern with Special Reference to Fallow Lands-An Empirical Investigation in Tamil Nadu. Indian J.Agric. Econ. 60: 629.

Rao KS, Pant R (2001) Land use dynamics & landscape change pattern in a typical micro watershed in the mid elevation zone of central Himalaya, India. Agric Ecosyst Environ. 86: 113-124. Doi:http://dx.doi.org/10.1016/S0167-8809(00)00274-7

Robbins P (2001) Tracking invasive land covers in India, or why our landscapes have never been modern. Ann Assoc Am Geogr. 91: 637-659. doi: 10.1111/0004-5608.00263

Shiferaw BA, Reddy VR, Wani SP, Rao GN (2006, a) Watershed management & farmer conservation investments in the semi-arid tropics of India: analysis of determinants of resource use decisions & land productivity benefits. J. Sat Agric. Res. 2: 1-25.

Shiferaw BA, Reddy VR, Wani SP, Rao GN (2006,b) Watershed management & farmer conservation investments in the semi-arid tropics of India: analysis of determinants of resource use decisions & land productivity benefits.Working Paper Series - International Crops Research Institute for the Semi-Arid Tropics, 16 pp 28.

Sreedevi TK, Wani SP, Sudi R, Patel MS, Jayes T, Singh SN, Shah T (2006) On-site & off-site impact of watershed development: a case study of Rajasamadhiyala, Gujarat, India. J. Sat Agric. Res. 2: 1-44.

Wakeel A, Rao KS, Maikhuri RK, Saxena KG (2005) Forest management & land use/cover changes in a typical micro watershed in the mid elevation zone of Central Himalaya, India. Forest Ecol Manag 213: 229-242. Doi:http://dx.doi.org/10.1016/j.foreco.2005.03.061

Wani SP, Anantha KH, Sreedevi TK, Sudi R, Singh SN, D'Souza M (2011) Assessing the environmental benefits of watershed development: Evidence from the Indian semi-arid tropics. J. Sustain Watershed Sci. Manag. 1: 10-20.

Warpa V, Singh H (2014) Cultivable Land & the Nature of Its Distribution in Cold Desert of Lahaul-Spiti District of Himachal Pradesh. In Landscape Ecology & Water Management (pp. 115-127). Springer Japan.

Wood SA, Jina AS, Jain M, Kristjanson P, DeFries RS (2014) Smallholder farmer cropping decisions related to climate variability across multiple regions. Glob. Environ. Chang.25: 163-172. Doi:http://dx.doi.org/10.1016/j.gloenvcha.2013.12.011

Table S10. Summary of studies (N=42) on forest area loss. Keywords in the third column are highlighted in bold for skimming.

Study #	LULCC	Key results	Study details	Methods	Ref
C1	Forest → Shrub/Barren	• **Illegal forest encroachment** & clear felling<br>• **Wood extraction for subsistence** by local village communities for house construction/repair, fuel wood, & manufacturing agricultural equipment's<br>• Illegal **cattle grazing** by villagers that inhibit regeneration of forests<br>• Conversions higher near forest edges (proximity to roads & settlements)	**Location**: Bhanupratappur forest division, Kanker district, Chhattisgarh<br><br>**Study period**: 1990-2000<br><br>**Focus**: Deforestation & forest degradation	Field investigations<br><br>Regional/local expertise<br><br>Statistical modeling (Landsat + secondary data on explanatory factors)	Kumar et al. 2014
C2	Forest → Shrub/Barren	• **Industrial development** due to availability of large coal reserves, & construction of Gobind Ballabh Pant Sagar reservoir<br>• **Land exploitation** for surface water, ground water, coal, building material, industrial waste disposal, quarrying for limestone, establishment of thermal power stations, cement factory, & construction of reservoirs	**Location**: Singaruli district, Madhya Pradesh<br><br>**Study period**: 1978-2010<br><br>**Focus**: Deforestation & forest degradation	Regional/local expertise<br><br>Historical literature<br><br>Land cover/change detection using satellite imagery & GIS mapping	Areendran et al. 2013; Singh et al. 1991; Singh et al. 1997; Khan and Javed (2012)
C3	Forest → Shrub/Barr	• **Poor land management** & forest fire<br>• **Wood extraction for subsistence**	**Location**: Dabka watershed, Kosi Basin in Lesser	Regional/local expertise	Rawat et al. 2012

	en	• **Overgrazing**<br>• **Soil erosion** & accelerated runoff from substandard construction of roads & buildings<br>• **Social/population pressure**	Himalayas, Nainital district<br><br>**Study period**: 1990-2010<br><br>**Focus**: Oak & Pine forests	Field investigations<br><br>Land cover/change detection using satellite imagery & GIS mapping	
C4	Forest → Shrub/Barren	• **Industrial development** (large-scale hydro-electric project)	**Location**: Sainj Valley, fragile mountain ecosystems of the Western Himalayas<br><br>**Study period**: 2005-2010<br><br>**Focus**: Deforestation & forest degradation	Land cover/change detection using satellite imagery & GIS mapping	Jolli (2012)
C5	Forest → Barren/Wasteland	• **Over-extraction of fodder for livestock** on common land	**Location:** Three dryland, drought-prone regions (Surendranagar, Amreli, & Jamnagar) of Surashtra, Gujarat<br><br>**Survey period**: circa 2005<br><br>**Focus**: Degradation on common pool resources	Survey of 1227 HH in six villages	Shah (2010)
C6	Forest → Shrub/Barren	• Timber harvesting at large scale/**unregulated management actions**<br>• **Wood extraction for subsistence** (fuel wood)<br>• **Shifting cultivation**, illegal encroachments (for agriculture) & unsustainable land use practices on land deforested for agriculture<br>• **Road constructions**<br>• **Overgrazing**	**Location**: Malkangiri district, Orissa<br><br>**Study period**: 1973-2004<br><br>**Focus**: Deforestation & forest degradation	Regional/local expertise<br><br>Field investigations<br><br>Land cover/change detection using satellite imagery & analysis of historical maps	Pattanaik et al. 2011

		• **Social/population pressure**			
C7	Forest → Shrub/Barren	• **Wood extraction for subsistence** (fuel wood)<br>• Livestock **grazing** & fodder<br>• **Illegal clear felling** & timber collection for household & agricultural purposes<br>• **Natural factors** (e.g. fire, mortality by insects, diseases)	**Location**: Eastern Ghats of Tamil Nadu<br><br>**Study period**: 1990-2003<br><br>**Focus**: Deforestation & forest degradation	Satellite mapping of land cover<br><br>Field investigations<br><br>Interviewing local people during the investigations	Jayakumar et al. 2009 and references cited therein
C8	Forest → Shrub/Barren	• Free **cattle grazing** by large land owners, in response to emerging milk markets<br>• **Wood extraction for subsistence** (fuel wood for cooking & heating water, sold to small-scale businesses & food stalls)<br>• Cut-&-carry **fodder** collection<br>• **Encroachment for agriculture** by farmers with large land holding<br>• **Illegal felling** & lopping in reserve & village commons<br>• **Population/social pressure**<br>• **Weak/inefficient institutional framework** for protection & monitoring (corruption, misunderstanding, alienation, & mistrust between forest department & villagers)<br>• **Increased dependence of poor on forest** due to low farm productivity (low technology)<br>• Small stone mines, & small timber collection	**Location**: Protected areas of Sariska Tiger Reserve, Eastern Rajasthan<br><br>**Study period**: 1980-2000<br><br>**Focus**: Deforestation & forest degradation<br><br>**Focus**: A case of inefficient forest protection by Joint Forest Management, where villagers & forest department jointly manage state forests & share forest revenues.	Group surveys in 37 villages<br><br>180 HH surveys in a subset of 4 randomly sampled villages	Heltberg (2001)
C9	Forest → Shrub/Barren	• **Wood extraction for subsistence** (fuel wood mainly for water heating in high rainfall region)<br>• **Fodder** collection for livestock	**Location**: Five protected areas of Eastern & Western Ghats of Peninsular India	Survey of 1245 HH from villages across the study sites	Davidar et al. 2010

		• Collection of green leaves for producing green manure sold to local plantation industries<br>• **Income dependence on forests** (inversely related to proportion of agricultural households).<br>• Proportions of wage labor households indicating more dependence on forests<br>• **Local markets based on tourism** (e.g. tea shops that use wood for energy)	**Survey period**: Various (in 1990s & 2000s depending on the study site)<br><br>**Focus**: Forest degradation		
C10	Forest → Shrub/Barren	• **Wood extraction for subsistence** (fuel wood, fodder & other products)<br>• **Illicit felling** by local people<br>• **Encroachment** (land clearing) of land **for agriculture**	**Location**: Pulianjolai Reserved Forests, Kolli hills of the Eastern Ghats of Tamil Nadu<br><br>**Study period**: 1990-1999<br><br>**Focus**: Forest degradation	Regional/local expertise<br><br>Satellite mapping	Jayakumar et al. 2002
C11	Forest → Cropland, Shrub/Barren	• Most deforestation is for **agriculture**<br>• **Increasing population pressure** (human & animal)<br>• **Wood extraction for subsistence** (food, fuel, fodder, manure, & non-timber forest products)<br>• **Overgrazing** by the livestock (removes regenerating seedlings through browsing, trampling, reduces natural regeneration)<br>• **Clear-felling for industrial wood material extraction** (1963-80) due to lack of forest policy<br>• Government intervention for integrated land use & natural resource management (1980-95) reduced deforestation & clear felling:	**Location**: Sadiyagad micro watershed, in mid-elevation zone of Central Himalaya<br><br>**Study period**: 1962-1996<br><br>**Focus**: Deforestation & forest degradation	Field investigations<br><br>Regional/local expertise<br><br>Satellite mapping & topographical maps combined with socioeconomic data collected from conducting series of workshops & interviews with local population	Rao and Pant (2001)

		Intervention through technology transfer & efficient agricultural services (e.g. irrigation, access to credits & local markets, subsidized agricultural inputs liker fertilizers, soil & water conservation programs, & promotion of agroforestry).			
C12	Forest → Cropland, Shrub/Barren	• **Deforestation for agriculture** in people managed forests<br>• **Wood extraction for subsistence** in government reserved forests (lopping of broad leaved trees)<br>• Strong **institutional framework** protected cropland expansion in reserved forests<br>• Oak forest degradation (promotes pine forest growth) by locals in government forests, because **oak forests generate no revenue to forest department**.	**Location**: Kuchgad micro watershed, Almora District, Central Himalayas<br><br>**Study period**: 1967-1997<br><br>**Survey period**: 1997-2000<br><br>**Focus**: Deforestation & forest degradation<br><br>**Related Study**: See Dhyani et al. (2006) who conducted studies in Khootgad & Mohnagad watershed in Central Himalayas where fodder production expanded to community land (due to emerging milk markets).	Discussion interviews with officials of management institutions (e.g. forest department, *vanpanchayats*, & local inhabitants in village meeting)<br><br>Regional/local expertise<br><br>Interpretation of satellite data, information on legal & policy changes	Wakeel et al. 2005
C13	Forest → Shrub/Barren	• Demand for **non-timber forest products**: growing market demand for medicinal plants<br>• **Population/social pressure** & increased consumption<br>• **Weak institutional framework**<br>• Need for cash within tribal society<br>• Joint Forest Management & private farm forestry reduced the pressure on forests	**Location**: Madhya Pradesh state<br><br>**Study period**: 1990s & early 2000s<br><br>**Survey period**: 2005-2006<br><br>**Focus**: Forest degradation & forest protection in protected	Interview with 34 forest officers of different rank, policy-makers, representatives of local forest user organizations, & forest/livelihood experts from civil society & academia	Véron and, Fehr (2011)

		• Less **income dependence on forests** by locals reduced the pressure on forests	areas	Qualitative data from 4 forest-dependent villages, & 7 villages where forests was not a focus	
C14	Forest → Cropland & Plantations, Shrub/Barren	• **Unplanned in-migration** from surrounding regions:<br>➢ Uncontrolled deforestation<br>➢ **Overgrazing** by cattle<br>➢ **Wood extraction** (fire wood)<br>• Emergence of plantations (e.g. rubber, tea) by in-migrants from land alienation/appropriation of tribe's land & government sponsored programs<br>• Degradation from **shifting cultivation**, cattle grazing by tribes as their land holdings decreased<br>• Implementation of **land reform** measures: Feudal landlords deforested private forests quickly to timber traders at throw away prices (circa 1960)	**Location**: Two villages in Attappady, Palakkad district, Kerala<br><br>**Study period**: Trends applicable after 1930s to present<br><br>**Survey period**: circa 1995<br><br>**Focus**: Compare & contrast the land use & crop selection patterns of aboriginals & settlers	Survey of 367 farm HH (6% sample) in two villages<br><br>Secondary data on settlements & land use patterns	Velluva and Velluva (2006)
C15	Forest → Cropland, Shrubland/ Barren	• **Encroachment for agriculture**<br>• Severe **infections**<br>• Urbanization/**population pressure**<br>• **Climate change** (shifts in vegetation due to low moisture)	**Location:** 11 study sites in Dehradun forest division<br><br>**Study period**: 1976-1999<br><br>**Focus**: Sal forest deforestation & degradation	Regional/local expertise<br><br>Satellite mapping & aerial photographs	Chauhan et al. 2003
C16	Forest → Shrubland	• **Availability of land for clearing**<br>• **Topography & Altitude**: forests are relatively stable in complex terrains & higher altitudes (proxy of accessibility)<br>• **Accessibility**: Proximity to roads & settlements	**Location:** Balkhila sub-watershed, Garhwal Himalayas<br><br>**Study period**: 1991-2001<br><br>**Focus**: Forest degradation	Regional/local expertise<br><br>Field investigations<br><br>Satellite mapping & topographic sheets	Joshi and Gairola (2004)

			(Oak to Pine forests) & fragmentation		
C17	Forest → Cropland, Shrubland, Bareland	• **Promotion of** tea **plantations** by government to economically mitigate the risk of agriculture<br>• Large-scale deforestation by **plywood industry** (prior 1996 until ban was imposed)<br>• **Population pressure** (resident & immigrant) driven by economic & political factors<br>• Urbanization & **industrialization**<br>• Inability of industrial sector to combat **unemployment** that shifted pressure on forests<br>• Natural calamities (earthquakes) lead to **proliferation of landless people**, opening up forest land for settlements<br>• **Low level of protection & monitoring** due to civil/society unrest (insurgency), & interstate conflicts along state borders	**Location:** Upper Brahmaputra Valley, Assam<br><br>**Study period**: 1947-present<br><br>**Focus**: Deforestation & forest degradation	Regional/local expertise<br><br>References supporting arguments	Sharma et al. 2012
C18	Forest to Cropland, Shrubland, Bareland	• Increasing **population pressure**<br>• Increasing need for **timber**<br>• **Wood extraction** for firewood	**Location**: Doodhganga watershed in Kashmir Himalayas<br><br>**Study period**: 1991-2005	Regional/local expertise<br><br>Statistical model linking satellite derived land cover maps with ground data on population & stream discharge	Showqi et al. 2014
C19	Forest → Shrubland, Bareland	• **Wood extraction for subsistence** (fuel wood, fodder & auxiliary non-timber forest products)<br>• **High income dependence on forest** by poor people with small land holdings	**Location**: Nainital district, Kumaoan Himalayas, Uttar Pradesh<br><br>**Survey period**: 1996	Survey of 233 HH based on stratified random sampling in 12 villages within the district	Reddy and Chakravarty (1999)

		• **Weak institutional framework** for common property rights/participatory resource management (involving local communities & public agencies)	**Focus**: Forest degradation; Impacts of restricting access to common property (forest products) on poverty		
C20	Forest → Cropland, Shrubland, Bareland	• **Encroachment for agriculture**<br>• **Wood extraction for subsistence** (fuel wood, fodder, & small timber collection)<br>• **Overgrazing**<br>• **Accessibility** to reserve forest & forest stocks (collection time/kg; family labor inputs)<br>• Large land owners substitute private fuels from farms (private trees) over forest fuel wood i.e. fragmentation of **land holdings** increase pressure on forests<br>• **Poor housing construction**: economically poor people (scheduled caste/tribes) use more wood for better heating in winter<br>• **Weak institutions** for natural resource management/forest protection	**Location**: Villages in the vicinity of Saariska Tiger Reserve, Alwar District, Rajasthan<br><br>**Survey period**: 1996-1997<br><br>**Focus**: Forest degradation; How people adapt to forest degradation to meet energy requirements	Survey of 180 HH in 4 villages (25% stratified random sampling) located at varying distance from the reserve	Heltberg et al. 2000
C21	Forest → Shrub/Barren	• **Coal mining:** major force of forest degradation in the study region.	**Location**: Part of Jaintia hills district, Meghalaya<br><br>**Study period**: 1975-2001<br><br>**Focus**: Forest degradation<br><br>Similar study of the same region was done by Prakash & Gupta (1998).	Field work & Regional knowledge<br><br>Remote sensing	Sarma (2005) Also see Prakash and Gupta (1998)
C22	Forest →	• **Population pressure:** Increased	**Location**: Six micro-	Intensive field	Tiwari

	Shrub/Barren	demand for natural resources in the region.<br>• **Excessive grazing.**	watershed in Lesser Himalayan Ranges & Siwalik Hills, Nainital district, Kumaon.<br><br>**Study period**: 1975-2005<br><br>**Focus**: General changes in land dynamics	surveys, observations, monitoring, and socioeconomic surveys.<br><br>Remote sensing and topographical maps	(2008)
C23	Forest → Shrubland, Bareland	• **Intentional forest-fires** by forest dwellers:<br>➢ To ensure current vegetation forms on stand & at landscape level remain consistent as they produce flow of specific ecosystem services (e.g. pasture, bodha grass used for roof thatching)<br>➢ **Fuel-wood** for domestic requirements (becomes available when trees are fully/partially burnt)<br>➢ To remove grass & reduce thickness of shrubs to enable **accessibility** for fuel wood collection	**Location**: Sadhukonda reserve forest, Chittoor district, Andhra Pradesh<br><br>**Survey period**: Late 2000s<br><br>**Focus**: Forest degradation through forest fires, or maintaining forests at degraded levels	Interview of 557 HH in 14 villages around the reserve forest	Schmerbeck et al. 2015
C24	Forest → Shrubland, Bareland	• **Intentional forest-fires** by forest dwellers:<br>➢ **Fuel wood** utilization<br>➢ Fire driven **fodder** for livestock	**Location**: Kadava-kurichi Reserved Forest & neighboring villages, Dindigul district, Tamil Nadu<br><br>**Survey period**: 1998-2000<br><br>**Focus**: Degraded dry forests	Survey of 473 HH from 19 villages that included both users & non-forest users from reserve forests	Schmerbeck (2003); Schmerbeck and Seeland (2007); Schmerbeck (2011)
C25	Forest → Shrubland, Bareland	• **Intentional forest-fires** by forest dwellers:<br>➢ Practice **shifting cultivation** by local tribes	**Location**: Biligiri Rangaswamy Temple Wildlife sanctuary, Karnataka	21 interviews in 5 villages with individuals knowledgeable in the	Roveta RJ (2008)

		➢ Litter fire in dry season to **prepare cultivation land** (e.g. to fertilize land, clean understory vegetation for security & mobility, control pests & diseases, improve food & fodder production)<br>➢ Collection of **non-timber forest products** after ban on shifting cultivation	**Survey period**: Circa 2005<br><br>**Focus**: Forest degradation<br><br>**Note**: See Saigal (1990) and Semwal et al. 2003 for similar case studies on fire-driven ecosystem services in Central India (fires to produce/collect tendu leaves used in cigarettes), & North-central India (fires to produce/collect a particular flower used in liquor).	use of fire in the area	
C26	Forest → Cropland, Plantations	• **Availability of land** for clearing (aspect)<br>• **Topography & Altitude**: forests are relatively stable in complex terrains & higher altitudes (proxy of accessibility)<br>• **Industrialization & economic growth**<br>• **Level of protection**	**Location:** Cauvery river basin, Karnataka<br><br>**Study period**: 2001-2006	Regional/local expertise<br><br>Statistical modeling of satellite land cover with explanatory factors	Lele et al. 2010
C27	Deforestati on & Forest Degradatio n	• Due to "**scientific management**" during 1977-1989.<br>• **Grazing cattle**'s from surrounding villages.<br>• Hydro-electric **dam construction**<br>• **Uncontrolled forest resource extraction** by villagers for minor forest products.	**Location**: Pench Tiger Reserve (PTR), Nagpur District, Maharashtra.<br><br>**Study period**: 1977-2007<br><br>**Focus**: Effects of parks on forestry	Field investigations<br><br>Remote Sensing	Mondal and Southworth (2010)
C28	Deforestati on/Forest degradation	• **Selective logging**<br>• **Clear felling for commercial plantations** (coffee, tea, and cardamom)<br>• **Forest fire**<br>• **Wildlife grazing**<br>• **Illegal invasion** after clearance	**Location**: Kalakad-Mundanthurai Tiger Reserve, South Western Ghats<br><br>**Study period**: 1973-2004	Detailed remote sensing analysis<br><br>Regional knowledge for interpretation of change	Giriraj et al. 2008

		• **Soil erosion** from human pressure	**Focus**: Forest cover change in biodiversity rich region		
C29	Forest → Shrub/Barren	• **Flouted mining regulations**	**Location**: Part of Bokaro district, Jharkhand<br><br>**Study period**: 1972-2006<br><br>**Focus**: Forest degradation	Detailed remote sensing analysis of changes in mining areas.<br><br>No ground work.	Malaviya et al. 2010
C30	Forest → Shrub/Barren/Wasteland	• **Mining**<br>• Increasing employment in mining: **Fire wood extraction** for cooking purpose<br>• Wood for sharpening of the tools of mining.	**Location**: Bijola mining area, Rajasthan.<br><br>**Study period**: 1971-1991<br><br>**Focus**: Impacts of mining on human ecosystem	Remote sensing<br><br>Regional expertise	Chauhan (2010)
C31	Deforestation	• **Government policy** that encouraged agricultural production (plantations), and migration from coastal to upland regions.<br>• Major investments in power and irrigation projects for **reservoirs and infrastructures**	**Location**: Kerala.<br><br>**Study period**: Late 1950s to 2000.<br><br>**Focus**: Impacts of growing population on land use patterns.	Analysis of several case studies from the region.	Wolman et al. 2001
C32	Forest → Cropland	• **Higher altitudes/favorable climatic conditions** reflecting farmer's attitude to maximize income<br>• **Moderate slopes** where traditional terracing was feasible<br>• Encroachment due to **weak institutional arrangements** of forest protection & lack of monitoring (especially community & protected forests)<br>• **Income dependence of local community on forests** ensures forest	**Location**: Pranmati watershed, Uttar Pradesh, Central Himalayas<br><br>**Study period**: 1963-1993<br><br>**Survey period**: 1994-1995<br><br>**Focus**: 60% agricultural expansion from community forest; 35% from protected forests; & 5% from reserved	Integration of data from existing maps, satellite mapping of land cover, participatory survey with villagers, & field measurements	Semwal et al. 2004; Sen et al. 2002

		protection (especially reserved forests)	forest. Locals generate income from reserved forests by pine resin extraction.		
C33	Forest → Cropland	• **Illegal felling** from increased level of insurgency<br>• Force settlement of **immigrant** population from neighboring countries for political reasons	**Location**: Sontipur district, Assam<br><br>**Study period**: 1994-2001<br><br>**Focus**: Deforestation in reserve forests	Regional/local expertise<br><br>Ground investigations<br><br>Satellite mapping of land cover	Srivastava et al. 2002
C34	Forest → Cropland	• **Availability of land for clearing**: depends on **slope, altitude & aspect**<br>• **Population pressure**: presence of settlements & proximity to roads	**Location:** Balkhila sub-watershed, Garhwal Himalayas<br><br>**Study period**: 1991-2001<br><br>**Focus**: Conversion of scrub & low-density pine forest to agriculture	Regional/local expertise<br><br>Field investigations<br><br>Remote sensing of land cover & topographic sheets	Joshi and Gairola (2004)
C35	Forest → Built-up, Cropland, Plantations	• Literacy (proxy) explains over 50% of deforestation in the region because literacy rate is population centric & leads to **development pressure** in neighboring forests	**Location**: Kerala state<br><br>**Study period**: 1961-1988<br><br>**Focus**: Deforestation trends	Regional/local expertise<br><br>Statistical modeling using panel data on district level statistics on land use & socioeconomic factors	Sivaram (2003)
C36	Forest → Built-up	• **Industrial development** of large-scale hydro-electric project	**Location**: Sainj Valley in fragile mountain ecosystems of the Western Himalayas<br><br>**Study period**: 2005-2010	Regional/local expertise<br><br>Satellite mapping of land cover	Jolli (2012)
C37	Mangrove Forest degradation	• **Social/population pressure**<br>• **Agricultural reclamation**<br>• **Wood extraction for subsistence** (fuel wood & construction materials)	**Location**: Bhitarkanika Conservation Area, Orissa (East coast of India)	Land cover/change detection using satellite imagery & GIS mapping	Ambastha et al. 2010

		• **Industrial development** (construction of jetties, roads, defense structures, missile testing site, inshore fisheries by mechanized vessels)<br>• Lack of other alternative resources/**accessibility** to roads, waterways, & markets<br>• **Level of protection**	**Survey period**: Circa 2006<br><br>**Focus**: Mangrove forest degradation.<br><br>**Significance**: The study area has second largest mangrove forests in India.	Survey of 324 HH (10% sample) in 35 inhabited villages representing all community & economic groups	
C38	Forest → Water bodies	• Inundation from **dam construction** (Balimela dam & Upper Sileru dam)	**Location**: Malkangiri district, Orissa<br><br>**Study period**: 1973-2004	Regional/local expertise<br><br>Field investigations<br><br>Satellite mapping of land cover & analysis of historical maps	Pattanaik et al. 2011
C39	Forest → Water bodies	• Construction of **large-scale dams** over rivers to divert water for irrigation	**Location**: 234 villages that will be submerged by Sardar Sarovar Project in Narmada valley covering Gujarat, Maharashtra, & Madhya Pradesh<br><br>**Study period**: 2000s<br><br>**Focus**: Study explored the potential impacts of a government dam construction project underway. The dam was opened in 2006	Survey of 5% of total HH in 6 tribal villages upstream of dam area &<br>located at the border of Gujarat & Maharashtra<br><br>Secondary data on socioeconomics	Singh and Mathur (2001)
C40	Forest → Water bodies	• Conversion to **aquaculture farms** for production of export-quality shrimps<br>• **Increased demand for prawns**: conversion increased by setback of Thailand aquaculture industry due to	**Location**: Eight administrative units of Sundarban, the coastal zone of Bay of Bengal	Expertise on mangrove ecosystems/aquaculture in India	Kumar (2012)

		prawn disease outbreak<br>• **Population/social pressure**: encroachment into fragile areas<br>• **Net relative land productivity**: differentials in returns on forest relative to other land use<br>• **Un-accounting of ecological services**: mangroves are reported to have insignificant returns in official statistics, because only economic returns are accounted	**Study period:** 1986-2004<br><br>**Focus**: Conversion of mangrove forests to hatcheries	Statistical modeling of satellite land cover & economic data	
C41	Forest → Water bodies	• Single factor causation: **Dam construction**	**Location**: Three major river basins in the Indian Himalayas along which 292 dams are under-construction or proposed.<br><br>**Study period**: 2000s<br><br>**Focus**: Impacts of hydropower development on biological diversity	Remote sensing<br><br>Topographical Maps<br><br>Regional Expertise<br><br>The study also provides future projections based on modeling.	Pandit and Grumbine (2012); Grumbine and Pandit (2013)
C42	Forest → Wasteland	• Single factor causation: **Advancement of mining and industrial activity**.<br>• Population pressure due to employment opportunities cause **wood extraction for subsistence**.	**Location**: Talcher-Angul region, Orissa.<br><br>**Study period**: 1973-2011<br><br>**Focus**: Focused on land degradation.	Remote sensing<br><br>Topographical maps<br><br>Ground validation using Google imageries.	Panwar et al. 2011

Ambastha KR, Hussain SA, Badola R, Roy PS (2010) Spatial analysis of anthropogenic disturbances in mangrove forests of Bhitarkanika Conservation Area, India. J Indian Soc Remote Sens. 38: 67-83. doi:10.1007/s12524-010-0013-y

Areendran G, Rao P, Raj K, Mazumdar S, Puri K (2013) Land use/land cover change dynamics analysis in mining areas of Singrauli district in Madhya Pradesh, India. Trop. Ecol. 54: 239-250.

Chauhan PS, Porwal MC, Sharma L (2003) Change detection in Sal forest in Dehradun forest division using remote sensing & geographical information system. J Indian Soc Remote. 31; 211-218. doi:10.1007/BF03030827

Chauhan SS (2010) Mining, development and environment: a case study of Bijolia mining area in Rajasthan, India. J. Hum. Ecol. 31: 65-72.

Davidar P, Sahoo S, Mammen PC, Acharya P, Puyravaud JP, Arjunan M, Garrigues JP, Roessingh K (2010) Assessing the extent & causes of forest degradation in India: Where do we stand? Biol Cons. 143: 2937-2944. Doi:http://dx.doi.org/10.1016/j.biocon.2010.04.032

Giriraj A, Irfan-Ullah M, Murthy MSR, Beierkuhnlein C (2008) Modelling spatial and temporal forest cover change patterns (1973-2020): A case study from South Western Ghats (India). Sensors 8: 6132-6153. doi:10.3390/s8106132

Grumbine RE, Pandit MK (2013) Threats from India's Himalaya dams. Science 339: 36-37. doi: 10.1126/science.1227211

Heltberg R (2001) Determinants & impact of local institutions for common resource management. Environ Dev Econ. 6: 183-208.

Heltberg R, Arndt TC, Sekhar NU (2000) Fuelwood consumption & forest degradation: a household model for domestic energy substitution in rural India. Land Econ. 213-232.

Jayakumar S, Arockiasamy DI, John Britto S (2002) Forest type mapping & vegetation analysis in part of Kolli hills, Eastern Ghats of Tamil Nadu. Trop Ecol. 43:345–349.

Jayakumar S, Ramachandran A, Bhaskaran G, Heo J (2009) Forest Dynamics in the Eastern Ghats of Tamil Nadu, India. Environ manage. 43:326-345. doi:10.1007/s00267-008-9219-y

Jolli V (2012) Does hydro-electric project development causes land use-land cover change at landscape level? A case study of temperate forest of Western Himalaya. . Water Land Dev. 17-22.

Joshi PK, Gairola S (2004) Land cover dynamics in Garhwal Himalayas—a case study of Balkhila sub-watershed. J Indian Soc Remote Sens. 32: 199-208. doi:10.1007/BF03030876

Khan I, Javed A (2012) Spatio-temporal land cover dynamics in open cast coal mine area of Singrauli, MP, India. J Geogr Inf Syst. 4: 521-529. doi: 10.4236/jgis.2012.46057

Kumar P (2012) Impact of economic drivers on mangroves of Indian Sundarbans: an exploration of missing links. Environ Dev Sustain 14:939-953. doi:10.1007/s10668-012-9361-9

Kumar R, Nandy S, Agarwal R, Kushwaha SPS (2014) Forest cover dynamics analysis & prediction modeling using logistic regression model. Ecol. Indic. 45: 444-455. Doi:http://dx.doi.org/10.1016/j.ecolind.2014.05.003

Lele N, Nagendra H, Southworth J (2010) Accessibility, demography & protection: Drivers of forest stability & change at multiple scales in the Cauvery Basin, India. Remote Sens. 2: 306-332. doi:10.3390/rs2010306

Malaviya S, Munsi M, Oinam G, Joshi PK (2010) Landscape approach for quantifying land use land cover change (1972–2006) and habitat diversity in a mining area in Central India (Bokaro, Jharkhand). Environ. Monit. Assess. 170: 215-229. doi:10.1007/s10661-009-1227-8

Mondal P and Southworth J(2010) Protection vs. commercial management: Spatial and temporal analysis of land cover changes in the tropical forests of Central India. Forest ecol manage. 259:1009-1017. Doi:http://dx.doi.org/10.1016/j.foreco.2009.12.007

Pandit MK, Grumbine RE (2012) Potential effects of ongoing and proposed hydropower development on terrestrial biological diversity in the Indian Himalaya. Conserv. Biol. 26: 1061-1071. doi: 10.1111/j.1523-1739.2012.01918.x.

Panwar S, Sinha RK, Singh G (2011) Time sequential surface change analysis of Talcher-Angul region of Orissa using Remote Sensing and GIS. Int J Geomatics Geosci. 1: 828.

Pattanaik C, Reddy CS, Reddy PM (2011) Assessment of spatial & temporal dynamics of tropical forest cover: A case study in Malkangiri district of Orissa, India. J. Geographical Sci. 21: 176-192. doi:10.1007/s11442-011-0837-6

Prakash A, Gupta RP (1998) Land-use mapping and change detection in a coal mining area-a case study in the Jharia coalfield, India. Int J Remote Sens, 19: 391-410. Doi:http://dx.doi.org/10.1080/014311698216053

Rao KS, Pant R (2001) Land use dynamics & landscape change pattern in a typical micro watershed in the mid elevation zone of central Himalaya, India. Agric Ecosyst Environ. 86: 113-124. Doi:http://dx.doi.org/10.1016/S0167-8809(00)00274-7

Rawat PK, Tiwari PC, Pant CC (2012) Climate change accelerating land use dynamic & its environmental & socio-economic risks in the Himalayas: Mitigation through sustainable land use. Int J Clim Chang Str. 4: 452-471. Doi: http://dx.doi.org/10.1108/17568691211277764

Reddy SRC, Chakravarty SP (1999) Forest dependence & income distribution in a subsistence economy: evidence from India. World Dev. 27:1141-1149. Doi;http://dx.doi.org/10.1016/S0305-750X(99)00057-1

Roveta RJ (2008) Traditional use of fire for the provision of ecosystem services: a case study in BRT Wildlife Sanctuary. Ph.D. Thesis, Institute for Siviculture, University of Freiburg, Freiburg, Germany.

Saigal R (1990) Modern forest fire control: the Indian experience. Unasylva (FAO).

Sarma K (2005) Impact of coal mining on vegetation: A case study in Jaintia Hills District of Meghalaya, India. International institute for geo-information science and earth observation enschede (The Nederlands) and Indian institute of remote sensing, national remote sensing agency (NRSA), Department of space, Dehradun, India, 1-85.

Schmerbeck J (2003) Patterns of forest use & its influence on degraded dry forests: A case study in Tamil Nadu, South India. Shaker Verlag, Aachen, Germany.

Schmerbeck J (2011) Linking dynamics & locally important ecosystem services of South Indian dry forests: an approach. J Res Ene Dev. 8: 149-172. doi: 10.3233/RED-120090

Schmerbeck J, Kohli A, Seeland K (2015) Ecosystem services & forest fires in India—Context & policy implications from a case study in Andhra Pradesh. Forest Policy Econ. 50: 337-346. Doi:http://dx.doi.org/10.1016/j.forpol.2014.09.012

Schmerbeck J, Seeland K (2007) Fire supported forest utilisation of a degraded dry forest as a means of sustainable local forest management in Tamil Nadu/South India. Land Use Policy 24: 62-71. Doi:http://dx.doi.org/10.1016/j.landusepol.2006.01.001

Semwal R, Nautiyal S, Sen KK, Rana U, Maikhuri RK, Rao KS, Saxena KG (2004) Patterns & ecological implications of agricultural land-use changes: a case study from central Himalaya, India. Agric Ecosyst Environ. 102: 81-92. Doi:http://dx.doi.org/10.1016/S0167-8809(03)00228-7

Semwal RL, Chatterjee S, Punetha JC, Pradhan S, Dutta P, Soni S, Sharma G, Singh VP, Malayia A (2003) Forest Fires in India: Lessons from Case Studies. World Wildlife Fund (WWF) for Nature-India Publication, New Delhi, India.

Sen KK, Semwal RL, Rana U, Nautiyal S, Maikhuri RK, Rao KS, Saxena KG (2002) Patterns & implications of land use/cover change: a case study in Pranmati watershed (Garhwal Himalaya, India). Mt Res Dev. 22: 56-62. doi: http://dx.doi.org/10.1659/0276-4741(2002)022[0056:PAIOLU]2.0.CO;2

Shah A (2010) Land degradation & migration in a dry land region in India: extent, nature & determinants. Environ Dev Econ 15: 173-196. doi: http://dx.doi.org/10.1017/S1355770X09990131

Sharma N, Madhusudan MD, Sinha A (2012) Socio-economic drivers of forest cover change in Assam: a historical perspective. Econ Polit Wkly 47: 64-72.

Showqi I, Rashid I, Romshoo SA (2014) Land use land cover dynamics as a function of changing demography & hydrology. Geo J 79: 297-307. doi:10.1007/s10708-013-9494-x

Singh JS, Singh KP, Agrawal M (1991) Environmental degradation of the Obra-Renukoot-Singrauli area, India, & its impact on natural & derived ecosystems. Environmentalist 11: 171-180. doi:10.1007/BF01263230

Singh NP, Mukherjee TK, Shrivastava BBP (1997) Monitoring the impact of coal mining & thermal power industry on landuse pattern in & around Singrauli Coalfield using remote sensing data & GIS. J Indian Soc Remote. 25:61-72. doi:10.1007/BF02995419

Singh RB, Mathur A (2001) Large-scale dams as drivers of land use/cover change in the tropical deciduous forests - A case-study of Sardar Sarovar Project (SSP), India, In Land Use & Cover Change, (Ed) Singh RB; Fox J; Himiyama Y, pp. 138-152.

Sivaram M (2003) Socio-economic causes of deforestation in Kerala State: an exploration. Indian J Forestry 26: 291-294.

Srivastava S, Singh TP, Singh H, Kushwaha SPS, Roy PS (2002) Assessment of large-scale deforestation in Sonitpur district of Assam. Curr. Sci. 82: 1479-1484.

Tiwari P (2008) Land use changes in Himalaya and their impacts on environment, society and economy: A study of the Lake Region in Kumaon Himalaya, India. Adv. Atmos. Sci. 25: 1029-1042. doi:10.1007/s00376-008-1029-x

Velluva S, Velluva S (2006) Land settlement & degradation: dynamics of land use in recently settled forest areas of Kerala. Land settlement & degradation: dynamics of land use in recently settled forest areas of Kerala. Serials Publications, Delhi, India, pp 205.

Véron R, Fehr G (2011) State power & protected areas: Dynamics & contradictions of forest conservation in Madhya Pradesh, India. Polit. Geogr. 30: 282-293. Doi:http://dx.doi.org/10.1016/j.polgeo.2011.05.004

Wakeel A, Rao KS, Maikhuri RK, Saxena KG (2005) Forest management & land use/cover changes in a typical micro watershed in the mid elevation zone of Central Himalaya, India. Forest Ecol Manag 213: 229-242. Doi:http://dx.doi.org/10.1016/j.foreco.2005.03.061

Wolman MG, Ramakrishnan PS, George PS, Kulkarni S, Vashishtha PS, Shidong Z, Qiguo Z, Wenmei C, Yi Z, Wolman MC, Long JF, Rosenzweig C, Solecki WD (2001) Growing populations, changing landscapes: Studies from India, China, and the United States. Indian National Science Academy, Chinese Academy of Sciences and US National Academy of Sciences, National Academy Press, Washington, DC.

Table S11. Summary of studies (N=23) on forest area gain. Keywords in the third column are highlighted in bold for skimming.

Study #	LULCC	Key results	Study details	Methods	Ref
D1	Forest protection/regrowth	• **Legitimacy of ownership** increases protection<br>• **Degree of monitoring** (preventing illegal harvesting, over harvesting & over grazing)<br>• **Forest/population users ratio**: very high values imply ineffective forest management; very low values implies difficulties in coordination between users; nominal ratio is ideal<br>• **Flexibility to adapt management practices** in response to changing local social & ecological needs. State/National level plans is not the best for all cases	**Location**: Study sites spread throughout India & Nepal. Exact locations not provided.<br><br>**Study period**: 1990s & 2000s	Synthesis of published local case studies at forest/community level (from International Forestry Resources & Institutions)<br><br>Satellite mapping analysis at landscape level (few square km in each case study area)	Nagendra (2009)
D2	Forest protection/regrowth	• **Better institutional framework** through below points:<br>➢ **Clear specification of property rights** reducing distress out-migration, being a part of labor force allocation decisions.<br>➢ Property rights depend on levels of asset ownership (cattle, participating in common property rights).<br>• **Dependence on common land**<br>• Level of **education** (exposure)	**Location**: Udaipur district, Rajasthan<br><br>**Survey period**: 1994<br><br>**Focus**: Reduction of deforestation & forest degradation followed by forest area gains	Survey of 32-35 randomly sampled HH each in 6 villages	Chopra and Gulati (1998)

D3	Forest protection/regrowth	• Conflicts due to **non-transparency in allocation of resources & benefits** with involved communities<br>• **Lack of/inefficient conflict resolution mechanism**<br>• **Weak institutional arrangements**<br>• **Inadequate peoples participation** due to the autonomy of state forest department<br>• **Poor collaboration** between state forest department & people<br>• **Centralization of adaption management practices** that did not suit the changing local social & ecological needs.	**Location:** Forest tracts of Central & Central-Eastern parts of India, covering the states of Madhya Pradesh, Chhattisgarh, Jharkhand, Orissa, & West Bengal<br><br>**Study period**: 1990-2000<br><br>**Focus**: An example of ineffective forest protection/regrowth through Joint Forest Management	Focused group discussions with 25 Joint Forest Management committees<br><br>Survey of 10% stratified HH with predominately agriculture-based livelihood with forest-based earning & wage incomes from non-timber forest products	Bhattacharya et al. 2010; Rishi (2007)
D4	Afforestation of degraded forest	• **Compensatory afforestation** by government for forest lost due to dam construction<br>• Afforestation by replanting of uprooted trees, or sub-standardized plantations (lack the originality & gene pool of originally deforested forest)	**Location**: 234 villages that will be submerged by Sardar Sarovar Project in Narmada valley covering Gujarat, Maharashtra, & Madhya Pradesh<br><br>**Study period**: 2000s<br><br>**Focus**: Study explored the potential impacts of a government dam construction project underway. The dam was opened in 2006	Survey of 5% of total HH in 6 tribal villages upstream of dam area &<br>located at the border of Gujarat & Maharashtra<br><br>Secondary data on socioeconomics	Singh and Mathur (2001)
D5	Forest protection/regrowth	• **Empowering people** to involve in Joint Forest Management<br>• **Institution building** at the community level<br>• **Voluntary people participation**, depends on:	**Location:** Haryana, Uttar Pradesh, & Bihar<br><br>**Survey period**: 1995-1996<br><br>**Focus**: Cases of successful joint	Survey of 13 HH in each of the 10 villages, in three states with different institutional setting	Lise (2000)

		➢ Social: Attitude towards & benefit from village meetings<br>➢ Economic: dependence on forest, forest quality<br>• Peoples participation increases with **education** (exposure), & **women involvement**	forest management. Locals depend on forest for subsistence, hence their cooperation with state government is essential to forest management		
D6	Grassland/ Shrubland → Forest	• Passive force: **Natural regeneration** following land abandonment<br>• Active forces: **Conscious community effort** to restore forests due to religious & cultural practices (e.g. nature worship)<br>• **Formalization of land boundarie**s: Transition from shifting cultivation to settled agriculture<br>• **Social awareness** of forest loss & importance of natural resource management	**Location**: Anthropogenic tropical forest-agricultural landscapes in two forest groves sites in Kodagu district of Western Ghats<br><br>**Study period**: This study focus on century scale trends<br><br>**Focus**: Reforestation in forest groves (grass-dominated open landscapes to forests). Groves are small fragments of tropical forests that have received community protection , whereas in buffer zones of groves, forest decreases from land-use change	Field investigations<br><br>Ecological surveys & historical literature	Bhagwat et al. 2014
D7	Grassland/ Fallow → Shrubland → Forest	• **Localized succession** & **disturbance dynamics**: such forests are being dominated by invasive species that can mature within 8-10 years	**Location**: Godwar, Rajasthan<br><br>**Study period**: 1986-1999	Remote sensing of land cover; Historical data; HH production information; Discourse of planners & state experts	Robbins (2001)
D8	Forest protection/r egrowth	• **Perception of environment favors** peoples participation<br>• **Quality of forest**<br>• **Personal benefits from forest**<br>• Importance & personal **benefit from meetings**	**Location**: Paschim Medinipur district, West Bengal<br><br>**Survey period**: 2011<br><br>**Focus:** Factors that affect	Survey of 150 HH belonging to 31 forest protection committees using stratified random sampling	Jana et al. 2014

		• Size of HH & land holdings (**income dependency on forests** & labor availability)<br>• **Adequate institutional checks** & balances	participation in Joint Forest Management. Study region is a backward district with 34% of population below poverty line.		
D9	Forest protection/regrowth	• **Women's involvement** improves forest protection/management because:<br>➢ They are more dependent on forests for income (greater family income, who otherwise would be unemployed)<br>➢ More sensitive to the economically sustainable goals under participatory forestry	**Location**: Bankura district, Bengal<br><br>**Survey period**: 2005-2006<br><br>**Focus**: Example of the role of female forest protection committee on forest conservation projects	Survey of 431 HH in 8 villages both involved & not involved in Joint Forest Management	Das (2011)
D10	Forest expansion	• **Warming climate** causes an upslope shifting of existing forest species	**Location**: Dabka watershed, Kosi Basin in Lesser Himalayas, Nainital district, Uttarakhand<br><br>**Study period**: 1990-2010<br><br>**Focus**: Expansion of mixed forests	Regional/local expertise<br><br>Field investigations<br><br>Satellite mapping of land cover	Rawat et al. 2012
D11	Forest protection/regrowth	• **Cessation of commercial logging** by park management caused forest regrowth<br>• **Reduction in the intensity of land use** (in tea estates)<br>• Forest stability in complex **topography & low population** areas<br>• **Deforestation** towards less- or unprotected peripheral areas due to **illegal timber & furniture markets** by transportation networks (road & rail)	**Location**: Landscape surrounding the Mahananda Wildlife Sanctuary in Northern part of West Bengal<br><br>**Study period**: 1990-2000	Regional/local expertise<br><br>Discussions with local forest officials<br><br>Satellite mapping of land cover	Nagendra et al. 2009

D12	Forest protection/regrowth	• Declared as **protected area**.<br>• **National-level conservation policy** in 1998 that banned felling of forests in national parks.	**Location**: Pench Tiger Reserve (PTR), Nagpur District, Maharashtra.<br><br>**Study period**: 1977-2007<br><br>**Focus**: Effects of parks on forestry	Field investigations<br><br>Remote Sensing & GIS	Mondal and Southworth (2010)
D13	Forest Regrowth (Wasteland → Forests)	• **Promotion of secondary & tertiary sectors of economic activities** in the region, rather than focusing on traditional primary resource development practices.<br>• Effective implementation of **Joint Forest Management** (JFM).<br>• Creation of **village level participatory institutions** for management of village forests.<br>• **Joint Restoration efforts** by forest department and NGOs in collaboration with local community.	**Location**: Six micro-watershed in Lesser Himalayan Ranges & Siwalik Hills, Nainital district, Kumaon.<br><br>**Study period**: 1975-2005<br><br>**Focus**: General changes in land dynamics	Intensive field surveys, observations, monitoring, and socioeconomic surveys.<br><br>Remote sensing and topographical maps	Tiwari (2008)
D14	Forest protection/regrowth	• **Involvement of women and young people** – they support forest conservation.<br>• **Attitude**: **Dependence on forest products** reduces their willingness to support state forest department with conserving forests.<br>• Forest conservation was **independent of wealth**. All people depended on forests.	**Location**: Kalakad–Mundanthurai Tiger Reserve, Southern Western Ghats, India.<br><br>**Study period**: Circa 2000<br><br>**Focus**: Attitudinal evaluation of conservation of the local villagers after implementation of a World Bank funded eco-development project.	Twelve villages located within 3 km from the reserve boundary<br><br>2–3% of the total households surveyed totaling to 677 surveys	Arjunan et al. 2006
D15	Forest protection/regrowth	• **Collaboration of village community with government forest agencies** (Joint forestry management) with nested	**Location**: Study of three villages in the Aravalli Hills, South Haryana.	Perspective article by regional expert from the Forest department, Government of India.	Kumar (2013) Also see Bhattach

		levels of authority is crucial to forest conservation.<br>• Ethnic homogeneity, small to medium size, autonomy in decision-making and high dependence on forests is not always the main factors to forest conservation.<br>• Attention to both short-term and long-term interests makes forest conservation more resilient.	**Study period**: Not mentioned (roughly 2000s)<br><br>**Focus**: Contrasting three villages that have full title over the common<br>lands and forest, and have taken three radically different alternatives to conserve them.		arya et al. 2010; Dilip Kumar (2015); Prasad and Kant (2003)
D16	Forest protection/regrowth	• **Lack of interest by villagers**: Due to low productivity, long gestation, uncertain incentives, lack of foreseeing long-term benefit.<br>• **Improving institutional conditions of forestry workers** is important for efficient JFM.	**Location**: Tamil Nadu, South India.<br><br>**Study period**: 1997+<br><br>**Focus**: Assessment of forester's perspective on infectiveness in implementing Joint Forest Management (JFM).	Interview with 28 forest officers of varying rank s from 5 forest divisions with largest number of JFM villages and history of implementing them.	Matta et al. 2005
D17	Forest protection/regrowth	• **Attitude towards conservation**: Depends on exposure, village-type, resident's occupation, caste, source of fuel for cooking, and educational qualifications, and size of land holdings.<br>• Clearly **defining land ownership and rights on forest products** improves villager's participation to forest conservation.<br>• **Regular monitoring** (self) of the impact of right-holders on forests.	**Location**: Sontipur and Golaghat district, Assam, North-East India<br><br>**Survey period**: 2010<br><br>**Focus**: Attitudinal analysis of forest dwellers and encroachers.	Survey of 190 households in four village forests (~10%) and two encroached villages.<br><br>Combined with secondary data on geographical location and demographic pattern	Mahanta and Das (2013)
D18	Forest protection/regrowth	**Suggested solutions**:<br>• **Granting forest rights** to rural people, combined with government/external interventions<br>• **Extension of technical facilities**	**Location:** Ranibundh forest range, Bankura district, West Bengal.<br><br>**Study period:** 2000s	Survey of 50% of inhabitants in each of seven chosen villages within the study area.	Datta and Sarkar (2012)

		• **Alternative rural employment** prospects<br><br>**Causes of failure until now based on which above solutions were suggested**:<br>• Predominance of private agencies in marketing of non-timber forest productions<br>• Risk of eviction<br>• Loss of customary rights to access forest resources<br>• Low employment prospects<br>• Lack of training about proper management of non-timber forest	**Focus:** Examined the causes of failure to conserve forests by local community despite their income dependence on non-timber forest products		
D19	Forest protection/regrowth	• **Low levels of village participation**<br>• Increased risk of poaching and regional conflicts – due to trading loss of linkages with outside agencies and neighboring villages in favor of close linkage to forest department.<br>• **Politicizing the administration** of forest resource – creates inequality in forest rights among various economic sections of the community.	**Location:** Gadabanikilo village, Ranpur block, Nayagarh district, Orissa. The study block is an unit for the implementation of development activities by government.<br><br>**Survey period:** 2005<br><br>**Focus:** Examines the negative impacts of shift from self-organized community management to joint forestry management (pre- and post-2002).	Extensive knowledge of the village.<br><br>Examination of government written records.<br><br>23 semi-structured interviews at community, NGO, forest federation, and forest department levels. 9 focus group discussions at the community level.	Nayak and Berkes (2008)
D20	Forest protection/regrowth	• **Lack of motivation of villagers** to protect despite timber benefit sharing mechanism because:<br>➢ **Incentive sharing was not performance-based**<br>➢ **Limited information** provision	**Location:** West Chhindwara Forest Division, Madhya Pradesh<br><br>**Survey period:** 2010-11	Interviews with forest officers of varying ranks from five JFM committees (of 321 JFMs).	Ota et al. 2013

		mechanism - forest officers were the way to motivate people.<br>➢ **Exclusion of committee members from monitoring** of harvested timber (related to forest officers perception and attitudes).	**Focus:** Institutional design of timber benefit sharing mechanism under Joint Forest Management (JFM) and its effectiveness as incentive to forest protection	40 randomly selected household interviews in each of the 5 committees<br><br>Secondary information e.g. working plan of forest division, micro-plans of management committees.	
D21	Forest protection/regrowth	• **Women participation in JFM enhances forest protection.** Can be achieved through:<br>➢ Policies that empower women to establish their own management unit<br>➢ Involving them in policy planning and implementation<br>• **Dependence on forest resources** (physical or monetary or both) enhances involvement in JFM	**Location:** Bankura district, West Bengal.<br><br>**Survey period:** 2005-06.<br><br>**Focus:** Crucial role of women participation in Joint Forest Management (JFM).	431 household surveys from eight villages with and without JFM. The sampled villages consisted of three forest divisions of the district.	Das (2011)
D22	Forest protection/regrowth	• Lack of clear **proprietary rights**<br>• Lack of appropriate **conflict resolution** mechanism<br>• Increasing autonomy by revenue and forest department: **Lack of space for people's involvement**<br>• **Poor support system**<br>• **Centralization of working plans** and siviculture decisions does not suit local conditions.	**Location:** Two Van Panchayats in Nainital and Almora district, Uttaranchal. Two forest protection committee's from Midnapore district, West Bengal.<br><br>**Survey period:** Circa 2000<br><br>**Focus:** On the declining effectiveness of institutions in protecting forests.	Discussion with committee members (subset of 35 members) and villagers (~150 randomly sampled households in total).	Ballabh et al. 2002
D23	Forest protection/regrowth	• Lack of appropriate policy to improve the **economic situation of forest fringe dwellers** and to reduce unsustainable	**Location:** Three south-western districts (Purulia, Bankura, and West Midnapore) of West	Combination of primary and secondary data.	Ghosal (2014)

		extraction.<br>• **Ineffectiveness of Joint Forest management** (framed at national level) to work at micro-level.<br>• **Lack of monitoring** at ground-level.<br>• **Lack of involving women** in forest management committees due to social taboos (tribal region). Women have more knowledge of the forest surrounding as they are the prime collectors of non-forestry timber products.	Bengal.<br><br>**Survey period:** 2008-09<br><br>**Focus:** Effect of policies that promote systematic and sustainable harvesting of non-timber forest products on forest conservation.	Primary data included fieldwork in 11 villages (sample size not mentioned as only qualitative discussion was provided). Includes interview with forest officers, and villagers.<br><br>Secondary data included government reports, newspaper reports, research reports, and journal articles.	

Arjunan M, Holmes C, Puyravaud JP, Davidar P (2006) Do developmental initiatives influence local attitudes toward conservation? A case study from the Kalakad–Mundanthurai Tiger Reserve, India. J Environ Manage 79: 188-197. Doi:http://dx.doi.org/10.1016/j.jenvman.2005.06.007

Ballabh V, Balooni K, Dave S (2002) Why local resources management institutions decline: a comparative analysis of Van (forest) panchayats and forest protection committees in India. World Dev. 30: 2153-2167.doi: http://dx.doi.org/10.1016/S0305-750X(02)00126-2

Bhagwat SA, Nogué S, Willis KJ (2014) Cultural drivers of reforestation in tropical forest groves of the Western Ghats of India. For. Ecol. Manage. 329: 393-400. Doi: http://dx.doi.org/10.1016/j.foreco.2013.11.017

Bhattacharya P, Pradhan L, Yadav G (2010) Joint forest management in India: Experiences of two decades. Resour Conserv Recycl. 54: 469-480. Doi:http://dx.doi.org/10.1016/j.resconrec.2009.10.003

Chopra K, Gulati SC (1998) Environmental degradation, property rights & population movements: hypotheses & evidence from Rajasthan (India). Environ Dev Econ. 3: 35-57.

Das N (2011) Women's dependence on forest & participation in forestry: A case study of joint forest management programme in West Bengal. J Forest Econ. 17: 67-89. doi:http://dx.doi.org/10.1016/j.jfe.2010.09.003

Datta SK, Sarkar K (2012) NTFPs and Their Commercialization Issues from the Perspective of Rural Livelihood and the State of Forest Resources: A Study of the Ranibundh Forest Range in West Bengal, India. J Sustainable Forestry 31: 640-660. Doi: http://dx.doi.org/10.1080/10549811.2012.678097

Dilip Kumar PJ (2015) Between Panchayat, Community and the State: The Case for Joint Institutions for Managing Forests in India. Administrative Culture 16: 4-23.

Ghosal S (2014) The Significance of the Non-Timber Forest Products Policy for Forest Ecology Management: A Case Study in West Bengal, India. Environ. Policy Governance. 24:108-121. doi: 10.1002/eet.1630

Jana SK, Lise W, Ahmed M (2014) Factors affecting participation in joint forest management in the West Bengal state of India. J Forest Econ. 20: 317-332. Doi: http://dx.doi.org/10.1016/j.jfe.2014.09.003

Kumar PD (2013) Village communities and their common property forests. Econ. Polit. Wkly 48(35): 33-36.

Lise W (2000) Factors influencing people's participation in forest management in India. Ecol. Econ. 34: 379-392. doi:http://dx.doi.org/10.1016/S0921-8009(00)00182-8

Mahanta R, Das D (2013) Attitudes Towards Biodiversity Conservation of Forests Dwellers and Encroachers: A Case Study of Assam in Northeast India. Small-scale For. 12: 307-319. doi:10.1007/s11842-012-9213-3

Matta J, Alavalapati J, Kerr J, Mercer E (2005) Agency perspectives on transition to participatory forest management: a case study from Tamil Nadu, India. Soc Nat Resour. 18: 859-870. doi:10.1080/08941920500248749

Mondal P, Southworth J (2010) Protection vs. commercial management: Spatial and temporal analysis of land cover changes in the tropical forests of Central India. For. Ecol. Manag. 259: 1009-1017. doi:http://dx.doi.org/10.1016/j.foreco.2009.12.007

Nagendra H (2009) Drivers of regrowth in South Asia's human impacted forests. Curr. Sci. 97: 1586-1592.

Nagendra H, Paul S, Pareeth S, Dutt S (2009) Landscapes of protection: forest change & fragmentation in Northern West Bengal, India. Environ manage 44: 853-864. doi:10.1007/s00267-009-9374-9

Nayak PK, Berkes F (2008) Politics of co-optation: community forest management versus joint forest management in Orissa, India. Environ Manage. 41: 707-718. doi: 10.1007/s00267-008-9088-4.

Ota M, Masuda M, Tani Y (2013) The Institutional Design and Effectiveness of Timber Benefit Sharing under Joint Forest Management in Madhya Pradesh, India. Small-scale For. 12: 215-234. doi:10.1007/s11842-012-9208-0

Prasad R, Kant S (2003) Institutions, forest management, and sustainable human development–experiences from India. Environ Dev Sustain 5: 353-367. doi:10.1023/A:1025772928833

Rawat PK, Tiwari PC, Pant CC (2012) Climate change accelerating land use dynamic & its environmental & socio-economic risks in the Himalayas: Mitigation through sustainable land use. Int J Clim Chang Str 4: 452-471. Doi:http://dx.doi.org/10.1108/17568691211277764

Rishi P (2007) Joint forest management in India: An attitudinal analysis of stakeholders. Resour Conserv Recycl. 51:345-354. doi:http://dx.doi.org/10.1016/j.resconrec.2006.10.009

Robbins P (2001) Tracking invasive land covers in India, or why our landscapes have never been modern. Ann Assoc Am Geogr. 91: 637-659. doi: 10.1111/0004-5608.00263

Singh RB, Mathur A (2001) Large-scale dams as drivers of land use/cover change in the tropical deciduous forests - A case-study of Sardar Sarovar Project (SSP), India, In Land Use & Cover Change, (Ed) Singh RB, Fox J, Himiyama Y, pp. 138-152.

Tiwari P (2008) Land use changes in Himalaya and their impacts on environment, society and economy: A study of the Lake Region in Kumaon Himalaya, India. Adv. Atmos. Sci. 25: 1029-1042. doi:10.1007/s00376-008-1029-x

Table S12. Hypothesized socioeconomic and biophysical factors (or their proxies) included in our analysis. The "Reference" column corresponds to individual case studies in our synthesis (see "Study #" in Tables S8-S11) based on which we grounded our hypothesis. See Table S15 for more detailed description of the variables that appears in the figures (showing standardized regression coefficients) presented in the results section.

**Broad clusters**	**Explanatory factor**	**Rationale**	**Reference**
Farm Characteristics	Average farm size	Evidence suggests two contrasting effects:<br><br>Larger farm size is positively related to fallow land as credits, labor, and water become limiting factors to expansion.<br><br>Small farm size is positively related to fallow land because of low productivity (uneconomical to mechanize).<br><br>There exist contrasting evidence to show both positive and negative relation between farm size and forest encroachment, when productivity of farms are low.	A2, A3, A6, A7, A8, A9, A11, A12, A14; B1, B5; C1, C6, C7, C8, C10, C11, C12, C14, C15, C19, C20; D8, D17
	Land Tenancy (Percent of leased-in area)	Higher land tenancy is expected to be negatively associated with fallow land, because it increases land access and provides stable livelihood to the poor.	A3, A13; B2, B5; C14
Demographic factors	Total human population density	Higher population pressure is expected to be negatively associated with fallow land due to increased land requirements.<br><br>Higher population pressure is expected to be positively associated with forest loss due to increased demand for natural resources.	A12; B12; C3, C6, C8, C11, C13, C14, C15, C17, C18, C22, C28, C33, C34, C37, C40, C42; D11
	Average number of heads per household (urban + rural)	Higher family size is expected to be positively associated with forest loss as it increases family labor availability to collect forest produce (e.g. fodder) even from distant locations. Forest loss/degradation would occur only if the forest harvest exceeds the sustainable yield.	C20; D8
	Urban population density	Evidence suggests two contrasting effects:	A16, A19;

	Proportion of urban population	Higher urban pressure is expected to be positively related to fallow land as it can bring new income opportunities, some of which can degrade land over time (e.g. brick kilns).<br><br>Higher urban pressure is expected to be negatively related to fallow land as it increases farmer's exposure to new technology and knowledge, cost advantage of transportation, adoption of high-yielding varieties, their quick sales and demand.<br><br>Higher urban pressure is expected to be positively related to forest loss due to increased demand for forest products (e.g. furniture's).	B5; C2, C15, C17, C34
	Female population density<br>Proportion of female population	Evidence suggests that male out-migration (for better income jobs than agriculture) will lead to higher fallow land, because out-migration of males increase the workload of females resulting in less efficient land management due to multi-tasking (on/off field activities) and lack of confidence and opportunity for long outdoor activities. Nonetheless, their participation and contribution to resource conservation has always been predominant throughout the country. Overall, we expect higher proportion of female population to be positively related to fallow land.<br><br>Higher proportion of female population is expected to be negatively related to forest loss because they are more dependent on forest for income (i.e. it generates greater family income, who otherwise would be unemployed). Dependency of forests generally makes them more sensitive to forest protection.	A9, A11; D5, D9, D14, D21, D23
Labor force	Working population density<br>Proportion of employed population<br>Marginal workers density<br>Proportion of marginal workers	Higher proportion of unemployed population (1 minus proportion of working population) is expected to be positively related to forest loss because it increases the economic dependence on forests and also increased availability/affordability of alternate energy sources including cooking fuel.<br><br>Higher proportion of marginal workers (<6 months/yr employed) is expected to be positively related to forest loss because they typically depend more on forests for income.	C9, C10, C14, C17, C42; D6, D8, D18

	Total cultivators density<br>Marginal cultivators density<br>Proportion of marginal cultivators<br>Total agricultural laborers density<br>Marginal agricultural laborers density<br>Proportion of marginal agricultural laborers<br>Female cultivators density<br>Proportion of female cultivators<br>Female marginal agricultural laborers density<br>Male marginal cultivators density<br>Proportion of male marginal cultivators<br>Male main agricultural laborers density<br>Proportion of main female cultivators<br>Proportion of main male agricultural laborers<br>Proportion of marginal female agricultural laborers	A cultivator is a person (family worker/single worker/employer) involved in cultivation of land owned or held from government or held from private persons or institutions for payment in money, kind or share of crop. A person who worked in another person's land for wages in cash, kind or share is an agricultural/wage laborer.<br><br>We expect lower cultivators per unit farm area to be positively associated with fallow land due to family labor shortage (typically due to migration of males to urban areas for off-farm jobs due to risk aversion attitude of farmers). Family labor shortage also provides less incentive to invest in soil-water management, furthering degradation.<br><br>We expect lower wage laborers per unit farm area to be positively associated with fallow land because labor shortage tends to increase wage rates which increase the cost of cultivation keeping land fallow.<br><br>We accounted for imperfect labor markets by including gender-wise variables. We also broadly accounted for income differences by splitting the variables as main and marginal workers. Marginal workers are those who had not worked for the major part of the reference period (i.e. less than 6 months).<br><br>We expected higher proportion of marginal workers and wage laborers will increase the pressure on forests, due to higher economic dependence on forests.	A1, A2, A6, A7, A8, A9, A10, A11, A13; B2, B12
	Density of community workers	Community workers include presence of governmental or non-governmental organization (NGO) that typically provides technical assistance and incentives (e.g. fertilizers) to agriculture. They also help with forest restoration efforts in collaboration with forest department and local communities, among others.<br><br>We expect higher density of community workers to be negatively associated with fallow land and forest loss, and positively associated with forest gain.	A17; B6, B7, B9, B13, B14; D15, D18

	Density of forestry workers	Forestry workers are people employed by forest department either on contract-basis or full-time employment. They are mainly involved in maintenance of forest, roads, wild life protection/census, wildlife watch, fire observation, manning of forest watch towers, interface with tourism, and extraction of grasses for army or other national use.<br><br>We expect forestry workers to be negatively associated with forest area loss and positively to forest area gain as they are a proxy for level of protection.	D13
	Industrial & Construction workers density	We expect industrial and construction workers density to be positively associated to forest loss due to two reasons. First, the variable is a proxy for the intensity of forest conversion to built-up areas. Second, the worker density is also a proxy for the pressure exerted on forests due to their dependence on forest for domestic purposes.	C2, C4, C11, C17, C26, C27, C31, C36, C37, C38, C39, C41, C42; D3
	Mining/Quarrying worker density	We expect mining and quarrying worker density to be positively associated with forest loss due to direct forest conversions for the mining/quarrying activity and indirectly due to dependence of the worker community on forests for domestic purposes.	A15, A16, A20; B3; C2, C8, C21, C29, C30, C42
Level of Education	Illiterate population density<br>Proportion of literate population<br>Female literate population density<br>Proportion of female population that is literate<br>Number of educational facilities	Evidence suggests two contrasting effects:<br><br>Higher education level and exposure is negatively associated with fallow land because farmers make better investment decision, better adapt to new technology, and soil-water conservation.<br><br>Higher education level and exposure is positively associated with fallow land because the literates tend to prefer off-farm jobs that provide higher and more stable income. The off-farm job typically is through urban migration (in which case causes family labor shortage converting land fallow) or by converting the farm land for other high-income purposes (e.g. brick kilns to serve near-by markets).<br><br>With higher education levels farmers also perceive higher returns to investment on land. The effect of this perception on fallow land is unclear because. With higher perceived returns famers tend to use	A6, A11; B1, B4, B5, B10, B13, B16; C35; D2, D5, D8, D9, D14, D16, D17, D18
Exposure	Access to information		A9, A10, A11; B1, B4, B5, B10, B12, B13, B16; D2, D5, D6, D8, D9, D14, D16, D17, D18, D20

		the land more effectively reducing fallow land. Concurrently, higher perceived returns tend to reduce their chances of meeting the expected returns that can lead to abandoning the land for better income opportunities.<br><br>Higher education level and exposure is expected to be negatively associated with forest loss (or positively to forest gain) because the population is more aware of the long-term benefits of forest protection. Some studies have contrastingly suggested that higher literacy rates increase the pressure on neighboring forests to meet development needs furthering forest loss.	
Irrigation Infrastructure	Proportion of cropland irrigated<br>Proportion of area irrigated by government canal<br>Proportion of area irrigated by private canal<br>Proportion of area irrigated by well without electricity<br>Proportion of area irrigated by well with electricity<br>Proportion of area irrigated by tube well without electricity<br>Proportion of area irrigated by tube well with electricity<br>Proportion of area irrigated by tanks<br>Proportion of area irrigated by rivers<br>Proportion of area irrigated by lakes<br>Proportion of crop area irrigated by other means<br>Availability of well irrigation with electricity<br>Availability of tube well	We expect extension of irrigation facility to be negatively associated with fallow land in small farms. In Agro-Ecological Zones with large farms with low productivity, we expect irrigation extension to increase fallow land as it typically leads to directing more concentrated efforts on the irrigated area at the expense of other areas (in large). Along the same lines, some studies have also suggested that improvement (shift) in irrigation facility (from less stable to more stable and reliable irrigation source) also can increase fallow land due to concentrated efforts in small areas.<br><br>We expect extension of irrigation facility to be negatively associated with forest loss, as it reduces the economic dependence of farmers on neighboring forests. At the same time, extension of irrigation infrastructure (e.g. dam construction) often leads to inundation of forest areas that can lead to forest loss. In such cases, we also tested for a cascading effect where government does compensatory afforestation (forest gain) by planting the uprooted trees or forest plantations in neighboring regions.	A1, A2, A3, A4, A5, A6, A9, A13, A14, A17; B2, B3, B5, B6, B7, B8, B9, B10, B11, B14, B15, B16; C8, C11, C12, C20, C38, C39, C41; D3

	irrigation with electricity		
	Availability of irrigation facility		
	Availability of well irrigation without electricity		
	Availability of tube well irrigation without electricity		
	Availability of tank irrigation		
Infrastructure (Electricity)	Availability of power supply for domestic purpose	We expect provision of electricity supply for domestic purpose to be negatively associated with forest loss as it will reduce the dependence on forest for firewood and building materials (especially during cold seasons).	C1, C6, C7, C8, C9, C10, C11, C12, C14, C18, C20, C23, C30, C37
	Availability of power supply for agriculture	We expect provision of electricity supply for domestic purpose to be negatively associated with fallow land as it allows use of modern equipment's for agriculture. The reliability (erratic) of power source is also an important factor that we do not account for due to lack of data.	A21
Poverty Indicators	Average income per capita	We expect lower income per capita to increase fallow land as it reduces the ability to invest in land owing to capital-intensive nature for adoption modern outputs (acquire assets and equipment's), and to cope with agricultural crisis.<br><br>We included scheduled caste and scheduled tribe populations as they are economically weaker sections of the community.<br><br>Evidence suggests two contrasting effects:<br><br>Lower per capita income are positively associated with forest loss as they are dependent on forest for livelihood and subsistence (when extraction exceeds sustainable yields), conditional of lack of other alternatives for livelihood.<br><br>Lower per capita income is negatively associated with forest loss for two reasons. First, they tend to protect forests because forests are important for their livelihood (attitude). Tribal population (proxy for	A2, A4, A6, A8, A9, A11; B1, B2, B4, B5; C1, C3, C6, C7, C8, C9, C10, C12, C13, C14, C18, C19, C20, C23, C24, C25, C32, C37
	Tribal population density		A9, A11, A12, A18; B1, B2, B5; C14, C20, C23, C4, C24, C25, C32, C37; D17
	Backward caste population (scheduled caste + tribe) density		
	Proportion of tribal population		
	Proportion of backward caste population		

		low income groups) is culturally linked to forests and they are typically motivated by state forest department to jointly manage forest through protection, restoration of degraded forest, and enrichment plantations.	
Accessibility (Navigation & Irrigation source)	Availability of market facility in the village/	Evidence suggests two contrasting effects: (rural infrastructure)<br><br>Improved accessibility is negatively associated with fallow land as it improves the capital investment capacity of the household, exposure to use of new technology and knowledge, provides cost advantage of transportation of high value crops, their quick sales, and increased demand.<br>Improved accessibility is positively associated with fallow land as it creases rural employment diversification due to new income opportunities (e.g. brick production, dairy industry) that can lead to conversion of cropland to fallow land among others.<br><br>Based on case-study evidence, we expect improved connectivity to be positively associated with forest loss due to increasing demand for forest products, and ease of illegal timber logging and transport.	A8, A9, A10, A15, A16, A19, A21; B3, B5, B6, B12; C1, C6, C9, C15, C16, C34, C37, C40; D11
	Market functioning frequency		
	Distance to nearest town		
	Availability of communication facility (e.g. bus, train)		
	Approach to pucca road		
	Approach to kachcha road		
	Approach to foot path		
	Approach to river		
	Approach to canal		
	Approach to waterways		
Critical support services	Number of agricultural credit societies/institutions	Overall, we expect access to agricultural credit society to be negatively associated with fallow land as it drives the capital availability to invest in farming. However, the access to capital depends on asset base of the farmer, so may not benefit all farmers equally.	A2, A11, A14, A17, A21; B1, B5; C8;
	Distance to nearest agricultural credit society/institution		
	Number of veterinary medical facilities	We expect access to veterinary medical facilities to be negatively associated with fallow land as it reduces oxen/livestock mortality rate, an key component of non-mechanized agriculture in India.	A8, A11, A21
Income dependency: Binary variables coded to indicate primary	Building/mining Materials	We expect extraction of building (e.g. mud for bricks) and mining (e.g. coal) materials will be positively associated to forest loss and fallow land due to direct land conversions to conduct the activity.	A15, A19, A20; C2, C17, C21, C29, C30, C42
	Dairy/cattle/leather	We expect that livestock-based activities will be positively associated with forest loss due to increased chances of overgrazing and fodder collection.	C8, C12
	Wool/Woolen Blankets		
	Poultry		

occupations of each village.	Coffee Production	We tested if villages dependent on specific plantations are prone to forest loss due to legal land conversions or illegal land encroachments.	C28, C31
	Tea production		C14, C17, C28
	Coconut Production		C31
	Rubber production		C14
	Forestry-related Products	We tested if villages dependent on forestry products for primary income are prone to forest loss due to over-extraction above sustainable yields.<br><br>Forestry products includes but not limited to extraction of tendu leafs, making agarbathis/incense sticks, baskets match sticks, brooms, beedi/cigarettes, paper products, and gutka.	C7, C9, C10, C11, C13, C19, C23, C25, C27, C28
	Making of Wooden Furniture's/timber	We expect a positive association between wood furniture/timber making villages and forest loss.	C6, C17, C18
	Manufacturing of wooden agricultural implements	We expect a positive association between villages making wooden agricultural implements and forest loss.	C1
	Prawn harvesting	We tested if forest loss is positively associated to prawn harvesting, specifically due to conversion of mangrove forests to aquaculture farms.	C40
	80+ other binary-coded variables to capture the other common primary occupations in India relevant to the three land-cover conversions investigated in this analysis.	Example: We included several crops (including Arecanut, Cotton, Rice, Wheat, Sugarcane, Bajra, Jowar, Pulse, Maize, Pigeon Peas, and Groundnut) to test if any crop-specific village occupation is prone to more forest encroachment.	-
Climate	Average seasonal temperature; Four variables: $T_{winter\ (avg)}$, $T_{pre\text{-}monsoon\ (avg)}$, $T_{sw\ monsoon\ (avg)}$, $T_{post\text{-}monsoon\ (avg)}$.	See Table S14 for an explanation of these variables.<br><br>Both temperature and rainfall are important factors influencing crop cover. Therefore, we tested for key seasonal variables to account for the impacts of climate change and variability on cropland conversion (to and from fallow land). We use squared variables to test for nonlinear effects of climate on cropland conversions.<br><br>As we estimated LULCC from decadal Landsat imageries, they capture only the decadal changes in LULCC, and can mask within-decade variations in LULCC. Especially, inter-annual climate	A2, A4; B3
	Average seasonal precipitation; Four variables: $P_{winter\ (avg)}$, $P_{pre\text{-}monsoon\ (avg)}$, $P_{sw\ monsoon\ (avg)}$, $P_{post\text{-}monsoon\ (avg)}$.		
	Squared average seasonal temperature; Four variables: $T^2_{winter\ (avg)}$, $T^2_{pre\text{-}monsoon\ (avg)}$, T^2_{sw}		

	$_{\text{monsoon (avg)}}$, $T^2_{\text{post-monsoon (avg)}}$	variability causes fluctuations in fallow land. However, the conversions between cropland and fallow inferred between decadal end points will reflect only the climate-effect of end point. Therefore, we used 1994-1995 climate data to study cropland ↔ fallow conversions during 1985-1995 decade, and 2004-2005 climate data to study cropland ↔ fallow conversions during 1995-2005 decade.	
	Squared average seasonal precipitation; Four variables: $P^2_{\text{winter (avg)}}$, $P^2_{\text{pre-monsoon (avg)}}$, $P^2_{\text{sw monsoon (avg)}}$, $P^2_{\text{post-monsoon (avg)}}$		
	Variation in average seasonal precipitation relative to long-term normal; Four variables: $P_{\text{winter (rel. to normal)}}$, $P_{\text{pre-monsoon (rel. to normal)}}$, $P_{\text{sw monsoon (rel. to normal)}}$, $P_{\text{post-monsoon (rel. to normal)}}$		A2, A4, A7, A8, A9
	Standard deviation in daily seasonal precipitation; Four variables: $P_{\text{winter (std)}}$, $P_{\text{pre-monsoon (std)}}$, $P_{\text{sw monsoon (std)}}$, $P_{\text{post-monsoon (std)}}$		A2, A4, A7, A8, A9
	Annual Mean Temperature	We used key static bioclimatic variables to test for the effects of passive forces (e.g. climate-driven shifts in natural vegetation) on forest area change.<br><br>We also hypothesized that colder and wetter regions (especially those with low village infrastructure) will be positively associated with forest loss due to increased dependence on forests for fuel wood and building materials.	C3, C7, C9, C14, C15, C18, C19, C20, C23, C24, C32, C37; D7, D10
	Mean Temperature of Wettest Quarter		
	Mean Temperature of Warmest Quarter		
	Mean Temperature of Coldest Quarter		
	Max Temperature of Warmest Month		
	Min Temperature of Coldest Month		
	Annual Precipitation		
	Precipitation of Wettest Quarter		
	Precipitation of Coldest Quarter		
	Precipitation of Driest Quarter		
	Precipitation of Warmest Quarter		
Edaphic Condition	Cation Exchange Capacity (proxy for soil fertility)	These factors determine the soil quality and extent of various forms of soil degradation, both crucial in determining crop and forest	A9, A12; B5, B11, B16; C3,

	Soil erosion<br>Soil depth<br>Soil salinity<br>Slope<br>Soil flooding	productivity.<br><br>We expect increased level of soil degradation to be positively related to fallow land and forest area loss. The degradation may result from both natural and anthropogenic factors (e.g. sub-standardized construction of roads, overuse of fertilizers).	C8, C14, C15, C20, C23, C28, C32, C34; D7
Elevation	Terrain	We expect positive association between topography and fallow land because cropland management is expected to be more suited to flat terrain or in areas with gentle slopes, also conducive to the construction of houses and infrastructure.<br><br>We expect negative association between topography and forest loss because rougher terrains are more difficult to access.	B12; C16, C26, C31, C32, C34; D7, D11
Political boundaries	State dummies/State-fixed effects	See detailed explanation provided in Text S1 (sub-section titled “Rationale for inclusion of state-fixed effects”).	A8; C6, C8, C11, C12, C13, C17, C19, C20, C31, C33; D1, D2, D3, D5, D6, D8, D13, D15, D16, D17, D18, D19, D20, D22, D23
Other variables	Protected areas	We expect negative association between protected areas and forest loss (and positively associated with forest gain). Protected areas however render no information of the level of protection. We included “density of forestry workers” as a variable proxy for the level of protection and control.	C1, C6, C8, C9, C10, C11, C12, C13, C17, C20, C23, C24, C25, C26, C27, C28, C32, C37, C40; D1, D11, D12
	Mined-out areas	We expect positive association between mined-out areas and forest area gain due to compensatory afforestation efforts by government	

		on mined-out areas to compensate for the forest loss. We expected a weak association because case studies suggest that the forest plantations do not survive over time in many cases, due to high levels of soil degradation caused from mining.	
	Sacred groves	We expect positive association between sacred forest groves and forest area gain as the forests are typically protected by local community due to cultural and religious beliefs (however, not in all cases).	D6
	Cattle density	We expect positive association between cattle density and forest loss because cattle's increases the animal pressure on forest from grazing. Due to data limitations, a static map (circa 2006) was used for land-conversion analysis during both decades. This is a minor concession as we are interested in the spatial variations of independent variables in our spatial regression model, rather than their absolute magnitude (note that we standardized all explanatory variables using *z*-score prior to running the regressions).	C1, C3, C5, C6, C7, C8, C9, C10, C11, C14, C19, C20, C22, C24, C25, C27

Table S13. Summary of various input datasets used in this study. Geographic Information System abbreviated as GIS. All are national database, and brought to 1km x 1km resolution for this analysis.

Data Code	Data	Spatial Resolution	Temporal Resolution/Coverage	Remarks
**Survey (Tabular data)**				
1	Over 200 socioeconomic variables from 'primary census abstract' and 'village directory'	Village level (~630,000 units)	Two census years (1991, 2001)	Tabular data: http://censusindia.gov.in/ This study ties the tabular data to village-level administrative boundaries.
**Remote Sensing (all data included ground surveys for interpretation and validation)**				
2	Land cover (Landsat MSS/TM)	30m (1:50k scale)	1985, 1995, 2005	Roy et al. 2015a
3	Sacred groves	23.5m (1:50k scale)	Circa 2005	Roy et al. 2015b; Satellite data interpreted using field maps from each state of India. Sacred groves typically are preserved over many decades.
4	Soil	Resampled to 1km in GIS (1:250k scale)	Static (1980-2001)	National Bureau of Soil Survey and Land Use Planning, India (NBSS&LUP 2002)
5	Mined-out areas	30m (1:50k scale)	1985, 1995, 2005	Roy et al. 2015, a; Variable culled from Level III classification.
6	Terrain	30m	Circa 2000	SRTM (http://glcf.umd.edu/data/srtm/); Gap filled using 10m CARTOSAT data sampled to 90m (Muralikrishnan et al. 2013).
7	Protected areas	Resampled to 1km (1:50k scale)	Two periods (1990s and 2000s)	Combination of data from natural features, GPS points, and inputs from states of India (WII 2012).
**Climate data (Gridded from observations)**				
8	Rainfall	Resampled to 1km in GIS (0.25°x0.25° lat/long)	Daily (1901-2014)	Pai et al. 2014; Gridded from ~7000 rain gauge stations (most comprehensive for India)
9	Temperature	Resampled to 1km in GIS (0.25°x0.25° lat/long)	Daily 1961-2007	APHRODITE (NCARS 2014)
10	Bioclimatic variables	1km x 1km	Static (contemporary conditions)	19 variables from Hijmans et al. 2005

Ancillary data				
11	Village/town boundaries of India	Resampled to 1km in GIS (1:10k scale or finer)	2001	Hard-copy maps: Survey of India Digital version: This study
12	State boundaries of India (for state dummies)	Resampled to 1km in GIS (1:250k scale)	Two census years (1991 and 2001)	Official data from Survey of India.
13	Agro-Ecological Zones of India	Resampled to 1km in GIS (1:250k scale)	Static (contemporary conditions)	Gajbhiye and Mandal (2000)
14	Cattle population density	1km x 1km	Static (circa 2006)	Robinson et al. 2014

WII 2012, Data provided by Wildlife Institute of India (2012).

Downloaded from http://glcf.umd.edu/data/srtm/

Gajbhiye KS, Mandal C (2000) Agro-ecological zones, their soil resource and cropping systems. Status of Farm Mechanization in India, Cropping Systems, Status of Farm Mechanization in India, pp 1-32.

Hijmans RJ, Cameron SE, Parra JL, Jones PG, Jarvis A (2005) Very high resolution interpolated climate surfaces for global land areas. Int J clim. 25:1965-1978. doi: 10.1002/joc.1276

Muralikrishnan S, Pillai A, Narender B, Reddy S, Venkataraman VR, Dadhwal VK (2013). Validation of Indian national DEM from Cartosat-1 data. J Indian Soc Remote Sens. 41:1-13. doi:10.1007/s12524-012-0212-9

National Center for Atmospheric Research Staff (Eds). Last modified 05 May 2014. "The Climate Data Guide: APHRODITE: Asian Precipitation - Highly-Resolved Observational Data Integration Towards Evaluation of Water Resources." Retrieved from https://climatedataguide.ucar.edu/climate-data/aphrodite-asian-precipitation-highly-resolved-observational-data-integration-towards

NBSS&LUP, Soils of India, NBSS Publ. No. 94, National Bureau of Soil Survey & Land Use Planning, Nagpur, 2002, pp. 130 + 11 sheet maps.

Pai DS, Sridhar L, Rajeevan M, Sreejith OP, Satbhai NS, Mukhopadhyay B (2014) Development of a new high spatial resolution (0.25× 0.25) long period (1901–2010) daily gridded rainfall data set over India and its comparison with existing data sets over the region. Mausam, 65:1-18.

Robinson TP, Wint GW, Conchedda G, Van Boeckel TP, Ercoli V, Palamara E, Cinardi G, Aietti LD, Hay SI, Gilbert M (2014) Mapping the global distribution of livestock. PLoS ONE. http://dx.doi.org/10.1371/journal.pone.0096084.

Roy PS, Behera MD, Murthy MSR, Roy A, Singh S, Kushwaha SPS, Jha CS, Sudhakar S, Joshi PK, Reddy CS, Gupta S, Pujar G, Dutt CBS, Srivastava VK, Porwal MC, Tripathi P, Singh JS, Chitale V , Skidmore AK, Rajshekhar G, Kushwaha D, Karnataka H, Saran S, Giriraj A, Padalia H, Kale M, Nandy S, Jaganathan C, Singh CP, Chandrashekhar MB, Pattanaik C, Singh DK, Devagiri GM, Talukdar G, Panigrahy RK, Singh H, Sharma JR, Haridasan K, Trivedi S, Singh KP, Kannan L, Daniel M, Misra MK, Niphadkar M, Nagbhatla N, Prasad N, Tripathi OP, Prasad PRC, Dash P, Qureshi Q, Tripathi SK, Ramesh BR, Gowda B, Tomar S, Romshoo S, Giriraj S, Ravan SA, Behera SK, Paul S, Das AK, Ranganath BK, Singh TP, Sahu TR, Shankar U, Menon ARR, Srivastava G, Neeti, Sharma S, Mohapatra U B, Peddi A, Rashid H, Salroo I, Krishna PH, Hajra PK, Vergheese AO, Matin S, Chaudhry SA, Ghosh S, Lakshmi U, Rawat D, Ambastha K, Kalpana P, Devi BSS, Gowda B, Sharma KC, Mukharjee P, Sharma A, Davidar P, Raju RR, Ketewa SS, Kant S, Raju VS, Uniyal BP, Debnath B, Rout DK, Thapa R, Joseph S, Chhetri P and Ramachandran RM (2015,b) New vegetation type map of India prepared using satellite remote sensing: Comparison with global vegetation maps and utilities. Int J Appl Earth Obs Geoinformation 39:142-159. doi:http://dx.doi.org/10.1016/j.jag.2015.03.003

Roy PS, Roy A, Joshi PK, Kale MP, Srivastava VK, Srivastava SK, Dwevidi RS, Joshi C, Behera MD, Meiyappan P , Sharma Y, Jain AK, Singh JS, Palchowdhuri Y, Ramachandran RM, Pinjarla B, Chakravarthi V, Babu N, Gowsalya MS, Thiruvengadam P, Kotteeswaran M, Priya V, Yelishetty KMVN, Maithani S, Talukdar G, Mondal I, Rajan KS, Narendra PS, Biswal S, Chakraborty A, Padalia H, Chavan M, Pardeshi SN, Chaudhari SA, Anand A, Vyas A, Reddy MK, Ramalingam M, Manonmani R, Behera P, Das P, Tripathi P, Matin S, Khan ML, Tripathi OP, Deka J, Kumar P, Kushwaha D (2015,a) Development of Decadal (1985–1995–2005) Land Use and Land Cover Database for India. Remote Sens. 7:2401-2430. doi:10.3390/rs70302401

Table S14. List of model simulations with key model parameters. The 'elastic-net' parameters correspond to the 'best model' i.e. the model from *k*-fold cross-validation with maximum percent of null deviance explained (see Fig. S28 for an example). The hypothesized explanatory variables are provided in Table S12.

**Land-cover conversion**	**Spatial estimation**	**Time**	**Buffer area (km)**	**Elastic-net parameters**		**Results**		**Sim #**
				α	$Log(\lambda)$	**Figure**	**% (of null) deviance explained**	
Cropland → Fallow land	National	1985-1995	6.6	0.8	-8.38	Fig. S5a	0.85	1
		1995-2005	7.3	0.9	-9.72	Fig. 3a	0.90	2
	AEZ2	1985-1995	1.5	0.6	-10.08	Fig. S7a	0.95	3
		1995-2005	7.4	0.8	-4.59	Fig. S7b	0.97	4
	AEZ8	1985-1995	5.2	0.7	-6.80	Fig. S8a	0.86	5
		1995-2005	1.2	0.6	-5.82	Fig. S8b	0.75	6
	AEZ4	1985-1995	2.6	0.7	-9.60	Fig. S9	0.75	7
	AEZ5	1995-2005	4.6	0.6	-7.02	Fig. S10	0.78	8
Fallow land → Cropland	National	1985-1995	7.7	0.8	-10.37	Fig. S5b	0.94	9
		1995-2005	7.7	0.7	-5.27	Fig. 3b	0.78	10
	AEZ8	1985-1995	1.7	0.8	-5.85	Fig. S11a	0.93	11
		1995-2005	7.8	0.6	-5.02	Fig. S11b	0.78	12
	AEZ6	1985-1995	7.7	0.8	-4.95	Fig. S12	0.96	13
	AEZ2	1995-2005	4.1	0.7	-10.09	Fig. S13	0.80	14
Gross forest area loss	National	1985-1995	6.5	0.8	-8.06	Fig. S15	0.76	15
		1995-2005	1.6	0.8	-7.85	Fig. 4a	0.78	16
	AEZ10	1985-1995	3.7	0.8	-5.01	Fig. S17a	0.88	17
		1995-2005	7.4	0.7	-9.97	Fig. S17b	0.84	18
	AEZ12	1985-1995	6.4	0.6	-8.35	Fig. S18a	0.80	19
		1995-2005	7.7	0.7	-6.46	Fig.S18b	0.93	20
	AEZ5	1995-2005	0.8	0.6	-6.81	Fig. S20	0.86	21

	AEZ19	1985-1995	5.4	0.9	-7.59	Fig. S19	0.87	22
	AEZ14	1995-2005	6.9	0.8	-4.53	Fig. S21	0.91	23
Gross forest area gain	National	1985-1995	7.5	0.8	-5.68	Fig. S22	0.79	24
		1995-2005	5.6	0.9	-4.86	Fig. 4b	0.91	25
	AEZ5	1985-1995	6.2	0.6	-5.29	Fig. S23a	0.91	26
		1995-2005	6.1	0.7	-5.68	Fig. S23b	0.81	27
	AEZ12	1985-1995	3.4	0.6	-6.92	Fig. S24	0.86	28
	AEZ14	1985-1995	5.4	0.9	-4.35	0.37 (low explanatory power)		29
	AEZ4	1995-2005	1.8	0.6	-10.32	Fig. S25	0.72	30
	AEZ10	1995-2005	5.8	0.8	-10.61	Fig. S26	0.85	31

Table S15. Description of all variables that features in at least one of the figures in the results section presenting standardized regression coefficients.

**Broad category**	**Explanatory variable name**	**Description**	**Variable type**	**Data source code**
Farm Characteristics	Average farm size	The average size of a farm in each 1km grid cell	Continuous	1
Demographic factors	Proportion of female population	Ratio of female population to total (male + female) population	Continuous	1
Labor force	Proportion of marginal agricultural laborers	Ratio of marginal agricultural laborers to total (main + marginal) agricultural laborers. Includes both male and female agricultural laborers. A person who worked in another person's land for wages in cash, kind or share was regarded as an agricultural laborer. Such a person had no risk in cultivation but merely worked in another person's land for wages. An agricultural laborer had no right of lease or contract on land on which he worked.	Continuous	1
	Proportion of female cultivators	Ratio of female cultivators to total (male + female) cultivators. Includes both main (>6 months employment) and marginal (<6 months employment) cultivators. A cultivator if a person engaged either as employer, single worker or family worker in cultivation of land owned or held from government of held from private persons or institutions for payment in money, kind or share of crop. Cultivation included supervision or direction of cultivation. A person who had given out his/her land to another person or persons for cultivation or money, kind or share of crop and who did not even supervise or direct cultivation of land was not treated as cultivator.	Continuous	1
	Proportion of main female cultivators	Ratio of main female cultivators to total (main + marginal) female cultivators. Terminology: Marginal (<6 months employment), and Main (>6 months employment).	Continuous	1

	Male main agricultural laborers density	Density male agricultural laborers in each grid cell who has worked for more than 6 months a year (main worker).	Continuous	1
	Male marginal cultivators density	Density male cultivators in each grid cell who has worked for less than 6 months a year (marginal worker).	Continuous	1
	Density of community workers	Density of community workers in each grid cell. Community workers can include health workers, presence of governmental or non-governmental organization (NGO) that helps with restoration efforts in collaboration with forest department and local communities, among others. They also provide technical assistance in agriculture.	Continuous	1
	Density of forestry workers	Density of forestry workers in each grid cell. People who are employed by forest department either on contract-basis or full-time employment. They are mainly involved in maintenance of forest, roads, wild life protection/census, wildlife watch, fire observation, manning of forest watch towers, interface with tourism, and extraction of grasses for army or other national use. They also collect tendu leafs and other minor forest produce (for government agencies). Lastly, they are also involved in working plan preparation.	Continuous	1
	Industrial & Construction worker density	Density of workers employed in manufacturing and, building and construction industry in each grid cell.	Continuous	1
	Mining/Quarrying worker density	Density of workers employed in mining or quarrying activities in each grid cell.	Continuous	1
Level of Education	Illiterate population density	Density of illiterate population (> 6 years old) in each grid cell. Includes both male and female.	Continuous	1
	Proportion of literate population	Ratio of literate population to total (literate + illiterate) population above 6 years old. Includes both male and female.	Continuous	1
	Access to information	Binary variable indicating access to newspaper, magazines, etc.	Categorical	1

Irrigation Infrastructure	Proportion of cropland irrigated	Ratio of irrigated cropland area to total cropland area. Includes all types of irrigation.	Continuous	1
	Proportion of area irrigated by govt canal	Ratio of cropland area irrigated by government canal to total irrigated area.	Continuous	1
	Proportion of area irrigated by well w/o elec	Ratio of cropland area irrigated by well without electricity to total irrigated area.	Continuous	1
	Proportion of area irrigated by well w elec	Ratio of cropland area irrigated by well with electricity to total irrigated area.	Continuous	1
	Proportion of area irrigated by tube well w/o elec	Ratio of cropland area irrigated by tube well without electricity to total irrigated area.	Continuous	1
	Proportion of area irrigated by tube well w elec	Ratio of cropland area irrigated by tube well with electricity to total irrigated area.	Continuous	1
	Availability of well irrigation w elec	Binary variable indicating the presence or absence of well irrigation with electricity.	Categorical	1
	Availability of tube well irrigation w elec	Binary variable indicating the presence or absence of tube well irrigation with electricity.	Categorical	1
	Availability of irrigation facility	Binary variable indicating the presence or absence of any type of irrigation facility.	Categorical	1
Infrastructure (Electricity)	Availability of power supply for domestic purpose	Binary variable indicating the presence or absence of power supply for domestic use.	Categorical	1
	Availability of power supply for agriculture	Binary variable indicating the presence or absence of power supply for agriculture use.	Categorical	1
Poverty Indicators	Average income per capita	Average income of a person in the village. Includes unemployed population in the person count.	Continuous	1
	Proportion of tribal population	Ratio of scheduled tribe population to total population.	Continuous	1
	Proportion of backward caste population	Ratio of (scheduled tribe + schedule caste) population to total population. Both scheduled caste and tribes are considered lower caste and economically weak.	Continuous	1
Accessibility (Navigation and/or Irrigation source)	Distance to town	Distance to nearest town. There are about 5160 towns in India and ~630,000 villages.	Continuous	1
	Market frequency	Dummy-coded variable indicating the frequency of markets (daily, weekly, fortnightly, no markets). The category “no markets” is used as reference	Ordinal	1

		category.		
	Availability of communication facility	Binary variable indicating availability of bus stand train stations, etc. that connects to nearby towns.	Categorical	1
	Approach to *pucca* road	Binary variable indicating access to *pucca* road in the village. *Pucca* road is a black-topped road (all weather roads).	Categorical	1
	Approach to river	Binary variable indicating access to river in the village.	Categorical	1
	Approach to canal	Binary variable indicating access to government canal in the village.	Categorical	1
Critical support services	Number of agricultural credit societies	Total number of agricultural credit societies present in the village.	Continuous	1
	Distance to nearest agricultural credit society	Distance to the nearest agricultural credit society (the nearest credit society may be in the same village or nearby villages).	Continuous	1
Income dependency (Primary Occupation)	Occupation (Building/mining Materials)	Binary variable indicating if building (includes brick kilns, marble, granite) and/or mining (primarily stone and coal) as one of the top three primary occupations of the village.	Categorical	1
	Occupation (Dairy/cattle/leather)	Binary variable indicating if primary occupation related to raising livestock such as cattle, goat, and sheep and making related products including dairy (milk, ghee), and leather.	Categorical	1
	Occupation (Coffee)	Binary variable indicating if primary occupation is coffee growing.	Categorical	1
	Occupation (Coconut Production)	Binary variable indicating if primary occupation is coconut plantations.	Categorical	1
	Occupation (Forestry Products)	Binary variable indicating if primary occupation is related to forestry products. Forestry products includes but not limited to extraction of tendu leafs, making agarbathis/incense sticks, baskets match sticks, brooms, beedi/cigarettes, paper products, and gutka.	Categorical	1
	Occupation (Wool/Woolen Blankets)	Binary variable indicating if primary occupation is related to sheep shearing or making woolen	Categorical	1

		products.		
	Occupation (Bamboo Products)	Binary variable indicating if primary occupation is related to bamboo production and related products.	Categorical	1
	Occupation (Wooden Furniture/timber)	Binary variable indicating if primary occupation is related to making wooden furnitures (e.g. chair) or timber extraction.	Categorical	1
	Occupation (Wooden Agricultural implements)	Binary variable indicating if primary occupation is related to manufacturing wooden equipment's used for agriculture including axe, carts, and wooden ploughs.	Categorical	1
Climate	$T_{\text{sw monsoon (avg)}}$	Temperature in each grid cell averaged over the southwest monsoon season (June-September; 'Rabi' season).	Continuous	9
	$T_{\text{post-monsoon (avg)}}$	Temperature in each grid cell averaged over the post-monsoon season (October-November).	Continuous	9
	$T^2_{\text{post-monsoon (avg)}}$	Squared temperature in each grid cell averaged over the post-monsoon season. Squared values are used to account for non-linear response of climate to crop cover.	Continuous	9
	$P_{\text{sw monsoon (avg)}}$	Precipitation in each grid cell averaged over the southwest monsoon season (June-September; 'Rabi' season).	Continuous	8
	$P_{\text{post-monsoon (avg)}}$	Precipitation in each grid cell averaged over the post-monsoon season (October-November).	Continuous	8
	$P^2_{\text{sw monsoon (avg)}}$	Squared precipitation in each grid cell averaged over the southwest monsoon season. Squared values are used to account for non-linear response of climate to crop cover.	Continuous	8
	$P_{\text{sw monsoon (rel to normal)}}$	Average southwest monsoon precipitation over a given time period relative (minus) to the long-term (1961-2005) average.	Continuous	8
	$P_{\text{post-monsoon (rel to normal)}}$	Average post monsoon precipitation over a given time period relative (minus) to the long-term (1961-2005) average.	Continuous	8
	$P_{\text{sw monsoon (std)}}$	Standard deviation in daily precipitation amounts in	Continuous	8

		each grid cell during the southwest monsoon season.		
	$P_{post\text{-}monsoon\ (std)}$	Standard deviation in daily precipitation amounts in each grid cell during the post-monsoon monsoon season.	Continuous	8
	Annual Mean Temperature	Variable name is self-explanatory.	Continuous	10
	Mean Temperature of Wettest Quarter	Variable name is self-explanatory.	Continuous	10
	Annual Precipitation	Variable name is self-explanatory.	Continuous	10
	Precipitation of Wettest Month	Variable name is self-explanatory.	Continuous	10
	Precipitation of Wettest Quarter	Variable name is self-explanatory.	Continuous	10
	Precipitation of Coldest Quarter	Variable name is self-explanatory.	Continuous	10
Edaphic Condition	Cation Exchange Capacity	Dummy-coded variable indicating the level of cation exchange capacity (CEE) of soil (<10 cmol/kg, 10-20 cmol/kg, 20-30 cmol/kg, and >30 cmol/kg). The level '<10 cmol/kg' is used as reference category. CEE is an indicator of soil fertility.	Ordinal	4
	Soil erosion	Dummy-coded variable indicating the level of soil erosion (None to very slight, Slight, Moderate, Severe, Very severe). The level 'None to very slight' is used as reference category.	Ordinal	4
	Soil Depth	Dummy-coded variable indicating the level of soil depth (Extremely shallow, Very shallow, Shallow, Moderately Shallow, Moderately Deep, Deep, Very deep). The level 'Extremely shallow' is used as reference category.	Ordinal	4
	Soil salinity	Dummy-coded variable indicating the level of soil salinity (Negligible (1-2 dS/m), Slight (2-4 dS/m), Moderate (4-8 dS/m), Moderate strong (8-15 dS/m), Strong (15-25 dS/m), Severe (25-50 dS/m), Very severe (>50 dS/m)). The level 'Negligible (1-2 dS/m)' is used as reference category.	Ordinal	4
	Slope	Dummy-coded variable indicating the slope level (Level to nearly level (0-1%), Very gentle (1-3%), Gentle (3-8%), Moderate (8-15%), Moderately	Ordinal	4

		steep (15-30%), Steep (30-50%), Very steep (>50%)). The level ‘Level to nearly level (0-1%)’ is used as reference category.		
Terrain/Slope	Terrain	Average elevation of land in each grid cell.	Continuous	6
Political boundaries	State dummies	Dummy-coded variable indicating which state administrative division each grid cell belongs to. We fix one state as the reference category relative (abbreviation: ‘rel’) to which we evaluate the effects.<br><br>The following state names have been abbreviated as follows: MP (Madhya Pradesh), AP (Andhra Pradesh), UP (Uttar Pradesh).	Categorical	12
Other variables	Protected areas	Protected areas (national parks and wildlife sanctuaries) in each grid cell.	Continuous	7
	Mined-out areas	Area that was mined in each grid cell.	Continuous	5
	Sacred groves	Area of sacred forest groves in each grid cell.	Continuous	3
	Cattle density	Density of cattle population in each grid cell.	Continuous	14

Table S16. AEZ-wise correlation analysis (2001 census) between average farm size and soil degradation (against two measures: erosion and salinization). All three variables: average farm size, soil erosion and soil salinity were ordered qualitative variables (Table S13). We used the Goodman-Kruskal gamma (g) statistic to measure the association between the ordinal variables. |g| = 1 indicates perfect correlation between the two variables. Negative g implies that soil erosion/salinization increases as farm size decreases, and vice-versa for positive gamma. All results reported at 95% confidence level from a one-sided test. Insignificant relationships are marked by hifen (‘-’). We did the analysis at 1km x 1km resolution, and each grid was weighed by the average number of farms (grid crop area/average farm size). AEZs with negative ‘g’ are regions where smaller farms are prone to higher soil degradation.

	Soil Erosion			Soil Salinity		
Spatial Domain	Goodman-Kruskal's gamma statistic (g)	Goodman-Kruskal's asymptotic standard error	z-value	Goodman-Kruskal's gamma statistic (g)	Goodman-Kruskal's asymptotic standard error	z-value
AEZ1	0.4733	0.0064	73.7361	-	-	-
AEZ2	0.4164	0.0002	2265.7084	0.4212	0.0003	1643.0032
AEZ3	0.0490	0.0005	106.5499	0.3622	0.0040	90.5521
AEZ4	-0.2270	0.0001	-1603.4043	0.0123	0.0001	84.7032
AEZ5	-0.1425	0.0002	-669.1248	0.1045	0.0003	301.3107
AEZ6	-0.1961	0.0001	-1505.6446	-	-	-
AEZ7	-0.0020	0.0002	-9.5359	-0.3545	0.0015	-240.0589
AEZ8	-0.0489	0.0002	-257.1600	-0.0803	0.0009	-89.5300
AEZ9	-0.1135	0.0004	-320.2257	-0.1488	0.0002	-658.9260
AEZ10	-0.1492	0.0002	-687.7616	0.1708	0.0003	489.6151
AEZ11	-0.0051	0.0004	-14.4712	-0.3381	0.0020	-170.2594
AEZ12	0.1987	0.0001	1460.7426	0.8416	0.0002	4183.3123
AEZ13	0.2800	0.0003	942.9212	-0.4896	0.0003	-1666.8518
AEZ14	-0.1759	0.0004	-424.8247	-0.3320	0.0018	-186.3924
AEZ15	0.2161	0.0003	755.6250	0.7128	0.0005	1471.9808
AEZ16	-0.0473	0.0014	-34.8690	0.8520	0.0018	473.0399
AEZ17	-0.2706	0.0008	-347.7385	-0.1786	0.0003	-137.8766
AEZ18	0.0810	0.0003	265.2050	0.5389	0.0006	916.3367
AEZ19	0.0155	0.0004	42.8529	0.4518	0.0009	501.7510
